# Characterization of ancestral Fe/Mn superoxide dismutases indicates their cambialistic origin

**DOI:** 10.1002/pro.4423

**Published:** 2022-09-21

**Authors:** Rosario Valenti, Jagoda Jabłońska, Dan S. Tawfik

**Affiliations:** ^1^ Department of Biomolecular Sciences Weizmann Institute of Science Rehovot Israel

**Keywords:** ancestral sequence reconstruction, cambialistic, evolution, metal specificity, oxygen, superoxide dismutase

## Abstract

Superoxide dismutases (SODs) are critical metalloenzymes mitigating the damages of the modern oxygenated world. However, the emergence of one family of SODs, the Fe/Mn SOD, has been recurrently proposed to predate the great oxygenation event (GOE). This ancient family lacks metal binding selectivity, but displays strong catalytic selectivity. Therefore, some homologues would only be active when bound to Fe or Mn, although others, dubbed cambialistic, would function when loaded with either ion. This posed the longstanding question about the identity of the cognate metal ion of the first SODs to emerge. In this work, we utilize ancestral sequence reconstruction techniques to infer the earliest SODs. We show that the “ancestors” are active in vivo and in vitro. Further, we test their metal specificity and demonstrate that they are cambialistic in nature. Our findings shed light on how the predicted Last Common Universal Ancestor was capable of dealing with decomposition of the superoxide anion, and the early relationship between life, oxygen, and metal ion availability.

## 1 INTRODUCTION

Life originated on our planet at least 3.4–3.2 billion years ago (giga‐annum or Ga).^8^, ^9^, ^10^, ^11^ Inarguably, the geochemical conditions that the first organisms experienced were very different from the ones surrounding extant life today. Life itself tremendously contributed to the transformation of the planet beyond recognition.^12^ For example, the emergence of oxygenic photosynthesis in the ancestors of cyanobacteria introduced molecular oxygen (oxygen hereafter) to the anoxic Archean atmosphere.^13^, ^14^

It is hypothesized that the first whiffs of biologically‐produced oxygen reacted with the reducing Archaean environment to finally accumulate and reach the atmosphere during the great oxygenation event (GOE) around 2.4 Ga.^15^, ^16^ The GOE was a double‐edged sword. Aside from the evident thermodynamic revolution, it posed a huge challenge for organisms unaccustomed to dealing with oxygen toxicity.^17^, ^18^, ^19^, ^20^ Moreover, metal ions such as Fe^2+^, used in a wide array of metalloproteins, and abundant in anoxic oceans, oxidized, and precipitated,^18^, ^21^, ^22^ contributing to the ecological catastrophe. However, for those life forms that eventually managed to tame it, oxygen unleashed huge amounts of energy and allowed impressive metabolic innovations, including the emergence of at least 700 new enzymatic reactions.^23^, ^24^

It has been continuously debated how life was able to survive and thrive through such a drastic change.^18^, ^25^ Among those lines, mechanisms for the generation of oxygen or its reactive species (ROS) on the Archaean Earth are continuously being discovered.^26^, ^27^, ^28^ Moreover, systems to deal with the toxic oxygen by‐products were proposed to be available to the Last Universal Common Ancestor (LUCA),^27^, ^29^, ^30^, ^31^ at the dawn of life, millions of years before the emergence of oxygen.

Superoxide anion (O_2_^˙−^) is the first product of the univalent reduction of oxygen. Due to its potential to initiate radical chain reactions and generate other ROS, O_2_^˙−^ is considered one of the most dangerous ROS.^17^, ^19^, ^32^ Superoxide dismutases (SODs) are enzymes that utilize a metal center to detoxify the superoxide anion by redox cycling (Equations 1a and 1b), thereby converting it to oxygen and hydrogen peroxide (H_2_O_2_).

(1a) $$O_{2}^{\cdot -}+H^{+}+\mathrm{SOD}\cdot M^{3+}\cdot {\mathrm{OH}}^{-}\to O_{2}+\mathrm{SOD}\cdot M^{2+}\cdot {\mathrm{OH}}_{2}$$

(1b) $$O_{2}^{\cdot -}+H^{+}+\mathrm{SOD}\cdot M^{2+}\cdot {\mathrm{OH}}_{2}\to H_{2}O_{2}+\mathrm{SOD}\cdot M^{3+}\cdot {\mathrm{OH}}^{-}$$

**FIGURE 1:**
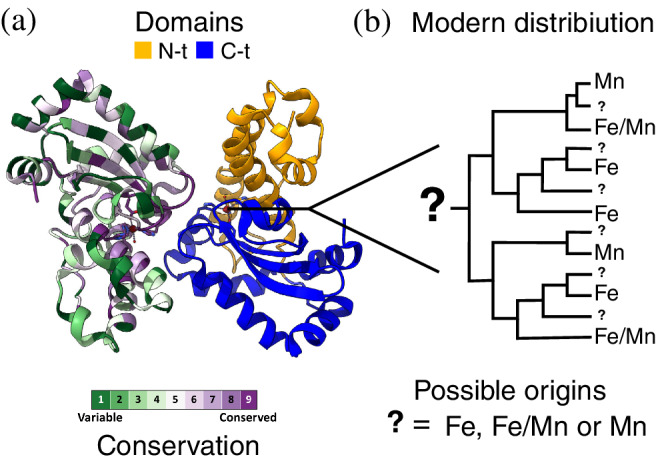
The Fe/Mn superoxide dismutase (SOD) family and the evolution of their metal utilization. Structure of a representative dimer from the Fe/Mn SOD family (a). One monomer is colored by conservation (green variable, purple conserved) while the other is colored by domains with the N‐ (orange) and C‐ (blue) terminal domains that form the fold indicated. The interface of the homodimer, as well as the contacts between domains and the active site, all display a high degree of conservation. Fe/Mn SODs are active when bound specifically to Fe (Fe‐SODs) or Mn (Mn‐SODs), with some able to function with either one (cambialistic or Fe/Mn‐SODs). The modern distribution of the metal specificities (b), for those proteins where it is known, shows a polyphyletic distribution. This distribution could have arisen from a Fe, Mn, or cambialistic ancestor. The protein model (PDB ID: 1D5N.pdb^1^) was generated using ChimeraX^2^ and conservation scores were calculated with ConSurf^3^

To date, three independently evolved SOD families are known to catalyze this reaction, each with a different metal center (M in equation 1): those with copper and zinc (Cu, Zn SOD),^33^, ^34^, ^35^ those with nickel (Ni SOD),^36^, ^37^ and those with iron or manganese (Fe/Mn SOD).^38^, ^39^, ^40^ Here we will focus only on the family of Fe/Mn SOD (hereafter just SOD), the first one to emerge. The Fe/Mn SOD family of enzymes is present across all kingdoms of life and has been recurrently dated back to LUCA.^29^, ^30^, ^31^ SODs are small (200 aa on average) and are well conserved in sequence and structure (Figure 1a). SODs are composed of two domains: an α‐helical N‐terminus and an α/β C‐terminus. Two residues from each domain form the active site contributing to the metal ion binding. Interestingly, it has been shown both in vitro and in vivo that the binding is unspecific, and that not only Mn and Fe, but also Ni, Zn, and cobalt (Co) can be incorporated into the active site.^41^, ^42^, ^43^

Despite their broad binding capacity, the metal specificity for SODs is a spectrum,^39^ with many examples of Mn‐specific SODs,^38^, ^41^, ^44^ Fe‐specific SODs^45^, ^46^ and enzymes that show activity with both metals, dubbed cambialistic.^47^, ^48^, ^49^ The reason for this range of specificities seems to lie in differential tuning of the redox potential of the metal ion in the active site.^40^, ^50^ Several nonconserved residues contribute to this tuning; therefore prediction methods for finding metal specificity based solely on the primary sequence have not given strong predictive values.^51^, ^52^ The distribution of metal specificities in contemporary SOD appears to be polyphyletic (Figure 1b),^39^ further complicating the understanding of the evolutionary history of the family.

Recently, our work has shown the power of utilizing phylogenetics to infer early geochemical conditions.^30^ We here extend this methodology to study the origin and specificity of the SOD family. Clarifying the evolutionary history of SODs would shed light not only on the environmental availability of the metal cofactor in the times of LUCA, but also, if active, on the need of early organisms to detoxify superoxide suggesting its prevalence in the biosphere prior to Earth oxygenation.

Traditionally, the first SODs have been considered to be Fe‐SODs. This assumption is based on the high availability of Fe^2+^ in the early biosphere,^53^ as supported by the high abundance of Fe‐dependent metalloproteins in the core proteome.^53^, ^54^, ^55^ Furthermore, the redox midpoint potential (Em) of Fe^2+/3+^ is close to the ideal value required for superoxide disproportionation.^40^ At a later stage, the oxidation and precipitation of Fe^2+^ from the oceans in the advent of oxygen^21^, ^56^ and the risk of Fe^2+^ reacting with this oxygen through the Fenton reaction,^53^ could apply evolutionary pressure for a transition to Mn‐SOD.

On the other hand, nonenzymatic disproportionation of superoxide by salts of Mn has been shown.^18^, ^57^, ^58^, ^59^ Today, several organisms utilize this mechanism to cope with oxidative stress.^58^, ^60^, ^61^, ^62^ Indeed, high concentrations of Mn alleviate the phenotype arising from the genetic deletion of SOD in yeast^63^ and bacteria.^64^ While originally the abundance of Mn in the Archaean ocean was debated,^65^, ^66^ newer studies suggest it could have been available for emerging life.^59^ Together these observations offer the alternative scenario of a Mn‐SOD as the founder of the Fe/Mn SOD family.

Finally, since the enzyme can bind both metal cofactors alike, and both cofactors were present in early oceans, the third possible scenario is an ancestrally cambialistic SOD. In this case, the posterior evolution of metal‐specific SODs could have derived from environmental changes in metal availability, for example, upon the GOE.^67^

To address the question of origin and metal specificity of the first SOD, in this study we used phylogenetic analysis and ancestral sequence reconstruction (ASR) to infer the sequences of early SODs dating back to LUCA and later nodes corresponding to the first whiffs of oxygen. We then expressed these ancestral SODs and characterized their activity and metal specificities. We found that the first SODs are indeed active against superoxide anion both in vivo and in vitro and are cambialistic. Together our findings suggest the evolutionary scenario for this family of enzymes.

## 2 RESULTS

### 2.1 Reconstruction of the early Fe/Mn SODs

**FIGURE 2:**
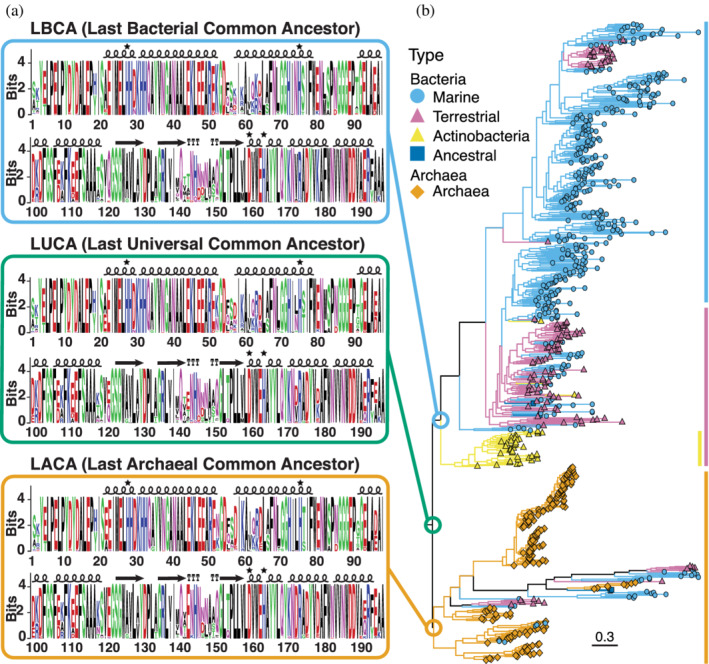
Probability logos for the earliest superoxide dismutases (SODs) and their phylogenetic tree. Probability logos (a) corresponding to the sequence reconstruction performed on the nodes LBCA (Last Bacterial Common Ancestor, light blue), LUCA (Last Universal Common Ancestor, green) and LACA (Last Archaeal Common Ancestor, orange) from a phylogenetic tree of SODs. The logos display high probabilities for residues located in the structured regions of the protein (*α*‐helices and *β*‐strands represented by loops and arrows above the sequence), and absolute conservation of the residues that coordinate the metal ion on the active site (stars). The phylogenetic tree (b) was constructed with all 707 Fe/Mn SOD sequences from 738 representative proteomes. The protein tree preserved the major splits of the species tree, namely a first split that largely separates bacterial and archaeal sequences (LBCA–LACA split); and some degree of separation between ancestrally terrestrial (pink) and marine (light blue) bacterial sequences. This resemblance is indicative of vertical inheritance, and it is compatible with a LUCA origin of the Fe/Mn SODs. Ancestral reconstruction was performed with PAML^5^ and the logos generated with WebLogo.^70^ The tree was generated by FastTree^4^ and midpoint rooted

In an attempt to understand the metal binding specificity of ancestral Fe/Mn SOD we sought out to infer what the sequence of such an ancestor would be. To do so, we used the concatenated Pfam Hidden Markov Models (HMM) profiles of present day N‐ and C‐terminal domains of Fe/Mn SODs (*SOD_Fe_N* and *SOD_Fe_C*).^68^ First we searched for members of these families using HMMsearch^69^ in 739 representative bacterial and archaeal proteomes (Table S1). The 707 identified sequences span all phyla analyzed. We constructed a phylogenetic tree of these sequences using FastTree^4^ (Figure 2b; see Section 4). The first split in the tree separates most bacterial and archaeal sequences (100% archaeal and 69.6% bacterial correctly assigned). Further on, the node that corresponds to the split between terrestrial and marine bacteria can be identified (with 89.3% of marine and 38.5% of terrestrial members correctly assigned). IQ‐Tree^71^ generated an equivalent tree.

As shown before,^30^ the node separating marine and terrestrial bacteria likely corresponds to the first sustained exposure of organisms to bioavailable oxygen. The broad distribution of the Fe/Mn SOD among prokaryotes (all phyla, 531 out of 738 species), its abundance, and the topology of the protein tree resembling the species tree, supports a LUCA origin for this family, as also reported previously.^30^, ^31^ Metal specificity, for the proteins where it is known, was polyphyletic, in line with other reports.^39^ We observed SOD paralogues in 148 out of 531 species containing SODs. Some of them cluster together on the tree, indicating possible gene duplication events, while most were dispersed, suggesting horizontal gene transfer (HGT) events. Sequences derived from *Actinobacteria* localize in a very basal split of the bacterial part of the tree, contrary to the position expected by their phylogeny (within the terrestrial clade). Curiously, *Actinobacteria* is one of the few phyla known to contain another class of SOD, Ni‐SOD.^37^ To address biases created by the presence of paralogs or the position of the *Actinobacteria* outliers, two alternative datasets were generated. Dataset S2, excluded *Actinobacteria* (File S1, 667 sequences), and Dataset S3, excluded all species that have more than one Fe/Mn SOD (potential paralogs; File S1, 383 sequences). Trees based on these datasets were generated (Figure S1). Overall, the topology of the tree across all three datasets is preserved, supporting the stability of the resulting tree.

Trees from Datasets [Link], [Link] and the corresponding alignments were used for the ASR, with two programs, PAML,^5^ and FastML.^6^ We decided to focus on the three main ancestral nodes: LUCA, the ancestor of all the sequences; LACA (Last Archaeal Common Ancestor); and LBCA (Last Bacterial Common Ancestor; see Figure 2b). The posterior probabilities logos for the ancestors of Dataset S1, reconstructed with PAML were high (Figure 2a, logos for all remaining datasets, nodes of interest, and FastML‐derived ancestors can be found in Figure S2), as can be expected from the highly conserved SOD sequences (Figure 1a). Overall, the marginal probabilities returned by PAML and FastML are almost identical. For Dataset S1, the most likely sequence corresponding to LUCA and LACA was identical; whereas for Dataset S3 the LUCA and LBCA most likely sequence are also the same. For this reason, in total 16 ancestors were further analyzed.

We expressed all 16 ancestors and characterized them. Here we focus mainly on the ancestors predicted by Dataset S1, specifically the ancestors reconstructed with PAML, but results obtained with the rest of the ancestors are reported in the supplement.

### 2.2 Ancestral SODs functionally complement the SOD‐null *Escherichia coli* mutant

To characterize the reconstructed ancestors we set out to express them in bacteria and measure their activity and metal specificity. First, a set of controls was chosen. The controls were the modern *E. coli* SODs: the Mn‐dependent SODa (WP_000122641.1) and the Fe‐dependent SODb (WP_000007283.1). The last control was the cambialistic SOD (CamSOD) from Staphylococcus sp. (WP_000874681.1), capable of displaying activity with both Mn and Fe at similar rates.^72^

**FIGURE 3:**
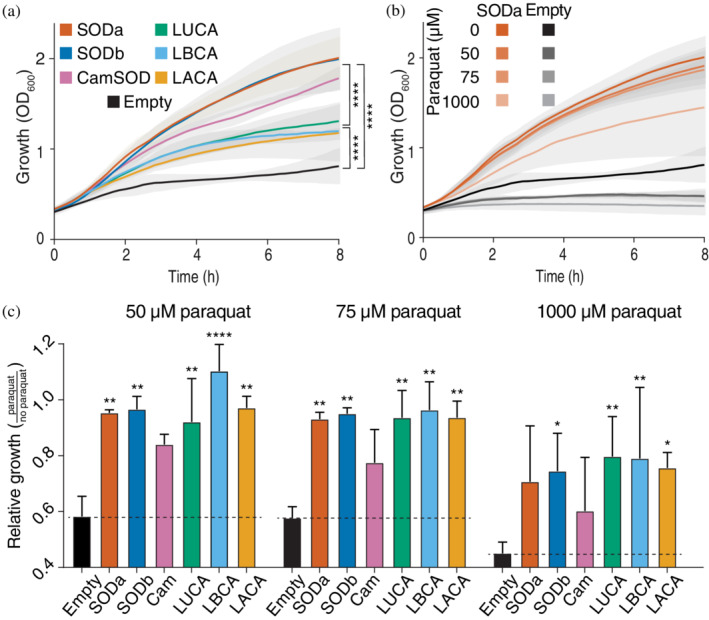
In vivo effect of superoxide dismutases (SODs). (a) Graph showing the aerobic growth of *E. coli* OX326a cells, devoid of Fe‐ and Mn‐SOD, and transformed with plasmids encoding the modern SODs: SODa (dark orange), SODb (dark blue), and CamSOD (pink); ancestral SODs: LUCA (green), LBCA (light blue), and LACA (light orange); or an empty plasmid (black). OX326a cells grew slowly in aerobic conditions when compared to cells expressing a modern SOD, while the cells with an ancestral SOD displayed an intermediate phenotype. (b) Example growth profile of the same cells when challenged with paraquat, which generates superoxide intracellularly. The growth was impaired as the concentration of paraquat increased, but the effect was higher in SOD null cells, especially clear at low concentration of paraquat. (c) Rescue of growth from paraquat toxicity in cells expressing ancestral and extant SODs. The ratio of maximum OD_600_ with and without paraquat treatment is shown for three different concentrations of paraquat. All the SODs, modern and ancestral, provided a degree of protection to the effect of paraquat when compared to SOD null cells. This protection was more evident in low concentrations of paraquat but was persistent throughout the conditions tested. Together, these experiments indicate that the ancestral SODs are active, though they still differ from their modern counterparts. All measurements were done in biological triplicates, with technical duplicates. In a and b, shading indicates SD, in c error bars represent SD. Statistical comparisons were done with two‐way ANOVA followed by Tukey's test in a, or Dunnett's multiple comparisons test to empty (displayed in the figure) and SODa (all nonsignificative) in c. **** represents *p*‐value < .0001; **p‐value < .01; and *p‐value < .05

To avoid interference produced by the native SODs present in *E. coli*, the strain OX326a was used. This strain, derived from K‐12 *E. coli* strain, has deletions in the genomic copies of SODa and SODb^73^ and presents slow growth in rich media under aerobic conditions.^64^, ^74^ Growth of OX326a cells expressing the different variants, as well as an empty plasmid, was monitored under aerobic conditions for 8 h under mild induction of the SODs (Figures 3a and S3). As expected, OX326a, devoid of SODs, exhibits a slow growth when compared to the same cells expressing the control SODs. The ancestors displayed an intermediate phenotype, growing faster than the cells with no SOD, but slower than those with extant SODs. The alternative ancestors (based on Dataset S2 or S3 or reconstructed with FastML) also displayed an intermediate phenotype (Figure S3), though in some cases the effect of adding the ancestral SODs was smaller.

To test whether the improved growth of OX326a cells expressing the control or the ancestral SODs was due to superoxide detoxification activity, the cells were challenged with increasing concentrations of paraquat (*N*,*N*′‐dimethyl‐4,4′‐bipyridinium dichloride). Paraquat produces O_2_^˙−^ intracellularly by redox cycling.^75^ This excess superoxide is toxic for the cells unless it can be processed by an active SOD. Therefore, cells exposed to growing concentrations of paraquat display increasingly impaired growth. For cells carrying a functional SOD, this decrease is less pronounced than for the OX326a cells without SOD (transformed only with an empty plasmid) as exemplified in Figure 3b. To test the activity of the ancestors in vivo, cells expressing each SOD in exponential growth phase were exposed to 0, 50, 75, or 1 mM paraquat and their growth was monitored by absorbance. For each construct and paraquat concentration, the rescue of growth from paraquat toxicity was calculated (Figure 3c). All the constructs provided significant protection against the toxicity of paraquat for the 50 and 75 μM conditions, except the CamSOD. For the alternative ancestors some degree of protection was observed, with their responses being significantly different from the cells with no SOD, and not significantly different from the cells expressing SODa (Figure S4), in line with the growth phenotype observed before.

### 2.3 In vitro activity measurements show the cambialistic nature of ancestral SODs

**FIGURE 4:**
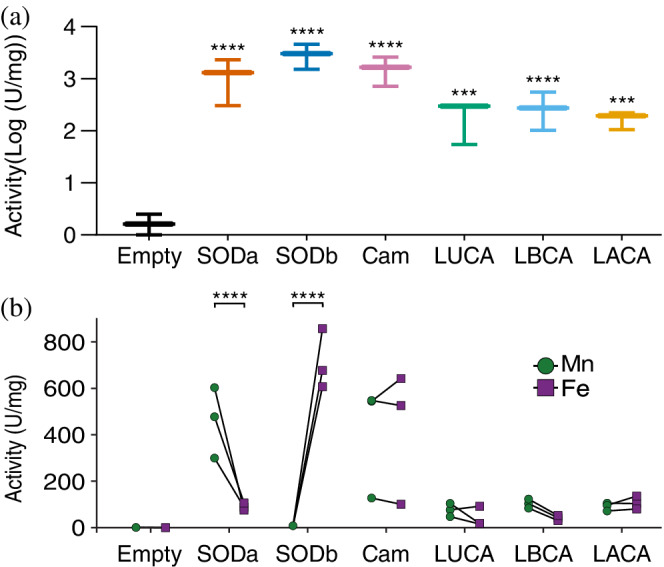
Superoxide dismutases (SODs) activity in lysate. Graphs showing the SOD activity in lysates from cells expressing SODs in rich media (a) or in minimal media (b) supplemented with Fe(II) or Mn(II) ions. Cells with no SOD displayed no activity, while the cells with the modern SODs tended to display higher activity than cells expressing ancestral SODs, in line with the in vivo observations. There is a large variability in the activity measured, due to differences in expression levels and metal availability. When metal ions were supplemented in minimal media, SODa showed higher activity with Mn, and almost none with Fe. For SODb the opposite was observed, as expected for both known metal specificities. Neither the CamSOD, as expected, or the ancestral SODs displayed a preference for one of the metals. This suggests that the first SODs were cambialistic. All measurements were carried in biological triplicates with technical duplicates. In a, bars represent min and max values. Activity measured from the same starter culture is linked by a line in b. The lysate activity was contrasted with Tukey's multiple comparisons test, while the metal preference was analyzed with Sidak's multiple comparisons test. **** represents *p*‐value < 0.0001 and ***p‐value < 0.001

The in vivo results showed that expressing the ancestors partially rescues the slow growth phenotype of SOD null cells, and protects the cells from the toxicity of paraquat, suggesting that they are active, and their metal specificity can be determined. To start characterizing them in more depth we measured SOD activity directly from the cell lysate. Cell lysate from OX326a, when transformed with an empty plasmid displayed no activity, as expected (Figure 4a). When the ancestor or control proteins were expressed in these cells, activity could be detected (Figure 4a). The activity of the ancestors was significantly higher than the background (*p*‐value <.001 in all cases). The alternative ancestors also displayed activity, following the same trend observed before: Dataset S1 > Dataset S3 > Dataset S2 (Figure S5).

To link the SODs activity to either manganese or iron, metal supplementation experiments were carried out. In this modification from the lysate assays, minimal media (M9) was used, to reduce the basal level of metals. As SODs have been shown to bind a mixture of metal ions in vivo,^43^, ^76^, ^77^ the M9 media was supplemented with an excess of Fe(II) or Mn(II) salts with the intent to bias metal content of the SODs. Then lysates were made and SOD activity was measured (Figure 4b). For SODa, Mn supplementation induced more activity than Fe, whereas for SODb the opposite was observed, matching their known metal specificities. CamSOD was also correctly identified in this assay as a cambialistic SOD, as it did not show any metal preference. Interestingly, all the ancestors displayed comparable activity with both metal ions (Figures 4b and S5) suggesting that they are cambialistic.

### 2.4 Metal replacement assays validate ancestral SODs as cambialistic

**FIGURE 5:**
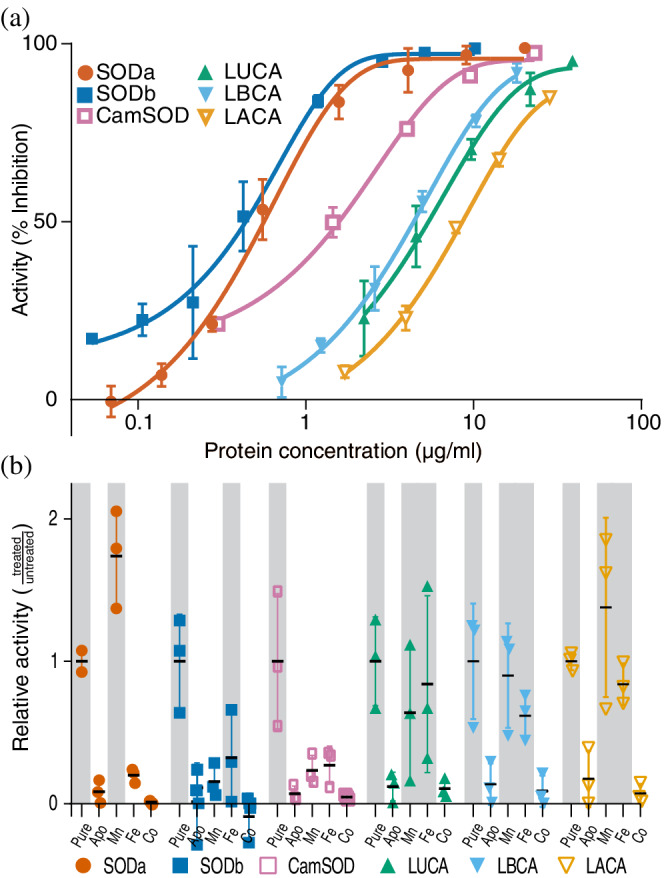
Superoxide dismutases (SODs) activity and metal reconstitution experiments. (a) Representative activity curves of purified SODs. The curves recapitulate the trends indirectly observed previously, with modern SODs, especially SODa and SODb displaying higher specific activity. (b) Relative activity levels of each protein when the metal ions were removed or replaced, compared to the purified, untreated proteins (Pure). Shading indicates an active condition. When the metals were removed (Apo), none of the proteins retained activity. The same was observed when Co was added, as expected for this metal ion that can bind but not confer activity. As expected, SODa only regained activity when bound to Mn. Despite tolerating the procedure poorly, the remaining SODb displayed activity only when bound to Fe, as expected. A trend was observed for CamSOD to recover activity with Fe and Mn, though the high variability on the activity of the pure protein probably prevented a clearer signal. All the ancestors displayed activity when bound to Fe and Mn, validating their cambialistic nature. All measurements were done in biological triplicates, with technical duplicates. Active conditions were determined by the lack of a significant difference in one sample *t* tests, assuming a theoretical mean of 1 (equal to that of the pure, untreated protein). In a, bars represent SD. In b, negative values were considered zero for display

The lysate experiments clearly suggest cambialistic ancestors, however, in lysate many other confounding factors exist, such as differences in the amount of protein expressed, the percentage of occupancy of the active sites, and the presence of other metals. Hence, to verify the lysate experiments in a more controlled setting we worked with the purified enzymes. For this, the modern proteins as well as the ancestors derived from Dataset S1 were purified from cells grown in rich media, without metal supplementation. As SODs function as dimers or tetramers, and heterodimers of SODa and SODb have been reported,^78^, ^79^ the protein purification was also carried out in OX326a cells, to avoid any contamination from endogenous SODs in the purified samples. Representative activity curves of the pure proteins show that SODa and b were the most active proteins, followed by the CamSOD and finally the ancestors (Figure 5a). This is in agreement with the results obtained on the in vivo and in lysate experiments and was consistent throughout biological replicates (Figure S6).

The purified proteins were stripped of metal ions to create the apo, inactive form. The apo form of the proteins retain very little to no activity (Figure 5b), in accordance with the lack of metal ions present as reported by inductively coupled plasma‐mass spectrometry (ICP‐MS) (Table S2). To promote the binding of specific metal ions to the active site of the apo proteins, aliquots of the proteins were incubated with mild heating in a buffer containing 50 μM of Fe, Mn, or Co ions.^80^ Cobalt was included as a negative control, as it has been shown to bind to the active site of SODs without conferring activity.^42^ The proteins loaded with the different metals were assayed for activity. In the case of SODa, only Mn restored the activity, whereas in the CamSOD similar levels of activity were observed with Mn or Fe. SODb shows almost no activity after being unfolded, with mild recovery with Fe. Finally, all the ancestors recovered activity with both Mn and Fe, corroborating their cambialistic nature. ICP‐MS measurements align with a complete occupancy of the active sites, with only the metal ion specified present in each case (Table S2).

### 2.5 Exploration of later nodes of the evolutionary tree of SODs reveal a Mn‐SOD coincident with predicted increase of intracellular oxygen

Since our ancestors were active and cambialistic, albeit with low activity compared to modern representatives, we decided to explore a more modern representative of the SOD lineage. We chose the ancestor belonging to the node of the Last Universal Oxygen Ancestor (LUOA), an ancestor proposed to represent the first organisms that started actively metabolizing oxygen.^30^ Only one sequence representing the LUOA was reconstructed and this gave rise to an active SOD, allowing OX326a cells to grow better that those with no SOD in anaerobic conditions (Figure S3) and conferring protection to paraquat (Figure S4). In lysate, the LUOA SOD displayed more activity than the earlier ancestors, and this activity was enhanced by Mn supplementation (Figure S5). The metal replacement experiments, performed as described before, validated the Mn specificity for this SOD (Figure S7).

## 3 DISCUSSION

In this work, we validated the LUCA origin of SODs through systematic phylogenetic analysis. We reconstructed ancestral sequences and characterized three most ancient nodes. The cells with ancestral SODs grew better and resisted the toxicity of paraquat more than cells devoid of SODs, though not to the same extent as when expressing modern SODs. We further corroborated the SOD activity against superoxide anion in lysates, specifically when supplementing for metal cofactors. This showed the cambialistic nature of the reconstructed first SODs, validated with in vitro experiments of metal removal and reconstitution. We finally performed a preliminary characterization of a more modern SOD, the one corresponding to the LUOA. The LUOA SOD showed higher activity than its ancestors, and Mn specificity.

Together, these results support the view that life and oxygen were always intertwined, at least enough to select for a LUCA that possessed an active SOD. It is important to notice that, though several sources of oxygen or ROS have been proposed to exist in early Earth,^26^, ^27^, ^28^ superoxide is unable to cross (at least modern) cellular membranes. This leaves open questions about the source of superoxide in LUCA. If superoxide was produced intracellularly, the enzymatic reactions or nonenzymatic processes that produced it may be identifiable.^81^, ^82^

Since the first split in the phylogenetic tree can be dated back only to LUCA, and as SOD has no outgroups, we could not explore instances more primitive than LUCA. Our first SODs were cambialistic, which in principle seems to be a less parsimonious scenario, since it would require to simultaneously tune the E_m_ potential of Fe and Mn appropriately. This might have derived from an environmental presence of both Fe and Mn, ions which are hard to distinguish,^83^, ^84^ which would select for an enzyme active regardless of the metal cofactor bound. It would be interesting to explore more nodes along the evolutionary tree of the SODs and characterize their metal cofactors. Since even today SODs have not evolved metal binding specificity, we would expect the transitions from cambialistic enzymes to Fe or Mn ones to be driven by an increase in specific activity. Concomitantly, transitions from non‐cambialistic to cambialistic activity should come with a trade‐off of specific activity (else a more active cambialistic enzyme would dominate in every scenario). Testing whether this logic stands throughout the evolutionary tree of SOD would help us understand if there are other factors at play. Toxicity of Fe through the Fenton reaction,^85^ the presence of paralogs,^39^ or the possibility of an SOD loaded with the non‐cognate metal having a peroxidase activity^86^, ^87^ have been proposed as external pressures adding to the selection of different SODs. Testing this hypothesis would aid our general understanding of the metalloproteins, but it would require assessing the activity of many more SOD sequences. As we showed a correlation between growth phenotype and enzyme activity, assaying huge numbers of SODs, both in rich media or media supplemented with a specific metal, could now be readily doable.

Early work by Carlioz et al. in 1988 already noted that a key residue for the hydrogen bond network in the active site, Gln 69 (SODb), is found in an analogous position in SODa, Gln 141 (from here on, residue numbers according to SODb, position 146 in SODa).^7^ Later works reiteratively found that this pair of residues represent the strongest signature for metal specificity identifiable in the primary sequence, and furthermore it is often replaced by His 141 in cambialistic SODs.^88^, ^89^ Accordingly, all the reconstructions, regardless of the dataset or software, displayed Gly at position 69 (Figure S2). At position 141, all LACA reconstructions predicted His, while for LBCA the same stands with the exception of the Dataset S2 (where actinobacteria are removed), where Gln is favored. The LUCA reflects the union of the above, with His in every case except in Dataset S2 where there was a comparable probability of His or Gln.

Finally, our single attempt to reconstruct the LUOA SOD portrayed a very active, Mn dependent enzyme. As LUOA corresponds to the first organisms actively metabolizing oxygen, the gain in activity is reassuring to find. It was also exciting to find a Mn‐SOD, since Fe^2+^ would have been scarce in the biosphere at the times of the LUOA and may have even been detrimental, given the Fenton reaction. A deeper exploration of this node, with several alternative ancestors, would be needed to further confirm these findings. Also, more experiments are required to determine if any slight preference for Mn or Fe can be readily detected in the ancestors we reconstructed, and if the trait depends on the node examined, dataset or software used. Regardless, our work indicates life was more prepared than previously thought to survive and thrive during the GOE.

## 4 MATERIALS AND METHODS

### 4.1 Identification of SOD sequences and ancestral sequence reconstruction

The Mn/Fe SOD has two domains (N‐ and C‐terminal) represented by two separate Pfam^68^ families (Sod_Fe_N and Sod_Fe_C, respectively). Using HMMsearch^69^ with Pfam‐defined gathering threshold, sequences containing both domains were identified in the set of 738 representative species spanning all the taxonomic prokaryotic families. The 707 sequences obtained belonged to all the phyla and 57 out of 59 classes present. The two classes without any SOD were the archaeal extremophiles Methanopyri (represented by a single species), and Thermococci, known to have superoxide reductases instead of SOD.^90^ The sequences were then aligned with Mafft (‐linsi option). The resulting alignment was trimmed with trimAl^91^ with the ‐gappyout option to remove positions where gaps dominate. Additionally, manual trimming was performed. Next, the phylogenetic tree using FastTree^4^ was calculated (‐pseudo ‐spr 4 ‐mlacc 2 ‐slownni parameters and the Jones–Taylor–Thornton [JTT] evolutionary model). An equivalent tree was obtained with IQ‐TREE^71^ (with ModelFinder + tree reconstruction + ultrafast bootstrap [1000 replicates] and WAG+I+G4 evolutionary model). No related proteins, therefore no obvious outgroups with activity other than SOD could be identified, so the tree was rooted in two ways: with midpoint and minimal ancestor deviation (MAD) software^92^ (File S1). The alignment and trees were then used as inputs for ASR with FastML^6^ and PAML For the reconstruction with PAML, marginal probabilities were used, while for FastML we took advantage of the joint probabilities function.^93^ The posterior probability logos for each reconstruction were generated with WebLogo.^70^

#### 4.1.1 DNA and cloning

pET29b(+) vectors encoding the sequences of the ancestral reconstructions and the cambialistic SOD were obtained from Twist Biosciences. Each variant was tagged on the C‐terminal end with a Strep‐tag II sequence^94^ to allow easy affinity purification. The sequences were subsequently subcloned into pET21b(+) vectors by standard PCR amplification and Gibson Assembly and validated by sequencing. The plasmids encoding SODa and SODb were taken from the ASKA collection.^95^ These encoded an N‐terminal His‐Tag.^96^ Strep‐tag II was preferred over His‐Tag for the rest of the sequences to avoid interferences in the metal reconstitution experiments produced by the metal affinity of the His‐Tag. Both N‐ and C‐ terminal tagging with small peptides has been shown not to perturb the activity or oligomerization of these enzymes.^97^, ^98^, ^99^ All plasmid sequences utilized in this work are listed in Table S3.

#### 4.1.2 Strains

The *E. coli* strain OX326a^73^ in which both chromosomal Mn/Fe SODs genes have been inactivated (SODa and SODb, respectively), kindly provided by Sarel Fleishman was used throughout the study. This strain carries a genomic kanamycin resistance marker and grows slower in aerobic conditions, with a higher tendency for mutations than the paternal K‐12 strain it was generated from References 64, 73, 100. OX326a was transformed with plasmids encoding each SOD or an empty plasmid by electroporation. A glycerol stock of the transformed cells was generated, from which fresh cultures were initiated for every experiment.

#### 4.1.3 Growth and paraquat response experiments

The protocol for growth and paraquat response experiments was adapted from methods in enzymology 349.^101^ Briefly, precultures of each strain grown in LB with appropriate antibiotics (kanamycin and chloramphenicol ‐SODa and SODb‐ or kanamycin and ampicillin ‐the rest‐) were grown to OD_600_ of 1, cooled on ice and diluted in prewarmed media to OD_600_ of 0.1 with or without induction (0.1 mM of Isopropyl *ß*‐d‐1‐thiogalactopyranoside [IPTG]). After duplication, 200 μl of each culture was deposited in a well of a 96‐well plate and challenged with 0, 50, 75, or 1,000 μM paraquat. The well was topped with parafilm oil to prevent evaporation. Growth was monitored every 10 min for 8 h using a multiwell plate reader (Eon, Biotek Gen5) while continuous shaking and 37°C were maintained. Each condition was measured in three biological replicates, with technical duplicates. To analyze growth, the whole OD_600_ curves were used. Data were analyzed by two‐way ANOVA followed by Dunnett's multiple comparisons test, contrasting each curve of the dataset to the one of Empty and of SODa. Results are explained in Figure S3 legend. Tukey's multiple comparisons test was also performed in the constructs displayed in Figure 3a. To analyze the paraquat data, the maximum value of OD_600_ from each curve was taken and normalized by the corresponding value of maximum OD_600_ in the samples with no paraquat. We performed two‐way ANOVA followed by Dunnett's multiple comparison test against SODa and Empty, for each concentration of paraquat. All significant differences are plotted in Figures 3c and S4.

#### 4.1.4 Lysate experiments

Lysates in rich media (LB) and in minimal media (M9) were prepared in parallel, from the same starter cultures, though the cells for the M9 cultures were washed three times with 1× sterile PBS to remove excess metals. M9 was supplemented with 0.34 mM Fe(II) or Mn(II).^102^ For the metal supplementation, high purity (>99%) Sigma ammonium iron(II) sulfate or manganese(II) sulfate were used. Glucose was used as the carbon source. Induction was done at OD_600_ 0.4–0.6 with 1 mM IPTG for 5 h at 37°C. Cells were collected by centrifugation, stored at −20°C and lysed by bath sonication right before use. Typically, 25 ml of culture were used and the pellet was resuspended in 1 ml of lysis buffer (20 mM TrisHCl, 100 mM NaCl, 1 mg/ml lysozyme, 0.1 μl/ml benzonase and EDTA‐free protease cocktail inhibitor [1:200, Abcam], pH 7.8). The lysate was cleared by centrifugation and protein concentration was determined using the Pierce BCA Protein Assay Kit (Thermo Fisher Scientific). Activity was determined as explained below. Data analysis was done with ordinary one‐way ANOVA followed by Tukey's multiple comparisons test. Differences with Empty are plotted (Figures 4a and S5a). For ancestors derived from Dataset S1, no other significant difference was observed. Most of the ancestors derived from Dataset S2 present differences with the modern SODs. The data of lysate with metal supplementation was analyzed by two‐way ANOVA followed by Sidak's multiple comparison test to contrast the activity conferred by Mn and Fe in each case. Only SODa, SODb, and LUOA showed significant differences, as presented in Figures 4b and S5b.

#### 4.1.5 Protein expression and purification

Protein expression was carried out in the same way as the lysate experiments, with the difference that 250 ml of 2xYT were used each time. The frozen pellets were resuspended in 10 ml of lysis buffer and allowed to thaw for 10 min at room temperature (RT) before freezing again. These freezing cycles improved lysis efficiency. After sonication and lysate clarification by high‐speed centrifugation, the lysates were applied to high affinity columns. For SODa and SODb the resin used was HisPur™ Ni‐NTA Superflow Agarose (ThermoFisher), while for the rest Strep‐Tactin® Sepharose® resin (IBA). In each case, the proteins were eluted in 12 ml of elution buffer (Tris 20 mM, NaCl 100 mM, pH 7.8, containing imidazole 150 mM or desthiobiotin 5 mM). Protein concentration was measured as before, prior removal of imidazole from the SODa and SODb samples with a desalting column (PD10, GE Healthcare). Proteins were kept at 4°C until utilization without observed changes in activity.

#### 4.1.6 Activity measurements

SOD activity was measured with the SOD Assay Kit (Sigma‐Aldrich). The amount of xanthine oxidase added to start the reaction was calibrated to produce an initial increase in absorption at 450 nm of 0.013–0.015 units in the absence of SOD.^103^ The slopes of the absorbance increase at 450 nm were used for calculations instead of the final absorbance values. Every measurement was done by duplicates in independent plates. Under these conditions, 1 unit of activity was defined as the amount of protein necessary to generate a 50% inhibition of the color formation in the condition with no SOD. As the reactions were carried out in 0.2 ml, factor of 5 was introduced to refer this amount to 1 ml of reaction. From this value and the protein concentration, the number of units per mg of protein is derived.

#### 4.1.7 Activity curves

For each of the pure proteins and each of the three biological replicates, five serial dilutions by half were chosen to land within the range of maximum sensitivity of the activity assay (ranging from 0.05 to 20 μg/ml final concentration depending on the protein) and activity was measured as explained before. The model of log(agonist) versus response from Graphpad Prism (version 8.0.1 for Windows) was fitted to the data and the parameters were utilized to determine the U/mg of protein.^101^, ^104^ The activity of each protein (U/mg) was contrasted by one‐way ANOVA followed by Tukey's multiple comparison test. Results from this test are explained in Figure S6 legend.

#### 4.1.8 Metal replacement experiments

For the metal removal and replacement experiments, several protocols were combined.^41^, ^72^, ^101^ Pure protein at 0.2–0.1 mg/ml was dialyzed against unfolding buffer (2 M GnHCl, 10 mM 8‐hydroxyquinoline, 10 mM EDTA, pH 3.8) at RT, overnight with soft agitation. Next, half of the buffer was removed and replaced by refolding buffer (20 mM TrisHCl, 100 mM NaCl, pH 7.8) and sucrose was added for a final concentration of 0.5 M. These intermediate unfolding conditions were sustained overnight and a final buffer exchange, to refolding buffer with 0.1 mM EDTA was performed and also sustained overnight. Together, this protocol generates protein unfolding, the chelation of the metal contained by the active site, and a subsequent refolding. The presence of chelators throughout the process facilitates the generation of the apo protein, as the binding constant to different metals has been described as weaker than the *K*_a_ of chelators, metal affinity being mainly determined by kinetics.^105^ In some cases, some precipitation was observed (i.e., very pronounced for SODb, in accordance with literature^46^, ^79^, ^106^), but not in the case of the ancestors derived from the reconstructions of the first dataset. Extensive centrifugation was performed on the final proteins to remove any aggregates. When needed, protein was concentrated with the utilization of a centricon (Amicon 10 kDa Merck Millipore) after refolding. The ApoSODs were desalted by gel filtration (PD10, GE Healthcare) into a buffer previously stripped of metals by the utilization of a Chellex column (Bio‐Rad). Protein concentration was determined using the Pierce BCA Protein Assay Kit (Thermo Fisher Scientific). Subsequently, the same preparation of ApoSOD was divided into four aliquots that were heated in the presence of 50 μM Mn, Fe, Co, or no metal ions, at 45°C^80^ for 1 h, and activity was measured as mentioned above. We determined with blanks that 50 μM metal ions did not interfere with the assay measurements, so the excess metal ions were not removed before activity determination. The salts used were the same as for lysate supplementation experiments for Mn and Fe, and Cobalt(II) sulfate heptahydrate, ReagentPlus®, ≥99% (Sigma) for Co. All the buffers were evaluated with ICP‐MS for purity. For each SOD and condition, the activity measured was normalized to the average protein activity of the pure protein (derived from the activity curves). Active conditions were identified by performing one sample *t* test considering a theoretical mean of 1 (equivalent to the activity of the pure, untreated protein). Samples that did not differ from this hypothetical mean were deemed active. Accordingly, all pure, untreated proteins were active, and all apo or Co‐loaded proteins were inactive. The rest of the results are displayed in Figures 5b and S7.

#### 4.1.9 Inductively coupled plasma mass spectrometry (ICP‐MS)

The Fe and Mn content of samples from the reconstitution experiments was measured. Excess metal ions were removed by multiple cycles of concentration and dilution with MS grade water (Bio‐Lab) in Amicon 10 KDa (Merck Millipore) centricons. Protein concentration was determined using the Pierce BCA Protein Assay Kit (Thermo Fisher Scientific). The Mn and Fe content was measured by ICP‐MS on the Agilent 7700 s in MS/MS mode with In as the internal standard to correct for instrumental drift and a He gas collision cell to avoid interferences. The most abundant isotopes of manganese and iron (i.e., Mn55 and Fe56) were used. Calibration standards were prepared by dilution of certified ICP elemental standards (Inorganic Ventures) in 1% (v/v) HNO_3_. Samples were also diluted to 1% HNO_3_ and ultracentrifugated to remove debris to closely match the standard matrix and avoid interferences from the organic content. The Agilent MassHunter software was used for ICP‐MS data analysis. The metal content was normalized to the protein concentration and expressed in units of atoms per active site Table S2.

#### 4.1.10 Data analysis

Statistical data analysis was performed using GraphPad Prism version 8.0.1 for Windows, GraphPad Software, San Diego, CA, www.graphpad.com. In all cases, three separate cultures (biological triplicates) were grown, and two measurements (technical duplicates) were made in each culture.

## AUTHOR CONTRIBUTIONS

**Rosario Valenti:** Conceptualization (equal); formal analysis (equal); investigation (equal); methodology (equal); project administration (equal); supervision (equal); validation (equal); visualization (lead); writing – original draft (equal). **Jagoda Jabłońska:** Conceptualization (equal); formal analysis (equal); investigation (equal); methodology (equal); project administration (equal); writing – original draft (equal). **Prof. Dan Tawfik:** Conceptualization (equal); funding acquisition (lead); methodology (equal).

## Supporting information


Dataset S1

Dataset S2

Dataset S3

**Figure S1** Alternative phylogenetic trees of Fe/Mn SODs. Alternative phylogenetic trees constructed with subsets of the Fe/Mn SOD sequences to control for the abnormal localization of the *Actinobacteria* (yellow) phyla on the tree (a), Dataset S2—no *Actinobacteria*, 667 sequences; or for the presence of paralogs (b), Dataset S3—no paralogs, 383 sequences. Both trees retain the main features observed when utilizing all the sequences, Dataset S1—nothing excluded, 707 sequences (Figure 1b); namely the split between archaeal (orange) and bacterial sequences and the partial split between sequences belonging to ancestrally marine (light blue) and terrestrial (pink) bacteria. The trees were constructed with FastTree^4^ and midpoint rooted.

**Figure S2** Probability logos for the sequences of all the nodes reconstructed. Probability logos corresponding to the sequence reconstruction performed on the LUCA (Last Universal Common Ancestor), the LBCA (Last Bacterial Common Ancestor), and the LACA (Last Archaeal Common Ancestor) nodes for Dataset S1 (nothing excluded, 707 sequences), Dataset S2 (no *Actinobacteria*, 667 sequences) and Dataset S3 (no paralogues, 383 sequences), with PAML^5^ or FastML.^6^ All the logos display high probabilities, mostly for the regions where a secondary structure element is present (*α*‐helices and *β*‐strands represented by loops and arrows above the sequence), and absolute conservation of the residues that coordinate the metal ion on the active site (stars). Positions suggested to be relevant for the metal specificity are marked with inverted triangles.^7^ Especially high similarity is seen for the same node and dataset, irrespective of PAML or FastML reconstruction. This was expected for such a conserved protein as the Fe/Mn SOD (see Figure 1), and two maximum likelihood‐based programs.

**Figure S3** Comparative growth of cells expressing different ancestral SODs. Graphs showing the growth of *E. coli* OX326a cells, devoid of Fe‐ and Mn‐SOD, and transformed with plasmids encoding the different sets of reconstructed ancestors (LUCA in green, LACA in light orange and LBCA in light blue). Cells transformed with a plasmid encoding SODa (dark orange) or an empty plasmid (black) are presented as guidance. In the control panel, cells transformed with plasmid encoding SODa or SODb, but uninduced (pink and dark blue), or cells expressing the LUOA ancestor (yellow) are displayed. All ancestors significantly differ in their growth phenotype from SODa and Empty. Reconstructions from Dataset S1 tend to confer better growth than those from Dataset S3, and them from Dataset S2. Cells encoding SODa and SODb, even when uninduced, grew indistinguishable from the SODa control, probably indicative of some leaky expression. Dunnett's multiple comparisons test: all but SODa and SODb uninduced display a significant difference in growth to SODa (*p*‐value < .0001 for all comparisons). When compared to empty, the same was obtained, except for the comparison with LBCA from Dataset S2, reconstructed with PAML and with FastML, that showed a smaller difference (*p*‐value < .05). All measurements were done in biological triplicates, with technical duplicates. Shading indicates SD of each curve.

**Figure S4** Paraquat effect in the growth of cells expressing different ancestral SODs. Quantification of the relative growth of *E. coli* OX326a cells, devoid of Fe‐ and Mn‐SOD, and transformed with plasmids encoding the different sets of reconstructed ancestors (LUCA in green, LACA in light orange and LBCA in light blue) and exposed to different levels of paraquat. Cells transformed with a plasmid encoding SODa (dark orange) or an empty plasmid (black) are presented as guidance. As controls, cells transformed with plasmids encoding SODa and SODb but uninduced (pink and dark blue) are displayed together with the LUOA ancestor (yellow). There is a trend for all the SODs to confer a certain degree of protection to paraquat, clearer at low doses. Dataset S2 differentiated less from cells with no SOD. Statistical significance to empty (as determined by Dunnett's test) is represented above the bars, while those that differ from SODa are indicated with lines. Measurements done in biological triplicates, with technical duplicates. Bars represent SD. *** represents *p*‐value < .001; **p‐value < .01; and *p‐value < .05.

**Figure S5** SODs activity in lysate. Graphs showing the SOD activity in lysates from cells expressing the different ancestors in rich media (a) or in minimal media (b) supplemented with Fe(II) or Mn(II) ions. Cells transformed with an empty plasmid (black) or with SODb (blue) are shown for guidance. Cells with no SOD displayed no activity, while all the ancestors displayed activity. There is a large variability in the activity measured, due to differences in expression levels and metal availability. When metal ions were supplemented in minimal media, all the ancestors but LUOA displayed comparable activity irrespective of the metal ion added. LUOA showed a preference for Mn, contrary to SODb, a Fe‐SOD. This suggests that the first SODs were cambialistic in nature. All measurements were carried in biological triplicates with technical duplicates. In a, bars represent maximum and minimum values. The lysate activity was contrasted with Tukey's multiple comparisons test, though only differences with empty are displayed. Modern SODs showed differences with ancestors reconstructed from Dataset S2. Particularly, SODb, displaying the higher activity, differed from LUCA P2, LBCA P2 and F2 (*p*‐value < 0.001); LUCA F2, LACA P2 and F2 (*p*‐value < 0.01); and LBCA F1 and LACA P3 (*p*‐value < 0.05). The metal preference was analyzed with Sidak's multiple comparisons test. **** represents *p*‐value < 0.0001 and ***p‐value < 0.001.

**Figure S6** SODs activity. Repeats of the activity curves of purified SODs from three independent purifications. Protein concentration was determined using the Pierce BCA Protein Assay Kit (Thermo Fisher Scientific). The curves were generated by fitting the model log(agonist) versus response from GraphPad Prism (version 8.0.1) to the data. Curves recapitulate the trends indirectly observed previously, with modern SODs, especially SODa and SODb displaying higher specific activity. Bars represent SD of measurements. From the parameters of the model, the activity (U/mg) of each SOD was determined. When analyzed by ordinary one‐way ANOVA, followed by Tukey's multiple comparisons, only SODa and SODb displayed significant differences with all the other SODs, but not between themselves. Differences with CamSOD and LUOA were small (*p*‐value <0.05 SODa, <0.01 SODb) and more pronounced with the rest of the ancestors (to all, SODa showed *p*‐value <0.001 and SODb <0.0001).

**Figure S7** Metal reconstitution experiments. Relative activity levels of ancestors from Dataset S1, reconstructed with FastML and the LUOA ancestor when the metal ions were removed or replaced, compared to the purified, untreated proteins. Shading indicates an active condition. When the metals were removed, none of the proteins retained activity. The same was observed when Co was added, as expected for this metal ion that can bind but not confer activity. LUCA/LACA and LBCA displayed activity when bound to Fe and Mn, validating their cambialistic nature. LUOA only recovered activity when bound to Mn. All measurements were done in biological triplicates, with technical duplicates. Active conditions were determined by the lack of a significant difference in one sample *t* tests, assuming a theoretical mean of 1 (equal to that of the pure, untreated protein).

**Table S1** List of species used for the phylogenetic study

**Table S2** Metal content in purified SODs, after metal removal and reconstitution, measured by ICP‐MS

**Table S3** List of constructs utilized in this work
