# Supplementary material for: Characterization of ancestral Fe/Mn superoxide dismutases indicates their cambialistic origin
Source: Protein Sci. 2022 Sep 21;31(10):e4423. doi: 10.1002/pro.4423 (PMC9490801; doi:10.1002/pro.4423)
Supplement: Supplementary file 1 — Dataset S1 [file PRO-31-e4423-s009.zip › PRO_4423_Dataset1_PhylogeneticTree.docx]

(((((A0A345UFZ3_1457365_Bacteroidetes_B_Mar:0.54476000,(A0A2Z4FGN2_1548548_Proteobacteria_B_Mar:0.48428000,(Q0AW95_335541_Firmicutes_B_Ter:0.36619000,((A0A0B7MKY0_499207_Firmicutes_B_Ter:0.24089000,A0A259UH52_1123289_Firmicutes_B_Ter:0.36516000):0.26836000,(A0A089IHE8_1536775_Firmicutes_B_Ter:0.23999000,(A8FE63_315750_Firmicutes_B_Ter:0.38712000,(A0A2T6C4K2_1242148_Firmicutes_B_Ter:0.14035000,Q67T03_292459_Firmicutes_B_Ter:0.28962000):0.04397000):0.03554000):0.14746000):0.10404000):0.07928000):0.10857000):0.13343000,(A0A0K1PI80_1391653_Proteobacteria_B_Mar:0.67002000,((((((A0A2B7GPY0_1608465_Halobacteria_A_Arc:0.21025000,A0A2R4X0R1_1679096_Halobacteria_A_Arc:0.14830000):0.03801000,((A0A1G8W4P7_890420_Halobacteria_A_Arc:0.05190000,(U1MGY5_1325472_Halobacteria_A_Arc:0.11982000,U1QN78_1085028_Halobacteria_A_Arc:0.08676000):0.13674000):0.05181000,(A0A365TEZ3_29295_Halobacteria_A_Arc:0.09803000,((A0A1Q1FM06_1932360_Halobacteria_A_Arc:0.02365000,(A0A1U7EY38_348780_Halobacteria_A_Arc:0.03213000,M1XSP2_268739_Halobacteria_A_Arc:0.04026000):0.02817000):0.05132000,R4W6K3_1333523_Halobacteria_A_Arc:0.08739000):0.01270000):0.02700000):0.01537000):0.03147000,(((R4W031_1333523_Halobacteria_A_Arc:0.10314000,F7PIA8_1033806_Halobacteria_A_Arc:0.11335000):0.02042000,(A0A1G9VZ43_996166_Halobacteria_A_Arc:0.03751000,((A0A202E7D5_253108_Halobacteria_A_Arc:0.06165000,((A0A3N6LM69_1679091_Halobacteria_A_Arc:0.01324000,((D3SY04_547559_Halobacteria_A_Arc:0.01739000,(A0A2Z2HUJ4_745377_Halobacteria_A_Arc:0.02184000,L0AKN7_797304_Halobacteria_A_Arc:0.07620000):0.01604000):0.01823000,A0A3N6M5P3_1679083_Halobacteria_A_Arc:0.01113000):0.01442000):0.00465000,(((M0L696_358396_Halobacteria_A_Arc:0.01251000,M0M5P3_1227454_Halobacteria_A_Arc:0.02603000):0.01543000,((A0A063ZJ32_1495067_Halobacteria_A_Arc:0.04807000,W0JR24_797299_Halobacteria_A_Arc:0.00625000):0.02535000,(M0CBS2_1230457_Halobacteria_A_Arc:0.05231000,(L0JLZ0_797303_Halobacteria_A_Arc:0.01327000,((A0A1H9BZT2_1186196_Halobacteria_A_Arc:0.00476000,(D2RVX0_543526_Halobacteria_A_Arc:0.01073000,((A0A1I0M6P7_1202768_Halobacteria_A_Arc:0.00055000,F8D6A5_797210_Halobacteria_A_Arc:0.06340000):0.00055000,A0A4P8WH38_88724_Halobacteria_A_Arc:0.02755000):0.00554000):0.02390000):0.01392000,(A0A2B7GK67_1608465_Halobacteria_A_Arc:0.01381000,L9ZI72_1227494_Halobacteria_A_Arc:0.02496000):0.01069000):0.00717000):0.02526000):0.02300000):0.00050000):0.01403000,((L0JX01_694430_Halobacteria_A_Arc:0.08900000,M0AWE7_29540_Halobacteria_A_Arc:0.09959000):0.05944000,((A0A1N7EDL3_588898_Halobacteria_A_Arc:0.01641000,L9WKK3_1227499_Halobacteria_A_Arc:0.02236000):0.00537000,(L9VRX1_1114856_Halobacteria_A_Arc:0.01091000,(L0IC00_797302_Halobacteria_A_Arc:0.04891000,L9WFH7_1230460_Halobacteria_A_Arc:0.02640000):0.00780000):0.00542000):0.00053000):0.00515000):0.00930000):0.02055000):0.05504000,D8J2G3_795797_Halobacteria_A_Arc:0.09453000):0.01416000):0.01621000):0.02367000,(((A0A3P3RB00_671145_Halobacteria_A_Arc:0.15879000,(M0MM71_1227455_Halobacteria_A_Arc:0.02801000,(M0MDJ5_931277_Halobacteria_A_Arc:0.02454000,M0M2F6_1132509_Halobacteria_A_Arc:0.07191000):0.02448000):0.03968000):0.01892000,(((A0A1H6J821_1267564_Halobacteria_A_Arc:0.08848000,W0K3A4_751944_Halobacteria_A_Arc:0.06825000):0.01859000,((A0A0F7PDN8_1604004_Halobacteria_A_Arc:0.13299000,(A0A0U5H1X7_1407499_Halobacteria_A_Arc:0.02862000,(A0A0U5CYX4_1407499_Halobacteria_A_Arc:0.06971000,((W0K3U0_751944_Halobacteria_A_Arc:0.05203000,(A0A1G9XX20_996166_Halobacteria_A_Arc:0.15642000,M0CQV9_797114_Halobacteria_A_Arc:0.12948000):0.05539000):0.01527000,(((A0A4P8WMJ7_88724_Halobacteria_A_Arc:0.05568000,(A0A3M9JL82_1853682_Halobacteria_A_Arc:0.01350000,(A0A3M9JV35_1853682_Halobacteria_A_Arc:0.02646000,U2YRD9_1261545_Halobacteria_A_Arc:0.03238000):0.06455000):0.06650000):0.02464000,(((A0A1G9XG20_660521_Halobacteria_A_Arc:0.05408000,((A0A0W1RIP5_1544718_Halobacteria_A_Arc:0.03026000,((I3R8L9_523841_Halobacteria_A_Arc:0.02092000,I3RBB8_523841_Halobacteria_A_Arc:0.00054000):0.04870000,(Q03300_309800_Halobacteria_A_Arc:0.00054000,(A0A0W1SLK0_1544718_Halobacteria_A_Arc:0.03685000,Q03301_309800_Halobacteria_A_Arc:0.00055000):0.01069000):0.00619000):0.02493000):0.01475000,(((A0A1V4C128_1853690_Halobacteria_A_Arc:0.00555000,A0A1V4C962_1853690_Halobacteria_A_Arc:0.01019000):0.02578000,(A0A0N8HZX4_699431_Halobacteria_A_Arc:0.01664000,A0A0P7GPP0_699431_Halobacteria_A_Arc:0.04882000):0.02034000):0.04097000,(((A0A2I8VJM1_755307_Halobacteria_A_Arc:0.00054000,A0A2I8VJQ7_755307_Halobacteria_A_Arc:0.01015000):0.05962000,A0A3P3RGH5_671145_Halobacteria_A_Arc:0.05314000):0.02030000,((((A0A345E419_1547899_Halobacteria_A_Arc:0.01594000,A0A368N5W7_1126245_Halobacteria_A_Arc:0.04779000):0.01265000,(A0A1H3W6N9_555874_Halobacteria_A_Arc:0.01059000,E4NT27_469382_Halobacteria_A_Arc:0.04410000):0.01444000):0.01715000,((U1QK35_1070774_Halobacteria_A_Arc:0.08146000,Q18HG3_362976_Halobacteria_A_Arc:0.11204000):0.04094000,((A0A1H3ILX3_660517_Halobacteria_A_Arc:0.02699000,A0A1H6AL08_699433_Halobacteria_A_Arc:0.02508000):0.00581000,(A0A2G1X3V2_1483399_Halobacteria_A_Arc:0.02186000,((A0A238VTU4_63740_Halobacteria_A_Arc:0.04865000,A0A256IHQ1_1383851_Halobacteria_A_Arc:0.00519000):0.01037000,((A0A081EVB3_2248_Halobacteria_A_Arc:0.00055000,A0A0F8D555_2248_Halobacteria_A_Arc:0.00520000):0.03783000,(A0A2G1X713_1483399_Halobacteria_A_Arc:0.00529000,(V6DS43_1173487_Halobacteria_A_Arc:0.01094000,V6DRS8_1173487_Halobacteria_A_Arc:0.02682000):0.00513000):0.01081000):0.00788000):0.01738000):0.02818000):0.02070000):0.02915000):0.02364000,(A0A1G9SJT8_660521_Halobacteria_A_Arc:0.04166000,((A0A1H6VKK8_1073996_Halobacteria_A_Arc:0.08976000,A0A0W1R4R4_1514971_Halobacteria_A_Arc:0.04347000):0.02264000,(M0DHS9_1227487_Halobacteria_A_Arc:0.04296000,V4HNI7_1324957_Halobacteria_A_Arc:0.08781000):0.03479000):0.02432000):0.01452000):0.00626000):0.02669000):0.00890000):0.02461000):0.02256000,F8DDU6_797210_Halobacteria_A_Arc:0.00911000):0.02277000,A0A285P0H4_558529_Halobacteria_A_Arc:0.02100000):0.00538000):0.00873000,(C7P4U1_485914_Halobacteria_A_Arc:0.05887000,M0B3T7_29540_Halobacteria_A_Arc:0.04550000):0.02357000):0.01642000):0.03484000):0.06580000):0.04972000):0.05407000,(A0A1I0MG92_355548_Halobacteria_A_Arc:0.04543000,P09737_64091_Halobacteria_A_Arc:0.09052000):0.09236000):0.05854000):0.00618000,((A0A1I0MM22_355548_Halobacteria_A_Arc:0.02358000,P09224_64091_Halobacteria_A_Arc:0.15379000):0.02190000,C7NYW1_485914_Halobacteria_A_Arc:0.09170000):0.01426000):0.03555000):0.01278000,((A0A285N4Y7_558529_Halobacteria_A_Arc:0.05705000,((A0A4D6HF93_1457250_Halobacteria_A_Arc:0.05559000,M0CPB2_797114_Halobacteria_A_Arc:0.03720000):0.03814000,(Q03302_272569_Halobacteria_A_Arc:0.05373000,(A0A1I6K766_767519_Halobacteria_A_Arc:0.04058000,A0A1I6LVZ5_767519_Halobacteria_A_Arc:0.03952000):0.04501000):0.00734000):0.01867000):0.00962000,(A0A161XL90_1679489_Halobacteria_A_Arc:0.02308000,A0A1N6WYU5_553468_Halobacteria_A_Arc:0.03866000):0.02930000):0.01119000):0.00681000):0.02509000):0.08685000,I3RA82_523841_Halobacteria_A_Arc:0.20986000):0.04389000,A0A1D8S4L0_1873524_Halobacteria_A_Arc:0.12822000):0.32923000,((A0A1M5PRJ6_490188_Proteobacteria_B_Mar:0.63637000,((A0A2P8KJW4_1105204_Proteobacteria_B_Mar:0.27317000,A0A4D7B288_1940610_Proteobacteria_B_Mar:0.33814000):0.04441000,((A0A0N1BGP9_1523432_Proteobacteria_B_Mar:0.26154000,(A0A257J8A7_2015570_Proteobacteria_B_Mar:0.17311000,A0A2T5FZN3_1735121_Proteobacteria_B_Mar:0.09580000):0.11386000):0.25293000,(A0A1P8FEK9_1904640_Proteobacteria_B_Mar:0.27414000,A0A1V1PJD5_1605283_Proteobacteria_B_Mar:0.39855000):0.28095000):0.05476000):0.26777000):0.55778000,((K0AYP0_1128398_Firmicutes_B_Ter:1.21581000,((A0A1L4CZX9_1915309_Proteobacteria_B_Mar:0.65548000,(A0A0K1P8N3_1391653_Proteobacteria_B_Mar:0.26482000,(A0A1L6LK10_888845_Proteobacteria_B_Mar:0.19381000,A9GL55_448385_Proteobacteria_B_Mar:0.19201000):0.16174000):0.41070000):0.11358000,(((((A0A062V7A0_1392998_Methanomicrobia_A_Mar:0.16062000,A0A062V9F8_1392998_Methanomicrobia_A_Mar:0.15026000):0.32259000,((D8JZF0_552811_Chloroflexi_B_Ter:0.28526000,(A0A0W0GJW2_1217799_Chloroflexi_B_Ter:0.19625000,A0A1P8F872_1839801_Chloroflexi_B_Ter:0.16789000):0.06097000):0.15259000,Q3Z7W8_243164_Chloroflexi_B_Ter:0.34907000):0.05974000):0.04495000,A0A0C9Q165_1197129_Planctomycetes_B_Mar:0.19701000):0.19966000,(F8L7W5_331113_Chlamydiae_B_Mar:0.55584000,(A0A090CZB7_1437425_Chlamydiae_B_Mar:0.34044000,F8L0F2_765952_Chlamydiae_B_Mar:0.23286000):0.18266000):0.21185000):0.16456000,(A0A1I1XIF6_54_Proteobacteria_B_Mar:0.71380000,(A7HBL1_404589_Proteobacteria_B_Mar:0.23765000,Q09C50_378806_Proteobacteria_B_Mar:0.35766000):0.27888000):0.23212000):0.48558000):0.19437000):0.09331000,((B4U6J9_380749_Aquificae_B_Anc:0.19868000,C1DW98_204536_Aquificae_B_Anc:0.21415000):0.28720000,((A0A218ZVX8_1961136_Thermoplasmata_A_Arc:0.07552000,(T0MPA5_667137_Thermoplasmata_A_Arc:0.13233000,((Q9HM56_273075_Thermoplasmata_A_Arc:0.19969000,(Q6L1T7_263820_Thermoplasmata_A_Arc:0.00737000,(A0A0N8VL92_312540_Thermoplasmata_A_Arc:0.03794000,S0AS08_333146_Thermoplasmata_A_Arc:0.08352000):0.05354000):0.16164000):0.12311000,T0M4N9_667135_Thermoplasmata_A_Arc:0.10807000):0.01791000):0.06521000):0.07078000,T0MZU0_261391_Thermoplasmata_A_Arc:0.11100000):0.19361000):0.23829000):0.20666000):0.61427000):0.15436000):0.10502000):0.07417000,((A0A3P3RLZ3_671145_Halobacteria_A_Arc:0.29728000,(A0A365T5J1_29295_Halobacteria_A_Arc:0.03224000,A0A365T5J6_29295_Halobacteria_A_Arc:0.01076000):0.16229000):0.15481000,((A0A1N7AFB9_588898_Halobacteria_A_Arc:0.25616000,(M0AV65_29540_Halobacteria_A_Arc:0.18463000,((D2S0I8_543526_Halobacteria_A_Arc:0.10432000,L0JXL3_694430_Halobacteria_A_Arc:0.05889000):0.16603000,(L9XNL7_1227499_Halobacteria_A_Arc:0.07131000,M0LBE9_1227454_Halobacteria_A_Arc:0.23536000):0.03689000):0.06285000):0.09237000):0.19038000,(A0A1H6VX19_1073996_Halobacteria_A_Arc:0.20924000,(A0A1I2QXX4_553467_Halobacteria_A_Arc:0.13991000,(A0A0W1R5I9_1514971_Halobacteria_A_Arc:0.07440000,(A0A365TF12_29295_Halobacteria_A_Arc:0.08139000,(A0A166S5V7_1679489_Halobacteria_A_Arc:0.09655000,A0A1N6WZ27_553468_Halobacteria_A_Arc:0.05073000):0.02381000):0.02444000):0.09411000):0.05958000):0.13450000):0.03302000):0.10000000):0.11586000,((F2KQT8_693661_Archaeoglobi_A_Arc:0.27988000,((A0A498GYD6_1550565_Methanomicrobia_A_Mar:0.19484000,(A0A0X3BJM4_86622_Methanomicrobia_A_Mar:0.00595000,I7KYD2_1201294_Methanomicrobia_A_Arc:0.00472000):0.12732000):0.11874000,((A0B701_349307_Methanomicrobia_A_Arc:0.22823000,(F4BZF7_990316_Methanomicrobia_A_Arc:0.08549000,G7WL45_1110509_Methanomicrobia_A_Arc:0.10523000):0.10893000):0.06265000,((((F7XLC9_679901_Methanomicrobia_A_Arc:0.27975000,(A0A0E3WWB9_1434107_Methanomicrobia_A_Mar:0.08605000,((A0A0E3NRG6_1434102_Methanomicrobia_A_Mar:0.04762000,Q8TQG9_188937_Methanomicrobia_A_Arc:0.05900000):0.01410000,(A0A0E3NWV4_1434100_Methanomicrobia_A_Mar:0.11253000,(K4MC62_1094980_Methanomicrobia_A_Arc:0.09720000,L0KZ58_867904_Methanomicrobia_A_Arc:0.10532000):0.01339000):0.04468000):0.04076000):0.07570000):0.26511000,A0A0Q4BCN0_1713724_Thermoplasmata_A_Arc:0.51511000):0.04795000,(P18868_187420_Methanobacteria_A_Arc:0.18560000,(U6EAT7_1379702_Methanobacteria_A_Arc:0.16509000,(A0A1D2W9T7_1860100_Methanobacteria_A_Arc:0.28401000,(A0A1D3L2G7_118062_Methanobacteria_A_Arc:0.12556000,F0TCH4_877455_Methanobacteria_A_Arc:0.15179000):0.06186000):0.03319000):0.07269000):0.18002000):0.06158000,((A7I8G0_456442_Methanomicrobia_A_Arc:0.10545000,L0HJ20_593750_Methanomicrobia_A_Arc:0.14332000):0.07679000,(Q2FSC2_323259_Methanomicrobia_A_Arc:0.29163000,(A7I921_456442_Methanomicrobia_A_Arc:0.15259000,L0HEM7_593750_Methanomicrobia_A_Arc:0.10163000):0.05086000):0.06871000):0.08463000):0.03691000):0.08678000):0.15936000):0.07930000,(((O93724_178306_Thermoprotei_A_Arc:0.01513000,(A1RW25_384616_Thermoprotei_A_Arc:0.04827000,G7VBY8_1104324_Thermoprotei_A_Arc:0.03205000):0.02345000):0.11998000,(Q9Y8H8_272557_Thermoprotei_A_Arc:0.15699000,(D9Q0R7_666510_Thermoprotei_A_Arc:0.13888000,L0AA53_1056495_Thermoprotei_A_Arc:0.12237000):0.24176000):0.10676000):0.11695000,((A8MAM7_397948_Thermoprotei_A_Arc:0.07060000,E1QRN4_572478_Thermoprotei_A_Arc:0.10802000):0.22679000,(T0LM88_667138_Thermoplasmata_A_Arc:0.30734000,((P80857_273057_Thermoprotei_A_Arc:0.06264000,Q08713_330779_Thermoprotei_A_Arc:0.11148000):0.02138000,((((Q96Y84_273063_Thermoprotei_A_Arc:0.04157000,(A0A2U9IFV2_41673_Thermoprotei_A_Arc:0.06308000,(F4B3P9_933801_Thermoprotei_A_Arc:0.06673000,A0A031LPG5_1160895_Thermoprotei_A_Arc:0.08350000):0.05518000):0.03910000):0.02620000,W7KP27_1326980_Thermoprotei_A_Arc:0.04562000):0.02739000,(A4YHX7_399549_Thermoprotei_A_Arc:0.02669000,H2C9I7_671065_Thermoprotei_A_Arc:0.03837000):0.07752000):0.03402000,A0A348B0W0_1670455_Thermoprotei_A_Arc:0.13390000):0.00944000):0.02931000):0.09232000):0.45527000):0.09061000):0.25032000):0.01376000,(((O84296_272561_Chlamydiae_B_Mar:0.63666000,(A0A090CXU8_1437425_Chlamydiae_B_Mar:0.20627000,(D6YU13_716544_Chlamydiae_B_Mar:0.24625000,F8KWM1_765952_Chlamydiae_B_Mar:0.18822000):0.10925000):0.20676000):0.16455000,((((A0A095XIK9_1230730_Firmicutes_B_Ter:0.29880000,A0A2Z3GPK6_114_Planctomycetes_B_Mar:0.39669000):0.20788000,(((((((((((((A0A1I5ZIY7_1227077_Bacteroidetes_B_Mar:0.26654000,(A0A142L0Q1_1690483_Bacteroidetes_B_Mar:0.16882000,A0A257L3N9_2015582_Bacteroidetes_B_Mar:0.12298000):0.09399000):0.03095000,(A0A085L1P1_1453500_Bacteroidetes_B_Mar:0.17400000,A0A2N3IHE2_2016530_Bacteroidetes_B_Mar:0.20248000):0.06103000):0.04222000,((((B3ER22_452471_Bacteroidetes_B_Mar:0.35824000,U2EL32_1033802_Proteobacteria_B_Mar:0.48706000):0.03961000,A0A0S2I4J1_1307839_Bacteroidetes_B_Mar:0.23503000):0.06345000,A0A1G6HPU7_1640674_Bacteroidetes_B_Mar:0.28324000):0.06986000,A0A1I2HT21_1003_Bacteroidetes_B_Mar:0.16532000):0.01113000):0.02893000,(M7Y218_1239962_Bacteroidetes_B_Mar:0.05750000,U5C2N4_1123057_Bacteroidetes_B_Mar:0.13909000):0.12148000):0.05351000,((A1ZTZ0_313606_Bacteroidetes_B_Mar:0.22560000,(A0A1G9F980_1075417_Bacteroidetes_B_Mar:0.17420000,A0A1G9H772_1075417_Bacteroidetes_B_Mar:0.13711000):0.05390000):0.03076000,(((A0A0D3LFT2_1257021_Bacteroidetes_B_Mar:0.17960000,I4APS4_880071_Bacteroidetes_B_Mar:0.21459000):0.04748000,(((A0A098SDX5_1524460_Bacteroidetes_B_Mar:0.07334000,A0A2D0ND73_1122177_Bacteroidetes_B_Mar:0.12283000):0.13136000,((A0A3E1EU86_1737063_Bacteroidetes_B_Mar:0.09129000,((A6EQH5_50743_Bacteroidetes_B_Mar:0.19897000,Q26GK1_156586_Bacteroidetes_B_Mar:0.08607000):0.06908000,(C0BHJ2_487796_Bacteroidetes_B_Mar:0.25288000,(A0A328YCR8_1101402_Bacteroidetes_B_Mar:0.07821000,A3J2V1_391598_Bacteroidetes_B_Mar:0.12030000):0.02828000):0.04667000):0.05810000):0.07732000,H6L8Y0_984262_Bacteroidetes_B_Mar:0.13240000):0.02422000):0.06438000,(A0A1Y1CJJ9_1717717_Bacteroidetes_B_Mar:0.14987000,(A0A1I1YWR4_385682_Bacteroidetes_B_Mar:0.13534000,A0A0L8V2C5_1409788_Bacteroidetes_B_Mar:0.24029000):0.02243000):0.06534000):0.05980000):0.03162000,G8R729_926562_Bacteroidetes_B_Mar:0.23156000):0.05147000):0.06304000):0.03799000,(((((A0A0L8V3P2_1409788_Bacteroidetes_B_Mar:0.27573000,A0A1I1VUU8_385682_Bacteroidetes_B_Mar:0.25601000):0.14059000,(A0A0S7C2S3_1678841_Bacteroidetes_B_Mar:0.36727000,U5Q5M1_1400053_Bacteroidetes_B_Mar:0.35529000):0.12603000):0.12384000,(A0A0S7C7E3_1678841_Bacteroidetes_B_Mar:0.22595000,(A0A1G6RPM1_1640674_Bacteroidetes_B_Mar:0.51624000,(A0A0F5VHM9_265726_Proteobacteria_B_Mar:0.32026000,A0A380N0R1_13276_Proteobacteria_B_Mar:0.39291000):0.09675000):0.07941000):0.03959000):0.07632000,((D0J9E3_600809_Bacteroidetes_B_Mar:0.53863000,(A0A328YDD7_1101402_Bacteroidetes_B_Mar:0.52414000,A3J5A6_391598_Bacteroidetes_B_Mar:0.31706000):0.15106000):0.11767000,(A0A0S2HVA3_1307839_Bacteroidetes_B_Mar:0.54853000,A0A2D0NIH0_1122177_Bacteroidetes_B_Mar:0.38073000):0.05324000):0.05280000):0.04159000,(((A0A2K9NRY1_960_Proteobacteria_B_Mar:0.56949000,A3J209_391598_Bacteroidetes_B_Mar:0.30316000):0.13522000,((A0A1U9JNI9_1938604_Proteobacteria_B_Mar:0.31550000,Q7M864_273121_Proteobacteria_B_Mar:0.15445000):0.10036000,((((A0A2T5G1R0_1735121_Proteobacteria_B_Mar:0.17198000,A0A4Q5S3N2_1978525_Proteobacteria_B_Mar:0.18012000):0.02604000,(A0A192D580_1112_Proteobacteria_B_Mar:0.11109000,(A0A0B1Q2K7_370622_Proteobacteria_B_Mar:0.22922000,A0A3T0EBQ7_1434191_Proteobacteria_B_Mar:0.33635000):0.11565000):0.02515000):0.09735000,A0A2P2EAB2_1445552_Proteobacteria_B_Mar:0.34609000):0.09738000,Q08ZM8_378806_Proteobacteria_B_Mar:0.33494000):0.07114000):0.12278000):0.09456000,((A0A1R4IAU7_1434842_Bacteroidetes_B_Mar:0.72465000,(M7YBF0_1239962_Bacteroidetes_B_Mar:0.17305000,U5C1V6_1123057_Bacteroidetes_B_Mar:0.31048000):0.09893000):0.14527000,(H6L6A2_984262_Bacteroidetes_B_Mar:0.65367000,((A0A2N3IJD2_2016530_Bacteroidetes_B_Mar:0.34630000,A0A1I2GPG4_1003_Bacteroidetes_B_Mar:0.29458000):0.06097000,(A0A1H3Y937_408074_Bacteroidetes_B_Mar:0.26676000,I4AH06_880071_Bacteroidetes_B_Mar:0.44734000):0.09241000):0.09653000):0.02880000):0.04567000):0.02575000):0.04380000):0.02031000,(((A0A1H3XW14_408074_Bacteroidetes_B_Mar:0.15251000,(A0A4Q2ZMN6_2044944_Bacteroidetes_B_Mar:0.25034000,A0A1R4L182_1434842_Bacteroidetes_B_Mar:0.22260000):0.05069000):0.02612000,(A0A191ZFD3_1860122_Proteobacteria_B_Mar:0.29013000,A0A2D0NBJ4_1122177_Bacteroidetes_B_Mar:0.40007000):0.03105000):0.02771000,(A0A0X3T641_1685380_Proteobacteria_B_Mar:0.25838000,A0A2Z2P4N8_1192854_Proteobacteria_B_Mar:0.44672000):0.14482000):0.05150000):0.01115000,(I0AK38_945713_Ignavibacteriae_B_Mar:0.25359000,I6ZUA8_1191523_Ignavibacteriae_B_Mar:0.17275000):0.12125000):0.07232000,(D3FDM4_469383_Actinobacteria_B_Ter:0.36271000,(A0A345UPY2_1457365_Bacteroidetes_B_Mar:0.25297000,(Q9RUV2_243230_Deinococcus-Thermus_B_Ter:0.18555000,((A0A242PAB3_1970738_Proteobacteria_B_Mar:0.22236000,V9H8J0_641147_Proteobacteria_B_Mar:0.28229000):0.04648000,((A0A085G4I0_910964_Proteobacteria_B_Mar:0.04552000,(((G7LTJ2_598467_Proteobacteria_B_Mar:0.05779000,((A0A085HE47_1005999_Proteobacteria_B_Mar:0.05265000,A0A1I0EA93_1123402_Proteobacteria_B_Mar:0.10782000):0.01226000,A0A097R7C0_1453496_Proteobacteria_B_Mar:0.05577000):0.02505000):0.01571000,A0A2G0QEY2_351679_Proteobacteria_B_Mar:0.21694000):0.01205000,((A0A0J8VRJ1_435910_Proteobacteria_B_Mar:0.02480000,(A0A085JMH6_1005995_Proteobacteria_B_Mar:0.03035000,A0A2I0FW47_2025587_Proteobacteria_B_Mar:0.11184000):0.03677000):0.04953000,Q1LSZ4_374463_Proteobacteria_B_Mar:0.43656000):0.02679000):0.00520000):0.03731000,(A0A1V3TPU9_1924934_Proteobacteria_B_Mar:0.18517000,D5VB54_1236608_Proteobacteria_B_Mar:0.19557000):0.09056000):0.04813000):0.11282000):0.05912000):0.04401000):0.03759000):0.04323000,(((A0A1G6AIZ5_439219_Firmicutes_B_Ter:0.42187000,(A0A0R2HP56_1449336_Firmicutes_B_Ter:0.13989000,(S0L434_1140003_Firmicutes_B_Ter:0.21528000,L8XU36_1261130_Proteobacteria_B_Mar:0.24678000):0.03327000):0.03189000):0.05783000,(((A0A031IK26_1470592_Firmicutes_B_Ter:0.24796000,A0A4R6BX88_198484_Firmicutes_B_Ter:0.14816000):0.04499000,(A0A099W947_1552123_Firmicutes_B_Ter:0.30059000,A8FF82_315750_Firmicutes_B_Ter:0.12218000):0.06658000):0.01211000,(A0A0K0GDK0_1637974_Firmicutes_B_Ter:0.10106000,A0A2P8H4Q1_1176648_Firmicutes_B_Ter:0.13485000):0.09802000):0.04470000):0.03021000,(((A0A143PHC2_1855912_Acidobacteria_B_Mar:0.19449000,Q02A56_234267_Acidobacteria_B_Mar:0.34824000):0.10773000,(Q1ARZ8_266117_Actinobacteria_B_Ter:0.32573000,(A0A2A6RIS4_2024553_Chloroflexi_B_Ter:0.18349000,(A0A2H3KUE0_1506545_Chloroflexi_B_Ter:0.16959000,A9WJC9_324602_Chloroflexi_B_Ter:0.10455000):0.05309000):0.14365000):0.06862000):0.03865000,(((A9AWV1_316274_Chloroflexi_B_Ter:0.21598000,(A0A399EPG5_2026184_Deinococcus-Thermus_B_Ter:0.23760000,Q67QL1_292459_Firmicutes_B_Ter:0.09624000):0.09340000):0.02619000,((A0A192WU48_1660251_Acidobacteria_B_Mar:0.34503000,I0IFX1_1142394_Planctomycetes_B_Mar:0.43805000):0.06318000,((((E8QXM5_575540_Planctomycetes_B_Mer:0.33690000,(A0A2I8DCS9_1758194_Proteobacteria_B_Mar:0.15351000,(A0A1E4V0M9_1843690_Proteobacteria_B_Mar:0.25765000,(A0A4R1JLY6_412034_Proteobacteria_B_Mar:0.21868000,(A0A086D0E9_1492922_Proteobacteria_B_Mar:0.16279000,A0A2N7UR03_1684789_Proteobacteria_B_Mar:0.10964000):0.08106000):0.08252000):0.01317000):0.07095000):0.07739000,(A0A142X6W1_1632864_Planctomycetes_B_Mar:0.22510000,A9AXW8_316274_Chloroflexi_B_Ter:0.58470000):0.07715000):0.03644000,A0A212PXD1_877466_Chloroflexi_B_Ter:0.26336000):0.04873000,((I0I4H8_926550_Chloroflexi_B_Ter:0.27132000,(A0A062XM00_1312852_Acidobacteria_B_Mar:0.31040000,A0A0K2SFV8_1555112_Firmicutes_B_Ter:0.19197000):0.11110000):0.05875000,A0A0M9UDM4_872965_Chloroflexi_B_Ter:0.14386000):0.04272000):0.03908000):0.03398000):0.02630000,((E6SLN6_644966_Firmicutes_B_Ter:0.21543000,(A0A2T6BC70_1242148_Firmicutes_B_Ter:0.17987000,((A0A089IEX7_1536775_Firmicutes_B_Ter:0.18007000,A0A1M6L352_1830138_Firmicutes_B_Ter:0.13206000):0.06452000,A0A418MLK8_2048547_Firmicutes_B_Ter:0.20509000):0.04200000):0.03790000):0.03998000,(((A0A326UA20_644383_Chloroflexi_B_Ter:0.37219000,(((A0A326U2A6_644383_Chloroflexi_B_Ter:0.09274000,D6TP22_485913_Chloroflexi_B_Ter:0.11345000):0.03311000,(A0A328VKE5_1825093_Chloroflexi_B_Ter:0.15762000,D6TCG4_485913_Chloroflexi_B_Ter:0.19480000):0.05304000):0.01881000,(A0A326U110_644383_Chloroflexi_B_Ter:0.13912000,(A0A401ZTK7_2014871_Chloroflexi_B_Ter:0.12973000,D6U4H7_485913_Chloroflexi_B_Ter:0.15256000):0.07811000):0.05491000):0.10083000):0.02953000,((((A0A367QAR4_1844469_Cyanobacteria_B_Ter:0.16515000,(A0A0D8ZQ77_1618023_Cyanobacteria_B_Ter:0.09340000,K9XDJ4_1173026_Cyanobacteria_B_Ter:0.09109000):0.04717000):0.11609000,G8NWQ2_682795_Acidobacteria_B_Mar:0.31094000):0.04471000,(A0A3S0Y3G1_211165_Cyanobacteria_B_Ter:0.26974000,(A0A0B6WW40_454194_Acidobacteria_B_Mar:0.06197000,A0A0B6WWE2_454194_Acidobacteria_B_Mar:0.13160000):0.06830000):0.03366000):0.02502000,(((A0A0C1R8Q1_1479485_Cyanobacteria_B_Ter:0.08336000,(A0A367QYP4_1844469_Cyanobacteria_B_Ter:0.12318000,A0A3S0ZDA7_211165_Cyanobacteria_B_Ter:0.10796000):0.05912000):0.17040000,(A0A1E5QNR3_1781255_Cyanobacteria_B_Ter:0.22786000,((K9XZ62_111780_Cyanobacteria_B_Ter:0.12062000,(K9T7F8_118163_Cyanobacteria_B_Ter:0.17555000,B7KHG4_65393_Cyanobacteria_B_Ter:0.24080000):0.06072000):0.11692000,(A0A2W1JHI3_1764569_Cyanobacteria_B_Ter:0.24119000,U9VUJ7_1385935_Cyanobacteria_B_Ter:0.29569000):0.09162000):0.04651000):0.03688000):0.02677000,(K9ULY3_1173020_Cyanobacteria_B_Ter:0.28617000,(((A0A433MYY1_211165_Cyanobacteria_B_Ter:0.11647000,G6FT84_372787_Cyanobacteria_B_Ter:0.09648000):0.03611000,((A0A0C1QR13_1479485_Cyanobacteria_B_Ter:0.13189000,(A0A0C2QL44_1233231_Cyanobacteria_B_Ter:0.10156000,A0A1Y0RQE1_1940762_Cyanobacteria_B_Ter:0.03360000):0.02675000):0.02142000,(K9XKI9_1173026_Cyanobacteria_B_Ter:0.15295000,(A0A1Z4UUG6_1973480_Cyanobacteria_B_Ter:0.11109000,K9PU33_99598_Cyanobacteria_B_Ter:0.11653000):0.05076000):0.02353000):0.03860000):0.01616000,A0A0D8ZRD9_1618023_Cyanobacteria_B_Ter:0.13752000):0.04000000):0.06641000):0.07647000):0.06903000):0.06531000,((I4EEM3_1129897_Chloroflexi_B_Ter:0.23453000,(A0A2Z3H6M4_114_Planctomycetes_B_Mar:0.38857000,A5UY95_357808_Chloroflexi_B_Ter:0.17421000):0.11152000):0.03431000,((A0A2S0NH98_1868589_Proteobacteria_B_Mar:0.18494000,A0A4D7B538_1940610_Proteobacteria_B_Mar:0.22490000):0.34928000,(A0A327L5R7_29409_Proteobacteria_B_Mar:0.17073000,(A0A0H1RDD0_1225564_Proteobacteria_B_Mar:0.20872000,A0A437M3F2_1979269_Proteobacteria_B_Mar:0.25263000):0.04690000):0.29220000):0.08979000):0.05323000):0.07873000):0.01277000):0.02318000):0.02284000):0.03411000):0.02236000,((((D7CSR0_649638_Deinococcus-Thermus_B_Ter:0.23756000,I0IDH9_1142394_Planctomycetes_B_Mar:0.39556000):0.06258000,A0A1B6B8G0_1048380_Firmicutes_B_Ter:0.41604000):0.02626000,((((A0A1H3A4M8_1123352_Firmicutes_B_Ter:0.10965000,M1ZG58_1288971_Firmicutes_B_Ter:0.05400000):0.26998000,(A0A4R1R4E5_1469948_Firmicutes_B_Ter:0.37312000,E1QXA5_633147_Actinobacteria_B_Ter:0.32901000):0.23097000):0.08044000,Q2S1T9_309807_Bacteroidetes_B_Mar:0.39111000):0.08179000,A0A259TZ62_716817_Rhodothermaeota_B_Mar:0.34406000):0.03095000):0.03300000,U2QZH4_1321779_Fusobacteria_B_Anc:0.32061000):0.05726000):0.02613000,(((W3Y1G4_936589_Firmicutes_B_Ter:0.33973000,(V6IV34_1395513_Firmicutes_B_Ter:0.38987000,V6J6E9_1395513_Firmicutes_B_Ter:0.26395000):0.11358000):0.06194000,Q6YQD2_262768_Tenericutes_B_Ter:0.58878000):0.04843000,((E4T355_694427_Bacteroidetes_B_Mar:0.35839000,(A0A142L7E6_1690483_Bacteroidetes_B_Mar:0.25714000,A0A257L9C2_2015582_Bacteroidetes_B_Mar:0.47407000):0.25837000):0.16728000,M1Z874_1288971_Firmicutes_B_Ter:0.17592000):0.12846000):0.04244000):0.01528000,(((A0A1L8ZD32_64897_Spirochaetes_B_Mar:0.00805000,A0A1L8ZDB1_64897_Spirochaetes_B_Mar:0.00679000):0.89660000,A0A1I1DPZ5_34097_Spirochaetes_B_Mar:0.32381000):0.10761000,(A0A0C7NIR4_1006576_Thermotogae_B_Anc:0.30205000,L8DAQ3_1268239_Proteobacteria_B_Mar:0.46281000):0.11978000):0.07352000):0.03686000):0.03868000,((A0A062XUH1_1312852_Acidobacteria_B_Mar:0.34239000,A0A1J0ADU4_1188229_Cyanobacteria_B_Ter:0.40246000):0.09132000,((A0A1B4XH23_1620215_Proteobacteria_B_Mar:0.68579000,(D7CXL9_649638_Deinococcus-Thermus_B_Ter:0.57027000,D7CR17_649638_Deinococcus-Thermus_B_Ter:0.36432000):0.19072000):0.07687000,(R6GYH2_1262911_Firmicutes_B_Ter:1.15058000,((A0A2P2BST4_1507512_Firmicutes_B_Ter:0.60540000,R6EU58_1262994_Firmicutes_B_Ter:0.73914000):0.00305000,(A0A2P2BQH2_1507512_Firmicutes_B_Ter:0.71689000,(R7QTT1_1262942_Firmicutes_B_Ter:0.75146000,R5HFY3_1263001_Firmicutes_B_Ter:0.43673000):0.05225000):0.06989000):0.07914000):0.14056000):0.08954000):0.11818000):0.04217000,(A0A2U1E3U8_46507_Firmicutes_B_Ter:0.50731000,((A0A0S7C1K2_1678841_Bacteroidetes_B_Mar:0.33578000,(((A0A1Y1CGP3_1717717_Bacteroidetes_B_Mar:0.28088000,(A0A0S2I3Z7_1307839_Bacteroidetes_B_Mar:0.43329000,R5NVX0_1262909_Bacteroidetes_B_Mar:0.71600000):0.05574000):0.04454000,A0A0L8V2C9_1409788_Bacteroidetes_B_Mar:0.30464000):0.05560000,((A0A1G6HQ17_1640674_Bacteroidetes_B_Mar:0.32034000,A0A1I1UAP9_385682_Bacteroidetes_B_Mar:0.14201000):0.11363000,((((A0A1Y3VAM6_1965650_Bacteroidetes_B_Mar:0.31320000,A0A1Y3VCW3_1965650_Bacteroidetes_B_Mar:0.66018000):0.15108000,(A0A2X0WVT3_179995_Proteobacteria_B_Mar:0.78773000,A0A1Y4C9N7_1965623_Bacteroidetes_B_Mar:0.27289000):0.17469000):0.24895000,(((R5JCK6_1262737_Bacteroidetes_B_Mar:0.23910000,(A0A1I2LRE0_1855325_Bacteroidetes_B_Mar:0.23428000,S8FFI4_888054_Bacteroidetes_B_Mar:0.23896000):0.13896000):0.09629000,((A0A0A2F0U5_1515615_Bacteroidetes_B_Mar:0.34741000,D7JFA1_575590_Bacteroidetes_B_Mar:0.17709000):0.08585000,(A0A0F5IRT0_1203610_Bacteroidetes_B_Mar:0.07071000,(A0A0A2F1H1_1515615_Bacteroidetes_B_Mar:0.45899000,A0A2V3PRT9_1605892_Bacteroidetes_B_Mar:0.21893000):0.04761000):0.06476000):0.14386000):0.08245000,(W0ERB5_880074_Bacteroidetes_B_Mar:0.47218000,(R5P756_1262909_Bacteroidetes_B_Mar:0.42905000,R5PAP5_1262909_Bacteroidetes_B_Mar:0.22143000):0.09748000):0.06487000):0.08044000):0.09003000,E4T0E9_694427_Bacteroidetes_B_Mar:0.21684000):0.16932000):0.02916000):0.03329000):0.07613000,((((Q6MHD3_264462_Proteobacteria_B_Mar:0.61662000,Q6MKI3_264462_Proteobacteria_B_Mar:0.26277000):0.07926000,((C8Q0X7_553217_Proteobacteria_B_Mar:0.36256000,(A0A0F6YK52_927083_Proteobacteria_B_Mar:0.18803000,(A0A1L6KZF6_888845_Proteobacteria_B_Mar:0.04005000,A9EWL0_448385_Proteobacteria_B_Mar:0.09542000):0.11919000):0.18365000):0.10310000,((((((A0A1Y5U185_745714_Proteobacteria_B_Mar:0.36614000,(((A0A192D2N1_1112_Proteobacteria_B_Mar:0.56705000,(A0A2T5FTW8_1735121_Proteobacteria_B_Mar:0.33239000,A0A4Q5RR52_1978525_Proteobacteria_B_Mar:0.22625000):0.23867000):0.11716000,((A0A257J713_2015570_Proteobacteria_B_Mar:0.57432000,A0A4Q3S5H0_1978230_Proteobacteria_B_Mar:0.35833000):0.24279000,A0A193LCR6_1548547_Proteobacteria_B_Mar:0.55904000):0.13087000):0.08263000,X5MEY9_1458461_Proteobacteria_B_Mar:0.34162000):0.08700000):0.05782000,((A0A0S2KFC6_1249552_Proteobacteria_B_Mar:0.03888000,A0A1E8CFB9_1524254_Proteobacteria_B_Mar:0.07386000):0.33190000,E0TI36_314260_Proteobacteria_B_Mar:0.46645000):0.14034000):0.04500000,((((A0A0M2R958_1549748_Proteobacteria_B_Mar:0.13472000,(A0A4R2PEU5_1188247_Proteobacteria_B_Mar:0.37591000,(((((G2KRC6_856793_Proteobacteria_B_Mar:0.38050000,(H0TES2_551947_Proteobacteria_B_Mar:0.14005000,(A0A4R2GX74_659006_Proteobacteria_B_Mar:0.16407000,((A0A2S0N786_1868589_Proteobacteria_B_Mar:0.09389000,A0A4D7B883_1940610_Proteobacteria_B_Mar:0.11796000):0.09572000,(A0A0H1RD07_1225564_Proteobacteria_B_Mar:0.08428000,A0A327KT03_29409_Proteobacteria_B_Mar:0.14307000):0.04334000):0.04478000):0.07403000):0.09325000):0.01826000,(A0A256FXP0_571255_Proteobacteria_B_Mar:0.12376000,(((A0A231UUB0_1876515_Proteobacteria_B_Mar:0.38151000,A0A0B1Q8Z2_370622_Proteobacteria_B_Mar:0.06901000):0.08207000,A0A090FD11_1505946_Proteobacteria_B_Mar:0.07459000):0.09088000,W3TXZ6_1402976_Proteobacteria_B_Mar:0.26155000):0.03495000):0.05741000):0.04373000,A0A1E3VXW9_1774968_Proteobacteria_B_Mar:0.27855000):0.06004000,(A0A285MB93_1798205_Proteobacteria_B_Mar:0.15416000,F2IV67_991905_Proteobacteria_B_Mar:0.09660000):0.04167000):0.03867000,A0A1I4SFU3_1166257_Proteobacteria_B_Mar:0.17298000):0.11032000):0.08007000):0.04232000,((S9QPA8_1123237_Proteobacteria_B_Mar:0.14465000,(D5BTR8_488538_Proteobacteria_B_Mar:0.15199000,(G6A1M0_909943_Proteobacteria_B_Mar:0.12620000,Q0F8L9_367336_Proteobacteria_B_Mar:0.12761000):0.04199000):0.05205000):0.35362000,A8U258_331869_Proteobacteria_B_Mar:0.18578000):0.03590000):0.06510000,(D0RR50_684719_Proteobacteria_B_Mar:0.28484000,(A0A437MD01_1979269_Proteobacteria_B_Mar:0.43681000,(A0A4R7K0K2_332522_Proteobacteria_B_Mar:0.17168000,(A0A4D7B4V5_1940610_Proteobacteria_B_Mar:0.25297000,(A0A285M297_1798205_Proteobacteria_B_Mar:0.06865000,S9RPC2_1123237_Proteobacteria_B_Mar:0.12752000):0.16019000):0.09172000):0.13549000):0.21365000):0.06523000):0.06888000,((A0A061Q889_1492281_Proteobacteria_B_Mar:0.39140000,(A0A2S6N6L1_333368_Proteobacteria_B_Mar:0.22869000,A0A366ENT2_1473586_Proteobacteria_B_Mar:0.20944000):0.14487000):0.08003000,R5L6H9_1262760_Spirochaetes_B_Mar:0.61390000):0.03675000):0.19271000):0.04056000,(Q9ZD15_272947_Proteobacteria_B_Mar:0.70501000,(A0A077FIP1_1528098_Proteobacteria_B_Mar:0.18559000,A0A0C1QWB0_86105_Proteobacteria_B_Mar:0.35363000):0.09764000):0.12807000):0.02014000,(((A0A1Y5TAA6_745714_Proteobacteria_B_Mar:0.14354000,A0A3M0CGB4_911205_Proteobacteria_B_Mar:0.24201000):0.07682000,(A0A0M4CZS5_1603606_Proteobacteria_B_Mar:0.28467000,(C7LSI9_525897_Proteobacteria_B_Mar:0.29657000,A0A091FCF4_1499107_Proteobacteria_B_Mar:0.21042000):0.09361000):0.09080000):0.05844000,(((A0A3N1MAT8_94_Proteobacteria_B_Mar:0.17965000,A0A2N3PTI0_382514_Proteobacteria_B_Mar:0.24270000):0.08134000,((A0A4P8L1J4_980445_Proteobacteria_B_Mar:0.33672000,Q3YRT4_269484_Proteobacteria_B_Mar:0.58746000):0.05650000,((B3EJL9_331678_Chlorobi_B_Mar:0.37260000,(A0A4Q5XXF3_1913988_Proteobacteria_B_Mar:0.29705000,U5QI99_1183438_Cyanobacteria_B_Ter:0.27792000):0.12458000):0.06055000,((A0A1P8FPF1_1904640_Proteobacteria_B_Mar:0.45222000,(A0A1R4H9Z6_360316_Proteobacteria_B_Mar:0.16015000,A0A1R4HH45_360316_Proteobacteria_B_Mar:0.19550000):0.37651000):0.07411000,((A0A1G6E3R2_617002_Proteobacteria_B_Mar:0.40117000,((((A0A1G6EXC4_617002_Proteobacteria_B_Mar:0.13868000,A0A1K1LIX2_1855339_Proteobacteria_B_Mar:0.17095000):0.04898000,((C7LNS7_525897_Proteobacteria_B_Mar:0.24434000,C7LRC1_525897_Proteobacteria_B_Mar:0.20398000):0.08112000,A0A2S6NHJ6_333368_Proteobacteria_B_Mar:0.37322000):0.00136000):0.00628000,(Q3SGN8_292415_Proteobacteria_B_Mar:0.55153000,A0A4R8ITH7_381308_Proteobacteria_B_Mar:0.28557000):0.06715000):0.02038000,A0A250KYU0_1432792_Proteobacteria_B_Mar:0.18207000):0.09211000):0.05266000,(W6M9I9_1400863_Proteobacteria_B_Mar:0.22321000,(((A0A0N0K024_1523432_Proteobacteria_B_Mar:0.26277000,A0A257EMF7_2015572_Proteobacteria_B_Mar:0.27393000):0.15045000,(A0A2N3PQX2_382514_Proteobacteria_B_Mar:0.34509000,A0A1T4W9E9_1121442_Proteobacteria_B_Mar:0.77585000):0.15945000):0.07484000,A0A1V1PIR0_1605283_Proteobacteria_B_Mar:0.77897000):0.10720000):0.07440000):0.06376000):0.05751000):0.06812000):0.09292000):0.04930000,(((A0A1C0VXQ7_1880991_Cyanobacteria_B_Ter:0.15512000,(A0A073CMZ0_388467_Cyanobacteria_B_Ter:0.08710000,A0YJL0_313612_Cyanobacteria_B_Ter:0.20789000):0.05768000):0.07625000,(K9VXC7_1173022_Cyanobacteria_B_Ter:0.10564000,((((A0A367QB34_1844469_Cyanobacteria_B_Ter:0.04593000,(A0A433N6A6_211165_Cyanobacteria_B_Ter:0.09285000,G6G070_372787_Cyanobacteria_B_Ter:0.03670000):0.04551000):0.00922000,(K9PE47_99598_Cyanobacteria_B_Ter:0.07945000,A0A0C1MX08_1479485_Cyanobacteria_B_Ter:0.05450000):0.02041000):0.03064000,A0A1Z4UU62_1973480_Cyanobacteria_B_Ter:0.07900000):0.19284000,K9UQB8_1173020_Cyanobacteria_B_Ter:0.22355000):0.02786000):0.07986000):0.03616000,((A0A0C2QFL4_1233231_Cyanobacteria_B_Ter:0.07768000,A0A1Y0RVB5_1940762_Cyanobacteria_B_Ter:0.10122000):0.16110000,((A0A1E5QE38_1781255_Cyanobacteria_B_Ter:0.11607000,(A0A1J0A9M2_1188229_Cyanobacteria_B_Ter:0.29739000,K9SBX7_1173025_Cyanobacteria_B_Ter:0.24411000):0.07291000):0.02862000,(B7KAA9_65393_Cyanobacteria_B_Ter:0.09511000,(((P77968_1111708_Cyanobacteria_B_Ter:0.06690000,(A0A0M2Q238_317619_Cyanobacteria_B_Ter:0.11822000,B0JGF5_449447_Cyanobacteria_B_Ter:0.07708000):0.03515000):0.08406000,(K9Z4N7_755178_Cyanobacteria_B_Ter:0.16599000,(K9XY88_111780_Cyanobacteria_B_Ter:0.06930000,L8LWB9_102125_Cyanobacteria_B_Ter:0.06224000):0.05973000):0.03526000):0.01818000,K9T440_118163_Cyanobacteria_B_Ter:0.08260000):0.02865000):0.07761000):0.06722000):0.03145000):0.05469000):0.01905000):0.02760000):0.10326000,(((A0A0H4WNU9_1297742_Proteobacteria_B_Mar:0.06828000,(Q09CK0_378806_Proteobacteria_B_Mar:0.17618000,A0A0K1PVI4_1391654_Proteobacteria_B_Mar:0.07015000):0.03161000):0.03970000,A0A0K1PWM2_1391654_Proteobacteria_B_Mar:0.16165000):0.02354000,A0A1I2A523_54_Proteobacteria_B_Mar:0.11891000):0.17903000):0.04369000):0.04139000):0.06936000,((((A0A4Q6B980_1977087_Proteobacteria_B_Mar:0.23984000,(A0A1Y2K534_1434232_Proteobacteria_B_Mar:0.18170000,(A0A0K0XVJ8_1579979_Proteobacteria_B_Mar:0.15360000,A0A1Y6CDA5_1513793_Proteobacteria_B_Mar:0.20215000):0.04103000):0.09023000):0.04363000,(A0A1V3PIV2_1945854_Proteobacteria_B_Mar:0.21495000,(A0A0D0S5X1_1199154_Proteobacteria_B_Mar:0.17054000,A0A2P1PVM9_2021234_Proteobacteria_B_Mar:0.14876000):0.05144000):0.07080000):0.00313000,(A0A1M5RQ54_490188_Proteobacteria_B_Mar:0.27935000,A0A0W0TNM8_453_Proteobacteria_B_Mar:0.19936000):0.03815000):0.02807000,(((A0A2K8LAI0_1921087_Proteobacteria_B_Mar:0.21553000,((((A0A4V2PNM7_412034_Proteobacteria_B_Mar:0.16162000,(((((A0A097R2J1_1453496_Proteobacteria_B_Mar:0.04176000,(A0A2G0Q1U7_351679_Proteobacteria_B_Mar:0.15368000,A0A085HJD6_1005999_Proteobacteria_B_Mar:0.10169000):0.03130000):0.03964000,E1VHB8_83406_Proteobacteria_B_Mar:0.14054000):0.02331000,K2JEV8_745411_Proteobacteria_B_Mar:0.09410000):0.05052000,((A8H3Z8_398579_Proteobacteria_B_Mar:0.13217000,(A0A0F5VGV2_265726_Proteobacteria_B_Mar:0.11796000,E1SRJ0_550540_Proteobacteria_B_Mar:0.06307000):0.04361000):0.04080000,(A0A2S1JPT9_359370_Proteobacteria_B_Mar:0.11861000,(A0A1Y0FVA0_1987723_Proteobacteria_B_Mar:0.06095000,A0A4Q5V9J9_1913989_Proteobacteria_B_Mar:0.09687000):0.07016000):0.04177000):0.00945000):0.03332000,(A0A094J7J3_1517416_Proteobacteria_B_Mar:0.08038000,(A0A1E2V9J2_197479_Proteobacteria_B_Mar:0.16822000,A0A2A5LZ91_2039467_Proteobacteria_B_Mar:0.12349000):0.05049000):0.04482000):0.03705000):0.01632000,(A0A0B4XLG0_391936_Proteobacteria_B_Mar:0.17308000,(((A0A1H4F8V2_152573_Proteobacteria_B_Mar:0.10530000,L8D9X1_1268239_Proteobacteria_B_Mar:0.15147000):0.04493000,(A0A0S2JHC6_58049_Proteobacteria_B_Mar:0.15792000,(A0A2N0WW65_2058089_Proteobacteria_B_Mar:0.15651000,A0A090IF69_80854_Proteobacteria_B_Mar:0.13824000):0.03384000):0.03184000):0.02846000,(A4BGS7_314283_Proteobacteria_B_Mar:0.11472000,(A0A1H6CZE7_568106_Proteobacteria_B_Mar:0.11506000,((A0A162GML5_1822219_Proteobacteria_B_Mar:0.11055000,A0A081NMU1_1137799_Proteobacteria_B_Mar:0.16730000):0.03617000,I2JH08_1168065_Proteobacteria_B_Mar:0.21688000):0.02996000):0.00982000):0.07225000):0.04478000):0.05371000):0.02559000,(A0A395JH00_644221_Proteobacteria_B_Mar:0.13204000,C7RD31_523791_Proteobacteria_B_Mar:0.04177000):0.16641000):0.05246000,(((A0A1R3VN56_233100_Proteobacteria_B_Mar:0.16645000,B5JWC0_391615_Proteobacteria_B_Mar:0.21659000):0.08779000,(W0TLC1_1076588_Proteobacteria_B_Mar:0.18318000,(A0A1Z4VNQ5_585455_Proteobacteria_B_Mar:0.13300000,(A0A0B0HBS3_2340_Proteobacteria_B_Mar:0.00363000,A0A1T2L771_1918949_Proteobacteria_B_Mar:0.04110000):0.23524000):0.06767000):0.05501000):0.03953000,((G2E543_765913_Proteobacteria_B_Mar:0.15420000,((A0A1E2ZEY7_1655433_Proteobacteria_B_Mar:0.02861000,(A0A1E2V0Q9_1818881_Proteobacteria_B_Mar:0.01710000,G2FFW9_1049564_Proteobacteria_B_Mar:0.07869000):0.01853000):0.14199000,(A0A1T2KSF0_1918948_Proteobacteria_B_Mar:0.11601000,A0A0F7K213_1543721_Proteobacteria_B_Mar:0.14236000):0.03986000):0.01924000):0.03066000,(D0LUJ8_502025_Proteobacteria_B_Mar:0.39064000,E1X0Y3_862908_Proteobacteria_B_Mar:0.27788000):0.12185000):0.03570000):0.02087000):0.03477000):0.03662000,(A0A2K9NQW2_960_Proteobacteria_B_Mar:0.34750000,((K1JSW8_742823_Proteobacteria_B_Mar:0.30113000,R6A170_1262986_Proteobacteria_B_Mar:0.32588000):0.04319000,((((A0A2I8DQ40_1758194_Proteobacteria_B_Mar:0.05844000,(A0A2T0XJH1_323426_Proteobacteria_B_Mar:0.08305000,(M1L4D0_1208918_Proteobacteria_B_Mar:0.24805000,M1M096_1208922_Proteobacteria_B_Mar:0.34816000):0.17621000):0.01782000):0.04117000,((A0A2G3K1V1_1559339_Proteobacteria_B_Mar:0.21141000,(A0A1U9JL93_1938604_Proteobacteria_B_Mar:0.06927000,(A0A2U2AEQ9_472582_Proteobacteria_B_Mar:0.24944000,(A0A142LJU4_1690485_Proteobacteria_B_Mar:0.28205000,A0A149W187_1789004_Proteobacteria_B_Mar:0.50661000):0.05857000):0.06376000):0.04379000):0.04347000,A0A0N1LB93_1523428_Proteobacteria_B_Mar:0.13236000):0.03934000):0.03244000,L9PNM3_1198452_Proteobacteria_B_Mar:0.12852000):0.03731000,(((V5ADP9_1408164_Proteobacteria_B_Mar:0.12707000,(A0A0N0JCV7_1523424_Proteobacteria_B_Mar:0.03989000,A0A0U3DNG9_1768242_Proteobacteria_B_Mar:0.09739000):0.02693000):0.04171000,A0A2P8KDF2_1105204_Proteobacteria_B_Mar:0.10308000):0.04399000,(((H0PZQ1_748247_Proteobacteria_B_Mar:0.07723000,A0A0K6IWK3_876478_Proteobacteria_B_Mar:0.15886000):0.04743000,(((A0A1R1I0D0_418702_Proteobacteria_B_Mar:0.05039000,(A0A401JC34_1559896_Proteobacteria_B_Mar:0.06285000,Q3SK26_292415_Proteobacteria_B_Mar:0.03313000):0.04122000):0.01763000,((A0A011QCI9_1454004_Proteobacteria_B_Mar:0.04112000,((A0A1A8XY42_1860102_Proteobacteria_B_Mar:0.05316000,C7RIZ7_522306_Proteobacteria_B_Mar:0.01982000):0.04650000,(A0A080M9P3_1453999_Proteobacteria_B_Mar:0.03778000,(A0A011NKF6_1454000_Proteobacteria_B_Mar:0.02402000,A0A011MHB8_1454001_Proteobacteria_B_Mar:0.04131000):0.06565000):0.01233000):0.01612000):0.05714000,A0A1G8BH66_83767_Proteobacteria_B_Mar:0.02103000):0.04777000):0.02642000,A0A497X9T4_1381557_Proteobacteria_B_Mar:0.01345000):0.03908000):0.04910000,A0A0Q6BJ75_1736373_Proteobacteria_B_Mar:0.06783000):0.04687000):0.05445000):0.03854000):0.04005000):0.05767000):0.02699000,((((A0A251X438_1570016_Proteobacteria_B_Mar:0.17754000,((A0A1B1YPZ1_1810504_Proteobacteria_B_Mar:0.18173000,((A0A1R4HJV0_360316_Proteobacteria_B_Mar:0.05139000,A0A1Z5HC40_113268_Proteobacteria_B_Mar:0.15590000):0.04583000,(A0A1J4QJC3_1414654_Proteobacteria_B_Mar:0.13803000,(A0A139SPB8_1680762_Proteobacteria_B_Mar:0.11036000,A0A1E4UW72_1843690_Proteobacteria_B_Mar:0.07590000):0.04141000):0.05333000):0.02193000):0.02073000,A0A250KTZ9_1432792_Proteobacteria_B_Mar:0.13227000):0.01367000):0.02764000,A0A0A6PC03_1003181_Proteobacteria_B_Mar:0.15197000):0.00956000,W6M2C8_1400863_Proteobacteria_B_Mar:0.16150000):0.03245000,A0A401JA31_1559896_Proteobacteria_B_Mar:0.16610000):0.00832000):0.02944000):0.08114000):0.04951000,((A0A2Z2L2V6_549298_Proteobacteria_B_Mar:0.33466000,((A0A1V0RDW6_28898_Proteobacteria_B_Mar:0.37742000,Q7M8L0_273121_Proteobacteria_B_Mar:0.38810000):0.23219000,A0A395JG70_644221_Proteobacteria_B_Mar:0.84665000):0.10050000):0.03712000,(((G2E6T6_765913_Proteobacteria_B_Mar:0.42597000,I2K993_1165841_Proteobacteria_B_Mar:0.50885000):0.14043000,(F8L8N3_331113_Chlamydiae_B_Mar:0.42554000,(A1ZNK9_313606_Bacteroidetes_B_Mar:0.11618000,(A0A1R4KBB1_1434842_Bacteroidetes_B_Mar:0.07629000,A0A1R4KQ40_1434842_Bacteroidetes_B_Mar:0.06691000):0.13660000):0.25438000):0.12716000):0.09401000,(A0A2D3WM99_2015906_Proteobacteria_B_Mar:0.41960000,A8PPX3_59196_Proteobacteria_B_Mar:0.35894000):0.13333000):0.05969000):0.03498000):0.10409000):0.22457000):0.19559000):0.29630000):0.21188000,((A0A0M5KZK2_1528099_Actinobacteria_B_Ter:0.38991000,(Q83GI4_203267_Actinobacteria_B_Ter:0.54546000,(((A0A1I1CI47_490629_Actinobacteria_B_Ter:0.22165000,A0A2T0GV44_33906_Actinobacteria_B_Ter:0.10424000):0.15122000,(K6W4W1_1184607_Actinobacteria_B_Ter:0.20865000,((E5XKX2_679197_Actinobacteria_B_Ter:0.30161000,(A0A3D9SUE6_111806_Actinobacteria_B_Ter:0.06119000,(A0A2T0ZW47_1629062_Actinobacteria_B_Ter:0.18467000,D3PZ20_446470_Actinobacteria_B_Ter:0.08604000):0.09689000):0.08660000):0.08377000,(A0A166QH71_683316_Actinobacteria_B_Ter:0.13720000,(A0A1V2RP88_1857892_Actinobacteria_B_Ter:0.06673000,(A0A0N1G8F9_1592327_Actinobacteria_B_Ter:0.06753000,A0A0N1H7L0_1592329_Actinobacteria_B_Ter:0.06897000):0.05403000):0.23298000):0.08824000):0.06526000):0.05344000):0.13742000,((((A0A166PIY3_37915_Actinobacteria_B_Ter:0.08422000,D5UQ12_521096_Actinobacteria_B_Ter:0.06346000):0.08191000,(A0A0Q5R2U1_1736349_Actinobacteria_B_Ter:0.05667000,K6WAV5_1108045_Actinobacteria_B_Ter:0.08814000):0.10111000):0.10556000,(A0A378YCD3_1823_Actinobacteria_B_Ter:0.01548000,(A0A386UD88_1389713_Actinobacteria_B_Ter:0.11464000,E5XUX1_679197_Actinobacteria_B_Ter:0.11823000):0.03262000):0.13097000):0.06047000,(((((((A0A1I3W540_1855324_Actinobacteria_B_Ter:0.08085000,((A0A168EFF5_1300344_Actinobacteria_B_Ter:0.04490000,A0A3N5AAM9_154117_Actinobacteria_B_Ter:0.14509000):0.10311000,(A0A2A9CZS9_556530_Actinobacteria_B_Ter:0.20974000,(A0A1G6RCD0_1814289_Actinobacteria_B_Ter:0.07022000,C7R4P3_471856_Actinobacteria_B_Ter:0.06625000):0.09182000):0.08797000):0.08786000):0.07807000,C0W267_525245_Actinobacteria_B_Ter:0.17227000):0.06899000,A0A1H6UCM7_1043493_Actinobacteria_B_Ter:0.18298000):0.02480000,((A0A329QP63_1981511_Actinobacteria_B_Ter:0.17954000,A0A2T0PUC9_1144618_Actinobacteria_B_Ter:0.13006000):0.07494000,A0A1I5SFS4_1523247_Actinobacteria_B_Ter:0.22644000):0.04515000):0.02638000,A0A1H0IML9_1090615_Actinobacteria_B_Ter:0.23139000):0.03533000,A0A223S566_1235441_Actinobacteria_B_Ter:0.15682000):0.04082000,(((A0A1H8D259_1424661_Actinobacteria_B_Ter:0.10206000,A4AJQ1_312284_Actinobacteria_B_Ter:0.11549000):0.17215000,(A0A0H0ZNJ8_1652545_Actinobacteria_B_Ter:0.11453000,A0A239QSP2_1945888_Actinobacteria_B_Ter:0.21773000):0.07517000):0.02691000,(A0A4R7JC83_993414_Actinobacteria_B_Ter:0.32137000,D4YMB9_585530_Actinobacteria_B_Ter:0.12054000):0.08459000):0.04164000):0.01792000):0.08335000):0.06901000):0.07419000):0.08440000,A0A2A9DLZ4_1724_Actinobacteria_B_Ter:0.25672000):0.15826000):0.09364000);
