# Supplementary material for: Characterization of ancestral Fe/Mn superoxide dismutases indicates their cambialistic origin
Source: Protein Sci. 2022 Sep 21;31(10):e4423. doi: 10.1002/pro.4423 (PMC9490801; doi:10.1002/pro.4423)
Supplement: Supplementary file 2 — Dataset S2 [file PRO-31-e4423-s011.zip › PRO_4423_Dataset2_PhylogeneticTree.docx]

(((O84296_272561_Chlamydiae_B_Mar:0.61337000,(A0A090CXU8_1437425_Chlamydiae_B_Mar:0.19140000,(D6YU13_716544_Chlamydiae_B_Mar:0.24285000,F8KWM1_765952_Chlamydiae_B_Mar:0.18884000):0.12399000):0.24506000):0.20120000,(((((((((A0A1G6AIZ5_439219_Firmicutes_B_Ter:0.41162000,(A0A0R2HP56_1449336_Firmicutes_B_Ter:0.13948000,(S0L434_1140003_Firmicutes_B_Ter:0.21508000,L8XU36_1261130_Proteobacteria_B_Mar:0.25093000):0.03520000):0.03450000):0.05919000,(((A0A031IK26_1470592_Firmicutes_B_Ter:0.24460000,A0A4R6BX88_198484_Firmicutes_B_Ter:0.14852000):0.04358000,(A0A099W947_1552123_Firmicutes_B_Ter:0.30209000,A8FF82_315750_Firmicutes_B_Ter:0.12156000):0.06793000):0.01131000,(A0A0K0GDK0_1637974_Firmicutes_B_Ter:0.09744000,A0A2P8H4Q1_1176648_Firmicutes_B_Ter:0.13862000):0.09973000):0.04432000):0.03057000,(((A0A143PHC2_1855912_Acidobacteria_B_Mar:0.19175000,Q02A56_234267_Acidobacteria_B_Mar:0.35087000):0.10273000,(Q1ARZ8_266117_Actinobacteria_B_Ter:0.32840000,(A0A2A6RIS4_2024553_Chloroflexi_B_Ter:0.18737000,(A0A2H3KUE0_1506545_Chloroflexi_B_Ter:0.16569000,A9WJC9_324602_Chloroflexi_B_Ter:0.10767000):0.04888000):0.14108000):0.07112000):0.03755000,(((A9AWV1_316274_Chloroflexi_B_Ter:0.21441000,(A0A399EPG5_2026184_Deinococcus-Thermus_B_Ter:0.23539000,Q67QL1_292459_Firmicutes_B_Ter:0.09832000):0.09656000):0.02828000,((A0A192WU48_1660251_Acidobacteria_B_Mar:0.34810000,I0IFX1_1142394_Planctomycetes_B_Mar:0.44317000):0.06190000,((((E8QXM5_575540_Planctomycetes_B_Mer:0.33581000,(A0A2I8DCS9_1758194_Proteobacteria_B_Mar:0.15447000,(A0A1E4V0M9_1843690_Proteobacteria_B_Mar:0.25832000,(A0A4R1JLY6_412034_Proteobacteria_B_Mar:0.22067000,(A0A086D0E9_1492922_Proteobacteria_B_Mar:0.16323000,A0A2N7UR03_1684789_Proteobacteria_B_Mar:0.11014000):0.07928000):0.08313000):0.01321000):0.06932000):0.07683000,(A0A142X6W1_1632864_Planctomycetes_B_Mar:0.22582000,A9AXW8_316274_Chloroflexi_B_Ter:0.58265000):0.07747000):0.03453000,A0A212PXD1_877466_Chloroflexi_B_Ter:0.26352000):0.04978000,((I0I4H8_926550_Chloroflexi_B_Ter:0.26961000,(A0A062XM00_1312852_Acidobacteria_B_Mar:0.31224000,A0A0K2SFV8_1555112_Firmicutes_B_Ter:0.19181000):0.11199000):0.05380000,A0A0M9UDM4_872965_Chloroflexi_B_Ter:0.14973000):0.04183000):0.03468000):0.03452000):0.02594000,((((A0A326UA20_644383_Chloroflexi_B_Ter:0.36297000,(((A0A326U2A6_644383_Chloroflexi_B_Ter:0.09609000,D6TP22_485913_Chloroflexi_B_Ter:0.10944000):0.02484000,(A0A326U110_644383_Chloroflexi_B_Ter:0.14577000,(A0A401ZTK7_2014871_Chloroflexi_B_Ter:0.12230000,D6U4H7_485913_Chloroflexi_B_Ter:0.15860000):0.07232000):0.06562000):0.02832000,(A0A328VKE5_1825093_Chloroflexi_B_Ter:0.15738000,D6TCG4_485913_Chloroflexi_B_Ter:0.19439000):0.03993000):0.10257000):0.03506000,(((A0A0C1R8Q1_1479485_Cyanobacteria_B_Ter:0.07726000,(A0A367QYP4_1844469_Cyanobacteria_B_Ter:0.12274000,A0A3S0ZDA7_211165_Cyanobacteria_B_Ter:0.10953000):0.06378000):0.16785000,(A0A1E5QNR3_1781255_Cyanobacteria_B_Ter:0.21875000,((K9XZ62_111780_Cyanobacteria_B_Ter:0.12573000,(K9T7F8_118163_Cyanobacteria_B_Ter:0.17727000,B7KHG4_65393_Cyanobacteria_B_Ter:0.23851000):0.05750000):0.12902000,(A0A2W1JHI3_1764569_Cyanobacteria_B_Ter:0.22774000,U9VUJ7_1385935_Cyanobacteria_B_Ter:0.30606000):0.08613000):0.04631000):0.04733000):0.05686000,((((A0A367QAR4_1844469_Cyanobacteria_B_Ter:0.17381000,(A0A0D8ZQ77_1618023_Cyanobacteria_B_Ter:0.09391000,K9XDJ4_1173026_Cyanobacteria_B_Ter:0.09104000):0.03881000):0.11252000,G8NWQ2_682795_Acidobacteria_B_Mar:0.31699000):0.04413000,(A0A3S0Y3G1_211165_Cyanobacteria_B_Ter:0.26859000,(A0A0B6WW40_454194_Acidobacteria_B_Mar:0.06513000,A0A0B6WWE2_454194_Acidobacteria_B_Mar:0.12894000):0.06382000):0.04046000):0.01969000,(K9ULY3_1173020_Cyanobacteria_B_Ter:0.27204000,(((A0A433MYY1_211165_Cyanobacteria_B_Ter:0.11603000,G6FT84_372787_Cyanobacteria_B_Ter:0.09708000):0.02997000,((A0A0C1QR13_1479485_Cyanobacteria_B_Ter:0.13236000,(A0A0C2QL44_1233231_Cyanobacteria_B_Ter:0.10080000,A0A1Y0RQE1_1940762_Cyanobacteria_B_Ter:0.03436000):0.02376000):0.02043000,(K9XKI9_1173026_Cyanobacteria_B_Ter:0.15567000,(A0A1Z4UUG6_1973480_Cyanobacteria_B_Ter:0.11346000,K9PU33_99598_Cyanobacteria_B_Ter:0.11394000):0.05033000):0.02538000):0.04167000):0.02065000,A0A0D8ZRD9_1618023_Cyanobacteria_B_Ter:0.13516000):0.04401000):0.10545000):0.02958000):0.06716000):0.06802000,((I4EEM3_1129897_Chloroflexi_B_Ter:0.23363000,(A0A2Z3H6M4_114_Planctomycetes_B_Mar:0.38252000,A5UY95_357808_Chloroflexi_B_Ter:0.17908000):0.11003000):0.03020000,((A0A2S0NH98_1868589_Proteobacteria_B_Mar:0.18152000,A0A4D7B538_1940610_Proteobacteria_B_Mar:0.22727000):0.34775000,(A0A327L5R7_29409_Proteobacteria_B_Mar:0.16871000,(A0A0H1RDD0_1225564_Proteobacteria_B_Mar:0.20998000,A0A437M3F2_1979269_Proteobacteria_B_Mar:0.25243000):0.04807000):0.29548000):0.09245000):0.05008000):0.07547000,(E6SLN6_644966_Firmicutes_B_Ter:0.20658000,(A0A2T6BC70_1242148_Firmicutes_B_Ter:0.18553000,((A0A089IEX7_1536775_Firmicutes_B_Ter:0.17908000,A0A1M6L352_1830138_Firmicutes_B_Ter:0.13211000):0.06233000,A0A418MLK8_2048547_Firmicutes_B_Ter:0.20711000):0.04163000):0.04142000):0.04057000):0.01444000):0.02620000):0.01829000):0.03957000,((((((A0A1H3XW14_408074_Bacteroidetes_B_Mar:0.15274000,(A0A4Q2ZMN6_2044944_Bacteroidetes_B_Mar:0.24716000,A0A1R4L182_1434842_Bacteroidetes_B_Mar:0.22417000):0.04942000):0.02594000,(A0A191ZFD3_1860122_Proteobacteria_B_Mar:0.28655000,A0A2D0NBJ4_1122177_Bacteroidetes_B_Mar:0.39690000):0.03393000):0.02822000,(A0A0X3T641_1685380_Proteobacteria_B_Mar:0.25637000,A0A2Z2P4N8_1192854_Proteobacteria_B_Mar:0.44717000):0.14491000):0.04956000,((((((A0A0L8V3P2_1409788_Bacteroidetes_B_Mar:0.27394000,A0A1I1VUU8_385682_Bacteroidetes_B_Mar:0.25737000):0.14131000,(A0A0S7C2S3_1678841_Bacteroidetes_B_Mar:0.37097000,U5Q5M1_1400053_Bacteroidetes_B_Mar:0.34988000):0.12596000):0.12570000,(A0A0S7C7E3_1678841_Bacteroidetes_B_Mar:0.22411000,(A0A1G6RPM1_1640674_Bacteroidetes_B_Mar:0.51952000,(A0A0F5VHM9_265726_Proteobacteria_B_Mar:0.31694000,A0A380N0R1_13276_Proteobacteria_B_Mar:0.39327000):0.09229000):0.07989000):0.03768000):0.07544000,((D0J9E3_600809_Bacteroidetes_B_Mar:0.53398000,(A0A328YDD7_1101402_Bacteroidetes_B_Mar:0.52378000,A3J5A6_391598_Bacteroidetes_B_Mar:0.31602000):0.14848000):0.11570000,(A0A0S2HVA3_1307839_Bacteroidetes_B_Mar:0.55010000,A0A2D0NIH0_1122177_Bacteroidetes_B_Mar:0.37910000):0.05235000):0.05303000):0.04038000,(((A0A2K9NRY1_960_Proteobacteria_B_Mar:0.56773000,A3J209_391598_Bacteroidetes_B_Mar:0.30176000):0.13523000,((A0A1U9JNI9_1938604_Proteobacteria_B_Mar:0.31448000,Q7M864_273121_Proteobacteria_B_Mar:0.15450000):0.10095000,((((A0A2T5G1R0_1735121_Proteobacteria_B_Mar:0.17256000,A0A4Q5S3N2_1978525_Proteobacteria_B_Mar:0.17996000):0.02509000,(A0A192D580_1112_Proteobacteria_B_Mar:0.11106000,(A0A0B1Q2K7_370622_Proteobacteria_B_Mar:0.22905000,A0A3T0EBQ7_1434191_Proteobacteria_B_Mar:0.33565000):0.11574000):0.02569000):0.09739000,A0A2P2EAB2_1445552_Proteobacteria_B_Mar:0.34656000):0.09698000,Q08ZM8_378806_Proteobacteria_B_Mar:0.33322000):0.07279000):0.11974000):0.09507000,((A0A1R4IAU7_1434842_Bacteroidetes_B_Mar:0.71861000,(M7YBF0_1239962_Bacteroidetes_B_Mar:0.17396000,U5C1V6_1123057_Bacteroidetes_B_Mar:0.30710000):0.09980000):0.14486000,(H6L6A2_984262_Bacteroidetes_B_Mar:0.65285000,((A0A2N3IJD2_2016530_Bacteroidetes_B_Mar:0.34213000,A0A1I2GPG4_1003_Bacteroidetes_B_Mar:0.29720000):0.05960000,(A0A1H3Y937_408074_Bacteroidetes_B_Mar:0.26608000,I4AH06_880071_Bacteroidetes_B_Mar:0.44590000):0.09080000):0.09865000):0.02619000):0.04652000):0.02695000):0.04287000,((((A1ZTZ0_313606_Bacteroidetes_B_Mar:0.23125000,(((A0A3E1EU86_1737063_Bacteroidetes_B_Mar:0.09197000,((A6EQH5_50743_Bacteroidetes_B_Mar:0.19649000,Q26GK1_156586_Bacteroidetes_B_Mar:0.08544000):0.07164000,(C0BHJ2_487796_Bacteroidetes_B_Mar:0.24824000,(A0A328YCR8_1101402_Bacteroidetes_B_Mar:0.08603000,A3J2V1_391598_Bacteroidetes_B_Mar:0.11619000):0.02947000):0.04555000):0.05794000):0.07301000,(H6L8Y0_984262_Bacteroidetes_B_Mar:0.11579000,(A0A098SDX5_1524460_Bacteroidetes_B_Mar:0.06872000,A0A2D0ND73_1122177_Bacteroidetes_B_Mar:0.12671000):0.15172000):0.02353000):0.06217000,G8R729_926562_Bacteroidetes_B_Mar:0.20807000):0.04971000):0.01809000,((A0A1I1YWR4_385682_Bacteroidetes_B_Mar:0.11627000,(A0A1Y1CJJ9_1717717_Bacteroidetes_B_Mar:0.16063000,A0A0L8V2C5_1409788_Bacteroidetes_B_Mar:0.24854000):0.02903000):0.08249000,(A0A0D3LFT2_1257021_Bacteroidetes_B_Mar:0.18311000,I4APS4_880071_Bacteroidetes_B_Mar:0.21309000):0.06054000):0.04088000):0.02175000,(A0A1G9F980_1075417_Bacteroidetes_B_Mar:0.15960000,A0A1G9H772_1075417_Bacteroidetes_B_Mar:0.14881000):0.07716000):0.05345000,((M7Y218_1239962_Bacteroidetes_B_Mar:0.05563000,U5C2N4_1123057_Bacteroidetes_B_Mar:0.14051000):0.11043000,((A0A1I2HT21_1003_Bacteroidetes_B_Mar:0.14719000,(A0A085L1P1_1453500_Bacteroidetes_B_Mar:0.17035000,A0A2N3IHE2_2016530_Bacteroidetes_B_Mar:0.21142000):0.04572000):0.04215000,((A0A142L0Q1_1690483_Bacteroidetes_B_Mar:0.16613000,A0A257L3N9_2015582_Bacteroidetes_B_Mar:0.12622000):0.10631000,(A0A1I5ZIY7_1227077_Bacteroidetes_B_Mar:0.21898000,(A0A1G6HPU7_1640674_Bacteroidetes_B_Mar:0.25584000,B3ER22_452471_Bacteroidetes_B_Mar:0.37736000):0.06806000):0.05803000):0.01825000):0.04487000):0.06284000):0.04331000):0.02575000):0.00909000,(I0AK38_945713_Ignavibacteriae_B_Mar:0.25263000,I6ZUA8_1191523_Ignavibacteriae_B_Mar:0.17322000):0.11946000):0.07015000,(D3FDM4_469383_Actinobacteria_B_Ter:0.35025000,(A0A345UPY2_1457365_Bacteroidetes_B_Mar:0.25061000,(Q9RUV2_243230_Deinococcus-Thermus_B_Ter:0.18566000,((A0A242PAB3_1970738_Proteobacteria_B_Mar:0.22106000,V9H8J0_641147_Proteobacteria_B_Mar:0.28477000):0.04562000,((A0A085G4I0_910964_Proteobacteria_B_Mar:0.04561000,(((((A0A085HE47_1005999_Proteobacteria_B_Mar:0.05298000,A0A1I0EA93_1123402_Proteobacteria_B_Mar:0.10750000):0.01218000,A0A097R7C0_1453496_Proteobacteria_B_Mar:0.05565000):0.02506000,G7LTJ2_598467_Proteobacteria_B_Mar:0.05776000):0.01570000,A0A2G0QEY2_351679_Proteobacteria_B_Mar:0.21787000):0.01204000,((A0A0J8VRJ1_435910_Proteobacteria_B_Mar:0.02481000,(A0A085JMH6_1005995_Proteobacteria_B_Mar:0.03029000,A0A2I0FW47_2025587_Proteobacteria_B_Mar:0.11176000):0.03677000):0.04935000,Q1LSZ4_374463_Proteobacteria_B_Mar:0.43601000):0.02700000):0.00524000):0.03727000,(A0A1V3TPU9_1924934_Proteobacteria_B_Mar:0.18532000,D5VB54_1236608_Proteobacteria_B_Mar:0.19577000):0.09041000):0.05048000):0.11421000):0.06270000):0.04489000):0.04640000):0.04252000):0.04815000,(((A0A1B6B8G0_1048380_Firmicutes_B_Ter:0.45384000,(((Q2S1T9_309807_Bacteroidetes_B_Mar:0.38573000,A0A259TZ62_716817_Rhodothermaeota_B_Mar:0.30266000):0.01681000,U2EL32_1033802_Proteobacteria_B_Mar:0.51360000):0.06811000,((D7CSR0_649638_Deinococcus-Thermus_B_Ter:0.23088000,A0A0S2I4J1_1307839_Bacteroidetes_B_Mar:0.34138000):0.04042000,I0IDH9_1142394_Planctomycetes_B_Mar:0.44575000):0.02556000):0.03111000):0.03308000,(U2QZH4_1321779_Fusobacteria_B_Anc:0.19384000,(A0A4R1R4E5_1469948_Firmicutes_B_Ter:0.39805000,E1QXA5_633147_Actinobacteria_B_Ter:0.30849000):0.23559000):0.15418000):0.03211000,(A0A1H3A4M8_1123352_Firmicutes_B_Ter:0.10890000,M1ZG58_1288971_Firmicutes_B_Ter:0.05342000):0.34198000):0.01105000):0.02865000,(((W3Y1G4_936589_Firmicutes_B_Ter:0.36701000,(V6IV34_1395513_Firmicutes_B_Ter:0.38522000,V6J6E9_1395513_Firmicutes_B_Ter:0.26466000):0.09180000):0.06866000,Q6YQD2_262768_Tenericutes_B_Ter:0.60961000):0.05076000,((E4T355_694427_Bacteroidetes_B_Mar:0.35338000,(A0A142L7E6_1690483_Bacteroidetes_B_Mar:0.25561000,A0A257L9C2_2015582_Bacteroidetes_B_Mar:0.47669000):0.25182000):0.15403000,M1Z874_1288971_Firmicutes_B_Ter:0.20643000):0.09565000):0.04195000):0.01726000,(((A0A1L8ZD32_64897_Spirochaetes_B_Mar:0.00804000,A0A1L8ZDB1_64897_Spirochaetes_B_Mar:0.00680000):0.87678000,A0A1I1DPZ5_34097_Spirochaetes_B_Mar:0.34428000):0.10831000,(A0A0C7NIR4_1006576_Thermotogae_B_Anc:0.30086000,L8DAQ3_1268239_Proteobacteria_B_Mar:0.46249000):0.10866000):0.09157000):0.03274000,((A0A095XIK9_1230730_Firmicutes_B_Ter:0.30309000,A0A2Z3GPK6_114_Planctomycetes_B_Mar:0.40307000):0.19991000,(A0A2U1E3U8_46507_Firmicutes_B_Ter:0.53524000,((A0A0S7C1K2_1678841_Bacteroidetes_B_Mar:0.33750000,(((A0A0S2I3Z7_1307839_Bacteroidetes_B_Mar:0.47798000,(A0A1Y1CGP3_1717717_Bacteroidetes_B_Mar:0.23754000,R5NVX0_1262909_Bacteroidetes_B_Mar:0.70811000):0.04776000):0.04135000,A0A0L8V2C9_1409788_Bacteroidetes_B_Mar:0.30479000):0.05313000,((A0A1G6HQ17_1640674_Bacteroidetes_B_Mar:0.31763000,A0A1I1UAP9_385682_Bacteroidetes_B_Mar:0.14203000):0.10872000,((((A0A1Y3VAM6_1965650_Bacteroidetes_B_Mar:0.31176000,A0A1Y3VCW3_1965650_Bacteroidetes_B_Mar:0.65531000):0.14300000,(A0A2X0WVT3_179995_Proteobacteria_B_Mar:0.78428000,A0A1Y4C9N7_1965623_Bacteroidetes_B_Mar:0.26650000):0.17250000):0.26323000,(((R5JCK6_1262737_Bacteroidetes_B_Mar:0.23771000,(A0A1I2LRE0_1855325_Bacteroidetes_B_Mar:0.23365000,S8FFI4_888054_Bacteroidetes_B_Mar:0.23898000):0.13794000):0.09933000,((A0A0A2F0U5_1515615_Bacteroidetes_B_Mar:0.34795000,D7JFA1_575590_Bacteroidetes_B_Mar:0.17442000):0.08629000,(A0A0F5IRT0_1203610_Bacteroidetes_B_Mar:0.07149000,(A0A0A2F1H1_1515615_Bacteroidetes_B_Mar:0.45704000,A0A2V3PRT9_1605892_Bacteroidetes_B_Mar:0.21545000):0.04798000):0.06370000):0.14487000):0.08356000,(W0ERB5_880074_Bacteroidetes_B_Mar:0.47390000,(R5P756_1262909_Bacteroidetes_B_Mar:0.42660000,R5PAP5_1262909_Bacteroidetes_B_Mar:0.22364000):0.09344000):0.06282000):0.07611000):0.08934000,E4T0E9_694427_Bacteroidetes_B_Mar:0.21316000):0.17156000):0.03117000):0.03243000):0.08727000,(((A0A2Z2L2V6_549298_Proteobacteria_B_Mar:0.34542000,((A0A1V0RDW6_28898_Proteobacteria_B_Mar:0.36918000,Q7M8L0_273121_Proteobacteria_B_Mar:0.39450000):0.23840000,A0A395JG70_644221_Proteobacteria_B_Mar:0.83252000):0.10394000):0.02208000,((A0A2D3WM99_2015906_Proteobacteria_B_Mar:0.41796000,A8PPX3_59196_Proteobacteria_B_Mar:0.35821000):0.13003000,((G2E6T6_765913_Proteobacteria_B_Mar:0.42881000,I2K993_1165841_Proteobacteria_B_Mar:0.50818000):0.14169000,(F8L8N3_331113_Chlamydiae_B_Mar:0.42216000,(A1ZNK9_313606_Bacteroidetes_B_Mar:0.11462000,(A0A1R4KBB1_1434842_Bacteroidetes_B_Mar:0.07672000,A0A1R4KQ40_1434842_Bacteroidetes_B_Mar:0.06704000):0.13845000):0.25402000):0.12385000):0.09381000):0.07705000):0.03665000,(((((((((D0LUJ8_502025_Proteobacteria_B_Mar:0.36975000,E1X0Y3_862908_Proteobacteria_B_Mar:0.29571000):0.12145000,(A0A1Z4VNQ5_585455_Proteobacteria_B_Mar:0.16341000,(W0TLC1_1076588_Proteobacteria_B_Mar:0.16846000,(A0A0B0HBS3_2340_Proteobacteria_B_Mar:0.00225000,A0A1T2L771_1918949_Proteobacteria_B_Mar:0.04240000):0.24510000):0.03093000):0.08962000):0.00967000,(G2E543_765913_Proteobacteria_B_Mar:0.15520000,((A0A1T2KSF0_1918948_Proteobacteria_B_Mar:0.11864000,A0A0F7K213_1543721_Proteobacteria_B_Mar:0.13992000):0.03126000,(A0A1E2ZEY7_1655433_Proteobacteria_B_Mar:0.02854000,(A0A1E2V0Q9_1818881_Proteobacteria_B_Mar:0.01722000,G2FFW9_1049564_Proteobacteria_B_Mar:0.07833000):0.01868000):0.15190000):0.02075000):0.03908000):0.03659000,((A0A1R3VN56_233100_Proteobacteria_B_Mar:0.17669000,B5JWC0_391615_Proteobacteria_B_Mar:0.19810000):0.10316000,((((A0A0B4XLG0_391936_Proteobacteria_B_Mar:0.18447000,(((A0A1H4F8V2_152573_Proteobacteria_B_Mar:0.10385000,L8D9X1_1268239_Proteobacteria_B_Mar:0.15235000):0.04546000,(A0A0S2JHC6_58049_Proteobacteria_B_Mar:0.15656000,(A0A2N0WW65_2058089_Proteobacteria_B_Mar:0.15660000,A0A090IF69_80854_Proteobacteria_B_Mar:0.13730000):0.03394000):0.03275000):0.02230000,(A4BGS7_314283_Proteobacteria_B_Mar:0.11593000,(A0A1H6CZE7_568106_Proteobacteria_B_Mar:0.11367000,((A0A162GML5_1822219_Proteobacteria_B_Mar:0.11025000,A0A081NMU1_1137799_Proteobacteria_B_Mar:0.16690000):0.03618000,I2JH08_1168065_Proteobacteria_B_Mar:0.21870000):0.03049000):0.01031000):0.07568000):0.03667000):0.04821000,(((((A0A097R2J1_1453496_Proteobacteria_B_Mar:0.04206000,(A0A2G0Q1U7_351679_Proteobacteria_B_Mar:0.15336000,A0A085HJD6_1005999_Proteobacteria_B_Mar:0.10118000):0.03115000):0.03956000,E1VHB8_83406_Proteobacteria_B_Mar:0.14200000):0.02355000,K2JEV8_745411_Proteobacteria_B_Mar:0.09383000):0.05178000,((A8H3Z8_398579_Proteobacteria_B_Mar:0.13161000,(A0A0F5VGV2_265726_Proteobacteria_B_Mar:0.11642000,E1SRJ0_550540_Proteobacteria_B_Mar:0.06427000):0.04406000):0.03297000,(A0A2S1JPT9_359370_Proteobacteria_B_Mar:0.11903000,(A0A1Y0FVA0_1987723_Proteobacteria_B_Mar:0.05699000,A0A4Q5V9J9_1913989_Proteobacteria_B_Mar:0.10070000):0.07188000):0.04636000):0.00960000):0.03083000,(A0A094J7J3_1517416_Proteobacteria_B_Mar:0.08493000,(A0A1E2V9J2_197479_Proteobacteria_B_Mar:0.16847000,A0A2A5LZ91_2039467_Proteobacteria_B_Mar:0.12239000):0.04502000):0.04538000):0.03643000):0.02401000,A0A4V2PNM7_412034_Proteobacteria_B_Mar:0.16312000):0.02550000,(A0A395JH00_644221_Proteobacteria_B_Mar:0.13060000,C7RD31_523791_Proteobacteria_B_Mar:0.04211000):0.15891000):0.04252000):0.00915000):0.02150000,A0A2K8LAI0_1921087_Proteobacteria_B_Mar:0.23143000):0.04967000,(((A0A250KTZ9_1432792_Proteobacteria_B_Mar:0.12969000,(A0A251X438_1570016_Proteobacteria_B_Mar:0.18032000,(A0A1B1YPZ1_1810504_Proteobacteria_B_Mar:0.18586000,((A0A1R4HJV0_360316_Proteobacteria_B_Mar:0.05017000,A0A1Z5HC40_113268_Proteobacteria_B_Mar:0.15592000):0.04156000,(A0A1J4QJC3_1414654_Proteobacteria_B_Mar:0.13506000,(A0A139SPB8_1680762_Proteobacteria_B_Mar:0.11093000,A0A1E4UW72_1843690_Proteobacteria_B_Mar:0.07490000):0.04568000):0.05360000):0.02191000):0.02217000):0.00771000):0.02440000,(A0A0A6PC03_1003181_Proteobacteria_B_Mar:0.14779000,W6M2C8_1400863_Proteobacteria_B_Mar:0.16251000):0.02543000):0.02468000,((((((V5ADP9_1408164_Proteobacteria_B_Mar:0.12672000,(A0A0N0JCV7_1523424_Proteobacteria_B_Mar:0.03971000,A0A0U3DNG9_1768242_Proteobacteria_B_Mar:0.09736000):0.02695000):0.04200000,A0A2P8KDF2_1105204_Proteobacteria_B_Mar:0.10322000):0.04320000,(((H0PZQ1_748247_Proteobacteria_B_Mar:0.07730000,A0A0K6IWK3_876478_Proteobacteria_B_Mar:0.15835000):0.04779000,(((A0A1R1I0D0_418702_Proteobacteria_B_Mar:0.05030000,(A0A401JC34_1559896_Proteobacteria_B_Mar:0.06270000,Q3SK26_292415_Proteobacteria_B_Mar:0.03312000):0.04113000):0.01764000,((A0A011QCI9_1454004_Proteobacteria_B_Mar:0.04120000,((A0A1A8XY42_1860102_Proteobacteria_B_Mar:0.05299000,C7RIZ7_522306_Proteobacteria_B_Mar:0.01980000):0.04627000,(A0A080M9P3_1453999_Proteobacteria_B_Mar:0.03749000,(A0A011NKF6_1454000_Proteobacteria_B_Mar:0.02400000,A0A011MHB8_1454001_Proteobacteria_B_Mar:0.04113000):0.06529000):0.01258000):0.01604000):0.05686000,A0A1G8BH66_83767_Proteobacteria_B_Mar:0.02095000):0.04757000):0.02649000,A0A497X9T4_1381557_Proteobacteria_B_Mar:0.01328000):0.03892000):0.05126000,A0A0Q6BJ75_1736373_Proteobacteria_B_Mar:0.06502000):0.04642000):0.05200000,(K1JSW8_742823_Proteobacteria_B_Mar:0.30367000,R6A170_1262986_Proteobacteria_B_Mar:0.31276000):0.06456000):0.03577000,(((A0A2I8DQ40_1758194_Proteobacteria_B_Mar:0.05963000,(A0A2T0XJH1_323426_Proteobacteria_B_Mar:0.08256000,(M1L4D0_1208918_Proteobacteria_B_Mar:0.24560000,M1M096_1208922_Proteobacteria_B_Mar:0.35021000):0.17583000):0.01715000):0.03860000,((A0A2G3K1V1_1559339_Proteobacteria_B_Mar:0.21224000,(A0A1U9JL93_1938604_Proteobacteria_B_Mar:0.07045000,(A0A2U2AEQ9_472582_Proteobacteria_B_Mar:0.24862000,(A0A142LJU4_1690485_Proteobacteria_B_Mar:0.27981000,A0A149W187_1789004_Proteobacteria_B_Mar:0.50488000):0.05899000):0.06348000):0.04308000):0.04200000,A0A0N1LB93_1523428_Proteobacteria_B_Mar:0.13239000):0.04365000):0.03366000,L9PNM3_1198452_Proteobacteria_B_Mar:0.12883000):0.00936000):0.09875000,A0A401JA31_1559896_Proteobacteria_B_Mar:0.12649000):0.04445000):0.01707000):0.02940000,((A0A1V3PIV2_1945854_Proteobacteria_B_Mar:0.19983000,(A0A0D0S5X1_1199154_Proteobacteria_B_Mar:0.16988000,A0A2P1PVM9_2021234_Proteobacteria_B_Mar:0.15167000):0.06258000):0.06624000,(A0A0W0TNM8_453_Proteobacteria_B_Mar:0.18704000,A0A1M5RQ54_490188_Proteobacteria_B_Mar:0.28340000):0.03850000):0.01591000):0.04488000,((A0A1Y2K534_1434232_Proteobacteria_B_Mar:0.18864000,(A0A0K0XVJ8_1579979_Proteobacteria_B_Mar:0.15235000,(A0A1Y6CDA5_1513793_Proteobacteria_B_Mar:0.12899000,(A0A0F6YK52_927083_Proteobacteria_B_Mar:0.19985000,(A0A1L6KZF6_888845_Proteobacteria_B_Mar:0.04039000,A9EWL0_448385_Proteobacteria_B_Mar:0.09410000):0.09927000):0.19573000):0.07954000):0.05704000):0.08335000,A0A4Q6B980_1977087_Proteobacteria_B_Mar:0.25327000):0.02515000):0.05311000,((Q6MHD3_264462_Proteobacteria_B_Mar:0.66901000,(((C8Q0X7_553217_Proteobacteria_B_Mar:0.30097000,X5MEY9_1458461_Proteobacteria_B_Mar:0.40855000):0.10726000,((((((A0A0C2QFL4_1233231_Cyanobacteria_B_Ter:0.07842000,A0A1Y0RVB5_1940762_Cyanobacteria_B_Ter:0.10060000):0.16363000,((A0A1E5QE38_1781255_Cyanobacteria_B_Ter:0.11605000,(A0A1J0A9M2_1188229_Cyanobacteria_B_Ter:0.29905000,K9SBX7_1173025_Cyanobacteria_B_Ter:0.24213000):0.07405000):0.02925000,(B7KAA9_65393_Cyanobacteria_B_Ter:0.09497000,(((P77968_1111708_Cyanobacteria_B_Ter:0.06698000,(A0A0M2Q238_317619_Cyanobacteria_B_Ter:0.11820000,B0JGF5_449447_Cyanobacteria_B_Ter:0.07720000):0.03510000):0.08416000,(K9Z4N7_755178_Cyanobacteria_B_Ter:0.16602000,(K9XY88_111780_Cyanobacteria_B_Ter:0.06958000,L8LWB9_102125_Cyanobacteria_B_Ter:0.06226000):0.05970000):0.03525000):0.01824000,K9T440_118163_Cyanobacteria_B_Ter:0.08273000):0.02877000):0.07393000):0.06483000):0.04048000,((A0A1C0VXQ7_1880991_Cyanobacteria_B_Ter:0.15761000,(A0A073CMZ0_388467_Cyanobacteria_B_Ter:0.08467000,A0YJL0_313612_Cyanobacteria_B_Ter:0.20921000):0.05286000):0.07534000,((A0A2N3PTI0_382514_Proteobacteria_B_Mar:0.24962000,A0A3N1MAT8_94_Proteobacteria_B_Mar:0.18403000):0.08203000,(K9VXC7_1173022_Cyanobacteria_B_Ter:0.08883000,((((A0A367QB34_1844469_Cyanobacteria_B_Ter:0.04554000,(A0A433N6A6_211165_Cyanobacteria_B_Ter:0.09332000,G6G070_372787_Cyanobacteria_B_Ter:0.03612000):0.04607000):0.00938000,(K9PE47_99598_Cyanobacteria_B_Ter:0.07918000,A0A0C1MX08_1479485_Cyanobacteria_B_Ter:0.05473000):0.02045000):0.03466000,A0A1Z4UU62_1973480_Cyanobacteria_B_Ter:0.07418000):0.20043000,K9UQB8_1173020_Cyanobacteria_B_Ter:0.21929000):0.03056000):0.05154000):0.05795000):0.03290000):0.04759000,((A0A4P8L1J4_980445_Proteobacteria_B_Mar:0.33549000,Q3YRT4_269484_Proteobacteria_B_Mar:0.57335000):0.04257000,((B3EJL9_331678_Chlorobi_B_Mar:0.38524000,(((A0A1G6E3R2_617002_Proteobacteria_B_Mar:0.40438000,(Q3SGN8_292415_Proteobacteria_B_Mar:0.57475000,(((C7LNS7_525897_Proteobacteria_B_Mar:0.24118000,C7LRC1_525897_Proteobacteria_B_Mar:0.20543000):0.07840000,((A0A4R8ITH7_381308_Proteobacteria_B_Mar:0.34009000,(A0A1G6EXC4_617002_Proteobacteria_B_Mar:0.13623000,A0A1K1LIX2_1855339_Proteobacteria_B_Mar:0.17211000):0.04018000):0.01425000,A0A2S6NHJ6_333368_Proteobacteria_B_Mar:0.37196000):0.00211000):0.02391000,A0A250KYU0_1432792_Proteobacteria_B_Mar:0.17931000):0.02663000):0.06736000):0.03572000,(W6M9I9_1400863_Proteobacteria_B_Mar:0.23363000,(((A0A0N0K024_1523432_Proteobacteria_B_Mar:0.26696000,A0A257EMF7_2015572_Proteobacteria_B_Mar:0.26859000):0.14087000,(A0A2N3PQX2_382514_Proteobacteria_B_Mar:0.33929000,A0A1T4W9E9_1121442_Proteobacteria_B_Mar:0.76989000):0.17277000):0.06553000,A0A1V1PIR0_1605283_Proteobacteria_B_Mar:0.77966000):0.10671000):0.07138000):0.06780000,(A0A1R4H9Z6_360316_Proteobacteria_B_Mar:0.17428000,A0A1R4HH45_360316_Proteobacteria_B_Mar:0.17994000):0.43265000):0.06400000):0.04724000,(A0A1P8FPF1_1904640_Proteobacteria_B_Mar:0.48196000,(A0A4Q5XXF3_1913988_Proteobacteria_B_Mar:0.27625000,U5QI99_1183438_Cyanobacteria_B_Ter:0.28767000):0.09968000):0.08347000):0.05651000):0.11318000):0.01941000,((A0A1Y5TAA6_745714_Proteobacteria_B_Mar:0.14694000,A0A3M0CGB4_911205_Proteobacteria_B_Mar:0.23847000):0.08841000,(A0A0M4CZS5_1603606_Proteobacteria_B_Mar:0.30470000,(C7LSI9_525897_Proteobacteria_B_Mar:0.29068000,A0A091FCF4_1499107_Proteobacteria_B_Mar:0.21919000):0.07635000):0.08929000):0.06213000):0.02521000,((Q9ZD15_272947_Proteobacteria_B_Mar:0.68846000,(A0A077FIP1_1528098_Proteobacteria_B_Mar:0.17207000,A0A0C1QWB0_86105_Proteobacteria_B_Mar:0.36661000):0.09391000):0.11224000,(((A0A1Y5U185_745714_Proteobacteria_B_Mar:0.35697000,((A0A192D2N1_1112_Proteobacteria_B_Mar:0.56935000,(A0A2T5FTW8_1735121_Proteobacteria_B_Mar:0.32808000,A0A4Q5RR52_1978525_Proteobacteria_B_Mar:0.22227000):0.22858000):0.15932000,((A0A257J713_2015570_Proteobacteria_B_Mar:0.58305000,A0A4Q3S5H0_1978230_Proteobacteria_B_Mar:0.35162000):0.24090000,A0A193LCR6_1548547_Proteobacteria_B_Mar:0.54404000):0.10631000):0.11595000):0.07754000,((A0A0S2KFC6_1249552_Proteobacteria_B_Mar:0.04201000,A0A1E8CFB9_1524254_Proteobacteria_B_Mar:0.07065000):0.30883000,E0TI36_314260_Proteobacteria_B_Mar:0.48943000):0.12085000):0.05390000,((A0A061Q889_1492281_Proteobacteria_B_Mar:0.40261000,R5L6H9_1262760_Spirochaetes_B_Mar:0.52475000):0.11653000,((((S9QPA8_1123237_Proteobacteria_B_Mar:0.14072000,(D5BTR8_488538_Proteobacteria_B_Mar:0.15146000,(G6A1M0_909943_Proteobacteria_B_Mar:0.12607000,Q0F8L9_367336_Proteobacteria_B_Mar:0.12724000):0.04217000):0.05589000):0.34660000,A8U258_331869_Proteobacteria_B_Mar:0.18792000):0.02356000,(A0A0M2R958_1549748_Proteobacteria_B_Mar:0.14389000,(((((A0A2S6N6L1_333368_Proteobacteria_B_Mar:0.24604000,A0A366ENT2_1473586_Proteobacteria_B_Mar:0.18877000):0.15701000,(A0A2S0N786_1868589_Proteobacteria_B_Mar:0.07540000,A0A4D7B883_1940610_Proteobacteria_B_Mar:0.14498000):0.07299000):0.02811000,(((A0A0H1RD07_1225564_Proteobacteria_B_Mar:0.07033000,A0A327KT03_29409_Proteobacteria_B_Mar:0.15533000):0.05210000,A0A4R2GX74_659006_Proteobacteria_B_Mar:0.17428000):0.03472000,H0TES2_551947_Proteobacteria_B_Mar:0.20958000):0.02243000):0.06628000,(A0A1I4SFU3_1166257_Proteobacteria_B_Mar:0.17389000,((A0A285MB93_1798205_Proteobacteria_B_Mar:0.16471000,F2IV67_991905_Proteobacteria_B_Mar:0.08241000):0.04689000,((A0A256FXP0_571255_Proteobacteria_B_Mar:0.12079000,(((A0A231UUB0_1876515_Proteobacteria_B_Mar:0.37744000,A0A0B1Q8Z2_370622_Proteobacteria_B_Mar:0.07228000):0.07584000,A0A090FD11_1505946_Proteobacteria_B_Mar:0.08116000):0.08452000,W3TXZ6_1402976_Proteobacteria_B_Mar:0.26699000):0.03310000):0.06171000,(A0A1E3VXW9_1774968_Proteobacteria_B_Mar:0.26685000,G2KRC6_856793_Proteobacteria_B_Mar:0.36400000):0.02757000):0.07708000):0.05304000):0.07445000):0.08363000,A0A4R2PEU5_1188247_Proteobacteria_B_Mar:0.41607000):0.04114000):0.04760000):0.06826000,(D0RR50_684719_Proteobacteria_B_Mar:0.29158000,(A0A437MD01_1979269_Proteobacteria_B_Mar:0.44095000,(A0A4R7K0K2_332522_Proteobacteria_B_Mar:0.17143000,(A0A4D7B4V5_1940610_Proteobacteria_B_Mar:0.25312000,(A0A285M297_1798205_Proteobacteria_B_Mar:0.06876000,S9RPC2_1123237_Proteobacteria_B_Mar:0.12728000):0.15944000):0.09226000):0.13205000):0.20385000):0.05806000):0.10166000):0.14377000):0.04651000):0.03493000):0.01230000):0.11496000,(((A0A0H4WNU9_1297742_Proteobacteria_B_Mar:0.07045000,(Q09CK0_378806_Proteobacteria_B_Mar:0.17372000,A0A0K1PVI4_1391654_Proteobacteria_B_Mar:0.07036000):0.02965000):0.04005000,A0A1I2A523_54_Proteobacteria_B_Mar:0.13807000):0.02228000,A0A0K1PWM2_1391654_Proteobacteria_B_Mar:0.14011000):0.16611000):0.04830000):0.03768000,(A0A2K9NQW2_960_Proteobacteria_B_Mar:0.39542000,Q6MKI3_264462_Proteobacteria_B_Mar:0.22895000):0.09595000):0.04770000):0.07882000):0.09796000):0.19665000):0.18671000):0.06288000):0.07414000,((A0A062XUH1_1312852_Acidobacteria_B_Mar:0.35038000,A0A1J0ADU4_1188229_Cyanobacteria_B_Ter:0.39734000):0.14830000,((A0A1B4XH23_1620215_Proteobacteria_B_Mar:0.65001000,(D7CXL9_649638_Deinococcus-Thermus_B_Ter:0.58175000,D7CR17_649638_Deinococcus-Thermus_B_Ter:0.35068000):0.20189000):0.09870000,(((R6GYH2_1262911_Firmicutes_B_Ter:1.15095000,(A0A2P2BQH2_1507512_Firmicutes_B_Ter:0.72341000,(R7QTT1_1262942_Firmicutes_B_Ter:0.75521000,R5HFY3_1263001_Firmicutes_B_Ter:0.42601000):0.04629000):0.04653000):0.02768000,R6EU58_1262994_Firmicutes_B_Ter:0.71110000):0.05051000,A0A2P2BST4_1507512_Firmicutes_B_Ter:0.60515000):0.16403000):0.06098000):0.05068000):0.27036000):0.15399000,(((((O93724_178306_Thermoprotei_A_Arc:0.01616000,(A1RW25_384616_Thermoprotei_A_Arc:0.04804000,G7VBY8_1104324_Thermoprotei_A_Arc:0.03211000):0.02226000):0.12651000,(Q9Y8H8_272557_Thermoprotei_A_Arc:0.15632000,(D9Q0R7_666510_Thermoprotei_A_Arc:0.13536000,L0AA53_1056495_Thermoprotei_A_Arc:0.12464000):0.24160000):0.10502000):0.09496000,((F2KQT8_693661_Archaeoglobi_A_Arc:0.28645000,((A0A498GYD6_1550565_Methanomicrobia_A_Mar:0.19776000,(A0A0X3BJM4_86622_Methanomicrobia_A_Mar:0.00601000,I7KYD2_1201294_Methanomicrobia_A_Arc:0.00469000):0.12488000):0.10430000,((A0B701_349307_Methanomicrobia_A_Arc:0.22725000,(F4BZF7_990316_Methanomicrobia_A_Arc:0.08478000,G7WL45_1110509_Methanomicrobia_A_Arc:0.10497000):0.10897000):0.06243000,(((A7I8G0_456442_Methanomicrobia_A_Arc:0.10468000,L0HJ20_593750_Methanomicrobia_A_Arc:0.14277000):0.07678000,(Q2FSC2_323259_Methanomicrobia_A_Arc:0.28934000,(A7I921_456442_Methanomicrobia_A_Arc:0.15115000,L0HEM7_593750_Methanomicrobia_A_Arc:0.10087000):0.05059000):0.06861000):0.08384000,((P18868_187420_Methanobacteria_A_Arc:0.18456000,(U6EAT7_1379702_Methanobacteria_A_Arc:0.16455000,(A0A1D2W9T7_1860100_Methanobacteria_A_Arc:0.28038000,(A0A1D3L2G7_118062_Methanobacteria_A_Arc:0.12590000,F0TCH4_877455_Methanobacteria_A_Arc:0.15086000):0.06153000):0.03286000):0.07243000):0.17922000,((F7XLC9_679901_Methanomicrobia_A_Arc:0.27801000,(A0A0E3WWB9_1434107_Methanomicrobia_A_Mar:0.08531000,((A0A0E3NWV4_1434100_Methanomicrobia_A_Mar:0.11239000,(K4MC62_1094980_Methanomicrobia_A_Arc:0.09703000,L0KZ58_867904_Methanomicrobia_A_Arc:0.10504000):0.01336000):0.04482000,(A0A0E3NRG6_1434102_Methanomicrobia_A_Mar:0.04745000,Q8TQG9_188937_Methanomicrobia_A_Arc:0.05906000):0.01415000):0.04121000):0.07506000):0.26232000,A0A0Q4BCN0_1713724_Thermoplasmata_A_Arc:0.50825000):0.04858000):0.06162000):0.03617000):0.10168000):0.16519000):0.11576000,((A8MAM7_397948_Thermoprotei_A_Arc:0.07154000,E1QRN4_572478_Thermoprotei_A_Arc:0.10635000):0.22575000,(T0LM88_667138_Thermoplasmata_A_Arc:0.30576000,((P80857_273057_Thermoprotei_A_Arc:0.06199000,Q08713_330779_Thermoprotei_A_Arc:0.11150000):0.02226000,((((Q96Y84_273063_Thermoprotei_A_Arc:0.04165000,(A0A2U9IFV2_41673_Thermoprotei_A_Arc:0.06284000,(F4B3P9_933801_Thermoprotei_A_Arc:0.06650000,A0A031LPG5_1160895_Thermoprotei_A_Arc:0.08336000):0.05507000):0.03894000):0.02617000,W7KP27_1326980_Thermoprotei_A_Arc:0.04559000):0.02783000,(A4YHX7_399549_Thermoprotei_A_Arc:0.02661000,H2C9I7_671065_Thermoprotei_A_Arc:0.03799000):0.07668000):0.03333000,A0A348B0W0_1670455_Thermoprotei_A_Arc:0.13344000):0.00941000):0.02807000):0.09279000):0.47001000):0.05207000):0.32913000,((A0A3P3RLZ3_671145_Halobacteria_A_Arc:0.29736000,(A0A365T5J1_29295_Halobacteria_A_Arc:0.03368000,A0A365T5J6_29295_Halobacteria_A_Arc:0.00921000):0.16927000):0.12299000,((A0A1H6VX19_1073996_Halobacteria_A_Arc:0.20562000,(A0A1I2QXX4_553467_Halobacteria_A_Arc:0.13353000,(A0A0W1R5I9_1514971_Halobacteria_A_Arc:0.07473000,(A0A365TF12_29295_Halobacteria_A_Arc:0.08104000,(A0A166S5V7_1679489_Halobacteria_A_Arc:0.09630000,A0A1N6WZ27_553468_Halobacteria_A_Arc:0.05036000):0.02440000):0.02394000):0.10025000):0.05744000):0.12024000,(A0A1N7AFB9_588898_Halobacteria_A_Arc:0.26092000,(M0AV65_29540_Halobacteria_A_Arc:0.18608000,((D2S0I8_543526_Halobacteria_A_Arc:0.10300000,L0JXL3_694430_Halobacteria_A_Arc:0.05984000):0.16436000,(L9XNL7_1227499_Halobacteria_A_Arc:0.07054000,M0LBE9_1227454_Halobacteria_A_Arc:0.23519000):0.03780000):0.06125000):0.08616000):0.19544000):0.06394000):0.09617000):0.04381000,((((A0A1M5PRJ6_490188_Proteobacteria_B_Mar:0.61617000,(((A0A1P8FEK9_1904640_Proteobacteria_B_Mar:0.28274000,A0A1V1PJD5_1605283_Proteobacteria_B_Mar:0.40439000):0.30521000,A0A4D7B288_1940610_Proteobacteria_B_Mar:0.31862000):0.04114000,((A0A0N1BGP9_1523432_Proteobacteria_B_Mar:0.24782000,(A0A257J8A7_2015570_Proteobacteria_B_Mar:0.16790000,A0A2T5FZN3_1735121_Proteobacteria_B_Mar:0.10133000):0.12910000):0.28907000,A0A2P8KJW4_1105204_Proteobacteria_B_Mar:0.25291000):0.03935000):0.29700000):0.49593000,((K0AYP0_1128398_Firmicutes_B_Ter:1.20500000,((A0A1L4CZX9_1915309_Proteobacteria_B_Mar:0.65069000,(A0A0K1P8N3_1391653_Proteobacteria_B_Mar:0.26409000,(A0A1L6LK10_888845_Proteobacteria_B_Mar:0.19337000,A9GL55_448385_Proteobacteria_B_Mar:0.19136000):0.16007000):0.40647000):0.12619000,(((((A0A062V7A0_1392998_Methanomicrobia_A_Mar:0.16000000,A0A062V9F8_1392998_Methanomicrobia_A_Mar:0.15024000):0.31974000,((D8JZF0_552811_Chloroflexi_B_Ter:0.28507000,(A0A0W0GJW2_1217799_Chloroflexi_B_Ter:0.19534000,A0A1P8F872_1839801_Chloroflexi_B_Ter:0.16897000):0.06262000):0.15248000,Q3Z7W8_243164_Chloroflexi_B_Ter:0.35026000):0.06027000):0.04611000,A0A0C9Q165_1197129_Planctomycetes_B_Mar:0.19645000):0.20026000,(F8L7W5_331113_Chlamydiae_B_Mar:0.55159000,(A0A090CZB7_1437425_Chlamydiae_B_Mar:0.34217000,F8L0F2_765952_Chlamydiae_B_Mar:0.23154000):0.18480000):0.21217000):0.16355000,(A0A1I1XIF6_54_Proteobacteria_B_Mar:0.71200000,(A7HBL1_404589_Proteobacteria_B_Mar:0.23668000,Q09C50_378806_Proteobacteria_B_Mar:0.35767000):0.27399000):0.23241000):0.47192000):0.19082000):0.10171000,((B4U6J9_380749_Aquificae_B_Anc:0.19540000,C1DW98_204536_Aquificae_B_Anc:0.21577000):0.28200000,((A0A218ZVX8_1961136_Thermoplasmata_A_Arc:0.07532000,(T0MPA5_667137_Thermoplasmata_A_Arc:0.13174000,((Q9HM56_273075_Thermoplasmata_A_Arc:0.19950000,(Q6L1T7_263820_Thermoplasmata_A_Arc:0.00736000,(A0A0N8VL92_312540_Thermoplasmata_A_Arc:0.03785000,S0AS08_333146_Thermoplasmata_A_Arc:0.08354000):0.05362000):0.16082000):0.12286000,T0M4N9_667135_Thermoplasmata_A_Arc:0.10805000):0.01775000):0.06501000):0.06807000,T0MZU0_261391_Thermoplasmata_A_Arc:0.11370000):0.19861000):0.23458000):0.26143000):0.57921000,((A0A1D8S4L0_1873524_Halobacteria_A_Arc:0.14281000,I3RA82_523841_Halobacteria_A_Arc:0.21096000):0.06212000,(((((A0A1I0MM22_355548_Halobacteria_A_Arc:0.03131000,P09224_64091_Halobacteria_A_Arc:0.14934000):0.01910000,(A0A0F7PDN8_1604004_Halobacteria_A_Arc:0.10993000,(A0A0U5H1X7_1407499_Halobacteria_A_Arc:0.03955000,(A0A0U5CYX4_1407499_Halobacteria_A_Arc:0.09341000,(((A0A285P0H4_558529_Halobacteria_A_Arc:0.02785000,((F8DDU6_797210_Halobacteria_A_Arc:0.01195000,A0A1G9XG20_660521_Halobacteria_A_Arc:0.06813000):0.01752000,((C7P4U1_485914_Halobacteria_A_Arc:0.06010000,M0B3T7_29540_Halobacteria_A_Arc:0.04647000):0.01331000,((((((V4HNI7_1324957_Halobacteria_A_Arc:0.08770000,M0DHS9_1227487_Halobacteria_A_Arc:0.04474000):0.03658000,(A0A1H6VKK8_1073996_Halobacteria_A_Arc:0.08959000,A0A0W1R4R4_1514971_Halobacteria_A_Arc:0.04264000):0.01944000):0.03451000,A0A1G9SJT8_660521_Halobacteria_A_Arc:0.03188000):0.00852000,((A0A3P3RGH5_671145_Halobacteria_A_Arc:0.05274000,(A0A2I8VJM1_755307_Halobacteria_A_Arc:0.00055000,A0A2I8VJQ7_755307_Halobacteria_A_Arc:0.01016000):0.06297000):0.02091000,(((A0A1V4C128_1853690_Halobacteria_A_Arc:0.01062000,A0A1V4C962_1853690_Halobacteria_A_Arc:0.00518000):0.02492000,(A0A0N8HZX4_699431_Halobacteria_A_Arc:0.00689000,A0A0P7GPP0_699431_Halobacteria_A_Arc:0.05880000):0.02185000):0.04202000,((I3R8L9_523841_Halobacteria_A_Arc:0.00619000,I3RBB8_523841_Halobacteria_A_Arc:0.01498000):0.03093000,((Q03300_309800_Halobacteria_A_Arc:0.01063000,Q03301_309800_Halobacteria_A_Arc:0.00055000):0.00854000,(A0A0W1SLK0_1544718_Halobacteria_A_Arc:0.01268000,A0A0W1RIP5_1544718_Halobacteria_A_Arc:0.05257000):0.01548000):0.01622000):0.01965000):0.00748000):0.01566000):0.03973000,((A0A345E419_1547899_Halobacteria_A_Arc:0.01580000,A0A368N5W7_1126245_Halobacteria_A_Arc:0.04777000):0.02122000,(A0A1H3W6N9_555874_Halobacteria_A_Arc:0.01077000,E4NT27_469382_Halobacteria_A_Arc:0.04382000):0.00593000):0.01601000):0.01605000,(A0A1H6AL08_699433_Halobacteria_A_Arc:0.01009000,((A0A1H3ILX3_660517_Halobacteria_A_Arc:0.01020000,(Q18HG3_362976_Halobacteria_A_Arc:0.10044000,U1QK35_1070774_Halobacteria_A_Arc:0.09038000):0.03614000):0.02135000,(A0A2G1X3V2_1483399_Halobacteria_A_Arc:0.02144000,((A0A238VTU4_63740_Halobacteria_A_Arc:0.04872000,A0A256IHQ1_1383851_Halobacteria_A_Arc:0.00517000):0.01036000,((A0A081EVB3_2248_Halobacteria_A_Arc:0.00055000,A0A0F8D555_2248_Halobacteria_A_Arc:0.00520000):0.03785000,(A0A2G1X713_1483399_Halobacteria_A_Arc:0.00524000,(V6DS43_1173487_Halobacteria_A_Arc:0.01088000,V6DRS8_1173487_Halobacteria_A_Arc:0.02669000):0.00523000):0.01081000):0.00781000):0.01788000):0.02731000):0.02105000):0.01586000):0.01982000):0.01306000):0.00651000):0.01992000,((W0K3U0_751944_Halobacteria_A_Arc:0.04780000,(A0A1G9XX20_996166_Halobacteria_A_Arc:0.16167000,M0CQV9_797114_Halobacteria_A_Arc:0.12572000):0.05760000):0.02209000,(A0A4P8WMJ7_88724_Halobacteria_A_Arc:0.05911000,(A0A3M9JL82_1853682_Halobacteria_A_Arc:0.01360000,(A0A3M9JV35_1853682_Halobacteria_A_Arc:0.02632000,U2YRD9_1261545_Halobacteria_A_Arc:0.03234000):0.06434000):0.06323000):0.01441000):0.00053000):0.02003000,(A0A1I0MG92_355548_Halobacteria_A_Arc:0.04171000,P09737_64091_Halobacteria_A_Arc:0.09407000):0.09220000):0.01895000):0.05424000):0.04719000):0.10862000):0.00515000,(((((A0A285N4Y7_558529_Halobacteria_A_Arc:0.06436000,(Q03302_272569_Halobacteria_A_Arc:0.06013000,(A0A4D6HF93_1457250_Halobacteria_A_Arc:0.05497000,M0CPB2_797114_Halobacteria_A_Arc:0.03725000):0.03181000):0.02409000):0.00744000,(A0A161XL90_1679489_Halobacteria_A_Arc:0.02310000,A0A1N6WYU5_553468_Halobacteria_A_Arc:0.03873000):0.02345000):0.01087000,((A0A1I6K766_767519_Halobacteria_A_Arc:0.04342000,A0A1I6LVZ5_767519_Halobacteria_A_Arc:0.03643000):0.04351000,(F7PIA8_1033806_Halobacteria_A_Arc:0.11773000,((A0A1G9VZ43_996166_Halobacteria_A_Arc:0.03938000,(D8J2G3_795797_Halobacteria_A_Arc:0.09756000,R4W031_1333523_Halobacteria_A_Arc:0.11095000):0.01382000):0.01377000,(((A0A3N6LM69_1679091_Halobacteria_A_Arc:0.01595000,((D3SY04_547559_Halobacteria_A_Arc:0.01462000,(A0A2Z2HUJ4_745377_Halobacteria_A_Arc:0.02173000,L0AKN7_797304_Halobacteria_A_Arc:0.07585000):0.01877000):0.02101000,A0A3N6M5P3_1679083_Halobacteria_A_Arc:0.00739000):0.01526000):0.00507000,(((M0L696_358396_Halobacteria_A_Arc:0.01240000,M0M5P3_1227454_Halobacteria_A_Arc:0.02601000):0.01538000,((A0A063ZJ32_1495067_Halobacteria_A_Arc:0.04794000,W0JR24_797299_Halobacteria_A_Arc:0.00619000):0.02527000,(M0CBS2_1230457_Halobacteria_A_Arc:0.05200000,(L0JLZ0_797303_Halobacteria_A_Arc:0.01336000,((A0A1H9BZT2_1186196_Halobacteria_A_Arc:0.00475000,(D2RVX0_543526_Halobacteria_A_Arc:0.01071000,((A0A1I0M6P7_1202768_Halobacteria_A_Arc:0.00055000,F8D6A5_797210_Halobacteria_A_Arc:0.06331000):0.00055000,A0A4P8WH38_88724_Halobacteria_A_Arc:0.02746000):0.00556000):0.02396000):0.01393000,(A0A2B7GK67_1608465_Halobacteria_A_Arc:0.01378000,L9ZI72_1227494_Halobacteria_A_Arc:0.02492000):0.01043000):0.00716000):0.02520000):0.02303000):0.00056000):0.01392000,((L0JX01_694430_Halobacteria_A_Arc:0.08877000,M0AWE7_29540_Halobacteria_A_Arc:0.09946000):0.05945000,((A0A1N7EDL3_588898_Halobacteria_A_Arc:0.01646000,L9WKK3_1227499_Halobacteria_A_Arc:0.02235000):0.00530000,(L9VRX1_1114856_Halobacteria_A_Arc:0.01098000,(L0IC00_797302_Halobacteria_A_Arc:0.04888000,L9WFH7_1230460_Halobacteria_A_Arc:0.02648000):0.00755000):0.00541000):0.00054000):0.00515000):0.00613000):0.02092000,A0A202E7D5_253108_Halobacteria_A_Arc:0.06420000):0.05044000):0.01068000):0.00619000):0.02348000):0.00503000,(A0A3P3RB00_671145_Halobacteria_A_Arc:0.14831000,(M0MM71_1227455_Halobacteria_A_Arc:0.02753000,(M0MDJ5_931277_Halobacteria_A_Arc:0.02437000,M0M2F6_1132509_Halobacteria_A_Arc:0.07177000):0.02472000):0.04731000):0.02165000):0.01658000,(C7NYW1_485914_Halobacteria_A_Arc:0.06626000,(((A0A1Q1FM06_1932360_Halobacteria_A_Arc:0.02081000,(A0A1U7EY38_348780_Halobacteria_A_Arc:0.03269000,M1XSP2_268739_Halobacteria_A_Arc:0.03953000):0.03068000):0.04889000,((A0A365TEZ3_29295_Halobacteria_A_Arc:0.09629000,(A0A1G8W4P7_890420_Halobacteria_A_Arc:0.11023000,R4W6K3_1333523_Halobacteria_A_Arc:0.08869000):0.01552000):0.01568000,(A0A2B7GPY0_1608465_Halobacteria_A_Arc:0.21300000,A0A2R4X0R1_1679096_Halobacteria_A_Arc:0.15886000):0.04170000):0.01133000):0.01825000,(U1MGY5_1325472_Halobacteria_A_Arc:0.11099000,U1QN78_1085028_Halobacteria_A_Arc:0.09604000):0.12160000):0.02382000):0.03083000):0.02014000):0.02068000,W0K3A4_751944_Halobacteria_A_Arc:0.05838000):0.02401000,A0A1H6J821_1267564_Halobacteria_A_Arc:0.07612000):0.05964000):0.33581000):0.19124000,(A0A0K1PI80_1391653_Proteobacteria_B_Mar:0.67053000,(A0A345UFZ3_1457365_Bacteroidetes_B_Mar:0.58744000,(A0A2Z4FGN2_1548548_Proteobacteria_B_Mar:0.48737000,(Q0AW95_335541_Firmicutes_B_Ter:0.38565000,((A0A0B7MKY0_499207_Firmicutes_B_Ter:0.24317000,A0A259UH52_1123289_Firmicutes_B_Ter:0.36078000):0.27002000,(A0A089IHE8_1536775_Firmicutes_B_Ter:0.23707000,(A8FE63_315750_Firmicutes_B_Ter:0.38178000,(A0A2T6C4K2_1242148_Firmicutes_B_Ter:0.14061000,Q67T03_292459_Firmicutes_B_Ter:0.28646000):0.04453000):0.03500000):0.14422000):0.08346000):0.09190000):0.07003000):0.08899000):0.09292000):0.04677000):0.17457000,x:0.17457000);
