# Supplementary material for: Characterization of ancestral Fe/Mn superoxide dismutases indicates their cambialistic origin
Source: Protein Sci. 2022 Sep 21;31(10):e4423. doi: 10.1002/pro.4423 (PMC9490801; doi:10.1002/pro.4423)
Supplement: Supplementary file 2 — Dataset S2 [file PRO-31-e4423-s011.zip › PRO_4423_Dataset2_SequenceAlignment.docx]

>A0A011NKF6_1454000_Proteobacteria_B_Mar

MEHKLPPLPFAIDALAPHMSQETLEYHHGKHHQAYVTNLNNLIKGS-EYETLDLEAIVRKAP------AGGIYNNSAQVWNHSFFWNCLRANGGGAPGGALAAAIDARWGSYAEFAKTFQASAVGNFGSGWTWLVRKPD-GTVDIVNMGAAGTPLTTADKALLCIDVWEHAYYIDYRNLRPKFVETFLNLVNWSFAEQNFAA---

>A0A011QCI9_1454004_Proteobacteria_B_Mar

MEHQLPQLPFAIDALAPHMSQETLEYHHGKHHQAYVTNLNNLIKGS-EYEALDLEAIVKKAP------AGGIYNNSAQVWNHTFFWNCLKPAGGGAPGGALAAAIDAKWGSFAEFAKAFQASAVGNFGSGWTWLVKKAD-GSVDIVNMGAAGTPLTTGDTALLCIDVWEHAYYVDYRNQRPKFVETFLQLANWTFAEKNFAG---

>A0A031IK26_1470592_Firmicutes_B_Ter

SKFELPELGYAYDALEPHIDARTMEIHHTKHHNTYVTNVNTALEG-TEHEGKSLEELLQNLDALPANIQTAVRNNGGGHWNHSFFWKLLKKNDGAAPTGELATAIDEAFGSFDAFKDAFAKAATTRFGSGWAWLIVD-G-GKLAVVSTPNQDTPVMEGKTPILGLDVWEHAYYLNYQNRRPDYINAFFNVVDWDHVAKLYADAK-

>A0A061Q889_1492281_Proteobacteria_B_Mar

--FELPKLPYEKNALEPHMSAQTLDFHHGKHHAKYVDVANDLVKGT-AHEGQSLEQVMVESFG----KNDKLFNNVGQIYNHNEFWKCMKPNGGGAVPEALKAKLVEAFGSVEAFAEEFVAAGTGQFGSGWVWLVVK-D-GKLAIMKTANAENPVVHGATPLLVADVWEHAYYLDYQNRRPDFLKTFIKLVNWDYVNERLSKA--

>A0A062XM00_1312852_Acidobacteria_B_Mar

SVHQLPPLPYAYDALEPYIDTQTMQIHHTKHHQAYVDNLNKAVTG-SPVESWSLEELIAKLSQVPEAIRTAVRNHGGGHLNHSLFWQMMAPNAGGTPQGELAQAIANAFGSFESFKEKFTAAAMGRFGSGWAWLVVKPS-GALDVYSTANQDSPLTDGDRPILGVDVWEHAYYLKYQNRRNEYVQAWWNVVNWNFVSELLRKARS

>A0A062XUH1_1312852_Acidobacteria_B_Mar

LSLELPPLPYAYDALEPYIDAETMRLHHDKHHAAYIAGLAKAVEQVPKLAEQTLEELLTSLAKVPEAVRTAVRNHGGGHYNHSLFWQCLAPNAPAAPEGELARAMARDFGSFESFKETFSKQAMGVFGSGWVWLVVE-G-GKLQVATTANQDTPLS-GKKPLLGLDLWEHAYYLKYQWRRAEYVAAFWHVVNWPFVARRFTQIAH

>A0A063ZJ32_1495067_Halobacteria_A_Arc

TDHELPPLPYDYDALEPALSEQVLTWHHDTHHQGYVNGLNSAEETLAENRENGDF---DSTP----GALSNVTHNGCGHYLHTLFWENMAPDGGGEPSGELADRIEEDFGSYEAWKGEFEAAAG--AAGGWALLVYDPVSKQLRNIAVDKHDQGALWGSHPILALDVWEHSYYYDYGPDRGSFIDSFFDVVNWDKAAEEYQTCLD

>A0A073CMZ0_388467_Cyanobacteria_B_Ter

MAFELPALPFADDALESSMSAKTFSFHHGKHHAAYVTNLNKLIDGT-GLADKSLEEIIKATAN--DPNKAGIFNNAAQVWNHTFFWNSLKAGGGGQPTGALADKINADFGSFDKFIEEFKAAATTQFGSGWAWLVLN-N-GTLKVTKTANAGTPIVDGLTPLLTLDVWEHAYYLDFQNARPAYIENFLQLVNWDFVAENFAAA--

>A0A077FIP1_1528098_Proteobacteria_B_Mar

MTFVLPNLPYNNNALEPYITFNTLNFHHGKHHNTYVTNLNNLIKDS-ELSSKSLEEIILSTAK--DTSKSGIFNNAAQVWNHTFYWHSMKASGGGMPSPTLLDKINEDFGSFDKFKEEFKNAGTTQFGSGWSWLVLE-G-NQLKITKTGNADLPLIHNQVALLTCDVWEHAYYLDYQNRRPDYITTFLKLVNWEFVEENFLNSQK

>A0A081EVB3_2248_Halobacteria_A_Arc

MSYELDPLPYDYDALEPHLSEQVLEWHHDTHHQGYVNGWNAAEETLEENRESGDF---SSSG----GALRNVTHNSSGHILHDLFWQNMSPEGGDEPEGALAEQIEEDFGSYEAWKGEFEAAAS--AAGGWALLVYDSFSNQLRNVVVDKHDQGAIWGGHPILALDVWEHSYYHDYGPARGEFVDNFFEVVDWDEPSSRYEQAVE

>A0A085G4I0_910964_Proteobacteria_B_Mar

MSYSLPSLPYAYDALEPHFDKQTMEIHHTKHHQTYVNNANAALESLPELAKLSVEELIANLDKVPADKKVALRNNAGGHANHSLFWKGLKT--GTTLQGDLKAAIERDFGSVDAFKETFEKAATTRFGSGWAWLVLKTD-GKLAVVSTANQDSPLMGSGYPLLGLDVWEHAYYLKYQNKRPDYIKAFWDVVNWDEAAKRLAEAK-

>A0A085HE47_1005999_Proteobacteria_B_Mar

MSYTLPSLPYAYDALEPHFDKQTMEIHHSKHHQAYVNNANAALESLPELKNLSAEELIAQLDKVPAEKRTALRNNAGGHANHSLFWKGLKK--GTQLTSELKAAIERDFGSVDAFKEKFEQAAATRFGSGWAWLVYK-D-GKLAVVSTANQDNPLMGSGYPIVGLDVWEHAYYLKYQNRRPDYIKAFWDVVNWDEAAKRFADVKK

>A0A085JMH6_1005995_Proteobacteria_B_Mar

MSYSLPSLPYAYDALEPHFDKETMEIHHTKHHQAYVNNANAALEG-TEFASLPVEELITKLDQVPADKKTALRNNAGGHANHSLFWKGLKT--GTTLQGELKAAIEKDFGSVEKFQAEFEKAAATRFGSGWAWLVKQGD--KLAVVSTANQDSPLMGSGFPILGLDVWEHAYYLKFQNKRPDYIKAFWNVVNWDEAAARFASAK-

>A0A085L1P1_1453500_Bacteroidetes_B_Mar

MAFTLPSLPYDKAALEPHIDAMTMEIHHGKHHAAYVNNLNAAIQG-TEYENKSIEELMSMVSKLS----VAVRNNGGGHYNHTLFWTIMAPNGGGTPSGELAAAIDSQFGSFDKMKEDFNKAAATRFGSGWAWLCVGAD-KKLFITSTPNQDNPLMDKGTPILGLDVWEHAYYLKYQNRRPEYIAAFWNVVNWPEVARRYQEAVK

>A0A086D0E9_1492922_Proteobacteria_B_Mar

MAHSLPSLPYAYDALEPKIDAMTMEIHHSRHHQTYVNNLNAALEG-TGLEDVPVDKLMADIDKVPQDKRQAVINNGGGHANHSLFWTVMSKDGGGAPTGKVAEAIDSELGGYANFQEEFKKAATGRFGSGWAWLVVTPE-KKLKVINTLNQDSPLMDGLTPILGLDVWEHAYYLKYQNKRPDYIAAFFDVINWDEVNRRYAEATA

>A0A089IEX7_1536775_Firmicutes_B_Ter

MAFQLPALPYANNALEPHIDALTMEIHHDRHHNTYVTNLNAALEKAPELQSKSIEELLTDLNAVPEAIRTAVRNNGGGHANHTLFWEVIGPNGGGAPTGALAAAIDSELGGFEKFKEDFAAAATTRFGSGWAWLVVK-D-GKLAVTSTPNQDNPISEGATPILGLDVWEHAYYLNYQNKRPDYIKAFWNVVNWEEVGKRYESAK-

>A0A089IHE8_1536775_Firmicutes_B_Ter

GGHTLPPLPYAYNALEPYIDEKTMRIHHDKHHQSYVDGLNKAEVKLADSRKNGDF---DLVK----HWERELAFNGAGHYLHTIFWNVMSPQGGGRPTGALLEAIEQSFGSYDSFKKQFTEAANKVEGGGWAILVWSPRSHRLEILTAEKHQNLSQWDVVPLLALDVWEHAYYLKHQNNRADYINDWWKVVNWPYVSERYAAASK

>A0A090CZB7_1437425_Chlamydiae_B_Mar

LSFLL-FSDYQAAYLIGNLDDELLKMHF-KLYQGYVLNSNNLLKKLQALNESGQN-KTPEFA----GLKRMLGWEFDGMLLHEYYFDNLGKSEN-PKDSPLLKKIESDFGSFDNWKKDFVATGA-MRGIGWVVAYIDPKEGRLINQWINEHDVGHLAGGKPLLVMDVFEHAYITQFGLDRAKYIEVFFDNINWKKVNERLTTAKS

>A0A090FD11_1505946_Proteobacteria_B_Mar

MAFELPALPYDYEALQPYMSKETLEYHHDKHHKAYVDNGNKLAAEA-GMGDLSVEEVVKQSFG----KNAGLFNNAAQHYNHIHFWKWMKKGGGGKLPGALQKAFDSDLGGYDKFKADFTAAGTTQFGSGWAWVSVK-N-GKLEISKTPNGENPLVHGGSPILGVDVWEHSYYIDYRNARPKYLEAFVSLINWDYVLELYEKAKG

>A0A095XIK9_1230730_Firmicutes_B_Ter

MAFTLPELPYAYDALEPVIDKETMQFHHDKHHDTYVNNLNKALEKAPEWAAKDIEEIVADWDNAPEEVRQAIRNNGGGHYNHSLFWQMMTPVKDSKLEGAIQAQIEKDFGSFDAFKKEFGAAATGRFGSGWAWLVWN-G-KKLAVVSTPNQDNPLADGQKPLLGIDVWEHAYYLKYQNKRADYVDNFFKVINWKFVNELFAAAKK

>A0A097R7C0_1453496_Proteobacteria_B_Mar

MSYTLPSLPYAYDALEPHFDKQTMEIHHSKHHQAYVNNANAALESLPELAKLTAEELIAQLDKVPAEKRTALRNNAGGHANHSFFWKGLKL--GTELKGDLKAAIERDFGSVDKFKEEFEKAAATRFGSGWAWLVLKAD-GKLAVVSTANQDSPLMGSGYPIAGLDVWEHAYYLKYQNRRPDYIKAFWSVVNWDEAAKRFTEAKK

>A0A098SDX5_1524460_Bacteroidetes_B_Mar

MAFELPKLPYAYDALEPHIDARTMEIHHTKHHAGYTSKLNAAIEG-TDLEKKSIKEILAHVSKHS----AGVRNNGGGFYNHKLFWEVMSPDGGGEPSERIHKAIMRDFGSFEKFKDEFANAAATQFGSGWAWLCVDRD-DKLFVTSTPNQDNPLMDDGTPILGLDVWEHAYYLNYQNRRPDYIEAFFNVIDWGKVSENYNDANE

>A0A099W947_1552123_Firmicutes_B_Ter

MTYELPKLAYTYDALEPNFDKETMEIHYSKHHNTYVTKLNEALEGHDDLAAKSIEDLVADLNSVPENIRTAVRNNGGGHANHSFFWTILSPQGGGEPTGALADAINAEFGSFEEFKTQFANSGVARFGSGWAWLVVK-D-GKLSIISTPNQDSPLTEGYTPVIGLDVWEHAYYLKFQNLRPKYIETFWNVVDWDKAAEYFNAAK-

>A0A0A2F0U5_1515615_Bacteroidetes_B_Mar

MKHELVKLPYGPNDLEPAISAETLAFHHGKHLQGYVDNLNKLIEGT-EFAEASLEDIVRKS-------EGALFNNAGQTLNHNLYFTQFAPNAGGAPTGKLLAAIEKKWGSFEAFQAEFLTACTTLFGSGWAWLASDDK-GELCITKEPNGSNPVVSGLKPLMGIDVWEHAYYLSYQNRRADHIKDVWNIIDWNVVAERY-----

>A0A0A2F1H1_1515615_Bacteroidetes_B_Mar

HRFELMKLPYATDALEPVISKATVELHHGKHLQGYVNNLNRLKAGT-VFEHVTLVSIVKQS-------KGGIFDNAGQLLNHNLYFAQFSPKPQ-ALSGALAEAIKKQWGSEAEFRAAFEREGMTLFGSGWLWLASDKA-GTLSIVKEANGSNPVVRDLTPLLGVDLWEHAYYLDYQNKRADHLKALWRIIDWQVVGQRYDQR--

>A0A0A6PC03_1003181_Proteobacteria_B_Mar

M-HTLPELPYAKNALEPHISAETLEYHYGKHHQTYVTNLNNLIKGT-EFENLSLEETIRKA-------SGGLFNNAAQIWNHTFYWNCLSPQGGGEPSGALADAIKRDFGSFESFKENFSKTAITTFGSGWGWLVKKPD-GTLALESTSNAATPLTGENKPLLTCDVWEHAYYIDYRNARPKYVEAFWNLVNWDFVAENFAA---

>A0A0B0HBS3_2340_Proteobacteria_B_Mar

MQHQLPELPYEKSALEPHISAETLEFHHDKHHATYVTNLNNLIEGT-EFADKSLEEIVTSAP------AGGIFNNAAQVWNHTFYFNCMAPEAGGAPSGEIAAAIDAAFGSFEDFKTAFSTSAATNFGSGWTWLVKNAD-GGVEIFNTSNAGCPLTEGLTPLLTIDVWEHAYYVDKRNARPAYVESWWNLVNWEFVNAQL-----

>A0A0B1Q2K7_370622_Proteobacteria_B_Mar

GPFTLGDLPYAADALDPVIDAETMTLHHDKHHRAYVDNLNKAVAEDEALQGVTLEDFVANAGTYP----AAVRNNAGGHWNHTFFWESMAPDAAGEPSEELAAAIEAVYGDMEAFKTAFNQAGAGRFGSGWVWLIVNDA-NELEITTTPNQDNPLMDKGTPIIGNDVWEHAYYLKYNNRRPEYLSAWWGVVNWDVISQRYDAALA

>A0A0B1Q8Z2_370622_Proteobacteria_B_Mar

MAFTLPELPYAYDALGPYMSKETLEFHHDKHHNAYVEMGNKLAAEA-GLGDASVEEVVKQSFG----KNQPLFNNAAQHYNHIHFWKWMKPNGGGSLPGKLQTAFDSDLGGYDKFRTDFIEAGKGQFGSGWAWVSVK-D-GKLEISKTPNGENPLVHGGTPILGVDVWEHSYYIDYRNLRPKYLEAFVNLINWDYVLERYEAAAR

>A0A0B4XLG0_391936_Proteobacteria_B_Mar

MAFELPALPYAKDALAPHMSAETLEFHYGKHHQTYVTKLNELVAGT-DKEGQSLEDIIKGAG------PGPLFNNAAQVWNHTFFWNSLSPNGGGEPTGALADAINAKWGSFDKFKEAFTNSAIGNFGSGWTWLVKNGN--ELEIVNTSNAATPLTDGKTALLTVDVWEHAYYIDYRNARPKYLEGFWALVNWEFAASNFA----

>A0A0B6WW40_454194_Acidobacteria_B_Mar

MAHELPPLPYPYDALEPYIDAQTMQLHHDKHHAAYVNNLNAALEKYPELQNKSAEDLIKDLNSVPEEIRTAVRNNGGGHVNHTMFWQIMKPNGGGEPTGPIAEAIKRTFGSFAEFKKQFNDAGMKRFGSGWVWLIRSKD-GQLQITSTPNQDNPLMEGHYPIMGNDVWEHAYYLKYRNRRNEYLEAWWNVVNWDEINRRFEQAR-

>A0A0B6WWE2_454194_Acidobacteria_B_Mar

GPHKLPPLPYEYNALEPYIDTETMQLHHDKHHAAYVNNLNAAIAKHPELSNRSAEDLIRNLQSVPADIREQVRNNGGGHVNHTMFWQIMKPNGGGEPTGPIAEAIKRTFGSFAEFKKQFNDAGMKRFGSGWVWLVRDRA-GRLQIISTPNQDSPLTQGLYPIMGNDVWEHAYYLKYQNRRNEYLEAWWNVVNWDEINRRFETATS

>A0A0B7MKY0_499207_Firmicutes_B_Ter

GGHKLPPLPYPYNALEPVISKRTLRIHHDRHHLAYVEGLNKAELRLVEARRMGDF---SLVK----HWERELAFHGSGHILHSIYWTVMAPGGLREPGYYTCLEIEKYFGGFAPFKEQFTEASIQVEASGWGVLCWVPSWMRLEILTAEKHQNLTQWGAIPLLVVDAWEHAYYLDYQNRRADYVRAWWSLVNWNEVERRLRLAMR

>A0A0C1MX08_1479485_Cyanobacteria_B_Ter

MAFVQEPLPYDFNALEPYMKGETFEYHYGKHHKAYVDNLNKLTDGT-ELADKSLEEVIKISFK--DSSKAGIFNNAAQVWNHTFFWNSLKPGAGGKPQGELASKIEKDFGSFDKFTEEFSNAAATQFGSGWAWLIDD-G-GTLKVTKTPNAENPLAHGQKALLTLDVWEHAYYIDFRNARPAYIKNFLQLVNWDFAAENYAKAY-

>A0A0C1QR13_1479485_Cyanobacteria_B_Ter

GAIQLPPLPYSYNALEPHIDAKTMQFHHDKHHATYVKNLNAALNKHPELKGKSVEALLRNLNNLPEDIRTTVRNNGGGHANHAMFWRIMKPKGGGEPTGAIASAIKESFGSFASFKKQFNEAGVGRFGSGWVWLVRTKD-GNLAVMTTPNQDSPIVEGNYPIMGNDIWEHAYYLKYQNRRADYLKAWWNVLNWDEINKRFVASST

>A0A0C1QWB0_86105_Proteobacteria_B_Mar

NNFTLPQLPYAKNALEPYVSEETLSFHHGKHHQTYVTNLNNLIKDS-PFAEMGLEDIIEKSYQ--KADKVGIFNNAAQVWNHAFLWHCMKKDGGGLPSKTLQDKINEDFGSMEKFVEEFKNAAITQFGSGWAWLVLD-K-DKLKVIKTGNAELPMVLGQKALLTIDVWEHAYYLDYQNRRADYVNTFIKLINWQFVEENFNSR--

>A0A0C1R8Q1_1479485_Cyanobacteria_B_Ter

TPAQLPPLPYAYDALEKVIDEETMKLHHDKHHAAYVNNLNDALKKHPELQSKSVESLLRDLNGVPEDIRNTVRNNGGGHLNHTLFWQLMSPQGGGEPTGAIATEINQTFGSFDAFKKQFNEAGTKQFGSGWVWLVRNPQ-GQLQIVSTPNQDNPIAEGSYPIIGNDVWEHAYYLKYRNRRAEYLEKWWNVVNWSEVNKRAQVTSQ

>A0A0C2QFL4_1233231_Cyanobacteria_B_Ter

MAFELPPLPYDYDALSPLISSDTLKFHHDKHHAGYVTNLNKLIEGT-ELANKSLEEIVLATAN--DSSKTGIFNNAAQVWNHTFYWNGLKK-GAGAPSGELAEKINASFGSLDEFKKQFKEAGATQFGSGYAWLVLD-N-GELKVVKTPNAANPITNGQTPLLTADVWEHAYYLDYQNRRPDYLDTFLELINWDFVAEQYANAAK

>A0A0C2QL44_1233231_Cyanobacteria_B_Ter

GAIQLPALPYAYEALEPHIDAGTMRFHHDKHHAAYVKNLNTALDKHPELKSRTVEQLLRNLNSVPEDIRKTVRNNGGGHVNHSMFWRIMKPKGGGEPRGPIADAIKQNFGNFATFKNQFNEAGAGQFGSGWVWLVRTKD-GKLAVATTANQDSPLSEGNYPIMGNDVWEHAYYLKYQNRRADYLSAWWNVLNWDEINRRFVEGGK

>A0A0C7NIR4_1006576_Thermotogae_B_Anc

MRFELPKLNYSYDSLEPYIDSRTMEIHYTKHHATYVNKLNEALEKHPELMNKNLEDLLKDLDNIPSDIRTTVRNNGGGHYNHTLFWSIMGPNGSRKPIGKLAQRIDEVFGSFDKFKLEFSDSALNRFGSGWAWLVLDKY-GHLSIISTPNQDNPIMIGLYPILGIDVWEHAYYLKYQNRRAEYIESWWNVVNWEEVEKNYLNIV-

>A0A0C9Q165_1197129_Planctomycetes_B_Mar

AAFGIPTMSYNASKLIG-FSDTLLKNHF-TLYQGYVTNTNKVLDTLSQMLKEGKT-ANPEFA----ELKRRLGWEFNGMRLHEYYFENLGGKAGINKEGKLAKKMADAFGDYATWEKDFRAVGA-MRGIGWAVLYQDSANGQLINFWVNEHDVSHPAGCNPILIMDVFEHAFMIDYGLKRADYIEAFFKNIDWSAAEARLK----

>A0A0D0S5X1_1199154_Proteobacteria_B_Mar

MAIELPPLPYDRTALEPHISGETIDFHYGKHHKSYVDNLNKMIEGT-EFADMPLEDIIRKS-------QGGMFNNAAQVWNHTFYWNCLSPNGGGEPTGKLAEAISKSFGDFATFKEQFSDTAVKTFGSGWGWLVQRTD-GSLALVSTSNANTPLTGSDTPLLTCDVWEHAYYIDYRNARPKYVETFWKLVNWDFVASNLK----

>A0A0D3LFT2_1257021_Bacteroidetes_B_Mar

MAFELPNLPYPSNALEPNIDQQTMEIHHGRHHKGYTDNLNKAIQG-TDMENKSIEEILKNVSGN-----NAVRNNGGGYYNHNLFWEVMSPNGGGQPTGAVAEAINKKFGSFDKFKEEFSNAAATRFGSGWAWLIVTGN-GEVDVTSTPNQDNPLMDKGTPILGLDVWEHAYYLKYQNKRVDYVGNWWNVVNWEAVNRRFEQARN

>A0A0D8ZQ77_1618023_Cyanobacteria_B_Ter

MAFELSPLPYNYDALDPYIDSQTMQLHHDMHHQAYVNNLNAAVDKHPELQSKSPEDLVRELNSLPQDIVSAVRNNGGGHVNHTMFWQIMAPNAGGEPTGAIADAIKDNFGDFESFKTRFNDTGTKQFGSGWVWLVRTSD-GKLEVVGTPNQDSPITGGHFPIMGNDVWEHAYYLKYQNRRAEYLKQWWNVVNWDEINNRLSMSTR

>A0A0D8ZRD9_1618023_Cyanobacteria_B_Ter

GKITLPPLPYDYKALEPHIDARTMRFHHDNHHATYVKNLNAALAKYPNLQSKSVEQLLREIQSVPEDIRTTVRNNGGGHENHSMFWRIMKPKGGGEPTGAIASAIKADFGSFATFKQQFNEAGSKRFGSGWVWLIRNPA-GKLQITSTPNQDSPLMEGNYPIMGNDVWEHAYYLKYQNRRAEYLTAWWNVINWDEVNRRLAQATK

>A0A0E3NRG6_1434102_Methanomicrobia_A_Mar

ELYKLPPLKYGYADLAPYISEEQLRIHHDKHHQGYVNNTNALLEMMDKARKEGTD---FDYK----TTAKALSFNLGGHVLHDYFWWEMTPAASKEPVGELADVIKDDFGSFERFKKEFSQVASSVEGSGWAALTFCNDTKRLGIMQIEKHNVNLVPDFPILMDLDVWEHAYYIDYKNDRSKFIEGFWNIVDWEEIDKYFKKTQK

>A0A0E3NWV4_1434100_Methanomicrobia_A_Mar

ELYKLPPLKFGYADLEPYISEEQLRIHHDKHHQGYVNNTNSILQMMEKARKDGTD---FDYK----ATAKALAFNMGGHVLHSYFWWELTPAASKEPVGELAEAIKEDFGTFERFKKEFSQVAASVEGSGWAALTFCRDTKRLGFMQIEKHNVNLIPDFPVLLVVDVWEHAYYLDYKNERGKFIDAFWNIIDWEEIDKYFKTIR-

>A0A0E3WWB9_1434107_Methanomicrobia_A_Mar

GLYKLPDLKYGYSDLEPYISEEQLRIHHDKHHQAYVNNANSLIEMMDKARKEGTD---FDYK----ATAKALSFNLAGHVLHDYFWWEMTPESSKEPVGELSEVIKEDFGGFDRFKKEFSQVASSVEGSGWAALTYCKDTERLMIMQIEKHNVNLVPDYPIIMDLDTWEHAYYIDYRNDRAKFIEAFWNIVDWEEIDKYFKKMRK

>A0A0F5IRT0_1203610_Bacteroidetes_B_Mar

MKFELIQLPYAANALEPVISKETIEFHHGKHLQAYVNNLNNLIPGT-KFENAPLEQIVAES-------DGAIFNNAGQILNHNLYFTQFSPKGGGKPSGKLAEAIDKTWGSFENFQKEFNTAGAGLFGSGWVWLAKDKD-GKLSITKEANGSNPVAKGLTPILGFDVWEHAYYLDYQNRRPDHLAALWKIVDWDAVGKRY-----

>A0A0F5VGV2_265726_Proteobacteria_B_Mar

MAFELPALPYEKNALEPHISQETLEYHHGKHHNTYVVKLNGLIEGT-EYADKSLEEIIKTS-------TGGVFNNAAQIWNHTFYWHCLSPNGGGEPTGAVADAIVKAFGSFDAFKAKFTDSAINNFGSSWTWLVKKAD-GSLDIVNTSNAGTPLTEGVTPLLTVDLWEHAYYIDYRNLRPDYMNGFWALVNWEFVAKNLAA---

>A0A0F5VHM9_265726_Proteobacteria_B_Mar

MSFTFPALPYPYDALEPYIDAKTMEVHHSRHHKTYFDKFISAIEN-TGLEQLTLKDIFSRVSEL----SPAVRNHGGGYYNHNLYWQCMSPNGGGQPVGLLAEAIRCHFGSFDDFKIAFSDAAAAHFGSGFIWLSVT-D-GRLEITSTPNQDNPLMDRGTPILALDVWEHAYYLTYQNKRPDYISAWWQVVDWDQVEQHYLDALH

>A0A0F6YK52_927083_Proteobacteria_B_Mar

MAFTLPELPYAKDALAPHISAETLEFHHGKHHATYVTNLNKFVDDP-SLQGKSLEDIIRTS-------KGPVFNNAAQVWNHTFYWHSMRPGGGGEPTGAIKGAIDKSFGSFATFKEQFSAKAVGHFASGWAWLVKNGD--KLEIVDTHDAGTPLTMDRKPILTCDVWEHAYYVDYRNARAKYVESWWNLVNWDHANSQL-----

>A0A0F7K213_1543721_Proteobacteria_B_Mar

MAHELPPLPYAQDALEPVISKETLEYHYGKHHQTYVTNLNNLVKGT-DLENASLEEIIMKA-------SGGQFNNAAQVWNHTFYWNCLCP-GGSAPSSQLSDAINSAFGSMDEFKKQFSASAAGNFGSGWTWLVKNSD-GGLEIVNTSNAANPMTDGKTPLLTIDVWEHAYYIDYRNARPKYLEAVWNIVNWEYVSANFAG---

>A0A0F7PDN8_1604004_Halobacteria_A_Arc

SDYELPPLPYDYDALEPHISEQVLTWHHDTHHQGYVNGWNNAEETLAENREAGTT---DDSA----AAIRNVTHNGSGHILHTLFWNNMSPNGGGEPEGDLRARIEEDFGSFEAWQDEFEAAAG--AASGWALLIYDTHAERLRNVVVDKHDQGALWGSHPILALDVWEHSYYFDYGPDRGSFIDAFFEVVDWDDVAENYEDVIA

>A0A0F8D555_2248_Halobacteria_A_Arc

MSYELDPLPYEYDALEPHLSEQVLEWHHDTHHQGYVNGWNAAEETLEENRESGDF---SSSG----GALRNVTHNSSGHILHDLFWQNMSPEGGDEPEGALAEQIEEDFGSYEAWKGEFEAAAS--AAGGWALLVYDSFSNQLRNVVVDKHDQGAIWGGHPILALDVWEHSYYHDYGPARGEFVDNFFEVVDWDEPSSRYEQAVE

>A0A0H1RD07_1225564_Proteobacteria_B_Mar

MSFTLPELPYAYDALQPYMSRETLEFHHDKHHAAYVNTGNNLLKGT-EFEGKSVEEVVKGSYG----KNQALFNNAGQHYNHIHFWMWMKPNGGGALPGKLEKKIVEDLGSVDKMKEDFIQAGVSQFGSGWAWLAVK-D-GKITVMKTPNGENPLVHGAQPILGCDVWEHSYYIDYRNRRPDYIKAFLNLVNWEHVEEMFESATK

>A0A0H1RDD0_1225564_Proteobacteria_B_Mar

GPLKLDPLPYAPSRNEPHIDAQTMELHHGKHHAAYVNNLNAALSRNGDLAKMPLHDMLAKLGDIPESVRTAIRNNGGGHANHTMFWQIMGGS-GGEPSGELKSAIDRDLGGFQKFQADFNTAGEKQFGSGWVFVTVSRD-GRLAIIAKPNQDTPLMDGQRVLMGNDVWEHAYYLKYQNRRPDYLKAWWNVLDWNRIGERYASAKA

>A0A0H4WNU9_1297742_Proteobacteria_B_Mar

MPFTLPELPYKKDALQPHMSAETLEFHHDKHHAAYVNNLNKLLDGK-AEANKSLEEIILSS-------DGGVFNNAAQVWNHTFFWNCMKPAGGGKPTGELAAAIDRDFGSFDKFKEEFSTAAATQFGSGWAWLVLE-G-GKLKVTKTGNADLPMKHGQKALLTIDVWEHAYYIDYRNLRPKFIETFLHLVNWDFVAQNLKG---

>A0A0J8VRJ1_435910_Proteobacteria_B_Mar

MSYTLPSLPYAYDALEPHFDKQTMEIHHTKHHQTYVNNANAALESLPEFANLPVEELITKLDQVPADKKTVLRNNAGGHANHSLFWKGLKK--GTTLQGDLKAAIERDFGSVEKFKEEFEKAAATRFGSGWAWLVKQGD--KLAVVSTANQDSPLMGSGYPILGLDVWEHAYYLKFQNRRPDYIKEFWNVVNWDEAAERFASQK-

>A0A0K0GDK0_1637974_Firmicutes_B_Ter

AKFELPELPYAYDALEPTIDKETMNIHHTKHHNTYVTNLNAAVEGISELEGKSVEELISNLDAVPEAKRTAVRNNGGGHANHSLFWKVLSPNGGGEPTGELAEKINAKFGSFDKFKEEFENAAKTRFGSGWAWLVSN-N-GELEVMSTPNQDSPLMEGKTPLLGVDVWEHAYYLKYQNRRPEYLAAFWNVVNWDEVAKNYKG---

>A0A0K0XVJ8_1579979_Proteobacteria_B_Mar

MAFTLPELPYAKDALEPHISAETLEYHYGKHHQTYVDKLNGLVEGT-ADADKSLEDIIRSS-------SGGLFNNAAQVWNHTFYWHCLAPNGGGEPGGALAELIDRDFGSFAAFKEQFTQTAINTFGSGWAWLVQNPD-GKLAITSTSNAETPLTGSAKPLLTCDVWEHAYYIDYRNARPKYMDAFWNLVNWDHVAAQLS----

>A0A0K1P8N3_1391653_Proteobacteria_B_Mar

--------KYEANHLLGKLSEAQLKAHF-GLYQGYVNKLNEIWEKLGTVDKSKANYSFNEYS----ELKRREPVAYNGTFLHELYFSNLGHEGAVSP--DLKKAIEASFGSFDNWMIDAKACLA--SAHGWTLTVYDWNLNRVSNMLVQSHHVGLWPNVSVLCAVDAWEHAYMIDYGTKKPDYVNNVMNALNWNAINERFAHINP

>A0A0K1PI80_1391653_Proteobacteria_B_Mar

KEYTLPPLPYAYEALDGYLSAEILHLHHDVHHAAYVKGLNTAAAGLADARKKGDF---AQIK----ALERAMEFNGSGHVLHSLYWNSMSPQGGGQPTGVLKTAIEASFGSVDAFRGQFAAAAKAAEASAWGVLAYEPLGDRLVVVAAENHQNMGFQGVQPLLCCDVWEHAYYLRYKADRASYVDRFFDVINWGSAEQRLRAVRP

>A0A0K1PVI4_1391654_Proteobacteria_B_Mar

MAFTLPNLPYAKDALAPHMSAETLEYHHGKHHAAYVTNLNKLLDGK-PEANKSLEEIIMSS-------DGGVFNNAAQVWNHTFFWSCMKPNGGGQPTGDLAAAITRDFGSFDKFKEEFSTAAATQFGSGWAWLVLD-G-GKLSIMKTANADLPMKHGKKALLTIDVWEHAYYIDYRNLRPKFIETFLSLVNWDFVSENLKKA--

>A0A0K1PWM2_1391654_Proteobacteria_B_Mar

MTFELPKLPYAKDALAPHVSAETLEYHYGKHHAAYVTNLNKALEGK-PEAAKSLEEIILSS-------EGPVFNNAAQVWNHTFYWNSMKPNGGGEPTGDLLAAIQRDFGSVAKLKEELTNAATTQFGSGWAWLVLD-G-GKLAVTKTGNADLPMKHGQKALLTLDVWEHAYYIDYRNARPKYIETFLHLVNWDFALANLKNA--

>A0A0K2SFV8_1555112_Firmicutes_B_Ter

MAHELPPLPYGFDALEPYIDAQTMQIHHDKHHGTYVNNLNAALESADALRALGVEQLLQRINEVPESIRTAVRNNGGGHANHSMFWEIMAPNAGGAPSGELGSAITSAFGSFDSFKETFAKAALGRFGSGWAWLVLK-G-GRLEVSSTANQDSPYMEGQVPILGLDVWEHAYYLKYQNRRPEYVQAWWNVVNWPEVNQRFLKARS

>A0A0K6IWK3_876478_Proteobacteria_B_Mar

MEHKLPELPYPKNALAPYISEETMEYHYGKHHQAYVTNLNNLIKGT-EYENMDLESIVKKAP------AGPIYNNAAQTWNHTFFWHCMKPNGGGEPKGALLDAIKAKWGSFDEFKKVFHTSAVGNFGSGWTWLVKKAD-GSVDVVNMGAAGTPLTTGDTPLLCIDVWEHAYYIDYRNRRADFVTAFLHLVNWEFAERNYAA---

>A0A0L8V2C5_1409788_Bacteroidetes_B_Mar

MSFELPKLSYAYDALEPLIDARTMEIHHSKHHAAYTSKLNDAVNG-TELEGKSIEELLANVSKHS----TAVRNNGGGFYNHNLYWEIMAPGGATQPEGDLLKAITDSLGSVDKFKDAFANAAATRFGSGWAWLVKQ-G-DSLVVSSTPNQDNPLMDKGTPILGIDVWEHAYYLKYQNKRPDYIEAFWKVINWDEVAKRFKG---

>A0A0L8V2C9_1409788_Bacteroidetes_B_Mar

MSFELPALPYAKNALEPYISEKTLDFHYGKHHQAYVNNLNNLIQGT-KFETADLETIVKES-------DGGIFNNGAQVWNHTFYFMQFAADGCKEPKDELKKAIEAEFGSVESFKDAFSKAAATLFGSGWAWLVKDAA-GKLSIVQTSNAANPMRDGLTPLMTCDVWEHAYYLDKQNARPKYIEDFWKVLDWKVVSERF-----

>A0A0L8V3P2_1409788_Bacteroidetes_B_Mar

EGHKFPDLPYGYDALEPYVDAQTMELHYDKHHRGYFGKFTDAIAG-TDLEKTPMPEIFAKIDQQS----DGVRNNGGGFYNHQLFWENMTPEQTAM-NEELKAAIEADFGSVDALKEEFGQAAKTQFGSGWAWLSVDAN-GKLFVSSTPNQDNPLMNQGTPILALDVWEHAYYLHYQNRRADYVDNFWNIVNWEVVGMRYQNA--

>A0A0M2Q238_317619_Cyanobacteria_B_Ter

MAYELPALPYAYTALEPSITQATLEFHHDKHHAAYVNNYNTAVAGT-EYDSMALEAVIKAVAG--DASKAGLFNNAAQAWNHSFYWLCMKSGGGGAPTGALADKITADFGSFEEFVTAFKTAGATQFGSGWAWLVLD-N-GTLKVTKTGNAENPMTSGQVPLLTMDVWEHAYYLDYQNRRPDYMTDFLKLVDWDFVAANLAAA--

>A0A0M2R958_1549748_Proteobacteria_B_Mar

MALELPALPYAYDALGPYMSAETLEFHHDKHHNAYVVNGNKLLEGS-DLEGKSLEDIVKGSFG--DASKAGIFNNAAQHWNHIEFWNMMKPNGGGAIPGELEKKIIEDFGSVDQFKADFVNAGVTQFGSGWCWLVLDDS-GKLAVTKTANGENPLVTGGHALLGCDVWEHSYYIDYRNARPDYVKAFVSLVNWEYVAERFSKAG-

>A0A0M4CZS5_1603606_Proteobacteria_B_Mar

MTIALPELPFPIAALEPHISARTFEFHHGKHHKAYVDNTNKLIEGT-PLAGKDLESIILAAAG--DQAKKGLFNNAAQVWNHSFFWKCLKPGGGGKPTGKVAARIDADLGGYEKFAADFKNAGATQFGSGWAWLVLK-D-GKLEIAQTANAETPLTKGHKPLLVVDVWEHGYYLDYQNRRPDFLQAFLHLVNWDFVNANLG----

>A0A0M9UDM4_872965_Chloroflexi_B_Ter

MAHELPALPYAYDALEPYIDARTMEIHHTKHHQGYVNNLNKALEAYPHLQDKSVEELLRNIESVPEDIRTAVRNNGGGHANHSLFWTIMAPNAGGAPSGELAAAIDAAFGSFDAFKEAFSKAAATRFGSGWAWLVVTAF-GELKVYSTPNQDSPYMFGHTPILGLDVWEHAYYLNYQNRRGDYIGNWWNVVNWDKVAEYYAAAKK

>A0A0N0JCV7_1523424_Proteobacteria_B_Mar

MEHTLPALPYAIDALAPHYSQETLEFHHGKHHNAYVVNLNNLQKGT-EFESLSLEEIIKKS-------SGGIYNNAAQIWNHTFFWNCMKPAGGGEPTGALAAAINAKWGSYAAFREAFVKSAVGNFGSGWTWLVKKAD-GSVDIVNTGAAGTPLTTADKALLTVDVWEHAYYIDYRNMRPKFVETFLKLVNWGFAEANFA----

>A0A0N0K024_1523432_Proteobacteria_B_Mar

SPLAQPPLPFADTALEPVISAKTLTFHYGKHHKGYFDTLHKLIADT-PHADATLEEIIVAAAA--DPAQKKVFNNAAQAWNHNFYWNSLCGD-RQTPEGDLAAAIDRDFGSLDGCKAALAEASINQFGTGWGWLVVD-G-GTLKAVSTEDADVPFVHGQVPLLTVDVWEHAYYLDYQNRRPDHVKAVVSHLNWAFAARNFAQS--

>A0A0N1BGP9_1523432_Proteobacteria_B_Mar

MPTEPQPLPFAAADL---LSDKLITSHHDNNYTAAVKRLAPLRAQIAGLD-PAAAPGFA-WN----GLKREELIAWNSMILHELYFAGLVKGAAMAP--GLGAAIERDFGSVARWQGEFAAMGKLGGGSGWVLLTWSPRDGRLTNQWASDHSQTLA-GATPLLALDMYEHAYAIDFGSKAAAYVDGFMVNHGWAEANRRFARV--

>A0A0N1LB93_1523428_Proteobacteria_B_Mar

MEHKLPELPYAMDALAPHISKETLEFHYGKHHQTYVTNLNNLIKGT-EFESMSLEDIVRKS-------SGGMFNNAAQIWNHTFYWFGFKPNGGGDATGAVAAAINAKWGSFAAFKEAFNKSALGNFGSGWTWLVKKAD-GTLDIVNTSNAATPLTTSDKPLLTCDVWEHAYYIDFRNRRADYLGSFWSLVNWDFVNQQFA----

>A0A0N8HZX4_699431_Halobacteria_A_Arc

TEYELPPLPYDYDALEPSISEQVLTWHHDTHHQGYVNGWNSAEETLAENRENGEF---GDSA----GAIRNVTHNGSGHILHDLFWKNMSPEGGDEPSGALADRIAEDFGSYEAWKGEFEAAAG--AAGGWALLVYDSYSNQLRNVVVDKHDQGALWGSHPILALDVWEHSYYYDYGPARGDFIDAFFDVVDWDEPAARYEQAVE

>A0A0N8VL92_312540_Thermoplasmata_A_Arc

ATETWE----VKEKLRPRISDTQIDYHFDAHYKGYVAKLNEIWSKLPSVDLSKANQNYSDLR----EMKLEETFNYDGSMLHEYYFESLTKD-HVEMPASVKEQIEKDFGSYENFVALFKATGT--AFRGWAHLIFDLNYGKLRVVGADIHSAAAIWNALMILPLDVYEHAYYVDYGAKRAPYLDAFMKNVNWKVVEKRLERAKR

>A0A0P7GPP0_699431_Halobacteria_A_Arc

SDYELPPLPYDYDALEPSISEQVLTWHHDTHHQGYVNGWNSAEETLAENRENGEF---GSSP----GAIGDVTHNGSGHILHDLFWKNMSPEGGDEPSGSLRDRIEEDFGSYEAWKGEFEAAAG--AAGGWALLVYDSYSNQLRNVVVDKHDQGALWGSHAILALDVWEHSYYYDYGPARGDFVDAFFDVVDWDEPSARYEQAVE

>A0A0Q4BCN0_1713724_Thermoplasmata_A_Arc

GFYSLPELKYGYGDLAPFISEQLLKIHHDGHHQKYVNQANALLEKLDKARQDGNM---PPMK----CDVQALSFNVGGHYLHSLFWDNMAPAGGGKPGGLIADLLDKEFGSFDRFKKEFTEVANSVESSGWAVLVMCLQTGRPLLVQVKDHHLYSIPGFRILMVLDVWEHAYYLDYRNEKARYNQAFWDVVNWDKVDERAENIVG

>A0A0Q6BJ75_1736373_Proteobacteria_B_Mar

MEHTLPPLPFAKDALAPHMSEETFEYHYGKHHQAYVTNLNNLIKGT-EFENLSLEDIIKKS-------SGGIYNNSAQIWNHTFFWNCLTPNGGGEPTGALADAIKAKWGSYEEFKKAFQTSAVGNFGSGWTWLVKKAD-GSVDIVNMGAAGTPLTTGDKALLTIDVWEHAYYIDHRNARPKFVETFLNLVNWEFAAKNFAA---

>A0A0R2HP56_1449336_Firmicutes_B_Ter

MTYQLPELPYAYDALTPYIDEETMHLHHDKHHNTYVTNLNAAIEKHPELGSKTIEELMSNLDAIPEDIRTAVRNNGGGHVNHTFFWEILAPNAGGVPTGEVKEAIDATFGSFDKFKEEFAAAATTRFGSGWAWLVLD-G-GKLSIISTPNQDSPLSEGKTPILGLDVWEHAYYLNYKNVRPEYIKAFWNIVNWDEVAKRYAAAK-

>A0A0S2HVA3_1307839_Bacteroidetes_B_Mar

HPYKLPELNYEYDALEPTIDELTMKTHHSKHHQGYTNKANKFIEQY-NLTGKPVVQIFAEITQHP----VSVRNNGGGFYNHSLFWTFITP-GGSDFNGEVAEAIKKEFGSFDDFKTAFEKQAATQFGSGWAWLVMTPE-GKLAVTQSSNQDNPLMPNGVPLLNLDVWEHAYYLEYQNKRTEYISNFWDIVNWEVVNERYLMAKK

>A0A0S2I3Z7_1307839_Bacteroidetes_B_Mar

MKFTLPELPFEKDALEPHISAKTIEFHYGKHHKTYVDKLNKLVEGT-EFANADLETIIKNA-------DGGIFNNGAQVWNHTFYFEALSGNPKAKPGGKLLGAIDKVFGNYEQFIEDFTNAGTTLFGSGWVWLIQNKD-GDLDIMQGQNAENPIRDGYKPLMTMDVWEHAYYLDTQNARPKYIENFFAVLDWEVIEKRLG----

>A0A0S2I4J1_1307839_Bacteroidetes_B_Mar

MSFKLTNLPYDFNALEPYIDAQTMEIHHDKHHGGYTKKLNAALEK-EGVNNNNIEDILSNVSKYS----VGVRNNGGGYYNHNLFWEIMSPNGGGEPTDEIGKAIKETFGSFDAFKDEFAQAAAGRFGSGWAWLVKD-N-GKLKIGSTPNQDNPLMDKGQPILGLDVWEHAYYLKYQNRRPEYIDAFFNVINWDKVNELYKA---

>A0A0S2JHC6_58049_Proteobacteria_B_Mar

MAIELPALPYEQNALEPHISAETLSFHYGKHHNTYVVKLNGLIGGT-EFENKSLEEIVKSS-------SAGIFNNAAQIWNHTFYWNSLSPNGGGEPSGDLLAAINSNFGSFDEFKAKFTDSAINNFGSSWTWLVQNAD-GSLAIVNTSNAATPLTDGVTPLITVDLWEHAYYIDYRNVRPDYLKGFWALANWDFAQANFS----

>A0A0S2KFC6_1249552_Proteobacteria_B_Mar

MKLELPELPYAANALEPHMSAETFSFHHGKHHNAYVVKGNELLADA-GIDADNLEDLVRESAK----VGGPLFNNVAQVFNHNFFWKSMKPNGGGAPTGAIADKINADFGSYDNFKKEFVNGGVGQFGSGWVWLVLD-G-GKLKIAKTPNAECPLTTSAKPILVCDVWEHAYYLDYQNRRPDFLTSFLNLVNWDFANENLA----

>A0A0S7C1K2_1678841_Bacteroidetes_B_Mar

MKFELPPLPYAPEALEPVISAKTISFHHGKHHQAYVNNLNNLIVGT-RFENATLEEIIRES-------DGGIFNNGAQVWNHTFYWEGLGGK-GGTPAGKLLEKINTSFGTFENFKAVFADAAAKLFGSGWAWLVEDQE-GALKIVQTSNAGNPLRDGLKPLFTCDVWEHAYYLDFQNRRPDYISAFWDIVNWDKVAERL-----

>A0A0S7C2S3_1678841_Bacteroidetes_B_Mar

AGLTFPALPYDLDALEPVIDKLTVEIHYDRHHRAYYNNFLKAVGG-TEMETMTIFEIFSKMSELP----VSVRNNGGGFFNHVLYWNNLSPKGGGEPGGELGEAITKYFGSFEAFRAKFDEAAKTRFGSGWAWLSVDPEKGELFISSTANQDNPLMNRGFPILGIDVWEHAYYLKYQNKRADYVESFWKIVNWQDVEARYKQFHE

>A0A0S7C7E3_1678841_Bacteroidetes_B_Mar

MKFEFPALPFAYDALEPHIDRMTMEIHHTKHHRAYFDKFTAAIQG-TELENKSLEEIFASISKAP----AAVRNNGGGFYNHNLFWEILSPKGGGLPGGKLAEAINKDFGSFEAFKTKFNDAAANRFGSGWAWLSVKAD-KSLCVCSSPNQDNPLMDPGLPIMGLDVWEHAYYLKYQNRRPDYINAFWSLVNWDKVSENYEKAL-

>A0A0U3DNG9_1768242_Proteobacteria_B_Mar

MEHLLPALPYAKDALAPHYSAETLEFHHGKHHNAYVVNLNNLQKGT-EFEALSLEEIVKKA-------SGPIYNNAAQIWNHTFFWNCMKPNGGGAPTGALADAINAKFGSFEAFKEAFTKSAVGNFGSGWTWLVKKAD-GTVDIVNTGAAGTPLTTADKALLTIDVWEHAYYIDYRNLRPKFVETFLSLANWEFAQANFA----

>A0A0U5CYX4_1407499_Halobacteria_A_Arc

ANYELSPLPYDYDALEPHVSEQVLTWHHDTHHQGYVNGWNAAEETLEANREEGDF---SASA----GAIGDVTHNGSGHILHDLFWQSMSPEGGDEPEGDLRARIEEDFGSYEAWEGEFRAAAS--AAGGWALLVYDSHSEQLRNVVVDKHDQGALWGSHPILSLDVWEHSYYYDYGPDRGDFVDNFFEVVDWEEPAARYADLVE

>A0A0U5H1X7_1407499_Halobacteria_A_Arc

SDYELPPLPYDYDALEPHISEQVLTWHHDTHHQGYVNGWNSAEETLEANREEGDF---SSSA----GAIGNVTHNGSGHILHSIFWNNMSPNGGGEPAGALAARIEEDFGSYEAWKGEFEAAAS--AAGGWALLVYDSHSEQLRNVVVDKHDQGALWGSHPILALDVWEHSYYYDYGPDRGDFVDNFFEVVDWDDVADRYEEVAA

>A0A0W0GJW2_1217799_Chloroflexi_B_Ter

-------MAYDTKKLLG-FSDNLLNNHF-TLYQGYVKNLNRLVETIDALSKAGKS-DTPEYA----ELQRRFGFEWNGMRLHEYYFDNLGGTGLAVSGGKLAQNLAATWGSREAWEKDFRATGA-MRGVGWVVLYQDIQNGRLFNTWINLHESGHLTGCQPVLVMDVWEHAFMIDYGLKRAEYIEAFFRNIDWSVCELRIK----

>A0A0W0TNM8_453_Proteobacteria_B_Mar

MAFTLPQLPYAMNALEPHISQETLEYHYGKHHNAYVTNLNKLIPGT-EYENLSLEEIIKKS-------SGGIFNNAAQVWNHTFYWHCLSPNGGGEPTGKLADAIKKDFGSFAAFQEQFTQTAATTFGSGWAWLVQEKD-GSLKITSTSNAGTPMTAGQQALLTCDVWEHAYYIDYRNVRPDYIKAFWSLVNWEFVESNMR----

>A0A0W1R4R4_1514971_Halobacteria_A_Arc

MSYELDPLPYDYDALEPHISEQVLTWHHDTHHQGYVNGWNSAEETLAENREAGEF---GSSG----GAMRSVTHNSSGHILHDLFWQNMSPEGGEEPSGDLADRIAEDFGSYDAWKGEFEAAAK--DASGWALLVYDTFSNQLRNVVVDKHDQGAIWGGHPILALDVWEHSYYYDYGPARGDFIDAFFEVVDWDEPSTRYDQAVE

>A0A0W1R5I9_1514971_Halobacteria_A_Arc

ATYELPELPYDYDALEPSIDARIMELHHDKHHQGYVDGANSALDTLEEMRSNGDY---GDIK----GVERNLAFNLSGHVNHTVFWENMSPDGGGEPGGELADALDEHFGGFDQFKDHFSAAAKGVEGSGWGFLAHDHVADKPIVTMAENHQNQTPQGVTPLLVIDVWEHAYYLQYENGRGDYVDNFWDIVNWDDVAERYDRAQS

>A0A0W1RIP5_1544718_Halobacteria_A_Arc

SDYELDPLPYDYDALEPHISEQVLTWHHDTHHQGYVNGWNAAEETLAENRDAGDF---SSSA----GALRNVTHNGSGHILHDLFWQNMSADGGAEPEGALADRIAEDFGSYEAWKGEFEAAAG--AAGGWALLVYDSFSNQLRNVVVDKHDQGALWGSHPILALDVWEHSYYYDYGPARGDFVSAFFEVVDWEEPSARYEQAVE

>A0A0W1SLK0_1544718_Halobacteria_A_Arc

MSYELDPLPYDYDALEPHISEQVLTWHHDTHHQGYVNGWNAAEETLAENREAGEF---GSSA----GALRNVTHNGSGHILHDLFWQNMSPEGGDEPEGALADRIAEDFGSYEAWKGEFEAAGS--AAGGWALLVYDSFSNQLRNVVVDKHDQGALWGSHPILALDVWEHSYYHDYGPARGDFISAFFEVVDWEEPSARYEQAVE

>A0A0X3BJM4_86622_Methanomicrobia_A_Mar

KKYELPPLPYAPDALEPHISKEQLSLHHDKHHQAYVTGANANLERLEKARREGTD---VDMK----ALLKELSFNIGGHILHTLFWPGMAPAGGGTPGGALADLIDREWGSFDRFKAEFSKAAASVEGSGWAALAYCTMTDRPMIMQIEKHNNNVYPTFQILMVLDVWEHAYYVDYKNNRGQFVDAFWNVANWGEVNRRLEKI--

>A0A0X3T641_1685380_Proteobacteria_B_Mar

MAFTLPDLPYDYNALDAAIDAQTMEIHHTKHHQTYVTKLNDAIKDSPN-ADKSLDELIANAGSIS----PAVRNNGGGHWNHSFFWEIMTGDSMGAPSGALADDIKATFGSFEDMKAQFNNAGATQFGSGWAWLTVSAE-GKLEISSTPNQDNPLMDKGTPILGLDVWEHAYYLRYQNRRPDYMQAWWDVVNWNKVEALYNAAK-

>A0A139SPB8_1680762_Proteobacteria_B_Mar

MAFVLPPLPYEKNALEPHISSETIEYHYGKHHNTYVVNLNNLVPGT-EFEGKSLEDIIKTS-------SGGIFNNAAQIWNHSFYWECLSPSGGGEPGGELGKAIDTAFGSFAKFKEEFTKTAIGTFGSGWAWLVKKAD-GSLALASTIGAGCPLTSGDTPLLTCDVWEHAYYIDYRNLRPKYVEAFWNLVNWDFVAKNYAA---

>A0A142L0Q1_1690483_Bacteroidetes_B_Mar

MSFELPKLPYEYTALEPHMDARTMEIHHSKHHNAYVTNLNNAIAG-TDAEKLTIEEICKHISKYP----AAVRNNGGGHYNHSFFWTILS-GTPSSPSAKLEAAINADLGGMDKFKEEFNKAATTRFGSGWAWLCVE-N-GKLKVCSTPNQDNPLMDNGTPVLGLDVWEHAYYLHYQNRRPDYCTAFWNVVNWAEVSRRFEAAL-

>A0A142L7E6_1690483_Bacteroidetes_B_Mar

SAFELPLLGYSFDALEPFIDAQTMQIHHDKHHLAYITKLNEAVVKEVSLQNQSLEQLVKNISKHPESIKTAIRNHGGGHWNHSFFWTLLKPN--TKPSEKMEKVITDNFGSMEQFKTNFEKAAMSVFGSGWAWVIKD-G-DKLAITTSANQDNPLMDQGTPVMGLDVWEHAYYLKYQNKRADYLQAFWNVLNWNKVEELYTTK--

>A0A142LJU4_1690485_Proteobacteria_B_Mar

TVFAQVALPYAKDALVPHVSAETIDFHYGKHHLTYVTNLNNLAKGT-EFENLTLEEVVKKA------PAGAIFNNAAQIWNHDFYFLGFKPGGGGKPSGALAAAIDKQFGSFEEFQKQFDAKAAGTFGSGWAWLCKKAD-GSLSLESTSNAATPLTQGMTPLLTCDVWEHAYYIDYRNSRPNYLKGFWAIVNWDFVAANFAK---

>A0A142X6W1_1632864_Planctomycetes_B_Mar

MAYTLPALPYAYNALEPHIDARTMEIHHTKHHQAYINNVNKALEGHADLAALPVDDLMKKLSSVPEAIRTVVRNNGGGHSNHTLFWTVLAPNAGGAPNGAVASAIDGAFGNFDTFKTQFNDAATKRFGSGWAWLSVD-K-GKLVVESTANQDTPLSEGRTPILGLDVWEHAYYLNYQNRRPDYITAFWNVVNWAEVNKRLAAAK-

>A0A143PHC2_1855912_Acidobacteria_B_Mar

MPYELPPLPYAHNALEPFIDEQTMQIHHGKHHQTYVNNVNAALEKYPELQGKPIDQLIADLNAIPEDIRTAVRNNGGGHANHTFFWAVMAPNAGGSPTGKIAEAINAKFGSFDAFKEAFAKAGTTRFGSGWAWLIKS-G-SGVEVTSTPNQDSPLMEGKMPLLGLDVWEHAYYLKYQNKRPDYIAAWWNVVNWDAVNNAF-----

>A0A149W187_1789004_Proteobacteria_B_Mar

VTHELMTLPFAMDALAPHISKETLEYHYGKHHRAYVNKLNELIKGT-EFEGLSLSDLVRKT-------TGPTFNNAAQVWNHNFYWYGLNPRGSDKPTGELAAAINKNFGSFDQFAAQFKTAAATKFGAGWTWLVKTED-GQLAIRNSNDAENPLQWNQIPLLTCDVWEHAYYIDYRNERPRYIETFWALVDWDFVARNLEGEGI

>A0A161XL90_1679489_Halobacteria_A_Arc

SNAELPPLPYDYDALEPHISEQVLTWHHDTHHQGYVNGLNSAEETLAENRESGDY---SSTA----GALGNVTHNGCGHYLHTLFWENMDENGGGEPEGELADRIEEDFGSYEGWKGEFEAAAS--AAGGWALLVYDPVAKQLRNVKVDKHDQGALWGSHPILAVDVWEHSYYYDYGPDRGDFIDNFFEVVDWDNVAEQYDNVVS

>A0A162GML5_1822219_Proteobacteria_B_Mar

MAIELPALPYAIDALAPHISQETLEFHHGKHHKTYVDKLNGMIPGT-EYEGKTLEEIITSS-------SGPVFNNAAQIWNHTFYWNCLSPNGGGAPTGKVADAINEAFGSFEEFQAAFNDKAVNNFGSSWTWLVKNAD-GSLEIVNTSNAGTPMTEGQTALLTVDLWEHAYYIDYRNVRPTYLNAFWALVNWGFVAENLG----

>A0A166S5V7_1679489_Halobacteria_A_Arc

ATYELPELPYSYDALEPSIDAHIMELHHDKHHQGYVNGANAALDTLEAMRENDEW---GDVK----GVERNLAFNLAGHINHSVFWENMSPDGGGEPGGELADALDDQFGSFDEFKANFSAAAKGVEGSGWGMLLYDHVGEIPIVTMAENHQNQSPM-ATPLLVLDVWEHAYYLQYENNRGEYVDNFWDVVNWDDVEERYDEASS

>A0A191ZFD3_1860122_Proteobacteria_B_Mar

MAFQLPELSYDYGALDKSIDAQTMEIHYSKHHGAYVTNCNNAIAG-TEWDDKPIEDIMANVSKLT----PAVRNNGGGHFNHTLFWSILSPNGGGEPTGALADDIASTFGSFSAFRDQVNTAGATRFGSGWAWLVVTPE-GKLAVCSTPNQDNPVMDKGTPILGIDVWEHAYYLRYQNRRPDYLKAIWDVISWDAVAQRYADAK-

>A0A192D2N1_1112_Proteobacteria_B_Mar

MAFKLAPLPYPDTALEPAISAETLSYHHGKHHQTYVDKTNAAIEGT-DHADKSLEEIIAAARG----SDKGLFNNSAQTWNHAFYWHSMAPE-TTEASDDLAAKIDEAFGSVEDLKQKLKDRGAGHFASGWVWLAEK-G-GKLSIEETHDADTLADGDFNPLLLIDVWEHAYYLDHQNKRPAYLDAVVKKLNWAFASENLARGTV

>A0A192D580_1112_Proteobacteria_B_Mar

AAFTLAPLPYGYDALEPVIDTATMTVHHSKHHQAYVDNLNKAVAADPALAGMTIEQLVVRAGTLP----AAVRNNAGGHWNHTFFWETMAPAARGAISPALAAAIDAQFGSMDAFKAAFKAAGTARFGSGWVWLIVGAD-GKLAITSTPNQDNPLMDRGTPIIGNDVWEHAYYLKYQSRRADYLDGWWQVVDWGKVSARYAAANA

>A0A192WU48_1660251_Acidobacteria_B_Mar

MAHELPSLPYAFDALEPHIDARTMEIHHGKHHAAYVSKLNAALEGHDGLAAKSIEDLMASLDEVPEDIRGAVRNNGGGHCNHSLFWKIMKPGGGGEPGGELADAINSVFGSFADMKKTLINVGVGQFGSGWAWLGIK-D-GKLHAGGTANQDSPLMFGGTPILGVDVWEHAYYLNYQNRRPDYLEAWFNTVNWDEVAANLKNA--

>A0A193LCR6_1548547_Proteobacteria_B_Mar

MKFEMKPLPYAYDALQPHISEQTVKFHYDKHHTGYMTKLQGQLAGT-PDEQKSLQEIVETS-------SGGVFNLAAQIWNHDFYWQSLTPNGGGAPGGELAEMIAAEFGDFATMRGKLKDEALGHFGSGWAWLVLGKD-NKLQICSTHDADNPLRAGATPLLTVDVWEHAYYLDTQNDRGGYLDRVLNLLNWQFAEDNLSAARK

>A0A1A8XY42_1860102_Proteobacteria_B_Mar

MEHQLPQLPFALDALAPHMSRETLEYHHGKHHQAYVTNLNNLIKGT-EYEALDLESIIRKAP------AGGVYNNSAQVWNHSFFWNCLTVNGGGAPGGALAAAIDARWGSFAEFSKAFQASAVGNFGSAWTWLVKKAD-GSVDIVNMGAAGTPLTTGDQALLCIDVWEHAYYVDYRNLRPKYVETFLNLANWGFAEQNFAG---

>A0A1B1YPZ1_1810504_Proteobacteria_B_Mar

MTHELPPLPYAIDALAPHISAETLEFHHGKHHKTYVDNLNKLIPGT-EFENLSLEDIVRKS-------SGGIFNNAAQIWNHTFYWNCLAPKAGGKPTGALAAAIDTAFGSFDTFKEKFSQTAITTFGSGWGWLVKNAA-GGVELVSTSNAGCPLTAGQTPLLTCDVWEHAYYIDYRNARPKYVESFWNLVNWDFVARNFG----

>A0A1B4XH23_1620215_Proteobacteria_B_Mar

YPFALAPLPFAYDALEPYIDAATMRIHHDKHHQTYITRLNAALETYPEWHGLSIEKLLLERSELPIAIRQTVHDQGGGHLHHQLFWEILKPGAGDRPTRALAQAIDRSFGSFDAFKARFVDTGARHFASGWVFLLVNPADGKLEILSCHDHDSAVLENKTALLLNDLWEHAYYLKYQSGRVDYLKVFWNVVNWEHVGHRLESALA

>A0A1B6B8G0_1048380_Firmicutes_B_Ter

MKFQLPNLTYAYDALEPHFDKQTMEIHHTKHHAGYTNNLNAALEG-KDI-DLSIEAILKGLEAIPEDVRVAVRNNGGGFYNHSLFWTLLSPNGGGEPKGKLKEALVKQFGSFETFKENFNEAAAKRFGSGWAWLTIK-D-GVLGIESTANQDSPLSDGKDVLLGLDVWEHAYYLKFQNKRPDYISAFWHLVNWDEVESRFEQFK-

>A0A1C0VXQ7_1880991_Cyanobacteria_B_Ter

MAFELPPLPFAADALESAMSANTFSFHHDKHHAAYVTNLNKLIEGT-ELADKSLEEIVSISFK--DPSKVGIFNNAAQVWNHTFFWSSLKPNGGGIPTGTLADKIAADFGSFDKFKDDFKAAAATQFGSGWAWLVLD-N-GALKVTKTPNAENPVVHGQIPLLTLDVWEHAYYLDFQNRRPDFIANFLNLANWDFAAENFAKAA-

>A0A1D2W9T7_1860100_Methanobacteria_A_Arc

GFYELPELDYGYKDLEPHISEQQLKIHHQKHHQAYVDAANAIFKLYDEAREKGED---FDVK----AKAKELAFNAGGHQLHTLFWKNMGPANGGEPTGTIAEYIKKDFGSFERFKKEFSQAAITTEGSGWAVVTLCKSTDRLIILQLEKHSVNTAPRWPPLMVLDVWEHAYYLDYKNVRPDFVAAFWNIVNWDEVNTRVDAWLK

>A0A1D3L2G7_118062_Methanobacteria_A_Arc

NFYELPELPYGYKDLEPYISEEQLKIHHKKHHQAYVDGANALLKKFDA-RAEEEE---FDLK----AVSKELSFHVGGFILHKLFWANMGPSCGGEPTGLIADYIEKDFGSFERFKKEFSQTAVSTEGSGWAALTLCRGTDRIFIMQIEKHNVNTVPGFRLMMVLDVWEHAYYLEYQNRRPDYVEAFWNLVNWDEVNRRIKVWLD

>A0A1D8S4L0_1873524_Halobacteria_A_Arc

SHPELPELPYDYDALEPHISEQVLEWHHDTHHQGYVNGLDSAEEALAEARETGET---HGTQ----AALRSVSHNGSGHYLHTMFWDNMSPNGGGEPEGALRERIEEDFGSYEAWRQEFEEAAG-YSAGGWALLVYDPVAKQLRNLAVQKHNDGALWGAHPILALDVWEHSYYFDYGPARGEFVDAFFEVIDWDDVAENFEKVSS

>A0A1E2V0Q9_1818881_Proteobacteria_B_Mar

MAHELPALPYAIDALEPVISKETLEFHHGKHHNTYVTNLNNLIPGT-EFENASLEDIIMKS-------SGGVFNNAAQIWNHTFYWNCLSPSDDNAPTGALADAINNTFGSFDEFKKKFATSGATNFGSGWTWLVQNAD-GSLEIFNTSNAGSPMTSGKKALLTADVWEHAYYIDYRNARPAYLEAFWKVVNWDFVAGNMS----

>A0A1E2V9J2_197479_Proteobacteria_B_Mar

MAFELPALPYEKNALEPHISQETLEYHYGKHHQTYVTKLNGLVEGT-DNAGKSLEELIKTAP------AGGLFNNAAQVWNHTFYWNCLSPNGGGEAKGAIADAINSKWGSFADFQKDFDEKAAANFGSGWTWLVKNSD-GSIEIVNTSNAGNPLTDGQTPLLTVDVWEHAYYIDYRNSRPNYLNAFWSLVNWDFVNQNFA----

>A0A1E2ZEY7_1655433_Proteobacteria_B_Mar

MAHELPALPYAIDALEPVISKETLEFHHGKHHNTYVTNLNNLIPGT-EFENASLEDTIMKS-------SGGVFNNAAQIWNHTFYWNCLSPNDDNAPGGALAGAIDAAFGSFDEFKKQFATSGATNFGSGWTWLVQNDD-GSLEIFNTSNAGTPMTSGKKALLTADVWEHAYYIDYRNARPAYLEAFWKVVNWDFVASNMS----

>A0A1E3VXW9_1774968_Proteobacteria_B_Mar

MAFELAPLPYAYDSLQPYMSAETLEYHHDKHHKAYVDNLNKLIEGT-DYEGKDLETIIKESFG----KDAGVFNNAAQNFNHIHFWPWMTKDGGGKVPGAILALIDRDLGGFDKFRADFLAAGATQFGSGWAWLTLK-D-GKLEVTKTPNGENPLVHGGWPLLGCDVWEHSYYIDYRNARPKYLEAWFNLINWDHVEELYAQAPG

>A0A1E4UW72_1843690_Proteobacteria_B_Mar

MAFELPPLPYAHDALQPHISKETLEYHHDKHHNTYVVNLNNLVPGT-EFEGKTLEEIVKTS-------SGGIFNNAAQVWNHTFYWNCLAPNAGGEPTGALAEAINTAFGSFDKFKEEFSKVSIGTFGSGWGWLVKKAD-GSLALASTIGAGCPLTSGDTPLLTCDVWEHAYYIDYRNVRPKYVEAFWNLVNWDFVAKNFAA---

>A0A1E4V0M9_1843690_Proteobacteria_B_Mar

MPHTLPPLPYAYAALEPHVDAQTMEIHHSKHHQAYVNNLNAALSG-HALAELPVETLLTRLQELPEAMRAAVINNGGGHANHSLFWTVMSPTGGGEPEAELRAAIDTQLGGFEAFKAAFTQAALSRFGSGWAWLSVTVD-KTLVVHSTGNQDSPLMNGLTPVLGLDVWEHAYYLRYQNRRPEYIAAFYNVVDWAEVSRRYHDALS

>A0A1E5QE38_1781255_Cyanobacteria_B_Ter

MAYELPPLPYAYDALEPHINKATLEFHHDKHHAAYVKNYNSMVEGT-EYADKPIEEVIKAIYG--DSSKSGMFNNAAQAWNHTFYWNSMKPNGGGTPTGAIADKINADFGGFDKFKEEFSKAGATQFGSGWAWLVLE-N-GTLKVTKTLNAENPLHNGQTPLLTMDVWEHAYYLDYQNKRPDYISTFISLINWDFVASNYSAAA-

>A0A1E5QNR3_1781255_Cyanobacteria_B_Ter

GPFQLPSLAYSYDALEPHIDARTMEFHHSRHHQAYVNNLNNAIARHPELRDRSLAELLGNLNNVPEDIRTTVRNNGGGHANHTMFWEIMGPNGGGEPTGAIANAINQNFGSFEQFQEQFNSAGGDRFGSGWVWLVRTPN-GQLQITTTPNQDSPLMEGNYPIMGNDVWEHAYYLNYQNRRADYLEAWWNVVNWDAVNQRYQSVAS

>A0A1E8CFB9_1524254_Proteobacteria_B_Mar

MKLELPDLPYAANALEPHMSAETLGFHHGKHHNTYVVKGNELLADA-GVDADNLEDLVRATAK----VGGPLFNNVAQVYNHNFFWQSMKPSGGGMPSGAIADKINADFGSYDDFKKEFSAGGVGQFGSGWVWLVLD-G-GKLKIAKTPNAETPLTTSAKPILVCDVWEHAYYLDYQNRRPDFLAAFLNLVNWDFANQNLA----

>A0A1G6AIZ5_439219_Firmicutes_B_Ter

MAIILPDLPYAYDALEPYIDAETMTLHHDKHHATYVANANAALEKHPE-IGEDLVALLSDVEQIPADIRQALINNGGGHLNHALFWELLSPE-KTEITEEVAAAINEAFGSFDAFQEAFTVAATTRFGSGWAWLVVNAE-GKLEVMSTANQDTPIMEGKQPILALDVWEHAYYLNYRNVRPNYIKAFFEIINWTKVAELYAAAKA

>A0A1G6E3R2_617002_Proteobacteria_B_Mar

SSFTLMPLPYPEDALEPVISAKTLQFHHGKHHQKYVDTLNKLISGT-EYVNKSLEEIIRDTDG--KEDKSKIFNNAAQVWNHQFFWKSLKPNGGGMPSNALMEKIEGSFGSFEDFKTGLSEAAVSQFGSGWAWLVYD-G-DGLKVMKTSNAKVPFTQGLKPLLTIDVWEHAYYLDYQNLRPKYVEAVIKLLNWEFALENMR----

>A0A1G6EXC4_617002_Proteobacteria_B_Mar

SPLALPPLPYAENALEPVITARTIGFHYGKHHKGYVDNLNKLVAGT-EYAEMPLREIIIGSAG--QPENSAIFNNAAQIWNHTFYWNSMKPKGGGEPPTALKQRMEASFSSVDACKQELAAAATTQFGSGWAWLVLD-G-DTLKVVKTGNADLPLTNGMKPLLTIDVWEHAYYLDYQNRRADYVNTVLNLINWDFAAKNLG----

>A0A1G6HPU7_1640674_Bacteroidetes_B_Mar

MPFELPKLPYDYNALEPYIDVTTMEIHHSKHHGAYTSNLNSAILG-TELENMSLDDILKDISKRP----VAVRNNGGGYYNHNLFWTILSPNGGGEPTGDIAKAINSQFGSFAAFKEEFNKAALSRFGSGWAWLVDV-Q-GRLVISSTPNQDNPKMDKGYPIFGIDVWEHAYYLKYQNRRNEYIAAFWSLINWDEINRRYSCI--

>A0A1G6HQ17_1640674_Bacteroidetes_B_Mar

MIHELPKLPYSIDALEPHLSKKTFEFHYGKHHQAYVTNLNNLIVGS-KFENATLETIIREG-------DGGIFNNGAQVWNHTFYFFQFSSEPLLMPSGPLAKAIDASFGSLPEFKEQFNKACATLFGSGWVWLAKNPQ-GKLEIIQESNAGNPLRQGLVPLLTCDVWEHAYYLDQQNRKPDYIQAFWNVLDWKIIEGRF-----

>A0A1G6RPM1_1640674_Bacteroidetes_B_Mar

RAYPFSALPYAYDALEKFVDKETMEIHYARHYKAYHSKFLAAIQG-SGMEGKSMEEIFASMSKL----SPAVKNNGGGYFNHKLFWEVMAPNGGGEPAGILMPAIVEAFGSFEAFKSQFEAAATGVFGSGWAWLAVDKT-GKLFIASTANQDNTLMDYGVPVLALDVWEHAYYLRYQNKRADYVSRFWTVVNWPMVEQKFAAAVP

>A0A1G8BH66_83767_Proteobacteria_B_Mar

MEHQLPQLPYAIDALAPHMSKETFEYHYAKHHQAYVTNLNNLIKGT-EYEALDLEAIVKKAP------AGGIYNNSAQVWNHSFFWNCLKPNGGGAPSGALAEAINKKWGSFDEFKKAFQASAVGNFGSGWTWLVKKAD-GSVDIVNMGAAGTPLTTGDKALLCIDVWEHAYYIDYRNLRPKFVETFLSLANWSFAEKNFG----

>A0A1G8W4P7_890420_Halobacteria_A_Arc

SNPELPELPYEYDALEPHISEQVVTWHHDTHHQGYVNGLDSAEETLAENREDGDF---GSSG----SAMRDVTHNGCGHYLHTLFWENMHPDGGGEPEGELRDRIEEDFGSYEGWKGEFEAAAG--AAGGWALLVYDPVAKQLRNVVVDKHDQGALWGAHPVLALDVWEHSYYYDYGPDRGEFIDNFFEVVNWDKAAEEYETCLA

>A0A1G9F980_1075417_Bacteroidetes_B_Mar

MAFELPSLPYAANALVPHIDQQTMEIHHGKHHGGYVAKLNAAVDG-TDMEGKSIEELLTHVSKYP----LAVRNNGGGHYNHRLFWKILSPNGGGQPTGELAQALDSAFGSFEAFKENFSDAAATRFGSGWAWLISQ-N-GKLALTSTPNQDNPLMDKGTPILGLDVWEHAYYLNYQNRRPDYISAFWNIVNWDQVAKNYQATL-

>A0A1G9H772_1075417_Bacteroidetes_B_Mar

MAFELPSLPYAYDALEPHIDAKTMEIHHTKHHGGYTTKLNGALEG-TDGASKSIEDILKNISQYS----GAVRNNGGGYYNHALFWPTLSPNGGGQPSGKLADAINAAFGSFDEFKKKFDDAAATRFGSGWAWLIVDGS-GKLAVTSTANQDNPLMDKGTPILGLDVWEHAYYLNYQNRRPDYISAFWNIVNWDQVTKNYEAAL-

>A0A1G9SJT8_660521_Halobacteria_A_Arc

MSYELDPLPYEYDALEPNISEQVLTWHHDTHHQGYVNGWNSAEETLAENREAGDF---GSSA----GALRNVTHNGCGHILHDLFWQNMSPEGGDEPSGDLADRIAEDFGSYEAWKGEFEAAAG--NAGGWALLVYDSFSNQLRNVVVDKHDQGALWGSHPILALDVWEHSYYYDYGPARGDFIEAFFNVVDWNEPSARYEQAVE

>A0A1G9VZ43_996166_Halobacteria_A_Arc

SHAELPPLPYDYDALEPSISEQVLTWHHDTHHQGYVNGLNAAEETLAENRESGDH---SSTA----GALGNVTHNGCGHYLHTLFWENMSPNGGGEPEGELRDRIEEDFGSYEGWKGEFEAAAS--AAGGWALLVYDPVAKQLRNVAVDKHDQGALWGAHPILALDVWEHSYYYDYGPDRGSFIDNFFDVVNWDKVAEEYSKVVG

>A0A1G9XG20_660521_Halobacteria_A_Arc

SDYELDPLPYDYDALEPNISEQVLTWHHDTHHQGYVNGWNSAEETLEAAREEGDF---SGSA----GAIRNVTHNGSGHILHDLFWNSMSPEGGDEPEGALADRIAEDFGSYEAWKGEFEAAAG--NAGGWALLVYDSFSNQLRNVVVDKHDQGALWGSHPILALDVWEHSYYHDYGPARGDFVEGFFNVVDWDEPSARYEQAVE

>A0A1G9XX20_996166_Halobacteria_A_Arc

ANHELDPLPYDYDALEPHISEQVLRWHHDTHHQGYVDGWNAADEALAANREAGTT---DGSA----DAIRDVTHNASGHVLHELFWQCMSPEGGDEPEGALRDRIEADFGSYGAWKAEFEAAAS--AAGGWALLVYDTHSQQLRNVVVDKHDQGALWGSHPILALDVWEHSYYHDYGPARGDFVEAFFSVVDWQEPTRRYGEAIA

>A0A1H3A4M8_1123352_Firmicutes_B_Ter

MVFKLPELKYSYNALEPHIDALTMETHHSKHHKAYVDNLNKALEGHAKSQEMDIEEILKSLTELPEEIRTAVRNNGGGHYNHTLFWEFMSPDGGGKPEGELANKIDEDLGGFDKFKEDFKKAALDQFGSGWAWLVLN-N-GRIEIVSTPNQDNPISQGKIPILGIDVWEHAYYLKYKNLRGDYVDAWWNVVNWKKVEEIFNKVK-

>A0A1H3ILX3_660517_Halobacteria_A_Arc

MSYELDPLPYDYDALEPHISEQVLTWHHDTHHQGYVNGWNAAEETLEENREAHDF---SSSA----GAIRNVTHNSSGHILHDLFWNSMSPEGGDEPTGALADRIEADFGSYEAWKGEFEAAAS--DASGWALLVYDSFSNQLRNVVVDNHDEGAVWGGHPVLALDVWEHSYYHDYGPARGDFVDNFFEVVDWEEPSARYEQAVE

>A0A1H3W6N9_555874_Halobacteria_A_Arc

MSYELDPLPYDYDALEPHISEQVLTWHHDTHHQGYVNGWNSAEETLEANREDGDF---GSSP----GAIRNVTHNGSGHILHDLFWNSMSPEGGDEPTGDLADRIEEDFGSYEAWKGEFEAAAG--AAGGWALLVYDSFSNQLRNVVVDKHDQGALWGSHPILALDVWEHSYYHDYGPARGDFVDNFFEVVDWEEPSARYEQAAE

>A0A1H3XW14_408074_Bacteroidetes_B_Mar

MAFTLPSLPYASDALEPHIDKQTMEIHHGKHHQAYVDNLNKAIAG-TENENKSLEELVANAGKIS----PAVRNNGGGHWNHSFFWTILGPNAGGAPKGKLADAINATFGSFEAFQEKFNNAGATRFGSGWAWLIVK-D-GKLEITSTPNQDNPLMDKGTPVLGIDVWEHAYYLKYQNRRPEYLKAFWNVVNWDEVGKRYESAL-

>A0A1H3Y937_408074_Bacteroidetes_B_Mar

APFVLPPLPYGYEALEPHIDKLTMEIHHDKHHAAYVKNLNDNIANTP-FANLTLEEIQHQVTE----KDKAIRNNGGGHYNHSLFWTLLSPT-KKTPSAKLKNAINGAFGSWEAFQTKFNDAAKSVFGSGWAWLIVTPD-KKLAVTTTPNQDNPLMNKGTPILALDVWEHAYYLKHQNKRPEYIEAFWNVVNWDEVEKLYNKA--

>A0A1H4F8V2_152573_Proteobacteria_B_Mar

MAFELPALPYAKNALAPHISEETLEYHYGKHHNTYVVKLNGLIEGT-DFAGKSLEEIVRTS-------EGGVFNNAAQVWNHTFYWHCLSPNGGGEPTGALADAINAKWGSFADFKAAFNDKAVNNFGSSWTWLVKKAD-GSLDIVNTSNADTPLAHGLTPVLTVDLWEHAYYIDYRNARPTYLDAFWALVNWDFASKNLAA---

>A0A1H6AL08_699433_Halobacteria_A_Arc

MSYELDPLPYDYDALEPHISEQVLTWHHDTHHQGYVNGWNAAEETLEENREAGDF---SSSA----GAIRNVTHNGSGHILHDLFWNSMSPEGGDEPTGALADRIEEDFGSYEAWKGEFEAAAS--AAGGWALLVYDSFSNQLRNVVVDKHDQGALWGSHPVLALDVWEHSYYHDYGPARGDFVENFFEVVDWEEPSARYEQAVE

>A0A1H6CZE7_568106_Proteobacteria_B_Mar

MSFELPALPYAKDALEPHISAETLEFHHGKHHNTYVTKLNGLVPGT-EFEGKTLEEIITSA-------PAGVFNNAAQIWNHTFYWYSLSPNGGGAPTGAIADAINAKWGSFEKFQEEFNDKAVNNFASCWTWLVKNSD-GSLEIVNTSNAGTPMTNGQKALLTVDLWEHAYYIDYRNVRPDYLKGFWALVNWDFANENFA----

>A0A1H6J821_1267564_Halobacteria_A_Arc

SEHELDPLPYDYDALEPHISEQVLTWHHDTHHQGYVNGLNAAEETLAENRESGDY---GSTA----SALGNVTHNGSGHYLHTLFWNNMSPNGGGEPAGDLADRIAEDFGSYEAWKGEFEAAAG--AAGGWALLVYDPVSKQLRNLKVDKHDQGALWGAHPILALDVWEHSYYHDYGPARGEFIDAFFEVVDWGEVADNYENVVS

>A0A1H6VKK8_1073996_Halobacteria_A_Arc

MSYELDPLPYDYDALEPHIDEQILTWHHDTHHQGYVNGWNSAEETLAENRDNGDF---SSSG----GAIRNVTHNSSGHILHDLFWQNMSPEGGDEPSGALAERIEEDFGSYDAWKGEFEAAAK--NASGWALLVYDSFSNQLRNVVVDKHDQGAIWGGHPILALDVWEHSYYYQFGPDRGGFIDAFFEVVDWDESDERYAQAVE

>A0A1H6VX19_1073996_Halobacteria_A_Arc

MTYELPDLPYDYDALEPHIDSRIMELHHDKHHQGYVDGANAARDALEGMRDSEDF---GDIK----SVKRALAFHLSGHVNHTIFWENMHPDGGGEPGGELADHIEADFGSFEAFKSEFAAAAGGVEGSGWGLLCHDAASDELIIAAAENHQNQTPQATTPILVCDVWEHAYYLQYENNRGEYVDNFFEVIDWDDVAQRFEQCHG

>A0A1H9BZT2_1186196_Halobacteria_A_Arc

ADHELPPLPYDYDALEPALSEQVLTWHHDTHHQGYVNGLNSAEETLAENREEGDF---GSTP----GALSNVTHNGCGHYLHTLFWENMSPNGGGEPEGDLADRIEEDFGSYEGWKGEFEAAAG--AAGGWALLVYDPVAKQLRNLAVDKHDQGALWGAHPVLALDVWEHSYYYDYGPDRGSFIDAFFDVVNWEKAEEEYQTCLD

>A0A1I0EA93_1123402_Proteobacteria_B_Mar

MSYSLPELPYAYDALEPHFDKETMEIHHSKHHQAYVNNANNALADLPDLAKLSVEALIADLDKVPAEKRPVLRNNAGGHANHSLFWKGLKV--GTTLAGDLKAAIERDFGSVDAFKEKFEQAAATRFGSGWAWLVYQ-D-GKLAVVSTANQDSPLMGSGFPIIGLDVWEHAYYLKFQNRRPDYIKAFWNVVNWDEAANRFAQVK-

>A0A1I0M6P7_1202768_Halobacteria_A_Arc

ADHELPPLPYDYDALEPALSEQVLTWHHDTHHQGYVNGLNSAEETLAENREEGDY---GSTP----GALKDVTHNGCGHYLHTLFWENMSPNGGGEPEGDLADRIEEDFGSYEGWKGEFQKAAG--AAGGWALLVYDPVAKQLRNLAVDKHDQGALWGAHPVLALDVWEHSYYYDYGPDRGEFIDAFFDVVNWEKAEEEYQTCLD

>A0A1I0MG92_355548_Halobacteria_A_Arc

SDYELPPLPYDYDALEPHISEQVLTWHHDTHHQGYVNGWNAAEETLEENRESDDH---GSTA----GALGDVTHNGSGHILHTLFWQSMSPEGGDEPSGALADRIAEDFGSYDAWQAEFEAAAS--AAGGWALLVYDPHSEQLRNVAVDKHDQGALWGSQPILALDVWEHSYYYDYGPDRGSFVDAFFEVVDWEEPRDRFEQAVD

>A0A1I0MM22_355548_Halobacteria_A_Arc

SQHELPPLPYDYDALEPHISEQVLTWHHDTHHQGYVNGLNSAEETLAENRDSEDY---GSTA----GALGNVTHNGSGHYLHTLFWENMDPNGGGEPSGELADRIAEDFGSYEGWKGEFEAAAS--AAGGWALLVYDPVAKQLRNVAVDKHDQGALWGSHPIMALDVWEHSYYHDYGPDRGSFIDAFFEVVDWDEVAANYDDVVS

>A0A1I1DPZ5_34097_Spirochaetes_B_Mar

-MFTLPELGYSYDALEPYIDAKTMEIHHTKHHKGYIDNLNAALEKHPEWADKSLKEILANIESVPEDIRMAVRNNAGGHHNHTLFWKLLKKNNGAKPTGELLKLIERDFGSFEKFQEMFETAAKTRFGSGWAWLMMDKN-GKLSIISTPNQDSPIMDGFFPLLGLDVWEHAYYLKYQNRRPDYIKEFWNIIDWDIVSSRMNGSCG

>A0A1I1UAP9_385682_Bacteroidetes_B_Mar

MKFSLPQLPYALDALEPRISKRTLEFHYGKHHQAYVNNLNKLILGT-KFENADLETIIKEA-------EGGIYNNGAQVWNHTFYFSSLSPNGGGVPTGPLAEAINESFGSFDAFKEKFSEAAATLFGSGWAWLVKNDD-GKLEIVKESNAGNPLRQGKVPILTCDVWEHAYYLDYQNKRPDYIQVFWDCVDWNIVGGRF-----

>A0A1I1VUU8_385682_Bacteroidetes_B_Mar

SGHLFPELGYAYDALEPYIDAQTMELHYDKHHRGYYTKFMKAIEG-TGLTKTPMPQIFAKISQYS----DTIRNNGGGFYNHQLFWENMSPN-GGEPSARLLNALVKNFGSFDAFKEAFGNAAKSHFGSGWAWLYMDAE-KNLKITSTPNQDNPLMDRGIPLLALDVWEHAYYLKYQNKRGEYVDNFWKVVNWKTVSTRWELAIK

>A0A1I1XIF6_54_Proteobacteria_B_Mar

YA----PAEYKASHLRG-ITDEQIEVHL-KLYGGYVNRTNALFAKTAGLSNEGKT-GDSSFQ----ELKRRLGWEWNGMRLHEYYFDGLTPKAGLRADNPFAKAVTAQFGSIEAWRADLAGVAK-MPGVGWALTYLDPNNGQIWNHWVEDHQDGHPASGRLLLALDVWEHAFAVYRPPERAAYIEDFFANVDWDVVAKRLA----

>A0A1I1YWR4_385682_Bacteroidetes_B_Mar

MAFELPKLDYAYNALEPYIDAQTMEIHHTKHHGAYTSKLNAAIEG-TDLANKSIEEILANVSQHS----AAVRNNGGGYYNHNLFWKIMTPNGGGKPSGQLLEAIEKDFGSFEQFKETFNNAAASRFGSGWAWLVKQEN-GKLVVGSTPNQDNPLMDKGTPILGLDVWEHAYYLKYQNKRPEYIDAFWNVVNWEEVAKRFSA---

>A0A1I2A523_54_Proteobacteria_B_Mar

MPFTLPELPYAKDALAPHISAETLEYHHGKHHAAYVNNLNKLLDGK-PEAELSLEEIILRS-------DGGVFNNAAQVWNHTFYWHSMRPNGGGRPTGELAEAIARDFGSFERFREEFANAATTQFGSGWAWLVVQ-N-GKLAVTKTGNADLPLKHGQTALLTIDVWEHAYYIDYRNARPKYIDTFLHLVNWDFVAGNLRGR--

>A0A1I2GPG4_1003_Bacteroidetes_B_Mar

KEFSLLPLPYPTEALEPHIDKMTMEIHYGRHHATYVQKLNEAVQN-TTFFGKKIEEILPAITE----KDAAIRNNGGGHYNHSLFWSILSPKS-TAPSGKLAEAINATFGSLDSFKTNFSDAAKAVFGSGWTWLCVGKD-KKLFISNTPNQDNPLMSVGTPILGLDVWEHAYYLKYQNKRADYINAFWQIVNWDEVAKRFASVS-

>A0A1I2HT21_1003_Bacteroidetes_B_Mar

MAFELPALPYDKAALEPHIDAMTMEIHHGRHHNTYVTNLNNAVKG-TEMENMSIEDLMKNISKYP----VAVRNNGGGHYNHSLFWQIMSPNGGGEPTGDLAAAINAKFGSYASFKEEFAKAAVGRFGSGWAWLIVD-N-GELKICSTPNQDNPLMDKGTPILGLDVWEHAYYLKYQNKRPDYITAFWNVVNWAEVAKRYAQAK-

>A0A1I2LRE0_1855325_Bacteroidetes_B_Mar

MNIPMPVLPYAPNALEPVISEQTISFHYGKHLQGYVNTINKLVEGT-EFAGKSLEDLVRTV------PEGPMYNNAGQALNHKFYFLQFSPRKDNVPTGRIAAAINSDFGSFEAFKKQFAQAATSLFGSGWAWLSQDES-GKLVITKEANGGNPLRSGLNPLMGIDVWEHAYYLDYQNRRADHVEAVWEIIDWKVVESRLK----

>A0A1I2QXX4_553467_Halobacteria_A_Arc

STYELPDLPYDYDALEPVIDERIMELHHDKHHQGYVDGANSALEQLEQMRESGDM---GNVK----SVKRNLAFNLSGHVNHTVFWENMSPDGGGEPEGELADALDEQFGGFEQFKSDFAAASKGVEGSGWGQLVYDHAADQLMVVANENHQNQQVSGSTPILVLDVWEHAYYLQYENNRGEYVDSFWDVVDWEDVAQRYEQAQS

>A0A1I4SFU3_1166257_Proteobacteria_B_Mar

MAFELNDLPYAYDALGPYMSRETLEYHHDKHHLAYVNNGNNLIKGT-EWEGKSIEEVVKGSFG----KNPALFNNVAQHYNHFYFWQWMKPNGGGSIPGELLNKINEDLGGLDKFRADFIQAGMTQFGSGWAWLSLK-D-GKLAISKTPNGENPLVSGATPLLGVDVWEHSYYIDYRNLRQKYLEAFFNLVNWEFVAELYSKAG-

>A0A1I5ZIY7_1227077_Bacteroidetes_B_Mar

MAFELPKLPYAYDALEPTFDAQTMEIHHSKHHQAYVTNLNAAITG-TEMENQSIEEIMHNIAKAP----APVRNNGGGHFNHSLFWTILSPNGGGQPSGAIGEAITSAFGSYDKFKEEFTKAATTRFGSGWAWLCKQAD-GSVQICSTPNQDNPLMPKGLPVLGLDVWEHAYYLKYQNRRPDYIAAFYNLINWDEVNKRFAEATP

>A0A1I6K766_767519_Halobacteria_A_Arc

SNPELPELPYDYDALEPHISEQVLTWHHDTHHQGYVNGLQSAEETLAENRSSGDF---GGSA----GALGNVTHNGSGHYLHTLFWENMSPNGGGEPEGDLRSRIEEDFGSYEGWKGEFEAAAG--AAGGWALLVYDPVAKQLRNVAVDKHDQGALWGSHPILAIDVWEHSYYYDYGPDRGSFVDAFFEVVDWEKAAEEYQKVVD

>A0A1I6LVZ5_767519_Halobacteria_A_Arc

SEPELPPLPYDYDALEPHISEQVLTWHHDTHHQGYVNGLQAAEETLAENRSSGDF---GGSA----GALGNVTHNGCGHYLHTLFWENMSPNGGGEPEGDLADRIAEDFGSYEGWKGEFEAAAG--AAGGWALLVYDPVAKQLRNVAVDKHDQGALWGAHPILALDVWEHSYYYDYGPDRGSFIDAFFEVVEWDKAAEEYQKCLD

>A0A1J0A9M2_1188229_Cyanobacteria_B_Ter

MSYEFPELPYAPDALAPYLTAEIFSYHYGKHHAAYVTNYNKLVKDT-EYADLPIETVVKKTYG--DPSQAAIFNNGAQAWNHTFYWNCMKPGGGGTPTGALAEKITADFGSFDQFKTAFKQAGVTQFGSGWAWLVLD-N-GTLKVTKTLNAENPFCFNQVPLLTMDVWEHAYYLVYQNRRPDYAQDFIHLINWDFVSTQYAAA--

>A0A1J0ADU4_1188229_Cyanobacteria_B_Ter

SAFTVPPLPYAYDALEPIIDRRTMTFHHDKHHRAYVNSLNGAIAKYPELAGQSIEQLLQNLAKLPSDIQTAVRNHGGGHYNHSLFWESMRPPQASAPTGALGLALAQNFGDFQTFQEQFAQAGMRVFGSGWVWLVGDKT-GQLQVMTTPNQDSPISVGLTPLLGNDVWEHAYYLNYQNRRADYLNAWWPVVNWPVVAQRYAALLG

>A0A1J4QJC3_1414654_Proteobacteria_B_Mar

MAFELPALPYAKNALEPHISQETLEFHHGKHHNSYVVNLNNLVPGT-EFEGKSLEDIIKTS-------SGGIFNNAAQIWNHTFYWNCLAPNGGGEPTGALADAIKAAFGSFDAFKEEFTKSCVTNFGSGWTWLVKKAD-GSVAIANTSNAGCPLTDNATPLLTCDVWEHAYYIDYRNVRPDYVKAFWALVNWEFVAENFAK---

>A0A1K1LIX2_1855339_Proteobacteria_B_Mar

ITHVLPPLPYADDALDPVISAHTLSFHYGKHHKAYVDNLNKLVAGT-ELADRSLEEIIAATAG--QADKAGVFNNAAQIWNHTFYWNSLKPGGGGQPPAALRERIEASFGSVDACKQELAAAATTQFGSGWAWLVRD-G-DTLKVVKTGNADVPLTKGLTPLLTLDVWEHAYYLDYQNRRADYVNAVLKLINWGFAADNHA----

>A0A1L4CZX9_1915309_Proteobacteria_B_Mar

MAKEIS------KHLLGKISDPQLEAHFGL-YEGYVKKLNEIEEKLEKTDKGLTNYSFGEFS----ELKRRHCVPYNGTYLHEMYFENLIST--ESPSPQFENLAKAAFGSVDNWKADVKATGL--AVPGWVVTCVETTTGKLKNVQIMEHHIGFPLNHVPVLVMDTWEHAFFLDFKANRGAYIDVFFKNINWSVVNARVTQCAK

>A0A1L6KZF6_888845_Proteobacteria_B_Mar

MAFTLPELPYSRDALAPHISAETLDFHHGKHHNAYVTKLNELVAAP-SLAGKSLEDLIRTT-------SGGVFNQAAQVWNHTFYWHSMKPQGGGEPTGALLAAIEKTFGSVATFREKFNAAAVGQFGSGWAWLVKNAA-GNLEIVQTSNAGCPLTEGKTPLLTCDVWEHAYYIDYRNARAKYVETWWNLVNWDHAASKL-----

>A0A1L6LK10_888845_Proteobacteria_B_Mar

-------MARDADHLLGGLSDQQLKAHF-TLYQGYVKKLNEIREKLGTADRSAPNYSFNEYS----ELKRREPVAYNGTVLHEMYFENLGN-GSTQPNEVTKRLITDSFGSFDNWVTDAKACLL--SAHGWLLTIFDYADGKLHNNLIRSHDVGLFANVHAMIAVDAWEHAYFFDYQTAKAKYVDNALSGLNWDVLNKRIGMVSG

>A0A1L8ZD32_64897_Spirochaetes_B_Mar

--FKLPELGYDYDAVEPYIDAKTMEIHHSKHHNGFVMNLNSILEKMDKIHLTDVSNILKNIHDFPEEFQTLIRNNAGGYSNHTLYFRTLRPGNKDNLFEKFKDDINAAFGSLDVLKANLKDTAMKIFGSGWAWLVLCPD-SGLKVISMPNQDSPLMKSYKPILGIDVWEHAYYLKYQNRKIEYVDAFLKALNWEEVSKIYNEVDN

>A0A1L8ZDB1_64897_Spirochaetes_B_Mar

--FKLPELGYDYDAIEPYIDAKTMEIHHSKHHNGFVMNLNSILEKMDKIHLTDVSNILKNIHDFPEEFQTLIRNNAGGYSNHTLYFRTLRPGNKDNLFEKFKDDINAAFGGLDVLKANLKDTAMKIFGSGWAWLVLCPD-SGLKVISMPNQDSPLMKSYKPILGIDVWEHAYYLKYQNRRIEYVDAFLKALNWEEVSKIYNEVDN

>A0A1M5PRJ6_490188_Proteobacteria_B_Mar

GQHTVRPLRFDPAKL---LSEKLIRSHHENNYAGSVKALNLIEGRLAAAMQDADFPPIV-YG----GLKREELHRVGSVVLHEHYFDNLG--GDGKAGGDVAKALSQVYGSVAAWEAEFRRTAMLAGGSGWCVLTFNAATGELRNQWASDHMHGAVAG-VPMLVLDMYEHSYHLDYGSAAARYLDAFLDNVDWQIVDRRFQLALH

>A0A1M5RQ54_490188_Proteobacteria_B_Mar

MALTLPELPYAKDALQPHMSAETLEFHYGKHHKAYVDNGNKLIAGT-EFENMSLEDIVKKS-------SGKIFNNAAQVWNHTFFWNCLTPNQGA-PGKKLTDALVKAFGGVEDFKKQFTETAIGTFGSGWAWLVKNAD-GTLAITSTSNAQTPLTEGKTPLLTCDVWEHAYYVDYRNARPNYVEHFWALVNWDFVEKNLG----

>A0A1M6L352_1830138_Firmicutes_B_Ter

MAFQLPPLPYAFNALEPHIDALTMEIHHDRHHGTYVNNLNAALEGHADLQSKSIEELLSHLDAVPENIRTAVRNNGGGHANHSMFWEILSPNGGGAPTGEIAKAIDETFGSFEKFQEEFTKAATGRFGSGWAWLVVD-G-GKLSIMSTPNQDNPMMEGKKPVLGLDVWEHAYYLKYQNKRPDYIKAFWNLINWAEVNKRFEAAK-

>A0A1N6WYU5_553468_Halobacteria_A_Arc

SNAELPPLPYDYDALEPHISEQVLEWHHDTHHQGYVNGLNSAEETLAENRESGDY---SSTA----GALGNVTHNGCGHYLHTLFWDNMDPNGGGEPEGDLADRIEEDFGSYEGWKGEFEAAAS--AAGGWALLVYDPVAKQLRNVKVDKHDQGALWGSHPILALDVWEHSYYYDYGPDRGDFISNFFEVVDWDEVADQYENVAP

>A0A1N6WZ27_553468_Halobacteria_A_Arc

ATYELPELPYDYDALEPTIDARIMELHHDIHHQGYVDGANAALDKLETMRDNDDW---GDVK----GVERNLAFNLSGHINHSVFWENMSPDGGGEPGGELADAMDEHFGGFDQFKSHFSAAAKGVEGSGWGMLVYDYVADKPIVTMAENHQNQSPM-AEPLLVLDVWEHAYYLQYENNRGEYVDNFWDVVNWDDVAERLDEAQS

>A0A1N7AFB9_588898_Halobacteria_A_Arc

MSYDLPDLPYEYDALEPHIDREIMELHHSGHHQAYVDGANDALEELEEMRDADDF---EGIK----AVKRDLAFNLSGHVNHAIFWENMAPDGGGEPTGTLAEAIERSFGSFEAFRAEFTQTAADVESVGWAMLFYEPLADELIIGQLESQHLLAHQDSTPLLTLDVWEHAYYLQYQNERDTYIDEWWNVVDWDDVAERYEAACA

>A0A1N7EDL3_588898_Halobacteria_A_Arc

TDHELPPLPYDYDALEPSISEQVVTWHHDTHHQGYVNGLNSAEETLADNRESGDF---DSTP----GALGNVTHNGCGHYLHTLFWENMSPNGGGEPEGDLADRIEEDFGSYEGWKGEFEAAAG--AAGGWALLVYDPVAKQLRNVAVDKHDQGALWGSHPILALDVWEHSYYYDYGPDRGEFIDGFFEVVNWDSAADEYQKCLD

>A0A1P8F872_1839801_Chloroflexi_B_Ter

--------MYTANHLKG-FSETLLNNHF-TLYQGYIKNTNKLFETLDALSKAGKS-DTPEFA----ELQRRFGFEWNGMRLHELYFGNLGGDGLLAPAVKLEKKLAEAWGSREAWEKDFRAVGS-LRGVGWAVLYQDPVSGALFNCWINLHESGHLAGCNPLLVMDVWEHAFMIDYGLKRADYIDAFFKNIDWAACEKRLK----

>A0A1P8FEK9_1904640_Proteobacteria_B_Mar

MRYHLTPIHCRPWLL---LSLKLIESHYENNYGGALRRLNAITAQLETLD-FAKTPPHV-VN----GLKRDELTALNSTLLHELYFASLG--GDGQPTPAMKEALARDFGSVDRWRDEFLAMAKLAGGSGWVVLSYIPRDRRLVNQYAAEHTQSVA-GGIPIFALDMYEHAYHIDFGANAQAYIEAFLRNADWKSVQVRYEDATK

>A0A1P8FPF1_1904640_Proteobacteria_B_Mar

GPFTLPSLPWDEAALDPVISARTMSFHYGKHHKAYVDKLNELAAGT-KYAEMPLEQVIVAAAK--DESAKKIFNNAAQIWNHTFFWNALKPGGGGKPNGEVAGAIDAAFGSPDAFKKKFAQAAVDQFGSGWAWLVVK-D-GKLSITSTSNAGTPITDGITPLLTIDVWEHAYYLDYQNKRPDFANAVIKLVNWDFANLQFKTAAQ

>A0A1Q1FM06_1932360_Halobacteria_A_Arc

SNPELPPLPYDYDALEPSISEQVLEWHHDTHHQGYVNGLESAEETLAENRENGDF---GSSG----SAIRNVTHNGSGHYLHTLFWENMDPNGGGEPSGELADRIEEDFGSYEGWKGEFEAAAS--AAGGWALLVYDPVADQLRNLVVDKHDQGALWGSHPILALDVWEHSYYYDYGPDRGSFIDGFFDVVDWDNVAEQYEKALS

>A0A1R1I0D0_418702_Proteobacteria_B_Mar

MEHQLPALPFAKDALAPHMSAETFDYHYSKHHNAYVVNLNNLIKGT-EYEALDLEAIVKKAP------AGGIYNNAAQVWNHTFFWNCLTPNGGGAPSGALADAINAKWGSLDAFKTAFQTSAVGNFGSGWTWLVKKAD-GSVDIVNMGAAGTPLTTGDKALLCVDVWEHAYYIDYRNLRPKFVETFLNLVNWSFAEANFAG---

>A0A1R3VN56_233100_Proteobacteria_B_Mar

MSIELPPLPYAKNALEPHISAETLEYHHDKHHATYVANLNKMIAGT-EFENMGLDDIVKKAP------AGGMFNNAAQIWNHTFYFEGLTPNGGGEPSGALADAIDKAFGSFEGFKEKFTAAGAGNFGSGWTWLVKKAD-GSVEIVNTDDAETPITDGVTPLLTMDVWEHAYYVDYRNARPKYIENFFNVVNWDFVAKNYGA---

>A0A1R4H9Z6_360316_Proteobacteria_B_Mar

TTIVLPPLPYLETALEPVISAKTVGFHYGKHHAGYITNLNNFIAGT-KYVGMSLEKIIKATAG--KRLDKMIFNNAAQTWNHTFYWNSLKPGAGDKPTGQLLKLIKRDFGHFSDFKQKYFDAAKGLFGSGWAWVIINKK-GKLEILTTSNADVPFTDGLRPLCVIDVWEHAYYLDYQNVRINYLNGVLNLINWDFAAQNIKRKY-

>A0A1R4HH45_360316_Proteobacteria_B_Mar

STIILPALPYAENALEPYISAKTVSIHYNKHHAGYVANLRSLIAGT-PYDVMPLKEIIKRTAG--KSLPKLIFNNAAQAWNHSFYWDSLKPGAGDKPSGYLLKLIRRDFGHFSNFKQKLFDAAKGHFGSGWVWVVLNKQ-RKLEIITTSNADVPFTQGLKPLCVIDVWEHAYYLDHQNKRVDYLNGVLNLINWDFAAKNSLAKM-

>A0A1R4HJV0_360316_Proteobacteria_B_Mar

MAFELPALPYAKNSLAPHISEETLDFHHGKHHQTYVTNLNNLVPGT-EFEGLSLEEIIAKS-------SGGIFNNAAQIWNHTFYWNCLSPNGGGEPTGALADAINAAFGSFAKFKEEFTKCSVTTFGAGWGWLVKNAD-GSLALVSTSNAGCPLTTGQTPLLTCDVWEHAYYIDFRNARPAYLEAFWALVNWDFAAKNFAA---

>A0A1R4IAU7_1434842_Bacteroidetes_B_Mar

FKFEIAKLNYPYSDLEPVIDAATMEIHYERHYAAYVKNANDAIIA-DNVSASDAKDLFSRISR----YSTKLRNNAGGAFNHELFWSILKPTQNNLPAGQLAQAISSNFGSLESFKEEFTKVALGQFGSGWAWLVKD-G-NRLKIGGTPNQDNPLMDKGTPILGLDVWEHAYYLNYQNKRADYVDKWWEIVNWEHVAQLYRTSK-

>A0A1R4KBB1_1434842_Bacteroidetes_B_Mar

ITQVLPELPYDKNALNPIITEETFDYHYGKHHATYVNNLSGLVKDT-PLETAKVEEIIRKGFS--E-NNAALFNNAAQHWNHTFFWHCLSPNGGKAPQGKIAELINRDFGSFENFKEQFSSTAVKLFGCGWAWLAQDEN-QKLEIVPMKDAHTPLTANKTPILTLDVWEHAYYIDYRNARPKFVEGFWEIINWDFANNNLK----

>A0A1R4KQ40_1434842_Bacteroidetes_B_Mar

ITQALPELPYDKNALNPIITEETFDYHYGKHHATYVNNLSGLVKDT-PLESASIEEIIQKGFA--E-NNAPLFNNAAQHWNHSFFWHCLSPNGGKAPDGKIAEFITRDFGSFETFKNTFSETAVKLFGCGWAWLAQNEQ-GILEIVPMKDAHTPLTENKKPILTLDVWEHAYYIDYRNARPKFVEGFWEIVNWDFANQNLK----

>A0A1R4L182_1434842_Bacteroidetes_B_Mar

MAFELEALPYAADALEPHIDKDTMEIHHDRHHQAYVDNLNKAIAG-TDAEGLSLEEINKNISKYA----AGVRNNGGGHFNHKLFWSVLGPNAGGEPTGELAEAINATFGSFSELKTQLQNAGATRFGSGWSWLIVKAD-GQLAVTSTPNQDNPLMDQGTPILGIDVWEHAYYLKYQNKRPAYLEAVFNVVNWGAVAERFQAAK-

>A0A1T2KSF0_1918948_Proteobacteria_B_Mar

MAHELPALPYDMNALEPTISQETLEFHYGKHHQTYATNLNNLVPGT-EFEDASLDEIVMKS-------SGGIFNNAAQVWNHTFYWNCLSPNGGGAPSGTLAVAINSAFGSFEEFKKQFAQSAATNFGSGWTWLVKNGD-GSVEIVNTSNAANPMTDGKTPLLTIDVWEHAYYIDYRNARPKYLEEIWNIVNWDFVGSNYDA---

>A0A1T2L771_1918949_Proteobacteria_B_Mar

MQHQLPELPYEKSALEPHISAETLEFHHDKHHATYVTNLNNLINGT-EFADKSLEEIVTSAP------AGGIFNNAAQVWNHTFYFNCMAPGAGGAPTGEIAAAIDAAFGSFEDFKSAFSTSAVTNFGSGWTWLVKNAE-GGVEIFNTSNAGCPLTEGLTPLLTIDVWEHAYYVDKRNARPAYVESWWTLVNWDFVNAQL-----

>A0A1T4W9E9_1121442_Proteobacteria_B_Mar

AAPKAPQLPYAKGSLAPAISQKTLDFHYGKHTLGYYSKLAALISGT-KYAKMPLEDIIRSTAS--NKSTRSIYNNAAQAWNHTFYWQGLKAKGGGMPPDAFGADLMETYSTLAHFQDVFIATAASVFGSGWAWIVKDKA-GKIDIIGTQGADTPLTMNKTPLFVVDVWEHAYYLDYQNRRSDYVQAFYYLANWDVVSKRYNAK--

>A0A1U7EY38_348780_Halobacteria_A_Arc

SNPELPPLPYDYDALEPSISEQVVTWHHDTHHQGYVNGLESAEETLAENREAGEF---GGSA----AAIRNVTHNGSGHYLHTLFWENMDPNGGGEPSGDLADRIEEDFGSYEGWKGEFKAAAG--AAGGWALLVYDPVADQLRNLVVDKHDQGALWGSHPILALDVWEHSYYYDYGPDRGEFIDGFFEVVDWDNVAEQYEKATQ

>A0A1U9JL93_1938604_Proteobacteria_B_Mar

MEHKLPELPYALDALAPTISKETLEFHYGKHHQTYVTNLNNLIKGT-EFENASLEEIVKKS-------SGGIFNNAAQVWNHTFYWFGLSPNGGGEPTGALADAITAKWGSFDEFKKAFNAVAAGTFGSGWAWLVKAAD-GSLELVSTSNAATPLTGDKTPLLTCDVWEHAYYIDYRNSRPNYLEGFWKLVNWDVVAKNFA----

>A0A1U9JNI9_1938604_Proteobacteria_B_Mar

AQFSLPPLPYAADALEPVIDKATMEIHHDRHHAAYVNNLNAQIKTYPELDTLSLEALQGKISQY----SPAVRNNGGGHYNHSLFWNVMAPGQGGQPSAALMQAINSQFGSLDELKKRFDQAAVSRFGSGWAWLMVTPD-KKLAISSTANQDNPLMDRGTPILALDVWEHAYYLKYQNKRADYVTQWWQVVNWQEVSRRYATALK

>A0A1V0RDW6_28898_Proteobacteria_B_Mar

--FELRKLPYNTNAFGDFLSAETFSYHHGKHHQTYVNNLNNLIQNT-EFAGKDLVSIIKNS-------SGGIFNNAAQVYNHDFYFDCISPSQSTCECPNLKAALEKNFGSLESFKDEFIKGATGVFGSGWFWLVLDSKTQKLEFVGTSNAATPICDDKIPLLVVDVWEHAYYVDHRNARPAYLEKFYAHINWAFVAKAYEWALK

>A0A1V1PIR0_1605283_Proteobacteria_B_Mar

--IQLPRLPWAPSSLEPAISARTIEAHYGKHHKGYVDKLNKLIIGT-DYDALTLEQIILETAGDRSKAGRAIFNNAAQIWNHTRYWDSLSPEGG-APDDALAAQIETDLGGLDKLKDELVRKGVDHFASGWVWLAWS-S-DKLQVIDTHDADNALVHGYDALLVLDLWEHAYYLDYQQERERHLRALVDMLNWKGANARFAKAR-

>A0A1V1PJD5_1605283_Proteobacteria_B_Mar

MRYEIAPLFCRPWTL---ITPRLIESHYENNYGAAVKRLNAIWTELDGPA-LNVASVQM-IH----RLKQEEASALNSALLHELYFASLG--GDGRP--TLAEALSRDFGSVDLWRNQFVALAELAGGSGWVLLAWLPRLGRLINQSVTDTSPPIA-GAIPILALDMFEHAFHIDFGPNASAYVAAFMRNIEWNAVRERYEDALS

>A0A1V3PIV2_1945854_Proteobacteria_B_Mar

MAIELPPLPYEKNALEPHISAETLEYHYGKHHQAYVTNLNKLIEGT-EFADAALEDIIRKS-------SGGVFNNAAQIWNHTFYWNSLGPKGGKEPNGKLADAISKAFGSFEKFKEEFTKSAAGNFGSGWTWLVQRPD-GALGIVNTSNAATPITGSDKPLLNVDVWEHAYYIDYRNARPKYLEAFWNLVNWEFAAKNLA----

>A0A1V3TPU9_1924934_Proteobacteria_B_Mar

MAYTLPELGYAYDALEPHFDAKTMEIHHSKHHQAYINNANAALEAHPELLEQCPGKLISNLSQVPADKLAAVRNNVGGHVNHTLFWKGLKT--GTTLQGALKDAIVRDFSSVENFQAEFEKAAATRFGSGWAWLVAN-E-GKLSVVSTANQDSPLMGAGYPILGLDVWEHAYYLNYQNRRPDYIKAFWNVVNWDEAARRFEEKVA

>A0A1V4C128_1853690_Halobacteria_A_Arc

SDYELPPLPYEYDALEPHISEQVLTWHHDTHHQGYVNGWNSAEETLAENRENGEF---GDSP----GAIGDVTHNGSGHILHDLFWQSMSPEGGDEPSGALADRIAEDFGSYDAWKGEFEAAAG--AAGGWALLVYDSYSNQLRNVVVDKHDQGALWGSHPILALDVWEHSYYYDYGPARGDFIDAFFEVVDWEEPAARYEQAVE

>A0A1V4C962_1853690_Halobacteria_A_Arc

TDYELPPLPYEYDALEPHISEQVLTWHHDTHHQGYVNGWNSAEETLAENRENGEF---GDSP----GAIRDVTHNGSGHVLHDLFWQSMSPEGGDEPSGALADRIAEDFGSYDAWKGEFEAAAG--AAGGWALLVYDSYSNQLRNVVVDKHDQGALWGSHPILALDVWEHSYYYDYGPARGDFIDAFFEVVDWEEPAARYEQAVE

>A0A1Y0FVA0_1987723_Proteobacteria_B_Mar

MAFELPALPYAKDALAPHISAETLEYHYGKHHKTYVDKLNGLVPGT-EYEGKSLEDIIKTS-------SGGVFNNAAQIWNHTFYWNCLSPNGGGEATGAVAEAINKAFGSFDKFKEEFTNSAINNFGSAWTWLVKKAD-GSVAIVNTSNAQTPLTDSVKAILTVDVWEHAYYIDYRNSRPNYLNAFWSLVNWEFVNAKYAE---

>A0A1Y0RQE1_1940762_Cyanobacteria_B_Ter

GAIQLPPLPYPYQALEPHIDAATMRFHHDKHHATYVKNLNAALDKHPELKSKTVEQLLRDLNSVPEDIRRTVRNNGGGHENHSMFWRIMKPKGGGEPTGAIASAIKQNFGNFAAFKKQFNEAGASRFGSGWVWLVRTKD-GKLAVTTTANQDSPFSEGNYPIMGNDVWEHAYYLKYQNRRADYLDAWWNVLNWDEINKRFAQASK

>A0A1Y0RVB5_1940762_Cyanobacteria_B_Ter

MAFELPPLPYDYNALEPYISARTLEFHHDKHHAGYVNNLNNLVKDT-ELANKSLEEVILATAN--DPAKAGIFNNAAQVWNHTFYWHCLKKD-GGSPTGELAEKINASFGSLDEFKKQFKEAGATQFGSGYAWLVVD-K-GELKVVKTLNAANPITNGQTPLLTSDVWEHAYYLDYQNRRPDYLQSVLNLINWDFVAEQYAKAAK

>A0A1Y1CGP3_1717717_Bacteroidetes_B_Mar

MIHELPQLPYALNELEPSISQRTLEFHYGKHHQAYVTNLNNLIKGT-AFEKASLDEIVRHS-------EGGIFNNGAQVYNHTFYFMALSPNGGGEPKGKLADALTETFGSLAAFKEEFSKAGATLFGSGWVWLVADSH-NKLRIVKKNNAGNPLTDGMTPILTMDVWEHAYYLDTQNARPKYIENFWNLVNWDVIEKRFEE---

>A0A1Y1CJJ9_1717717_Bacteroidetes_B_Mar

MAFELPKLQYAYDALEPHIDARTMEIHHSKHHAGYTNNLNVAIAG-TDIEGQSIENILANISKQS----VAVRNNGGGFFNHDLFWNVMSPNGGGNPSGSLLAAIEKDFGSFESFKEEFSKAAATRFGSGWAWLVKQSN-GKLVVSSTPNQDNPLMDKGTPILGLDVWEHAYYLKFQNKRPDYISEFWNVVNWEEVAKRFQD---

>A0A1Y2K534_1434232_Proteobacteria_B_Mar

MAFELPALPYAKDALEPHISKETLEYHYGKHHQTYVTNLNGLIEGG-PDADKSLEEIIHSA-------QGGLFNNAAQVWNHTFYWNCLSPHGGGAPGGALADAINAKWGSFDAFKEAFTKCAVTTFGSGWAWLVKKPD-GTLDLLSTSNACCPLTGDAKPLLTCDVWEHAYYVDYRNARPKYVEAFWNVVNWAFVADNFNG---

>A0A1Y3VAM6_1965650_Bacteroidetes_B_Mar

----MPELPYAPETLAPKMSRETLDYHYGKHLQTYVDNLNRLIPGT-PYAEMPLDEIVRRA-------DGAVFNNAAQAWNHTFFFRSLTPTQSAMPE-TLAAKLAAAFGSVEAFREQFTKAAVGLFGSGWVWLAADRS-GNLSIVAKPNAGNPMTDGLRPVLTVDVWEHAYYIDYRNRRADFVAAWWDLVDWKQVAERCIPRRW

>A0A1Y3VCW3_1965650_Bacteroidetes_B_Mar

SRFEPRELPYDYGALAPYLSEETMRFHHDKHYASYVAKLNELLLDS-PLAGQPLEDILLAA-------DGPLYNNAAQAWNHAFFFEQLSPQPQREPSPELRAAIDRDFGSLDALVTRMNQAAAGLFGSGWVWLAADRQ-GHLSILAEPNAGNPLRKGLIPLLGIDVWEHAYYIDYRNRRADAVAALWNVVDWRCVSDRYARR--

>A0A1Y4C9N7_1965623_Bacteroidetes_B_Mar

MKHLMPELPYGKEAFAPVLSEESFDYHYGKHLQTYIDTLNKLIEGT-GYENMSMEEIIMKA-------DGPVYNNAAQAWNHTLFFLTLTPAKKTMPE-KLSSLVRRDFGSEEQFRDQFIAKATAQFGSGWGWLSVDKE-GKLVITTESNAGNPLRGGLRPIMTLDVWEHAYYIDYRNRRADYLKAVWEHIDWDKVAERL-----

>A0A1Y5TAA6_745714_Proteobacteria_B_Mar

MTIELPNLPYDKAALEPHISARTLEFHHGKHHQTYVTNLNKMIEGT-DLAGASLEAIIKASAG--KADKAGIFNNAAQVWNHTFYWNSMAPKGGGKPTGAIAAKIDSDLGGYDAFAEAFKNAGATQFGSGWAWLVLD-G-GKLKVTKTPNADLPLAHGQTAILTADVWEHAYYLDYQNRRPDYLATFLSLVNWDFANANLAAA--

>A0A1Y5U185_745714_Proteobacteria_B_Mar

MTFELPDLPYSKDALAPHMSAETLSFHHDKHQKSYVEKLNSAVNGT-EFAGKSLEEIIRATAG--KSGESGIFNNAAQTWNHTFFWHSMSPKGGGAPDGALARQIDRDFGGFDKFRSEFLEKGKGQFGSGWVWLVLA-N-GKLEVVSTGNADTPIAHDSQPLLTCDVWEHAYYLDYQNRRPDYLESFLNLANWAFAAEEFANQGE

>A0A1Y6CDA5_1513793_Proteobacteria_B_Mar

MAFTLPELPYPKDALSPHISAETLDFHHGKHHNAYVTKLNGLVDGT-PNAEKSLEDIIKSS-------EGGLFNNAAQVWNHTFYWNCLSPNGGGEPSGAVADAINKNFGSFAEFKEKFTNAALTQFGSGWAWLVKKSD-GGLDIMQTANAGCPITENVTPLLTCDVWEHAYYVDYRNARPKYVDAFWSLVNWDFVASQMS----

>A0A1Z4UU62_1973480_Cyanobacteria_B_Ter

MAFTQLPLPYDFNALEQYMKGETFEYHYGKHHKAYVDNLNKLTDGT-ELADKSLEEVIQISFK--DPAKAGIFNNAAQVWNHTFFWNSLTPAGGGAPTGELAERINKDFGSFDKFKEEFSTAAATQFGSGWAWLVDD-G-GTLKVMKTPNAENPLAHGKKALLTIDVWEHAYYIDFRNARPAFIKNFLNLVNWEFAASNLAA---

>A0A1Z4UUG6_1973480_Cyanobacteria_B_Ter

STIELPPLPYAYDALEPHIDARTMQFHHDKHHAAYVNNLNKALDKYPELKNKTVEQLLQNLDNVPADIRTTVRNNGGGHVNHSMFWKIMKPNGGGEPTGEIATAINDNFGSFADFKKQFNEAGAGRFGSGWVWLVRNKN-GKLEITTTANQDSPLMEGKYPIFGNDVWEHAYYLKYQNRRPEYLDAWWNVVNWDEINQRFADASK

>A0A1Z4VNQ5_585455_Proteobacteria_B_Mar

MTHTLPDLPYPGNALEPHISAETLEYHHGKHHATYVANLNKLIEGT-EYADKALEDIIRSAP------AGGLFNNAAQVWNHTFYWNCMSPNGGGEPAGALADAIKAAFGSFADFKEKFSTSGATNFGSGWTWLVQKSD-GSVDIMNTSNAGCPLTEGVTPLLTLDVWEHAYYIDYRNARPKYIEAFWNVVNWDFVAGNFQG---

>A0A1Z5HC40_113268_Proteobacteria_B_Mar

MAYELPALPYAKDALEPHISEETLEFHYGKHHQTYVTNLNNLVPGS-EFEGASVENLMMRA-------SGGIFNNAAQVWNHTFYWHSLSPNGGGEPTGALADGINAAFGSFAEFKEAFTKCAVTTFGSGWAWLVKNAD-GSLALVSTSNAACPLTEGQTPLLTCDVWEHAYYIDYRNARPKYLEAYWALVNWEFAAENYAG---

>A0A202E7D5_253108_Halobacteria_A_Arc

ADHELPPLPYDYDALEPSISEQVVTWHHDTHHQGYVNGLNAAEETLEENRESGDY---GSTA----GALGNVTHNGSGHYLHTLFWENMSPNGGGEPAGDIADRIEEDFGSYEGWKGEFEAAAS--AAGGWALLVYDPVSKQLRNLAVDKHDQGALWGSHPILALDVWEHSYYYDYGPDRGEFIDGFFDVINWDSVDEEFQKCLD

>A0A212PXD1_877466_Chloroflexi_B_Ter

MAHTLPPLPYPYDALEPYIDAQTMEIHHTRHHQAYVNNLNAALEKYPELQDVPVETLLRHLPSVPEDIREAVRNNGGGHLNHSLFWTLMKPGGGGEPSGALAEAIAKAFGSFANFKELFTKAAMGRFGSGWAWLSMTAF-GKLVVHSTPNQDNPVMEGLFPLLGLDVWEHAYYLKYQNRRADYVQAWWNVVNWDQVAANYAKALE

>A0A218ZVX8_1961136_Thermoplasmata_A_Arc

MAEKWE----AKDKLKPKISDQQIEYHFETHYKGYVNKLNEIWEKLSTADRSKANQNYSEFR----ELKLEETFNFDGSLLHELYFDNLSKG-QGQIPEDLRKHLEKDFGSYEKWVEDFKATGV--AFRGWSLLVYDLNTGKLRNIGADVHNTNGIWNAIVLMALDVYEHAYYTDYGPKRAPYLDAFMKNVNWPAVSKRYEKAKK

>A0A231UUB0_1876515_Proteobacteria_B_Mar

MAFSLPDLPYAYDALAPHMSAETLEYHHDKHHNAYLETMNGIIEGT-DYESMELEEIVKKSHK----DDSKLFNQSAQFYNHVHFWKWMKPEGGGQLPEKLQQAFESDLGGYDKFREDFINAGKTQFGSGWAWVFVK-D-GKLHIDNGLNGENPLIMGGTPILGVDVWEHSYYIDYRNARPKYLEAFVHLINWDYVLERYEVATS

>A0A238VTU4_63740_Halobacteria_A_Arc

MSYELDPLPYDYDALEPHISEQVLTWHHDTHHQSYVNGWNAAEETLAANREEGDF---SSSG----GALRSVTHNSSGHILHDLFWQNMSPEGGDEPEGALADRIEEDFGSYEAWKGEFKAAGG--AASGWALLVYDTFSNQLRNVVVDKHDQGAIWGGHPILALDVWEHSYYHDYGPARGEFIDNFFEVVDWEEPSARYEQAVE

>A0A242PAB3_1970738_Proteobacteria_B_Mar

MAYTLPPLPYAYDALEPYIDTETMHLHHDKHHQTYVNNANALLESLPEVKDFCPETLLKNLDKVPADKVTGVKNNVGGHVNHTLFWELLKV--GTELKGELKAAIEKDFGSVDAFKEKFSAAAATRFGSGWAWLVLK-N-DKLEVVSTANQDSPVMGSGTPILALDVWEHAYYLKYQNRRADYIGAFWNVINWDKVAELYAEAKK

>A0A250KTZ9_1432792_Proteobacteria_B_Mar

MTHELPPLPYAKNALEPHISAETLEYHYGKHHQTYVTNLNNLIAGT-DFEKLPLEDIIKKS-------SGGIFNNAAQVWNHTFYWNSLKPNGGGAPAGALADAINQAFGSFDKFKEEFTKCAIGTFGSGWAWLVKNAD-GKLAIVSTSNAGNPMTSDQTPLLTCDVWEHAYYIDYRNARPKYVEAFWNLVNWDFASKNFGA---

>A0A250KYU0_1432792_Proteobacteria_B_Mar

AALTLPPLPYRENALAPVISARTLGFHYGKHHKGYVDNLNKLVAGT-ELADLPLEEIIAATAD--KPDRKAIFNNAAQAWNHTFYWRSLRPKGGGTPPAALRQKIEQSFGSVDACKKELADAAVTQFGSGWGWLVQD-G-DNLRVVKTSNANQPTPSTLKPLLTIDVWEHAYYLDYQNRRADYVNALLKLINWEFAAENLR----

>A0A251X438_1570016_Proteobacteria_B_Mar

MAHQLPALPYAMDALAPHISKETLEFHYGKHHQTYVDKLNALIPNT-EFTDLSLEEIVKKS-------SGGIFNNAAQVWNHTFYWNCLSPNGGGAPTGAIAEAINAKFGSFDKFKEEFTATAINTFGSGWGWLVKNPD-GSLALMSTSNAGTPMTEGKTALLTCDVWEHAYYVDYRNARPKYVEAFWNLVNWDFVNANLAA---

>A0A256FXP0_571255_Proteobacteria_B_Mar

MAFELPPLPYDYDALAPFMSRETLEFHHDKHHQAYVTNGNKLLEGS-GLEGKSLEEIVKESFG----KNQALFNNAGQHYNHLHFWNWMKKDGGGKLPGNLEKAIESDLGGYDKFREDFIAAGAGQFGSGWAWLSVK-N-GKLEISKTANGESPLVHGATPILGVDVWEHSYYIDYRNARPKYLEAFVSLINWDYVSELYEKA--

>A0A256IHQ1_1383851_Halobacteria_A_Arc

MSYELDPLPYDYDALEPHISEQVLEWHHDTHHQGYVNGWNAAEETLEANREEGDF---SSSG----GAIRNVTHNSSGHILHDLFWQNMSPEGGDEPEGDLADRIEEDFGSYEAWKGEFEAAAS--AASGWALLVYDTFSNQLRNVVVDKHDQGAIWGGHPILALDVWEHSYYHDYGPARGEFVDNFFEVVDWEEPSARYEQAVE

>A0A257EMF7_2015572_Proteobacteria_B_Mar

SPLEQPPLPFARDALAPAISADTIGVHYDKHHKGYFDTLVKLVAGS-PLAGQTLEQIIRATTD--DPAQVKIFNNAAQAWNHNFYWASLSPK-ALTPGGALAAAIARDFGDLAAVQAALIKAGTEQFGSGWAWLVAD-G-DTLKVVATHDAGVRFTSGQRPLLTVDVWEHAYYLDVQNRRADYLKAVVGHLNWDFAAANFAAD--

>A0A257J713_2015570_Proteobacteria_B_Mar

--FKLADLPYAHDALEPVISSRTLHFHHDKHHGGYIKTLNALLEGA-ETAPTVLEDVIREASM---SGDSKVFNSAAQAWNHSFFWASMTAE-PQRPDRDMDAAIRFGFGDLAGLKLAFVKEGAEHFGSGWVWLVADRS-GDLSVRSTHDADDTLGQALTPLLVCDLWEHAYYLDHQNDRKAYLEDWFALPNWNFAGEQLAAAKG

>A0A257J8A7_2015570_Proteobacteria_B_Mar

APPQPKPLPFDPNTI---ISEKLLTSHHGNNYVGAVKRLGSIETQLAGLD-MATAPGFL-LN----GLKREELIAWNSMILHELYFGGLG--PANRPGAALSRAIERDFGSEAKWRAEFSAMGKLGGGSGWVLLTYSHRDNRLMNQWAMDHTMTMA-GATPILAMDMYEHAYAMDYGAKAAAYVDAIMATFNWTRADAIFAKATA

>A0A257L3N9_2015582_Bacteroidetes_B_Mar

MSFELPKLTYEYTALEPHIDARTMEIHHSKHHNAYVTNLNAALAG-TEGENHSIEDICKNISKYP----AAVRNNGGGHFNHSLFWTIMGPNKGGQPTGKLMDAITNELGGWDKFKEDFNKAGATRFGSGWAWLCVGAD-KKLSVCSTANQDNPLMDKGTPILGCDVWEHAYYLHYQNRRPDYMAAFWNVINWDEVSRRYEAAL-

>A0A257L9C2_2015582_Bacteroidetes_B_Mar

GKYELPALGYAYNSLEPFIDAQTMEIHYSKHHQAYINKLNEAMDKEPSLKNKSIEELLLNLASVPASVKGAVRNHGGGHWNHSFFWKSLKT--GTSMGSEFTKLATASFGDIENFKTTFEKVSMGVFGSGWVWVILQ-N-KQLKIVTSANQDNPLMDMPKVILGIDLWEHAYYLKHQNKRIDYVTGFWNVVNWDEIEGLLK----

>A0A259TZ62_716817_Rhodothermaeota_B_Mar

MAFELPDLPYSYDALEPHIDEQTMRIHHGKHHAGYTTKLNAALEG-TEWADQPIEETLANLDALPEDKQTAVRNNGGGFLNHGIFWTTMSPSGGGEPTGDLAKAIDEAFGSFDAFKEKFSEAAKGQFGSGWAWLELASD-GSLQVHGHANQNNPVMHGAKPIIGLDVWEHAYYLKYQNKRPDYVSAWWNVVNWDEADRRYRSYNA

>A0A259UH52_1123289_Firmicutes_B_Ter

GKHRLPPLPYAYDALEPIISEDTLRIHHDHHHKAYVDGLNKAEVALAQARAGKDY---TFIK----YWENELAFNGSGHILHSIYWTILTQKGGGEPGPDTVREITRRFVSFDAFLEQLIAATIKVEASGWGILAWNPSWNYLEILIAEKHQNLTQWGSIPILVLDVWEHAYYLDYQYQREEYVRRILELINWNEVETRLDLAIR

>A0A285M297_1798205_Proteobacteria_B_Mar

MAFELPALPYAHAALAAKMSQETLELHHDKHHQAYVTALNNFIENA-DLQGKSLEDIIAATYG--NDERAGIFNQAGQHWNHIHFWNALSPNGGG-IPGSLEAKLVEAFGSVDQFKADFKTAATTQFGSGWAWLIQKAD-GSLSVTKTPNGVNPLATGETALLGLDVWEHSYYVDFRNRRPDYVSNFLNLANYEFAESNLA----

>A0A285MB93_1798205_Proteobacteria_B_Mar

MAFELPELPYSYDALGDFMSAETLEYHHDKHHLAYVTNGNNLLQDS-GLEDKSLEEIVKESYG----KNSGLFNNAAQHFNHVHFWKWMKPVAEESVPGALASKIESDLGGMDKFRADFINAGITQFGSGWAWLALV-D-GKLAVTKTPNGENPLVHGGVPLLGCDVWEHSYYIDYRNARPKYLEAWFNLVNWEYVAELLEAAS-

>A0A285N4Y7_558529_Halobacteria_A_Arc

SNPELPPLPYDYDALEPHISEQVLTWHHDTHHQGYVNGLESAEETLAENRESGDH---SSTA----GALGNVTHNGSGHYLHTLFWENMDPEGGDEPEGDLADRIEEDFGSYEGWEEEFRAAAS--AAGGWALLVYDPVAKQLRNVAVDKHDQGALWGSHPILALDVWEHSYYYDYGPDRGDFIDAFFEVVDFDKVAEEYEKVSG

>A0A285P0H4_558529_Halobacteria_A_Arc

SDYELPPLPYDYDALEPHISEQVLTWHHDTHHQGYVNGWNSAEETLAENREEGDF---SSSA----GAIRDVTHNGSGHVLHDLFWQSMSPEGGDAPEGDLADRIEEDFGSYEAWKGEFEAAAS--AAGGWALLVYDSHSNQLRNVVVDKHDQGALWGSHPILALDVWEHSYYHDYGPARGEFVDNFFEVVDWDEPSARYEQAVE

>A0A2A5LZ91_2039467_Proteobacteria_B_Mar

MAFELPALPYEKNALEPHISAETLEYHYGKHHNTYVTKLNGLVEGT-DNANKSLEEIIKSA-------SGPVFNNAAQVWNHTFYWHCLSPNGGGEPTGAAKEAIEKAFGSVEEFKKEFSDKAANNFGSGWTWLVKKAD-GSVAIVNTSNAETPLTGADKPVLTVDVWEHAYYIDYRNSRPNYLEAFWKLVNWDFVNENLA----

>A0A2A6RIS4_2024553_Chloroflexi_B_Ter

MPFELAPLPYAYDALEPHIDEATMRFHHDNHHNTYVTNLNNALAKYPQLADQSLEQLLTKLDELPDDIRMVVRNNGGGHWNHTFFWQIMAA-GGGAPSGALAEAINASFGSFETFKEQFKAAGLGRFGSGWAWLVSDAA-GTLSIVSTPNQDNPLMEGKTAILGVDVWEHAYYLKYQFKRPAYLDAWWNVVNWPKVAELYAAAQK

>A0A2B7GK67_1608465_Halobacteria_A_Arc

ADHELPPLPYDYDALEPALSEQVLTWHHDTHHQGYVNGLNSAEETLAENREEGDF---GSTP----GALKNVTHNGCGHYLHTLFWENMSPNGGGEPEGDLADRIEEDFGSYEGWKGEFEAAAG--AAGGWALLVYDPVSKQLRNVAVDKHDQGALWGSHPILALDVWEHSYYYDYGPDRGDFIDAFFDVVNWEKAEEEYQTCLG

>A0A2B7GPY0_1608465_Halobacteria_A_Arc

GAHSLSAAAHAFNNLPRKSAEQVLTWHHDTHHQGYVKGLDSAEKTLAENRESGDF---GSSG----SAMRSVTHNGCGHYLHTLFWNNMSPNGGGEPSGDLRERIEDDFGSYEGWKGEFEAAAS--AAGGWALLVYDPVTKQLRNVVVDKHDQGALWGAHPVLALDVWEHSYYYDYGPDRGSFIDAFFEVVDWEHATEQYEKSVD

>A0A2D0NBJ4_1122177_Bacteroidetes_B_Mar

MSYTLPELSYAHDALEPHFDAQTMQIHHQRHHQGYVNNLNNAIAG-TEAEQESLEDILQNISKYT----TAVRNNGGGHYNHALFWETLSPSPKTSPTGKLADEINKVFGSLENFKEQFKQTGLTRFGSGWAWLIVKYN-GTIGITSTANQDNPLMDRGFPILGIDVWEHAYYLKYQNKRAEYLEAFWSVLDWTAVEQKYEAALA

>A0A2D0ND73_1122177_Bacteroidetes_B_Mar

MAFQLPDLPYAYDALEPHLDARTMEIHHTKHHQGYTNKLNDAIAG-TDLEKKSIKEILANVSKYP----AGVRNNGGGFYNHDLFWKVMSPDGGGVPSEKIHKAIMRDFGSFEKFKEEFSAAAGSRFGSGWAWLCVDSD-DKLFICSTPNQDNPLMDNGTPILGLDVWEHAYYLNYQNRRPDYVSAFFNVINWHEVTERYNDANK

>A0A2D0NIH0_1122177_Bacteroidetes_B_Mar

MAFKLPELPYYYDSLEPHIDEETMKVHHQKHHNGYTQKLNKAIEG-TDLENQPIEKILRNVSKY----STAVRNNGGGYYNHSLFWKSMSPKGGGQPEDRIHKGLEMAFGGFDQFKKKFKEAATGQFGSGWAWLCVDEPVGDLYICSTPNQDNPIMDKGVPILGIDVWEHAYYLKYQNRRADYIDAFFEVVDWKVIEERYLAART

>A0A2D3WM99_2015906_Proteobacteria_B_Mar

MKHRLPQLPYKMDALAPYISEETLRYHHGKHHAGYVNKLNALIEGT-EYEEMSLEQIIHQS-------DGAIFNNAAQTFNHNFYWHSLSPM-KTEPSDPFRRAIEETFGSMDAFKETFVQAATGQFGSGWAWLVLDQH-DRLLIETTSNAHTPIEHHRTPLFVCDVWEHAYYIDYRNERPKYVETFWDLINWDYASKIFEDKEH

>A0A2G0Q1U7_351679_Proteobacteria_B_Mar

MSFELPALPYAKDALEPHISAETLEYHYGKHHNAYVVNLNNLIKET-EFADKSLEEIIKTS-------ESGIFNNAAQVWNHTFYWHSLSPNGGGEPTGKVADAINSAFGSFTEFKQQFTDAALKNFGSGWTWLVKKTD-GSLAIVNTSNAATPLTGEDKPVLTVDVWEHAYYIDYRNARPQYLEHFWALVNWKFVEENLA----

>A0A2G0QEY2_351679_Proteobacteria_B_Mar

MSYSLPSLSYSYDELEPHFDKQTMEIHHTKHHQTYVTNTNNALEAFPELIKLDIDDLIQQLDKIPADKRAFVRNNAGGHSNHSLFWKGLKK--DTVLGGILKTAIERDFGSIDSFKEKFEQAAATRFGSGWAWLVLKED-GKLAVVSTANQDSPLMGSGYPILGLDVWEHAYYLKYQNRRPDYIKAFWHVVNWDEATKRYVEKTK

>A0A2G1X3V2_1483399_Halobacteria_A_Arc

MSYELDPLPYDYDALEPHISEQVLEWHHDTHHQGYVNGWNAAEETLEENREAGDF---SSSG----GAIRNVTHNSSGHILHDLFWQSMSPEGGAEPEGTLADRIAEDFGSYEAWKGEFEAAAS--AASGWALLVYDTFSNQLRNVVVDKHDQGAVWGGHPVLALDVWEHSYYHDYGPARGEFVDNFFEVVDWEEPSARFEQAVE

>A0A2G1X713_1483399_Halobacteria_A_Arc

MSYELDPLPYDYDALEPHISEQVLEWHHDTHHQGYVNGWNSAEETLEENRESHDF---SSSG----GAIRNVTHNSSGHILHDLFWQNMSPEGGDEPEGALADRIEEDFGSYEAWKGEFEAAAS--AASGWALLVYDTFSNQLRNVVVDKHDQGAIWGGHPILALDVWEHSYYHDYGPARGEFVDNFFEVVDWEEPSDRYEQAVE

>A0A2G3K1V1_1559339_Proteobacteria_B_Mar

MEHKLPELPYAIDALAPHMSKETLEYHYGKHHQTYITNLNNLIKGT-EHENASLEEIVKKAP------AGGLFNNAAQVWNHTFFWMGFKPNAGDAPTGKLLAAITAKWGTVDEFKKAFNTSAAGNFGSGWTWLVKKPD-GSLDIVNTSAAATPLTTADTALLTADVWEHAYYIDYRNSRPNYLEAFWKLVDWDAVAARLG----

>A0A2H3KUE0_1506545_Chloroflexi_B_Ter

MPFELPALPYAYTALEPHIDEATMHFHHDNHHNTYVTNLNNALANYPDLQDKSLEALIGDLDALPEAIRMVVRNNGGGHWNHALFWETMGPNAGGEPTGALGTAIAAAFGSFAEFKEKFKAAAIGRFGSGWAWLVVNPD-GTVAITSTPNQDTPVMEGKKAILGLDVWEHAYYLKYQYKRAGYVDAWWNVVNWAKVAELYDAARA

>A0A2I0FW47_2025587_Proteobacteria_B_Mar

MSYSLPSLPYAYDALEPHFDKETMEIHHSKHHQAYVNNANAALEG-TEFANLPAEELITKLDQLPADKKGPLRNNAGGHVNHSFFWKGLKT--GTTLQGDLKAAIEKEFGSVDAFKEAFEKAAATRFGSGWAWLVKK-D-GKLAVVSTANQDSPLMGSGTPIIGLDVWEHAYYLKYQNKRPDYAKAFWNVVNWDVAAENFAKA--

>A0A2I8DCS9_1758194_Proteobacteria_B_Mar

MAHTLPALPYAYDALEPHIDALTMEIHHGKHHQTYVNNLNAALEG-AGLSDEPVESLVARLDQLPAAITAAVRNNGGGHANHSLFWTVMSPRGGGAPDGALAQAIASDLGGLDAFREAFTKAALTRFGSGWAWLSVTPA-GKLVVESSANQDSPLMHGNTPILGLDVWEHAYYLKYQNRRPEYIGAFYNVIDWSEVARRYAAAVG

>A0A2I8DQ40_1758194_Proteobacteria_B_Mar

MAHTLPPLPYELDALAPHISKETLEFHYGKHHQTYVTNLNNLIPGT-EFENLSLEEIVKKS-------SGGIFNNAAQIWNHTFYWNSLAPKAGGAPSGKLADAINAKWGSFDAFKEAFNKSAAGNFGSGWTWLVKKAD-GSVDIVNTSNAATPLTTADKPLLTCDVWEHAYYIDYRNARPKYLENFWALVNWDFAAKNFA----

>A0A2I8VJM1_755307_Halobacteria_A_Arc

MSYELDPLPYEYDALEGSISEQVLTWHHDTHHQGYVNGWNSAEETLAENRESGDF---SSSA----GAIRSVTHNGCGHILHDLFWQCMSPEGGSEPSGALADRIEEDFGSYEAWKGEFEAAAG--AASGWALLVYDSFSNQLRNVVVDKHDQGALWGSHPILALDVWEHSYYYDYGPKRGDFVSAFFDVVDWNEPAARYEQAVD

>A0A2I8VJQ7_755307_Halobacteria_A_Arc

MSYELDPLPYEYDALEGSISEQVLTWHHDTHHQGYVNGWNSAEETLAENRENGDF---SSSA----GAIRSVTHNGCGHILHDLFWQCMSPEGGSEPSGALADRIEEDFGSYEAWKGEFEAAAG--AASGWALLVYDSFSNQLRNVVVDKHDQGALWGSHPILALDVWEHSYYYDYGPKRGDFVSAFFDVVDWNEPTARYEQAVD

>A0A2K8LAI0_1921087_Proteobacteria_B_Mar

MPFELPELPYARNALEPHISAETLDYHYGKHHNAYVTNLNNLIAGT-GHENSSLEEIIRSS-------TGPLFNNAAQVWNHTFYWNCLSPQGGGEPSGELGDAINRTFGSFGAFKEKFTASAATNFGSGWTWLVKNED-GTIEIVNTSNAGNPLTNGQKPLLTCDVWEHAYYIDYRNARPSYLEAFWHLVNWNFADNNL-----

>A0A2K9NQW2_960_Proteobacteria_B_Mar

MSFTLPELPYAKMALAPHMSEETLNYHYGKHHNAYVTNLNNLIKET-KFEKMTLEEIILSS-------EGPVFNNAAQIWNHTFFWNSLSPNGGGEATGKVAELITKKWGSFEAFKEAFTKSAVSNFGAGWTWLALNKA-GELEVVNTSNAQTPITTGHKPLITVDIWEHAYYIDYRNERPKFINAFWALVNWDFANKNL-----

>A0A2K9NRY1_960_Proteobacteria_B_Mar

PPFELAALPYAAGSLAPAIDQKTMEIHHDKHHQAYVDNLNKAIGK--E--KDDLMTIMSKVSG----KEAAVRNNAGGHFNHTFFWNILSGDKKQKMPERLEKEIEKTFGSVDKFKEAFEKAGASQFGSGWVWLIRDNS-GKLAITTTANQDNPLMDKGRPILAVDVWEHAYYLNYQNKRADYLKSIWKVVNWSQVDAFDREVTA

>A0A2N0WW65_2058089_Proteobacteria_B_Mar

MAIELPALPFEKNALEPHISAETLEFHYEKHHATYVVKLNGLIEGS-DLANSSLEEIVKNS-------EGPVFNNAAQVWNHTFYWNCLAPNAGGEPTGPIADAINQTFGSFDAFKAAWNDKAVNNFGSSWTWLVKKSD-GALEIVNTSNAATPLTDSVTVLLTVDLWEHAYYIDYRNVRPNYLNGFWALVNWDFVNSNYA----

>A0A2N3IHE2_2016530_Bacteroidetes_B_Mar

MAFTQAPLPYAENALEPHIDAQTMNIHYGKHHAAYVTNLNNAIAG-TDAENMSIEDLCKNISKYP----VAVRNNGGGHYNHTLFWEIMSPNGGGTPTGELAEAINKAFGSFDKFKEEFSKAAMGRFGSGWAWLCVMED-KSLKICSTANQDNPLMDRGTPILGLDVWEHAYYLKYQNRRADYVAAFFNVVDWNKVAEKYKKAI-

>A0A2N3IJD2_2016530_Bacteroidetes_B_Mar

QAFELPPLPYPYEALEPYFDTQTMQIHHTKHHATYVKNLNEAIAG-TKFEKMSLEEILANLTD----KDTKIRNNAGGHYNHSLFWKILTPKQSSQPTGKLAEAINTQFGGFEKFKEAFSEKAKSLFGSGWTWLAYEKK-QGLFLENTPNQDNLLMKIAKTIFGLDVWEHAYYLKYQNRRADYIQAFWNVVNWDEVSKNYEIALQ

>A0A2N3PQX2_382514_Proteobacteria_B_Mar

APFVQPPLPFSDTALAPVISAQTVQFHYGKHHAGYFAQLNQLTANT-PFAEMTLEQVVIKSASG---PDPRIFNNAAQAANHNFYWEGLKP-GGSAPGGALAQAIERDFGDLKSFKDAFVTHAVGLFGSGWAWLVED-G-GKLALFDGGNADTPLAHGKRPLAVVDVWEHAYYLDYQNRRADHVRAVVTLINWDVVRDRLSA---

>A0A2N3PTI0_382514_Proteobacteria_B_Mar

MALELPALPYDPKALEPFISANTLSFHHGKHHATYVNNYNNLTKDT-PNADKPLELVIKEVAG--DASKAGLFNNAAQVWNHTFFWNSLKHGGGGKPTGELLSKIEADLGSFDKFLEDFKAAATTQFGSGWAWLVLD-G-GKLKVTKTANADTPLAHGQTALFTVDVWEHAYYLDYQNRRPDFVSAVLNLANWDFVAQNLAKAK-

>A0A2N7UR03_1684789_Proteobacteria_B_Mar

MAHTLPELPYAYNALEPHIDATTMEIHHSRHHQTYVNNLNGALEG-TGLEDVPVDELVANLDRVPEEKRQAVINNGGGHSNHSMFWQVMSPNGGGAPKGKVGEAIDSELGGFDDFKDAFVKAALGRFGSGWAWLSVTPE-KKLVVENTLNQDNPLMHGNTPVLGLDVWEHAYYLKYQNKRPDYVAAFFNVVDWDEVERRYQAAVA

>A0A2P1PVM9_2021234_Proteobacteria_B_Mar

MAIELPALPYARDALAPHISAETIDFHYGKHHQTYVTNLNNQIKGT-EFENLSLEEIIRKS-------SGGMFNNAAQIWNHTFYWNCMKPSGGGEPTGKLADAINKSFGSFQAFKDQFTQTALTTFGSGWAWLVQRAD-GSLALVSTSNAATPLTGPDRALLTCDVWEHAYYIDFRNARAKYVETFWNLVNWDYVSAQMA----

>A0A2P2BQH2_1507512_Firmicutes_B_Ter

--FDKIVLPYGFRDLEPYIDEDTLEIHYGKHLQTYVNNLNNILKGYEEFKGKTLEDILKNVSSLPEEIRQSVINQGGGVYNHNLYFANLSPNPCETPKGDLLNKINDTFGSVENLKSELNKIAISKFGSGYASLLMDEN-KNLYVKATSNQDTTLEEGLTPILTIDVWEHAYYLNYQNRRAEYVENIWHVIDWSKVEKLYEK---

>A0A2P2BST4_1507512_Firmicutes_B_Ter

TPKE-PPLPYSYDSLEPYIDKETMILHYDKHYKSYLDKLNNAIKGYPDLYACSISDLLTCLDCLPSEIAKTIKDNGGGVYNHEFFFEIMSPN-RKKLHGKLKSAINRDFKSFDNFKNEFNKASLSVFGSGWAWLVSDDS-GNLSIITTQNQNTPITLNLKPIIGIDVWEHAYYLKYQNKRAEYINNWFNIINWSKAEENYIQNLK

>A0A2P2EAB2_1445552_Proteobacteria_B_Mar

PAFVQAALPYGFEALEPVIDTATMRLHYERHHGAQVAGLNTAVAANPTYAGKSLEALLAGASTAP----AAIRNNAGGHWNHSFYWQIMAPVGGGAPSPELAAAITRDFGSLDAFKAQFRAASMGRFGSGWAWVVVGKD-GKLAIGSTPNQDNPLMDRGKPILGNDVWEHAYYLKYQNKRADYVDAWWSLINWNKVSSLYAEAIK

>A0A2P8H4Q1_1176648_Firmicutes_B_Ter

MAYQLPELPYAYDALEPHIDKETMNIHHTKHHNTYVTNVNAALEGNEELASKSVEELISDLSSVPEEIRTAVRNNGGGHANHSLFWQLLSPNGGGNPTGALAEAIDSKFGSFDEFKTQFENAGKTRFGSGWAWLVSN-N-GELEVTSTQNQDSPLMEGQTPLLGVDVWEHAYYLKYQNRRPDYLAAFWNVVNWEEVSRRFDATK-

>A0A2P8KDF2_1105204_Proteobacteria_B_Mar

MEHVLPPLPYPLDALAPEYSKETLEYHYGKHHNAYVVNLNNLQKGT-EFENMELEEIVKKS-------SGGIYNNAAQIWNHTFFWNCMKPAGGGEPTGALAKAIDAKWGNYAAFKEAFVKSAVGNFGSGWTWLVKKAD-GTVDIVNMGAAGTPLTTGDKPLLTVDVWEHAYYIDYRNLRPKYVEAFLKLVNWDFAAKNFG----

>A0A2P8KJW4_1105204_Proteobacteria_B_Mar

MDLQPRPLTVDFSRL---LSERLVASHHENNYAGAVRRLNAIRQQLAQLD-WAHTPVFL-VN----GLKREELIAFNSAWLHELYFDNLGSDG-VLPDGGLSIALERDFGSVDRWRAEFMALAKMGGGSGWALLSWSTREGRLVNHWAADHTHLMA-GATPVLALDMYEHAYHMDFGSKAGAYVDAFIKNIRWEAVQEQYGAAVA

>A0A2R4X0R1_1679096_Halobacteria_A_Arc

SHPELPDLPYAYDALAPSISEQVLTWHHDTHHQGYVNGLDAAEQTLAENRANGDF---ASSG----AALRSVTHNGCGAYLHELFWTNMSPNGGGEPSGSLRERIEEDFGSYEGWKGEFEAAAS--AAGGWALLVYDSKAGQLRNVVVDKHDQGALWGAHPILALDVWEHSYYHDYGPARGDFVDAFFDVVDWDAVAEEYSSVVG

>A0A2S0N786_1868589_Proteobacteria_B_Mar

MSFTLDDLPYAYDALQPYMSKETLEYHHDKHHQAYVTNGNNLIKGT-EFEGKSLEEIVKGSFG----KHAGIFNNAGQHYNHIHFWKWMKPNGGGKIPGGLEKALIDSFGSIEKAKEDFIQGGVTQFGSGWSWLAVQ-G-GKIVVTKTPNGESPLVHGGVPILGVDVWEHSYYIDYRNRRPDYLKAFVHLVNWEYVDELFQKATA

>A0A2S0NH98_1868589_Proteobacteria_B_Mar

GPFTLPPIGYAYDALEPHIDKMTMEIHHTRHHAAFLATINNAAKTYDELKPANTEKVLRNLSAVPENLRPGVRNTLGGYWNHVHFWDIMTPGGAKQPSGPLLQAINATFGDVDKMRQQFNQASVGRFGSGWGWLVVGRD-GKLAVVSTPNQDNPLMDGKGVVMGIDVWEHAYYLKYQNRRPDYVTTWWNVVNWDKAAANFQKAMG

>A0A2S1JPT9_359370_Proteobacteria_B_Mar

MAFELPELPYAKNALAPHISEETLEYHYGKHHKTYVDKLNGLLEGT-ADADKSLEDVIKSS-------SGGVFNNAAQVWNHTFYWNCLSPNGGGEATGAIAEAINAAFGSFDQFKEAFTTSAVNNFGSGWTWLVKNAD-GSVAIVNTSNAATPLTEGVTPLLTCDVWEHAYYIDYRNLRPKYMEAFWALVNWEFVNQNFA----

>A0A2S6N6L1_333368_Proteobacteria_B_Mar

MSFVLDPLPYAYDALAPYISSETLEFHHDKHHAAYVANANNLIKGT-QWEGKPIEEVIKTSHG----KNPGVFNNVAQIYNHAEFWKYLKPAGGGKVPGKVEKALVEAFGSVEKALEDLTQAGVTQFGSGWAWLEVK-D-GKVAVSKTPNAENPLIHGAAPILTVDVWEHAYYIDYRNRRPDFIKTVLNLVNWEYVEAQYEKAVG

>A0A2S6NHJ6_333368_Proteobacteria_B_Mar

APRAPIALPYDDHALEPVISANTLGFHYGKHYKGYLDTLAKLTAGG-DLAEAPLDQIIALSSG--APDKTALFNNAAQAWNHAFYWNSLKPGGGGTPPAALAQRIEADFGSLDAVKKDLSAAALSQFGSGWAWLIED-A-GKLKVVKTGNADTPLAAGKKPLLTIDVWEHAYYLDYQNRRADYVAAVIKLLNWEFALTNLG----

>A0A2T0XJH1_323426_Proteobacteria_B_Mar

MAHTLPPLPYAMDALAPTISKETLEFHYGKHHQTYVTNLNNLIPGT-EFESASLEEIVKKS-------SGGVFNNAAQIWNHTFYWNSLSPNGGGEPTGKLADGITAKWGSVAAFKEAFNKSAAGNFGSGWTWLVKKAD-GSLDIVNTSNAGTPLTTTDVALLTCDVWEHAYYIDYRNARPKYLESFWSLANWSFAAQNLA----

>A0A2T5FTW8_1735121_Proteobacteria_B_Mar

MAFELPPLPFDKTALEPVLSAETFDFHHGKHHKAYVDKTNGWIDEK-GLAGLSLIDVVKKAKD---TGDKGLFNNAAQIWNHSFFWSSLAPEGSTKPSEKLQALIDSGFGSTAALLDKLAAEAVGHFASGWAWLVLE-G-DALKVTSYHDADTPIVEGVKPLFTLDVWEHAYYIDYRNARPKFAESVLKVINWDFVSQNLDGLGA

>A0A2T5FZN3_1735121_Proteobacteria_B_Mar

APPQPVPLPFDPKTI---LSEKLLVSHHDNNYVGAVKRLGAIAAQIASLD-PAAAPGFL-LN----GLKREEIIAWNSMILHELYFAGLG--APTRPGKALAAAIERDFGSDARWRSEFAAMGKLAGGSGWVLLTYSHRDDRLVNQWAADHTMTLA-GATPIVALDMYEHAYAIDYGARAAAYVDAYMGAINWTSADNRFAKAAA

>A0A2T5G1R0_1735121_Proteobacteria_B_Mar

GPFSLAPLPYAYDALAPVIDEATMKLHHDKHHQAYVDALNKAVTADPALQGQTLEQLVAKAGTLP----TVVRNNAGGHWNHTFYWKTMTPAKSGAPSGALAAAIEASFGSLDKFKAAFKEAGTKQFGSGWVWLIVGAD-GKLAITSTPNQDNPLMDKGLPVLGNDVWEHAYYLTYNNRRGDYLEAWWKVVDWAEVSRRFAAARR

>A0A2T6BC70_1242148_Firmicutes_B_Ter

MAHELPELPYSADALEPHIDARTMEIHHGRHHKTYVDKLNAALEGNTALQDKSIEDLLRNIDQVPENIRTAVRNNGGGHANHTLFWQIMSPNGGGQPTGDLATAINDTFGSFEKFQEEFTNAAVGRFGSGWAWLIVKND-GSLAVINTLNQDSPYMEGHTPILGLDVWEHAYYLKYQNKRPEYIKAWWNVVNWDEVNKRYQEAKK

>A0A2T6C4K2_1242148_Firmicutes_B_Ter

GGHVLPPLPYPYEALEPYIDAKTMRLHHDEHHKSYVDGLNKAEIMMAQARKTGNF---NLIK----HWEREAAFNGAGHYLHTIFWEIMSPKGGGKPSGKLLKQIRKDFGSFEAFKKHFSAAAEKVEGGGWAILVWAPRAQRLEILQAEKHQNLSQWDVVPLLVLDVWEHAYYLKYPNKRKAYIRAWWNVVNWPAVECRYLQARK

>A0A2U1E3U8_46507_Firmicutes_B_Ter

--IELIKLPYAYDALEPVISKETIEYHHDKHHQAYVNKLNELIEG-TELADMHPCEILKNLDKAPADKKQAIINQGGGVYNHNVYWTQLAKDGQKEPKGELKEAIDKAFGSFDEFKEKFEKAGAGQFGSGWAWLVEK-D-GKLEIMTTANQDAPISKGYKVLLNNDVWEHAYYIDYRNKRAEYLKKFWDIVDWSVVEERFVK---

>A0A2U2AEQ9_472582_Proteobacteria_B_Mar

MSFELPKLPYAMDALAPHISKETLEYHYGKHHQTYVNTLNTLIEGT-EFANASLEEIILKA-------SGPMFNNAAQVWNHTFYWESLKPQGGGAATGKIAEEINKKWGSFEEFQKAFDKCAIGTFGSGWAWLVKNAD-GSIELVSTSNAATPLTAGQTPLLTCDVWEHAYYIDYRNSRPNYLENFWALVNWDKVNERLG----

>A0A2U9IFV2_41673_Thermoprotei_A_Arc

KKYELPPLPYKLDALEPYISKDIIDLHYNGHHKGYVNGANSFLDRMQKVTKGELSSGQYDIQ----GLLRGLVFNINGHKLHSLYWENMAPNGGGKPGGSLADLIEKQYGSFDKFKALFTEAANSLPGTGWTVLYYETENGNLQIMTFENHFQNHIAELPIVLILDEFEHAYYLQYKNKRADYVNAWWNLVNWDYANKKLEKYLN

>A0A2V3PRT9_1605892_Bacteroidetes_B_Mar

MKFEMPKLPYATNALEPVISQQTVELHYGKHLQTYVNNLNNLVPGT-KFENSDLITIIKES-------DGAIFNNAGQILNHDLYFASFTPGTGGSPKGKLAEAITAKFGSFENFQKEFNTAGAGLFGSGWVWLAKDAS-GNLSIEKEPNGSNPVVKGLTPILGIDVWEHAYYLDYQNRRADHLAEVWKIINWDVVSARY-----

>A0A2W1JHI3_1764569_Cyanobacteria_B_Ter

EEFVLPPLPYAYDALSAYVDEQTMMIHHDKHHAGYVKNLNDAIAKHPDLKGKSVEALLQDLDNIPEDIRVTVRNNGGGHANHTMFWETMTPDSKGQPTGKLAKAINSSFGSFDAFKEAFNTAGTKQFGSGWAWLVLTPD-NKLEVTSTANQDSPLLDGNTPIMGNDVWEHAYYLKYQNKRGDYLNAWWNVVNWDQVGQRYEQALS

>A0A2X0WVT3_179995_Proteobacteria_B_Mar

MSFAITPLKFEKTAV-PFLSENTFNFHHGKHFQTYIDTANKLVAGT-DYEGKTLEEIITTA-------SGPLFNNAAQAWNHAFYFNCIGTAKKEVP-AKLLELINANFDSFESFAEKFIASATTNFGSGWTWLVQTGP-NFLKIVNTSNAGNPMVDGFTPLLTVDVWEHAYYLDYQNRRADYLKDFVEYIDWEFVASNLK----

>A0A2Z2HUJ4_745377_Halobacteria_A_Arc

TDHELPPLPYDYDALEPSISEQVVTWHHDTHHQGYVNGLNSAEETLAENRESGDF---DSTP----GALSSVTHNGCGHYLHTLFWENMSPDGGGEPAGDLAERIEEDFGSYEGWKGEFEAAAG--AAGGWALLVYDPVAKQLRNVAVDKHDQGALWGSHPILACDVWEHSYYYDYGPDRGSFVEGFFDVVNWDKVDEEYQKCID

>A0A2Z2L2V6_549298_Proteobacteria_B_Mar

MKFKLPKLPYAQDALEPVISKETIEYHYGKHHQTYVTNLNNLIEGT-EHSGQTIEEIIKAS-------SGGLFNNAAQVYNHTFYWNCFSPN-KTKPSNQLKVAIVETFGSFDKFKEEFSKTAVTTFGSGWAWLVKDSS-GKLEILSTSNAGCPLTNNKHPLLTFDVWEHAYYIDHRNARPKHVKALWDIINWEFVSEQFAK---

>A0A2Z2P4N8_1192854_Proteobacteria_B_Mar

MSFTFPDLPYDFTALEPHVDAQTMEIHYDRHHRTYFNNFTGAVNDTP-LADKSLEEVMAQVDS---STSPAIRNNGGGYYNHILYWNSMC-NGGSAPSSELATAIDSTFGSMAELKTGLKQAGITRFGSGFAWLIVK-N-GKLEITSTPNQDNPLMSQGTPILALDVWEHAYYLKYQNKRPDYIDGWLEVVDWAKTSERFAAASA

>A0A2Z3GPK6_114_Planctomycetes_B_Mar

KGFALPKLPYAFDALEPVVDAKTMEIHHGKHHQAYVDNLNKALAGKPDLLAKPIVELVRDWKKLPADLQAPVRNNGGGHLNHTWFWQMMKKD-GGAPKGELAKAIDASFGSLDGFKKEFATAATTQFGSGWAWLVKGKE-KPLAVVKTPNQDNPVTDGQAVLLGCDVWEHAYYLKYQNKRADYVNAWFSVVNWDFVAELFAAK--

>A0A2Z3H6M4_114_Planctomycetes_B_Mar

MAHTLPALPYAFDALEPHIDAKTMEIHSQKHHKAYVDNLNKALESAPELANKDITTLLREIASVPASIKQAVINNGGGHHNHALFWDVMGPNAGGEPTGAIADAIKDAFGDFAKLKETVKANGTGQFGSGWSWVVYNPS-GKLEAIKKPNQDSPLMDGLVPVLGVDVWEHAYYLKYQNLRPAYIDAWWNVVNWKAVEAKYAAAKA

>A0A2Z4FGN2_1548548_Proteobacteria_B_Mar

GEHKLPELPYAYDALEPFIDEQTMRLHHDIHHNGYVNGLNKAEADLAAARESGDF---SKIA----DIQRRLAFHGSGHVNHTLFWRNMCPPGDKDPSGDLAKQIEKDFGSLENLKAQFAAAAKTVEGNGWGFLVWSPEGGYLTTVAAENHQKNFVNNQIPLLVLDVWEHAYYLKYQNKRGDYVENFFKIVNWENVAQNFANAKA

>A0A326U110_644383_Chloroflexi_B_Ter

MSYELPPLPYAYDALEPYIDAQTMQLHHDKHHATYVTNLNAAVKDLPELASLPVEKLIQRLSDVPENVRTAVRNNGGGHANHTMFWNIMKPGGSNAPTGELASAINETFGSFDAFKQAFNDAGAKRFGSGWAWLALDRS-GKLSVISTPNQDSPLMDGLYPVLGNDVWEHAYYLKYQNRRPEYLNAWWNVVNWDEVARRYEEGRT

>A0A326U2A6_644383_Chloroflexi_B_Ter

MAFELPPLPYDYNALEPYIDAQTMQLHHDKHHATYVNNLNAALQNQASLANQSVEDLLRHINEVPENIRTAVRNNGGGHANHTMFWQIMKPNGGGEPKGDLANAINAKFGSFDAFKQLFNDTGAKRFGSGWVWLVLDRG-GNLEVISTANQDSPLMEGLYPVMGNDVWEHAYYLKYQNRRPEYLNAWWNVVNWDEVEKRYAQGRG

>A0A326UA20_644383_Chloroflexi_B_Ter

QEFTLPPLGYAFNALEPYIDARTMELHHDKHHATYVKNLNAALKE-VALQASSAEDVLRKLNDIPEAKRETVRNNAGGHVNHTMFWQIMRPGGKKEPQGELAEALKSTFGSIDEFKQKFNKVGAGRFGSGWVWLVMRKD-GKLHLKSTPNQDSPILENEYPIMGNDVWEHAYYLKYQNKRADYLNAWWHVVNWDEVEKRYQQAKK

>A0A327KT03_29409_Proteobacteria_B_Mar

MSFSLPDLPYAHDALAPYMSRETLEYHHDKHHAAYVNTGNTLLKGS-EWEGKSLEEIVKGSYG----KNQALFNNAGQHYNHTHFWKWMKPGGGGKLPGALEKAIVADLGSVAKMKEDFVQAGVTQFGSGWAWLAVK-D-GKIIVTKTPNGESPLVHGAVPILGADVWEHSYYIDYRNRRPDYLKAFLSMVNWDYVAELYAAASK

>A0A327L5R7_29409_Proteobacteria_B_Mar

GPFRLDPLAYPANALEPHIDAKTMEIHHDRHHQAYVNNLNAAVKDHGQVAAMPLQDILAKLSEMPESIRTAVRNNGGGHANHTMFWQVMGPGGGGAPSGPLAEAITRDLGGFDKLKSDFNGAGARVFGSGWVFVTVTPD-GKLALESKPNQDTPLMEGKKVLFGNDVWEHAYYLTYQNRRPDYLAAWWNTVNWSKVGERYAAAKA

>A0A328VKE5_1825093_Chloroflexi_B_Ter

MAFELPPLPYSFDALEPHIDAQTMQIHHDKHHGTYVANLNSALEGHP-FANLPVEEVLRRINEVPENVRTAVRNNGGGHANHSMFWRIMKPNGGGQPSGDLAKAIEQTFGSFENFKAQFNDTGLKRFGSGWVWLVLDKQ-GKLQVISTANQDSPYMDGLYPVMGNDVWEHAYYLKYQNRRADYLNAWWNVVNWEEIERRYAEGLS

>A0A328YCR8_1101402_Bacteroidetes_B_Mar

MAFELPQLPYAYDALEPHIDARTMEIHHSKHHNAYTTNLNAALAG-TDLEGKSIEEILKNLDMSQ----AAIRNNGGGFFNHNLFWTVMSPNGGGLPTGDLASAIDTAFGSFDAFKAAFSKAGATQFGSGWAWLCVK-D-GKLEVCGTPNQDNTLMPGGTPILGMDVWEHAYYLHYQNRRPDYIEAFFNVINWDEVSRRFHEAI-

>A0A328YDD7_1101402_Bacteroidetes_B_Mar

GAFEVTKLGYKYDDLAPHIDAMTMEIHYSKHYVTYTNALNNLLKG-TEQENIPIEDIYKKLDL----NNNELKNNLGGYYNHTLFFDILGCKSNEKPSDTLTSSINKNFGSLESFKTQFEDAANKVFGSGWAWLVVSKS-GQLSITTTQNQDNPLLSKGIPILGIDLWEHAYYLNYQYRRKKYIEAFFKVINWKKVSEKYDEALK

>A0A345E419_1547899_Halobacteria_A_Arc

MSYELDPLPYDYDALEPHISEQVLTWHHDTHHQGYVNGWNSADETLEANREDGDF---GSSA----GAIRNVTHNGCGHILHDLFWNCMSPEGGDEPSGDLADRIEEDFGSYDAWKGEFEAAAG--AASGWALLVYDSFSNQLRNVVVDKHDQGALWGSHPILALDVWEHSYYHDYGPARGDFVDNYFEVVDWSEPSARYEQAVE

>A0A345UFZ3_1457365_Bacteroidetes_B_Mar

GEYVLPELNYAYDALEPHIDAQTMELHHSRHHQAYVNGLNTALSRLAEARENDDF---SIVK----HWSRETAFHGGGHFLHAMFWEIMSPNGGGEPRDTLRNAINRSFGSYVGFKRHFKAASNAVEGSGWGIMAFEPNSGNLVIHQAERQSDLTLWVTQPLVMVDVWEHAYYLRYQNRRGEYVDNFMEVLDWDKVAEMYEAAAS

>A0A345UPY2_1457365_Bacteroidetes_B_Mar

MAFTLPELPYAYDALEPHIDAKTMEIHHTKHHNAYITKANDALAG-TDLADASVEEVLTSLDKLPADKKQGVINNAGGHANHTLFWSVLSPNGGGNPTGELAGAIDSTFGSFDAFKEAFANAAATRFGSGWAWLVVDGE-GKLHVTSTANQDSPVMSGHTPILGLDVWEHAYYLNYQNRRPDYISAFWSVVSWDKVNELYLAAK-

>A0A348B0W0_1670455_Thermoprotei_A_Arc

RKYELPQLPYKLDALEPYISKDIMDVHYNGHHKGYVNGANSLVDRLEKVVKGEVTS--YDVQ----GIVRGLAFNINGHKLHDLFWKNMAPAGGGKPGGALADLIQKQYGGFDRFKQVFTEVANSLPGTGWTVLYYDTENGNLQLMTFENHFMNHIAELPIIMIVDEFEHAYYLQYKNKRADYVSAWWNVVNWDYAEEKLKKYMK

>A0A365T5J1_29295_Halobacteria_A_Arc

----------DYDALEPSIDAQIMKLHHDKHHQGYVEGANAALQKLMKMRDNRDF---EDIK----HIKRDLSFNLSGHILHSIFWEVMSPDGGRTPDGDLANAIQRDFGSISAFKREFSAAAENVEDSGWGMLVYDHLADQLLVTQAEDHNDLAVQASTPLLVLDVWEHAYYLQYQTERGKYVDAFWNIVDWDEVSARYTAVKQ

>A0A365T5J6_29295_Halobacteria_A_Arc

STYSLPPLSYDYDALEPSIDAQIMKLHHDKHHQGYVEGANAALQKLMKMRDNGDF---EDIK----HIKRDLSFNLSGHILHSIFWEVMSPDGGGTPDGDLANAIQRDFGSISAFKREFSAAAENVEDSGWGMLVYDHLADQLLVTQAEDHNDLAVQAATPLLVLDVWEHAYYLQYQTERGKYVDAFWDIVDWNEVNARYKTVKQ

>A0A365TEZ3_29295_Halobacteria_A_Arc

SNAELPPLPYDYDALEPHISEQVLTWHHDTHHQGYVNGLNDAEETLAENRENGEF---GSSG----AAIRNVTHNGCGHYLHTLFWNNMSSNGGGEPDGELADRIEEDFGSYEGWKGEFEAAAK--NASGWALLVYDPVAKQLRNLVVDKHDQGALWGSHPILALDVWEHSYYYDYGPDRGDFISNFFEVVDWDEVADQYDTVRS

>A0A365TF12_29295_Halobacteria_A_Arc

ATYELPELPYDYDALEPNIDARIMELHHDKHHQGYVDGANAALETLSEMRENDEW---GDVK----GVERNLAFNLSGHVNHSVFWENMSPDGGGEPGGELADALDDHFGGVDAFKSHFSAAAKGVEGSGWGLLLYDHVSDEPIVSMAENHQNQTPQGTTPLLVLDVWEHAYYLQYENNRGEYVDNFWNVVDWDDVAERYDEASS

>A0A366ENT2_1473586_Proteobacteria_B_Mar

MTFSLPELPYAYDALGPYMSRETLEYHHDKHHLAYVNNGNNAIKGT-EWEGKPIEEIIKGSFG----KTPAVFNNIGQHYNHSLYWLSMKPNGGGAIPGELEKALVESFGSVDKAKEDLAQAAVTQFGSGWAWLSLK-D-GKLEITKTGNAESPLVHGAQPLITVDVWEHAYYIDYRNRRPDFVKAVLNLINWEFAAEQFAKA--

>A0A367QAR4_1844469_Cyanobacteria_B_Ter

MAFELPPLPYNYDALEPYIDTETMRLHHDLHHQAYVTNVNAAVEKHPELNSKSIEELIVNLNNLPEDIRTAVRNHGGGHLNHTMFWQIMAPNADGEPTGAIADIINNNFGDFETFKQHFNDAGAKQFGSGWVWLVRTPD-EKFAITSTPNQDNPMTSGSFPIMGNDVWEHAYYLKYQNRRPEYLKQWWNVVNWDEINKRLQMSKH

>A0A367QB34_1844469_Cyanobacteria_B_Ter

MAFSQPPLPFDFNALEPYMKGETFEYHYGKHHKAYVDNLNKLTEGT-ELADKSLEDVIQISFK--DSSKAGIFNNAAQVWNHTFFWNSLKPAGGGTPTGELAAKIDKDFGSFDKFKEEFSNAATTQFGSGWAWLIDD-G-GTLKVIKTPNAENPLAHGKKALLTLDVWEHAYYIDYRNARPAFIKNFLQLINWEFAAANYAKA--

>A0A367QYP4_1844469_Cyanobacteria_B_Ter

YPAKLPPLPYDYGALEKAIDAETMKLHHDAHHASYVNNLNDALKRYPDLQKNSVEALLKDLNKVPEDIRTKVRNNGGGHLNHTIFWQIMSPQGGGEPTGAIAQEINQTFGSFDAFKKQFNVAGGDRFGSGWVWLVRNPQ-GKLQIVSTANQDNPITEGLYPILGNDVWEHAYYLRYRNRRPEYLNNWWNVVNWSEINRRNQISST

>A0A368N5W7_1126245_Halobacteria_A_Arc

MSYELDPLPYDYDALEPHISEQVLTWHHDTHHQGYVNGWNSAEETLEANRGEDDF---GSSA----GAIRNVTHNGCGHILHDLFWNCMSPEGGDEPSGDLADRIAEDFGSYEAWKGEFEAAAG--DASGWALLVYDSFSNQLRNVVVDEHDQGALWGSHPVLALDVWEHSYYHDYGPARGDFVDNFFQVVDWSEPSTRYEQAVE

>A0A380N0R1_13276_Proteobacteria_B_Mar

MSYTFQDLPYAYDALEPVIDEATMHLHKDKHHKTYFDKFVAAIAE-TDFNGKSLPEIFANISAL----SPAIRNNGGGYYNHDLFLNGMAPQENNTPSGELANAIKAAFGSFEDFQKAFADAAVNQFGSGWAWLVLQ-D-GKLVVSSTPNQDNPLMDRGTPILGLDVWEHAYYLNYQNKRPDYVSNWWKVVNWDYVSEQYAAAK-

>A0A395JG70_644221_Proteobacteria_B_Mar

MTIQLKALPYKYDELEPFVSEETLLQHHAMHHRDYVDSLNAEIRGT-PHSNKGLSEIVLSA-------EGRIYQNAAQVWNHNFYWQCLSPSRDLLPSRELLEAIEDNYGSFDLFKVAFENAVVHQFSAGWTWLIRLAS-GRLRIINTKDADTPVTDNSMPLLVIDVWEHAYYIDYRHRRKNYVRQFWGHVNWPFVSQNYRK---

>A0A395JH00_644221_Proteobacteria_B_Mar

MSFELPALPYDRDALAPRISAETIDYHYGKHHQAYVNNLNGMIEGT-DHAGKSLEDIIRSS-------DGGLFNNAAQVWNHTFYWHSLSPNGGGEPSGDLAAAITSTFGSFAEFKDKFTASAGGNFGSGWTWLVKNAS-GELEIVNTSNADTPITDSVTPLLTVDVWEHAYYVDYRNARPEYLKNFWELANWEFAAQNYAA---

>A0A399EPG5_2026184_Deinococcus-Thermus_B_Ter

MSFKLPELPYPKDALEPHIDAQTMEIHHGKHHATYVNNLNAALEKHPELHSWSIEDLLTKIAQVPEDIRTAVRNNGGGHHNHTLFWDILTPGGAKEPTGRLAEAIQATFGSFEELKNKMTQAGLTRFGSGWAWLVKDKD-GKLLVYSTANQDSPLMEGHTPLLGIDVWEHAYYLKYQNRRPDYLAAIWNVINWDKVAERF-----

>A0A3E1EU86_1737063_Bacteroidetes_B_Mar

MAFELPKLPYAHDALEPNIDAKTMEIHHGKHHQGYTTKLNAAIEG-TDLEGKSIEEILK-AGKDK----AAVRNNGGGYYNHNLFWTVMSPNGGGNPIGDIKDAIDSAFGSFDAFKDEFSKAAATQFGSGWAWLCVK-D-GKLEVCSTPNQDNPIM-EGTPVLGLDVWEHAYYLNYQNRRPDYIEAFFNVINWEEVNRRYNEAK-

>A0A3M0CGB4_911205_Proteobacteria_B_Mar

MSFELPALPYAQDALEPHISANTLSFHHGKHHNTYVVNLNKLVEGT-EYDGKSLEDIMKATAG--QADKAGIFNNAAQVWNHTFYWHSMSPNGGGSPSGDLAAKIDEDFGGYDAFADAFKAAGATQFGSGWAWLVLD-G-GKLAVVKTPNAECPLTDGAVPLITMDVWEHAYYLDYQNARPAYMETFLKLVNWEFAARNLAAAA-

>A0A3M9JL82_1853682_Halobacteria_A_Arc

SDYELPPLPYEYDALEPSISEQVLNWHHDTHHQGYVNGWNSAEETLEEAREEGDF---SGSA----GAIRNVTHNGSGHVLHTLFWESMGPEGGDAPEGDLAERIEEDFGSYEAWKGEFEAAAS--AAGGWALLVYDTHSNQLRNLVVDKHDQGALWGAQPILALDVWEHSYYYDYGPARGDFVSAFFDVVDWEEPSARYEQAVE

>A0A3M9JV35_1853682_Halobacteria_A_Arc

SDYELPPLPYEYDALEPSISEQVLNWHHDTHHQGYVNGWNSAEETLEEAREEGDF---SGSA----GAIRNVTHNGSGHVLHSLFWECMSPNGGDEPAGALAAAIQEDFGSYEAWKGEFEAAAS--AAGGWALLVYDTHSNQLRNLVVDKHDQGALWGAQPILALDVWEHSYYYDYGPARGDFIDAFFDVVDWETPADRYEEAVS

>A0A3N1MAT8_94_Proteobacteria_B_Mar

MAFELPPLPYAPNALEPHMSANTFSFHHAKHHQAYVTNLNNLIKDT-PLADKSLEEIILATAS--DASKAGVFNNAAQVWNHTFFWNSMKPAGGGAPSGDLAKKIDAAFGSLAKFKEEFKAAAVGQFGSGWAWLVLD-G-SDLKIVKTGNAGTPMTSGQKALLTVDVWEHAYYLDYQNRRPDFVQTFLHLVNWDFAAKNLG----

>A0A3N6LM69_1679091_Halobacteria_A_Arc

TDHELPPLPYDYDALEPSISEQVVTWHHDTHHQGYVNGLNSAEETLAENRESGEF---GSTP----GALGNVTHNGCGHYLHTLFWENMAPNGGGEPDGDLADRIEEDFGSYEGWKGEFEAAAG--AAGGWALLVYDPVAKQLRNVAVDKHDQGALWGSHPILALDVWEHSYYYDYGPDRGSFIDGFFDVVNWDSVDEEYQKCLD

>A0A3N6M5P3_1679083_Halobacteria_A_Arc

TDHELPPLPYDYDALEPAISEQVVTWHHDTHHQGYVNGLNSAEETLAENRESGDF---DSTP----GALGNVTHNGCGHYLHTLFWENMSPDGGGEPDGDLADRIEEDFGSYEGWKGEFEAAAG--AAGGWALLVYDPVAKQLRNVAVDKHDQGALWGSHPILALDVWEHSYYYDYGPDRGSFIDGFFDVVNWDSVADEYQKCLD

>A0A3P3RB00_671145_Halobacteria_A_Arc

SNPELPSLPYDYTALEPHISEQVLTWHHDTHHQGYVNGLESAEEELAQNRADGDF---STTG----GALSNVTHNGCGHYLHTMFWENMSPDGGGTPEGALADRIEEDFGSYEGWKGEFEAAGT--AASGWALLVYDPVAKQLRNLRVNRHDQGALWGAHPILALDVWEHSYYYDYGPDRGSFIDAFFEVIDWTNVSEQYTKVVS

>A0A3P3RGH5_671145_Halobacteria_A_Arc

MSYELPPLPYEYDALEPHISEQVLTWHHDTHHQGYVNGWNSAEETLAENREAGEF---DGSA----SALRSVTHNGCGHVLHDLFWQNMSPEGGAEPSGALADRIEEDFGSYEAWKGEFEAAAG--DASGWALLVYDSFSNQLRNVVVDKHDQGALWGSHPILALDVWEHSYYYDYGPARGDFVDAFFEVVDWEEPATRYEQAVE

>A0A3P3RLZ3_671145_Halobacteria_A_Arc

DRYTLPPLPYDYDALAPAIDEQIMRLHHDKHHQGYVDGANEALDKLDTMRQKDRF---NSIK----PIKRDLSFNVSGHILHSVFWESMSPNGGGRPAGGLATALERDFGSVDGAIAEFCTAAKNVESSGWGLLVYDHLADRLLVTQAEAHNDLAVQGATPLLVIDVWEHAYYLQYTNDRGAYVDAFLDVVDWETVRSRYEAVTS

>A0A3S0Y3G1_211165_Cyanobacteria_B_Ter

MAHTLAPLPYDYAALEPYIDAQTMQLHHDKHHAAYVNNLNAALEKYPDLMNKSIEELIINLEQIPSEIRTVVRNNAGGHFNHTLFWKSMSPNGSRTPTGAIAEAIEVTFGSFDNFKQQFNEAGMKQFGSGWVWLTFNRS-GKLEIVTTPNQDSPLTTGLYPLLGNDVWEHAYYLKYQNRRKDYLDAWWNVVDWDEVNRRFETARQ

>A0A3S0ZDA7_211165_Cyanobacteria_B_Ter

NPAQLPPLPYPYNALEKAIDAETMKLHHDRHHATYVENLNNALNQYPQLQDRSVEALLRDLNSVPEDIRTTVRNNGGGHLNHTIFWQIMSPQGGVEPTGEIAQEINQTFGSFEEFRKQFNEAGGDRFGSGWVWLVRNPQ-GQLQITSTPNQDSPITEGSYPIMGNDVWEHAYYLRYQNRRAEYLNNWWNVVNWQEINRRAQASRQ

>A0A3T0EBQ7_1434191_Proteobacteria_B_Mar

SAFSLPGLPYAYDALEPVIDAETMELHHSRHHQTYVNGLNSALEGRSDLQGTSLEDILARVSDLP----KAFRNHGGGHWNHTFFWESMTPADSGAPSGALAEAINAKFGSLDEMKSQFNSAGAGQFGSGWAWLIVNGN-GELEITATPNQDNPLMDRGTPILGNDVWEHAYYVTYRNRRADYLDAWWQVVNWDVASERYDAAVA

>A0A401JA31_1559896_Proteobacteria_B_Mar

MIHELPALPYAKDALQPHISAETLEYHYGKHHQAYVTNLNNLIKGN-EFESMTLEEIIKKS-------SGGVFNNAAQVWNHTFYWNCLSPNGGGEPSGALGDAIKAKWGSFDAFKEAFSKAAVGTFGSGWAWLVKTAD-GQIDIVSTSNAATPMTSGQKALMTCDVWEHAYYVDYRNARPKYVEAFWNLVNWKFVAQNFAS---

>A0A401JC34_1559896_Proteobacteria_B_Mar

MEHTLPALPFAMDALAPHMSRETFEYHYAKHHQAYVTNLNNLIKGT-EFESRSLEDIVKSAP------AGGVYNNAAQVWNHTFFWNCLSPNGGGAPTGALADAINARWGSFDAFKTAFQTSAVGNFGSGWTWLVKKAD-GSVDIVNMGAAGTPLATGDKALLCVDVWEHAYYIDYRNLRPKFVETFLNLVNWQFASANFA----

>A0A401ZTK7_2014871_Chloroflexi_B_Ter

MAFELPKLPYDYSALEPYIDTQTMQLHHDKHHATYVTNLNNALQGH-DFANLPIEQVIRRLNEVPESARTAVRNNGGGHINHTMFWEIMTPGGSKTPTGELASAIDATFGSFDAFKTAFNDAGVKRFGSGWAWLVLDKS-GKLAITSTANQDSPFIDGNYPVLGNDVWEHAYYLKYQNRRPEYLGAWWNVVNWDIVGDRYKTALS

>A0A418MLK8_2048547_Firmicutes_B_Ter

MAHELPPLPYAYDALEPYIDEMTMKVHHDGHHGTYVKNLNAALEGHPELQSKSIEDLLHDINTVPESIRTAVRNNGGGHANHSMFWQIMSPNGGGQPSGALADALSTTFGGFEKFKEEFGKAAATRFGSGWAWLVVK-D-SKLSIISTANQDSPYMENQLPIFGLDVWEHAYYLKYQNRRPEYISAFWNVINWAEVEKRYQSAK-

>A0A433MYY1_211165_Cyanobacteria_B_Ter

GEVKLPPLPYTPTALEPYVDAATMRIHHGKHHATYVKNLNAALDKYPQLKNRSVEDLLRNLNSVPEDIRTAVRNNGGGHVNHSMFWRIMKPKGGGEPTGAIASAIKQNFGSFENFKKQFNEAGTKRFGSGWVWLVRNPN-GRLEIMTTGNQDTPLSEGKYPIMGNDVWEHAYYLKYQNRRADYLNAWWNVVNWDEINRRFADAQK

>A0A433N6A6_211165_Cyanobacteria_B_Ter

MAFTQPPLPFPMDALESHMKAETFEYHYGKHHKAYVDNLNKLTEGT-ELANKPLEEVIQISFK--DSSKTGIFNNAAQVWNHTFFWNCLKPSGGGQPTGELGSKIEKDFGSFDKFKEEFSNAAATQFGSGWAWLVDD-N-GTLKVTKTPNAENPLVHGQKALLTLDVWEHAYYIDFRNARPAFIKNFLNLVNWDFAAEQYTKA--

>A0A437M3F2_1979269_Proteobacteria_B_Mar

GPHSLAPLPYAPNALEAAIDAQTMEIHHGRHHAAYVGNLNTLLQGQAELSAMPLDQLVMNLSRAPEAIRTGLRNNAGGHANHTMFWQIMGGQ-GGAPTGEVAEAITRDLGGYDKLRTDFNGAGTRVFGSGWVFVTVSNA-GQLAIVTKPNQDTPLMDGVRVLMGNDVWEHAYYLRYQNRRADYLTAWWNVLDWNKINERYAAAKA

>A0A437MD01_1979269_Proteobacteria_B_Mar

MAFTLPSLGYSTSALAANMCQETLELHHGKHHNAYVTALNGLIESK-GLAGKSLETIVAEAGRA-GADGLPVLNQAGQHWNHVLFWQVMSPSGGGKLPAKLAAKIDSDLGGLAAFKEAFKQAGVTQFGSGWAWLILDAS-GKLKVTKTANGANPISTGETPILGADVWEHAYYLDFRNVRPNYLDNFLKLVAWDVVEELMEKGGL

>A0A497X9T4_1381557_Proteobacteria_B_Mar

MEHTLPALPYAMDALAPHMSKETFEYHYAKHHQAYVTNLNNLIKGT-EYEALDLEAIVKKAP------AGGVYNNAAQVWNHTFFWNCMKPNGGGAPTGALADAINKKWGSLDEFKKAFQTSAVGNFGSGWTWLVKKAD-GSVDIVNMGAAGTPLTTGDKALLCVDVWEHAYYIDYRNLRPKFVETFLNLVNWDFAAKNFA----

>A0A498GYD6_1550565_Methanomicrobia_A_Mar

KKYELPPLPYEANALEPYISQEQLSLHHDKHHQAYVKGANADLEKIEQARRDNAS---IDEK----AILKELSFNIGGHILHSLFWPTMAPAGGGTPGGALADALDREFGSFERFKSEFSQAASSVEGSGWAALAIDEITGRPMVMQIEKHSNNVYPSAPIIMVLDMWEHAYYVDYRNSRADFIDAFWNVVNWDEVNRRIEQRV-

>A0A4D6HF93_1457250_Halobacteria_A_Arc

SEPELPPLPYDYDALEPHISEQVLTWHHDTHHQGYVNGLESAEATLAENRESGDF---GSSA----AAIGNVTHNGCGHYLHTLFWDNMDPNGGGEPSGDLADRIEEDFGSYEGWKGEFKAAAG--AAGGWALLVYDPVAKQLRNVKVDKHDQGALWGAHPILALDVWEHSYYYDYGPDRGSFIDAFFEVVDWDNVAEQYEKTVG

>A0A4D7B288_1940610_Proteobacteria_B_Mar

MTHRIMPLPFKPPRL---LSERLLASHYENNYGGAVRRLNAIEQRLGETD-WSTAPIFD-IN----GLKREELVAANSAILHEIYFDGLG--GSGDAEGDLATALERDFGSVAAWRAQFTAVAKQAGGSGWTLLTWSERHDRLMIQWAADHTNCLA-GGVPILALDMYEHAYHIDFGAKAGAYVDAFMKNIHWERVGTRHGRAIA

>A0A4D7B4V5_1940610_Proteobacteria_B_Mar

MAFELPSLPYATDALAGAMSAETLEFHHGKHHQAYVTALNGFVDND-ALKGKSLDEIVRLSNG--KADLAPVFNNAGQHWNHILFWQALSPKGG-RLPSALETKLIADFGSVEAFKEAFKAQATGQFGSGWAWLVLAKD-GRLKVTKTANGSNPLATDEKVLLGLDVWEHSYYIDFRNRRPDYVTNFLKLANYEFAEAQLKAA--

>A0A4D7B538_1940610_Proteobacteria_B_Mar

GPFALPPLGYGYDALEPNIDTMTMTIHHQRHHGAFIGNLNTFAGQYPALKPDAIETVLRDLAAAPDAIRTGIRNNLGGHWNHVHFWEIMTPGGAKEPGTELATAINGAFGDLGQFRQRFNAAAVGRFGSGWAWLVVDKD-KKLAVVSTPNQDNPLMDGKGVVLGVDVWEHAYYLKYQNRRPDYVTTWWNTVNWTKAGANFTKAMA

>A0A4D7B883_1940610_Proteobacteria_B_Mar

MSFTLDDLPYAHDALQPYMSKETLEYHHDKHHLAYVNNGNNLIKGT-EFEGKSLEDVVKGSFG----KNAGLFNNAGQHYNHIHFWKWMKPNGGGKIPGGLEKAIVDGFGSVDEMKAKFIESCVTQFGAGWGWLAVQ-N-GKVVTMKTPNGESPLVHGAKPILGCDVWEHSYYIDYRNRRPDYAKAFIHLVNWDYVDEMFQAATK

>A0A4P8L1J4_980445_Proteobacteria_B_Mar

AVFKLPSLPYPEDALEPFVSARTLSFHYGKHHQGYVNNLNNLVKGK-AFERMSLEEVIVKTAG--DSGQASVFNNAAQVWNHTFYWNSMQKDGGGPPQGPVAAKISEAFGSYENFRKEFAKAAATVFGSGWAWLVLE-N-GKLKITQTSNADTPAAHGQKAVLTIDVWEHAYYLDYQNQRVDYIQTFLHLLNWDFVAQNLE----

>A0A4P8WH38_88724_Halobacteria_A_Arc

ADHELPPLPYDYDALEPALSEQVLTWHHDTHHQGYVNGLNAAEETLAENREDGDY---SSTP----GALKDVTHNGCGHYLHTLFWENMSPNGGGEPEGDLADRIEEDFGSYEGWKGEFQKAAG--AAGGWALLVYDPVAKQLRNIAVDKHDQGALWGAHPVLALDVWEHSYYYDYGPDRGDFIDAFFDVVNWEKAEEEYQTCLD

>A0A4P8WMJ7_88724_Halobacteria_A_Arc

SDYELPPLPYDYDALEPALSEQVLTWHHDTHHQGYVNGWNAAEETLEENREEGDF---SSSA----GALRNVTHNGSGHVLHTLFWESMSPEGGDEPEGDLADRIAEDFGSYEAWKGEFEAAAS--AAGGWALLVYDSHSEQLRNVVVDKHDQGALWGSHPILALDVWEHSYYHDYGPARGDFVESFFDVVDWEEPSSRYQAAVE

>A0A4Q2ZMN6_2044944_Bacteroidetes_B_Mar

MAYTLPALPYDFSALEPHIDAQTMQIHHDKHHQAYVDNLNKALEG-SEKADMPIEELMATIDQYP----AAVRNNGGGHYNHSLFWEVLGAN-GGEPTGALKTAIDDAFGSLDTLKEKMADAGAKRFGSGWAWLIVK-D-GKLAVTSTPNQDNPLMPQGTPIFGIDVWEHAYYLNYQNKRPDYLKAIWNVVNWNEVARRYEAASG

>A0A4Q3S5H0_1978230_Proteobacteria_B_Mar

--FILPDLPYARDALSPIVSGETLDYHHGKHHKTYVETLNKLLAEK-GETPESLEAVVKSA------GPGKLFNNAAQAWNHGFFWESMTPS-PAQPSGALAEAIEA-FGGHAALGEKFVETGVGQFGSGWAWLVWK-D-GKIAVAPSHDADTPLANGAFPLLVCDVWEHAYYLDHQNDRKGFLTAWFKLANWEFAGVQLAAAQG

>A0A4Q5RR52_1978525_Proteobacteria_B_Mar

MAFELPPLPYAKDALAPHMSPETLEFHHGKHHKAYVDKTNGFVADK-GLGGKSLSEVILHAKE---TGDKGLFNNSAQIWNHSFFWQCLTPNYA-APAGKLADLITEGFGSPEELVKKLVAESTNHFSNGWGWLVLE-D-GKLKVTSLHDADSPVAEGMVPLLTIDVWEHAYYIDYRNARPKYLETVTKLINWAFVEQNLDGQGV

>A0A4Q5S3N2_1978525_Proteobacteria_B_Mar

AGFVLESLPYTANALEPAIDAQTMTIHHGKHHQAYVDNLNKAVAVDPTLADQTLGELVAKAGSLP----AAVRNNAGGHWNHNFFWRTMAPPAEGTPSPALLAAIEASFGSLDQFKAAFRDSGTKRFGSGWVWLIVGGD-GKLAIASTPNQDNPLMDRGVPILGNDVWEHAYYLQYQNRRGDYLDGWWQVVNWAEVSRRYAAAAA

>A0A4Q5V9J9_1913989_Proteobacteria_B_Mar

MAFELPALPYAKDALAPHISAETLDFHHGKHHKTYVDKLNGLVPGT-EFEGKSLEEVIKSS-------SGPVFNNAAQIWNHTFYWHCLSPNGGGAPNGPVAEAINKAFGSFDKFKEDFNTSAVNNFGSSWTWLVKKAD-GSVAIVNTSNAGTPLTDSVTPILTVDLWEHAYYIDYRNARPTYLNAFWSLVNWEFVNANFAK---

>A0A4Q5XXF3_1913988_Proteobacteria_B_Mar

MAFTLPALPYARDALAPHISENTLNFHYGKHHQAYVDNLNKLVAGT-PLENAPLEEVVKQSWA--E-KKAPIFNNSGQVWNHTFYWHSMKPGGGGKPTGLVADRINKDFGSYDEFKRLFAEAGATQFGSGWAWLVLK-D-GKLSVTKSPNAETPLTEGVTPILTMDVWEHAYYLDTQNARPKYIETFVELVNWDFANQNLSDAK-

>A0A4Q6B980_1977087_Proteobacteria_B_Mar

MAFKLPELPYAKDALAPHISAETLEYHYGKHHQAYVDNLNKLVAGT-PNENKSLEELVKTT-------EGGIFNNAAQVWNHTFYWYCLKPKGGGAPTGKIGDAITKNFGSFDTFKEQFSEAAKTQFGSGWAWLVKKAD-GSLAIEKTANAGNPMTSGLKPLMTCDVWEHAYYIDYRNARPKYVESFWNLVNWDFVEQQLG----

>A0A4R1JLY6_412034_Proteobacteria_B_Mar

MSYSLPELPYAYDALEPHIDARTMEIHHTRHHQTYVNKLNAAIEG-SELAKLPVEALLGRIDELPEDKRQAVINNGGGHANHSLFWTVMSPNGGGEPTDVVAQAITKDLGGFAAFREAFTQSAVARFGSGWAWLSVSQN-NTLVVENSLNQDSPLMHSHTPILGLDVWEHAYYLKYQNKRPEYIQAFFNVINWPEVEKRYLAAIA

>A0A4R1R4E5_1469948_Firmicutes_B_Ter

--YKQYQLTYDFNSLEPYIDALTMETHYSKHHAAYTKNLNDAVEK-AGIENKSIETLLASLGKIGDDLRTAIRNNGGGFYNHNLYFSTMSPNGGGEPEGDLAKAIADSFENFSLFKEKLTALALGQFGSGWAWLSAGPN-GELKLSASPNQDNPIIE-YIPILGIDVWEHAYYLKYKNIRADYVKEYFNVIDWKSVAEYYKRARD

>A0A4R2GX74_659006_Proteobacteria_B_Mar

MTFKLPELPYAYDALQPYMSKETLEYHHDKHHQAYVDTGNKLLAGT-ELEGKSVEEIVKASYG----KNQPLFNNAGQHYNHLHFWNWMKPNGGGAIPGAIEKKIVSDIGSVEKFKEDFVQAGVGQFGSGWAWLALK-N-GKLEIMKSPNGENPLVHGATPILGVDVWEHSYYIDYRNRRADYLKAFVNLVNWEYVEKLFNEAT-

>A0A4R2PEU5_1188247_Proteobacteria_B_Mar

-MLTLPDLPYDYKALEPYMSAETLEYHHDKHHNAYVTAGNKLMEGT-PYADMDLESAIRESFK----SDPKLFNQIAQHWNHMEFWHWMSPKGGGAIPGNLESQIKKDFGSVDDFKEAFVQAGVTQFGSGWCWLVWK-D-GKLAVTQTPNGENPLVHGGTALLGCDVWEHSYYIDYRNARPKYLEAFFHLVNWERVTEIYEQAS-

>A0A4R6BX88_198484_Firmicutes_B_Ter

MAFELPQLPYAYDALEPHIDKETMEIHHTKHHNTYVTNLNAAVAG-TEFENQSIEELMANINSVPEDKQTAVRNNGGGHYNHSLFWQLLSKD-GGTPNGEVAEAINAKFGSFEAFQEAFAEAATKRFGSGWAWLVVN-N-GELEVVSTPNQDNPLMEDKTPILGLDVWEHAYYLNYQNKRPDYIKAFWNVVNWDKVNELYTAAK-

>A0A4R7K0K2_332522_Proteobacteria_B_Mar

MAFELPKLPFSENALAEKMCQETLELHHGKHHNAYVTTLNTLVEDS-NLQGKSLEEIVKLSYG--RDDLQKVFNQAGQHWNHNLFWENLSPKGGG-IPGKLEQKINEDFGSVDTFKEEFKTAATGQFGSGWAWLIVGSD-GKLKVTKTPNAVNPLATGEKAILGLDVWEHSYYLDFRNRRPDYVMNFLNLANYEYAEELLNKA--

>A0A4R8ITH7_381308_Proteobacteria_B_Mar

QIHVLEVLPYAVNALSPVISSNTVEVHYGKHHKGYVDKLNKQVEGT-EFSDMSLESLITGTAG--KPDHVGIFNNAAQAWNHTFYWRSLKQKGGGEPPAMLKDKIEASFDSLDACREELVKAATTRFGSGWAWLVQD-G-DQIKVVSTGNADNPIANGMTPLLTIDVWEHAYYLDYQNRREEYVRAVLKLINWEFAAENLD----

>A0A4V2PNM7_412034_Proteobacteria_B_Mar

MAFELPALPYAKNALEPHISAETLEYHHGKHHQTYVTKLNGLIEGT-EFEGKSLEEIITSS-------TGGVFNNAAQHWNHSFYWNCLSPK-GGQPAGALADAINAEFGSFDAFKEQFNTSAAGNFGSGWTWLVKTKD-GKLAIVNTSNAANPMTDGATPLLTCDVWEHAYYIDYRNSRPNYLKGFWELVNWEFVGENFAK---

>A0B701_349307_Methanomicrobia_A_Arc

KFYTLPELPYGAKDLEPYISEAQLKLHHDKHHLAYVNGANAILEKLDKARREGAD---IDQK----STLKELSFHIGGHLLHSLFWANMRKPVG--PVGALADEIKDEFGSFERFRKEFTAAAVSVEGSGWAALAYCMQTRRPIIMQIEKHNTNICPMFRLLLVLDVWEHAYYLDYRNDRAKFVEAFWQIADWEEANRRFESLIG

>A0YJL0_313612_Cyanobacteria_B_Ter

MAFELPELPYPADALESSMSKQTFSFHHGKHHAAYVNKLNGMIEGT-DLANKSLEEIVKATAN--DPSKSGIFNNAAQVWNHSFFWKCMKPGGGGKPSGALAEKINADFGSYEKFAEEFKNAGATQFGSGWAWLVLE-N-GSLKVKKTLNAINPIIEGVTPLLTMDVWEHAYYLDFQNARPGYIDNFLKLVNWDFVAENLAAAQ-

>A1RW25_384616_Thermoprotei_A_Arc

KKYTLPNLPYAYNALEPYIAEEIMRLHHQKHHQGYVNGANAALEKLEKFRRGEAQ---IDIR----AVLRDLSFNLNGHILHSIFWPNMAPPGGGKPGGRTADLINKFFGSFEKFKEEFSLAAKNVEGVGWAILVYEPLEEQLLVLQVEKHNLMHAAGAQVLLALDVWEHAYYLQYKNDRGSYVDNWWNVVNWDDVERRLEKALN

>A1ZNK9_313606_Bacteroidetes_B_Mar

IEQKLPELPYDKNALNPIISEETFDFHYGKHHAAYVNNLKGMVEGT-VLEEAGVKEVIQKAHQ--D-KNLGLFNNAAQHWNHSFFWHCLSPNGGGTPQGEIKKMIDRDFGSFDAFKSKFSETAVKLFGCGWAWLAQGEQ-GKLEIIPMKEAYTPLTENKTPILTLDVWEHAYYIDYRNARPKFVEGFWEIVNWDFANKNLQ----

>A1ZTZ0_313606_Bacteroidetes_B_Mar

MAFEQKALPYAYNALEPHFDAQTMEIHYGKHHAGYTSKLNAAIEG-TDLANHSIEDILKNVGSHS----TGVRNNGGGFYNHSLFWEILSPE-GGVPSGDLEAAIKEAFGSLNGLKEKFNAAAATRFGSGWAWLIVGED-GKLHVTSTPNQDNPLMDKGTPILGLDVWEHAYYLKYQNRRPDYISAFWNIINWEKVAEKYKAAEK

>A3J209_391598_Bacteroidetes_B_Mar

AQFSQKALPYGYSDLEPFVDAQTMEIHYSKHHAGYVKNLNKALEGTAD-EKLSLTEILAKVSIL----STAIRNNAGGHYNHELFWSVLTPEKNTKMSAELEKAVIETFGSFEVLKEKLNAAGATRFGSGWAWLVVTKE-GKLVISSTANQDNPLMDKGTPIFGIDVWEHAYYLKYQNKRADYLSALWNVVNWTEVSKRYKAAMP

>A3J2V1_391598_Bacteroidetes_B_Mar

MAFELPQLSYAFDALEPHIDAKTMEIHHSKHHNAYVTNLNAAIAG-TELEGKSIEDIMKNLDMNN----MAVRNNGGGHFNHTLFWEIMSPNGGGLPTGELATAIDAAFGSFDAFKAEFSKAGATRFGSGWAWLCVK-D-GKLEVCSTPNQDNPLMPGGQPILGMDVWEHAYYLNYQNRRPDYMEAFFNVINWTEVAKRFSASK-

>A3J5A6_391598_Bacteroidetes_B_Mar

GIFQMRGLKYAYDGLEPHIDGRTMEIHYSKHHLGYANKLNKAVLG-TDLELKTVEEILKNLDT----NNKEIRNNAGGYYNHNLFFEILNPKGGGTPNGALAEAITTEMGSFDNLQKQLTEAAARQFGSGWAWLVVTRD-GKLAITSTANQDNPLMPRGTPILAIDVWEHAYYLNYQNKRSEYLTSIFNVIDWNIVNAKYETAVS

>A4BGS7_314283_Proteobacteria_B_Mar

MAFELPELPYAKTALEPHISAETLEFHHGKHHATYVTKLNGLIPGT-EFEGKSLEDIIKSS-------SGGVFNNSAQIWNHTFYWHSLSPNGGGQPTGALAEAINAKWGSFEDFQKAFDEKAVNNFGSSWTWLVKNSD-GSLDIVNTSNAGTPITEGQTPIITVDLWEHAYYIDYRNARPKYLEAFWALANWEFAAENFAK---

>A4YHX7_399549_Thermoprotei_A_Arc

KKFELPPLPYKVDALEPYISKDIIDVHYNGHHKGYVTGANTFMERFNKVIKGELQSGQYDVQ----GLMRGIVFNINGHKLHALYWDNMAPAGGGQPGGALADLIVKQYGSYDRFKQVFTETANSLPGTGWTVLYYDTENGNLEIMTFENHFMNHIAELPILLILDEFEHAYYLQYKNKRADYVTNWWNVVNWDYAEKKLQKYLK

>A5UY95_357808_Chloroflexi_B_Ter

MAFTLPPLPYDFSALEPHIDTMTMQIHHGKHHQAYVTNLNAALEGHPSLQNATIEEILSNINDVPESIRQAVINNGGGHHNHTLFWNIMGPNGGGEPTGALARAINDTFGSFAEFKAKIKDAGIKRFGSGWAWLVKDKD-GKLHIYSTPNQDSPLMQGHIPILGVDVWEHAYYLKYQNRRPDYIDAWWNTVNWEAVAARFEQ---

>A6EQH5_50743_Bacteroidetes_B_Mar

MAFELPKLPYAHDALAPHIDEQTMQIHHGKHHQGYTNNLNAAIEG-TDLEGKTIENILINLDMDN----KAVRNNGGGFYNHSLFWEVMSPNGGGEPTGEIMSAIKSSFGSFADFKEKFSTAAKTQFGSGWAFLCVKED-GSLDICGCPNQDNPLMPGGQPILGLDVWEHAYYLNYQNKRPDYVSAFFNVINWDEVNARFLKSK-

>A7HBL1_404589_Proteobacteria_B_Mar

------VKRYTPSKVRG-ISDKQLEEHL-KLYEGYVKRTNALTEKLQAICADGKASADPVFA----ELTRRLGFEYSGMVNHEYYFDNMAPGAQAEPGGKLRKALEASFGKYETWLADFRAIAT-MPGIGWAMTFQDPTTGWLSNHWINLHQDNVPVGFKPVLVMDGWEHAYMRDYATERAKYVDAFFKNVSWEAVEKRIA----

>A7I8G0_456442_Methanomicrobia_A_Arc

KFYTLPKLPYDYGALKPYISEEQLKLHHQKHHQAYVNGANAIYEKFDKARKENAD---FDVK----ATLKELSFHLGGFKLHNLFWENLAPAGGGMPKGALAEALDAEFGKFDRFKKEFTQAATGAEGSGWAALTFCKKTGRPLIMQIEKHNTNVFPGFGIIMVLDVWEHAYYLDYKNDRAKFVDAFWNIVNWDEIAKRFDHIRK

>A7I921_456442_Methanomicrobia_A_Arc

KLYTLPKLPYDYAALEPYISKAQLTLHHTKHHQAYVTGANAIFEKLDKARKEKTD---LDQK----ATLKELSFHIGGFRLHDIFWENLAPAGGGQPEGELAKAIDAEFGSFEWFKKEFTAAASSVEGSGWAVLTASTTTPRLLINQIEKHNVNVYPGFRILLVLDVWEHAYYLDYKNDRAKYIEAFWNLTNWDAVNKQFITHLK

>A8FE63_315750_Firmicutes_B_Ter

GGHQLPPLPYRYDALEPFISKEIMYLHHQKHHQSYVDGLNQAELALKRARRTNDF---KMIK----HWERELAFNGAGHYLHCIFWFSMSPSGKRKPTGQMLRQIEQSFESYDAFKAQFSAAAKQVEGVGWAILVWAPRSQRLEILQAERHQFLSQWDVIPLLALDVWEHAYYLQYLNEKAAYVDRWWNVVDWHEPEARLKQAQQ

>A8FF82_315750_Firmicutes_B_Ter

MAFKLPELPYAYDALEPHIDKETMTIHHTKHHNTYVTNLNKAIEGVSALEDQSIEELVANLNSVPENIRTAVRNNGGGHANHSLFWTLLSPNGGGAPTGELADAIEKELGGFEKFKSDFAAAAAGRFGSGWAWLVVN-N-GKLEITSTPNQDSPLTEGKTPILGLDVWEHAYYLNYQNRRPDYISAFWNVVNWDEVARLYSEAK-

>A8H3Z8_398579_Proteobacteria_B_Mar

MAFELPALPYAKNALEPHISEETINFHYGKHHNTYVVKLNGLVEGT-ELEQKSLEEIIKTS-------TGGIFNNAAQIWNHTFYWNCLSPNGGGEATGPVAEAIIAAFGSFEAFKAQFTDSAVNNFGSAWTWLVKNAD-GTVAIVNTSNAATPLTDGVTPIMTVDVWEHAYYIDYRNVRPDYLAHFWQLVNWDFVNANFAA---

>A8MAM7_397948_Thermoprotei_A_Arc

KRYELPPLPYNVNALEPYISGQVIDVHYNGHHKGYVNGANAAIDRLEKIIKNEVTS--YDIQ----GLLRNLFFNINGHKLHTLYWNSMAPAGGGTPGGYLGDLVIKQFGSYDKFRNLFNEVMRSLPGSGWAVLYYDTETGNLVFTTFENHYNQHIAELPVILIIDEFEHAYYLQYKNNRNAYLDAIWNVLNWGEAENRLRKYIK

>A8PPX3_59196_Proteobacteria_B_Mar

MKHQLPPLPYSLEALAPIISKETLEYHYGKHHKAYVNKLNELIPNT-TFETLSLENIIKKA------PKGPIFNNAAQTWNHTFYWHCMTPLAKNKLKGALEESICKSFGSFSQFKEAFTNAAIAQFGSGWAWLIKDAH-G-LKIETTSNALTPLSEDKICLLTCDVWEHAYYIDYRNARPHYLEKFWDIVNWDFVAENFEKQ--

>A8U258_331869_Proteobacteria_B_Mar

MAFELPPLPYAYDALAPYMSAETLEFHHDKHHNAYVVNGNNLLKDS-GLEGKSLEEVVKASYK--NDKLAGLFNNAAQHWNHIEFWQWMKPKGGGAMPGELEKRIAADFGSVAKFKEDFVNAGVTQFGSGWCWLAVGSD-GKLKVTKTANGVNPVALGETALLGCDVWEHSYYIDYRNRRPDYVKAFLSMVNWERVAELLGKAS-

>A9AWV1_316274_Chloroflexi_B_Ter

MAFELPALPYATDALEKAIDAQTMEIHHGKHHAAYVNNLNAALEKYPELQSKSLHELISDLNSLPEDIRTAVRNNGGGHANHSLFWNILSPNGGGEPTGKLGDEINATFGSLDAFKEQFAKAATTRFGSGWAWLIRTSE-GKLAVTSTANQDSPVMEGQTPLLGLDVWEHAYYLRYQNRRPDYIKTWWEVLDWNKVAEN------

>A9AXW8_316274_Chloroflexi_B_Ter

GKFTLPALPYDYGDLEPHLDAETMYIHHQKHHQALVTNLNAALADYPKLQSMNVAELLHGLSSLPSAIQTAVRNNAGGHANHAIWWATLSPKGGGNAGGALAAAINAKYGSFNSFKTTFNDIAARRFGSGWGWLSTDSK-GTLYLASYPNQDSPYMEGLTPLLGVDVWEHAYYLKFRQKRAEFLSAWWNVINWGEVGRRYDLAIA

>A9EWL0_448385_Proteobacteria_B_Mar

MTFTLPELPYSRDALVPHISAETLDFHHGKHHNAYVTKLNELVAAA-ALAGKSLEDLVRTT-------SGAVFNQAAQVWNHTFYWHSMKPQGGGEPPAALRAAIDEAFGSVSSFKEKFTAAAIGQFGSGWAWLVKNGS-GKLEIVQTGNAGSPLTEGKTPLLTCDVWEHAYYIDYRNARAKYVEAWWNLVNWDHAASKL-----

>A9GL55_448385_Proteobacteria_B_Mar

-------MSREPDNLLGGLSDLQLKAHF-TLYQGYVKKLNEIWEQLGKADRSAPNYSYNAYS----ELKRREPVAFNGTVLHELYFENIGN-GSTQPSAQSKKLIEASFGSFDAWLADAKASLL--SAHGWTVLVYDYQEQKLFNNLVRTHDVGLFANTHIMVAIDAWEHAYFADYQTKKADYVANVLSGLNWDVINARLAQIGP

>A9WJC9_324602_Chloroflexi_B_Ter

MAFEVPPLPYDYNALEPYIDEATMHYHHDNHHQTYVTNLNNALANYPELQSKTIEELLGNLDAIPEAIRTAVRNNGGGHWNHTFFWEIMAPNAGGAPTGDLAAAIDAAFGSFDAFKEKFKAAALGRFGSGWAWLVAAKD-GSLSIMSTPNQDNPLMEGKTAILGLDVWEHAYYLKYQYKRAAYVDAWWNVVNWAKVADHYAAAKG

>B0JGF5_449447_Cyanobacteria_B_Ter

MAYTLPPLPYDYTALEPCITKSTLEFHHDKHHAAYVNNYNGLVKDT-ELDALSLEEVIVKVAG--DASKAGVFNNAAQAWNHSFYWDCMKPGGGGAPTGALADKIQADFGSFEKFVEAFKTAGATQFGSGWAWLVLD-N-GTLKVTKTGNADNPLTAGQVPLLTMDVWEHAYYLDYQNRRPDYISDFLKLVNWDFVAANLAAA--

>B3EJL9_331678_Chlorobi_B_Mar

MAYTQPALPYAENALAPHISAETIGFHYGKHHATYISKYNGMVAGT-PFDDQTIEDVIVATAN--DPEKAGLFNNGAQAWNHSFYWNSLSPDGGGAPGGQIGEKINADFGGYDKFREELANAAATQFGSGWAWLVLD-K-GQLKIVKTANAQTPLTNGMFPILTIDVWEHAYYLDYQNRRPDYVSAVIELLNWNFAASNFEAAQV

>B3ER22_452471_Bacteroidetes_B_Mar

MAFELPTLPYAPEALEPHIDATTMQLHHAKHHQAYLDKFNKAIEG-TDLAARSIEDILKNVSNY----SVVVRNNGGGYYNHNLFWPMLTPNGGGEPTGAIVDAINQKFQGFAQFKEAFAQAALEQFGSGWAWLCKDIT-GELFICSTANQDNPIMDRGIPVLGLDVWEHGYYLKYQNRRAEYIDAFWNIVNWEEVNKRFQAPMP

>B4U6J9_380749_Aquificae_B_Anc

--MKLE----PKNHLKPSISNEQIEPHFEAHYKGYVAKFNEIQDKLADSDRAKANQNYSEYR----ELKVEETFNYMGTVLHELYFGHLTP--KGTPSEALKKKVEEDFGSWDNCVTELKAAGI--AFRGWAILGLDIFSGKLMINGLDAHNLYNLTGLIPLIVLDTYEHAYYVDQKNKRPPYIDAFLNSLNWDVINERFEKAIK

>B5JWC0_391615_Proteobacteria_B_Mar

MSFELPTLPYEKNALEPTISAETLEFHHGKHHAAYVNKLNDMIQGT-EFENLSLEEIIQKS-------SGGMFNQAAQIWNHTFYFTGLSPNGGGEPTGALADAINAEFGSFDEFKAKFNAAGAGNFGSGWTWLVKNAE-GKLEIVNTDDAETPLTNGQTPVITVDVWEHAYYIDYRNARPKYLEEIWNIIDWSVAAERFGA---

>B7KAA9_65393_Cyanobacteria_B_Ter

MAYKLPDLPYDYTALEPHISKSTLEFHHDKHHAAYVNNFNKAVEGT-ELDSMSIEDVIKKIAG--DSSKTGLFNNAAQAWNHTFYWNCMKPNGGGTPSGALADKINADFGSFDKFVEEFKNAGGTQFGSGWAWLVLD-N-GTLKVTKTPNADNPLTSGQVPLLTMDVWEHAYYLDYQNSRPGYMEKFINLINWDFVAENFSKA--

>B7KHG4_65393_Cyanobacteria_B_Ter

EPFKLPPLPYSYNALEPYIDEETMRFHHDKHHAGYTKKFNAAISKYPDLKIQSAEELLSNIDKLPKNIQTTVRQNGGGYLNHAIFWEIMSPNGGGQPTGEIAEAINQEFGSFEAFKNAFNEAGNSRFGSGWAWLVLDKK-GKLQVVSTPNQDSPLMEGMYPIMGNDVWEHAYYLKYRNDRGQYLQQWWNVVNWNEVNKRFLLVKV

>C0BHJ2_487796_Bacteroidetes_B_Mar

MAFELPSLSYAYDALEPNIDSRTMEIHHSKHHNGYTTNLNNAIAG-TPLADQSIEEILKGLDLNN----KAVRNNGGGYYNHRLFWNIMSPNGGGLPTGTLATAIDSAFGSFDNFKSEFAKAAATQFGSGWAWLCVHVD-GTVKVCATANQDNPLMPGGTPILGIDVWEHAYYLNYQNRRPDYIQAFFNVIDWNKVNELFTKGA-

>C1DW98_204536_Aquificae_B_Anc

KVVKLQ----PKDHLKPKISDEQIEVHFEAHYKGYVAKYNEIQEKLASADRSKANQNYSEYR----ALKVEESFNYMGVVLHELYFENLVAGGKGEPSPELKKMIEEYFGSVNNCINEIKATGI--ACRGWATLSYDLYNKILVVNGFDAHNQYGFVYSVPLIVLDVYEHAYYVDQKNKRPPYIDAFFKNLNWEVVNERFNKAVK

>C7LNS7_525897_Proteobacteria_B_Mar

APIEIAALPYAENALEPVISAKTIGFHYGKHHMGYLGNLNKLIAGS-EYADMPLEKIITETSG--KADKIAIFNNAAQTWNHSFYWQSLSPKGGGTPPADLQNRIKDSFGDLDTCLKDLSAAAVGRFASGWAWLVAD-G-DELKVMNTMNADTPITSGLKPLLTIDVWEHAYYLDYQNRRADYVKAVLKLINWEFAAKNLG----

>C7LRC1_525897_Proteobacteria_B_Mar

SLQVLPFLPYAENALEPVISAKTIGFHYDKHHKGYVDNLKKLVAGT-RFADMTLEKIMSETAG--KSDNIAIFNNAAQTWNHNFYWQSLRPSGGAELAAVLQKKIEVSFGSLDACKKELAAAAMAQFGSGWAWLVMD-S-DMLKVVKTANAEVPMTMSMKPLLTIDVWEHAYYLDYQNRRADYVKAVLTLINWEFAAENLGC---

>C7LSI9_525897_Proteobacteria_B_Mar

MIFVLPDLPYSKDALSPCISAKTLDFHHGKHHQLYIDNTNKLIAGT-DLEGQTLREIVMATAN--DPAKAGIFNNAAQVWNHSFYWRCMKAGGGGAPTGAVAEGINKAFGNYENFAKAFKEAGMTQFGSGWAWLVEK-N-GKLEIMKTGNADTPMAHGAKALLTADVWEHAYYLDYQNRRADYLQDFLKLINWEFVNQQLAK---

>C7NYW1_485914_Halobacteria_A_Arc

SDPELPPLPYDYDALEPHISEQVLTWHHDTHHQGYVNGLAAAEETLAENRESGEF---GSSA----GALGNVTHNGSGHYLHTLFWDNMSPNGGGEPSGELRDRIEADFGSYEGWKGEFEAAAS--AAGGWALLVYDPVAKQLRNVTVDKHDQGALWGSHPIMALDVWEHSYYYDYGPDRGSFVDAFFEVVDWDEVASQYETAVG

>C7P4U1_485914_Halobacteria_A_Arc

SEHELPPLPYDYDALEPHISEQVLTWHHDTHHQGYVNGWNAAEETLEANREAGDF---SSSS----GALRNVTHNGSGHVLHDLFWQSMSPTGGDEPSGALADRIEEDFGSYEAWKGEFEAAAS--AAGGWALLVYDSFSNQLRNVVVDKHDQGALWGSHPVLALDVWEHSYYHDYGPARGEFVDNFFEVVDWEEPSQRYEQVVE

>C7RD31_523791_Proteobacteria_B_Mar

MAIELPALPYDRDALAPHISEETINYHYGKHHQAYVTNLNKMIDGT-DFAGKSLEDIIRTS-------EGGVFNNAAQVWNHTFYWNSLSPNGGGEPSGALADAINSAFGSFADFKEKFTASAAGNFGSGWTWLVKNSS-GNLEIVNTSNAGTPITDGVTPLLTVDVWEHAYYVDYRNARPEYLKHFWELVNWDFAAKNFA----

>C7RIZ7_522306_Proteobacteria_B_Mar

MEHQLPQLPFAMDALAPHMSRETFEYHYAKHHQAYVTNLNNLIKGT-EYEALDLEAIIRKAP------AGGVYNNSAQVWNHTFFWNCLTPNGGGAPGGALAAAIDAKWGSFAEFSKAFQTSAVGNFGSAWTWLVKKAD-GSVDIVNMGAAGTPLTTGDKALLCIDVWEHAYYIDYRNLRPKYVETFLSLANWRFAEQNFAG---

>C8Q0X7_553217_Proteobacteria_B_Mar

MSITLPDLPYAKNGLEPHISAETLEFHHDKHHAAYVNKLNELLPGS-GLEGKELDEIIKATAD--DSSKATIFNQAAQVWNHTFYWNCLTANGGGEPTGDLKAKIEEAFGSYAKFREEFKNAAVSQFGSGWAWLVADSG-GKLSIIKTANADTPLAHNQVAVLTCDVWEHAYYIDYRNRRPDYVDTFLKLVNWDYANAKYKGQEA

>D0J9E3_600809_Bacteroidetes_B_Mar

MSFKLPKLSYSYKDLEPYIDQKTMEIHYTKHHAGYTNNLNKAIEG-TDLTHLSIEEILRRSNI----EKPIIRNNGGGFYNHNLFWEILIPHDYTHPSKYLKNAIEKNFKSFDAFKEKFTSIAMNRFGSGWTWLCVK-E-KRLTICSTANQDNPVMLEGVPILGLDVWEHAYYLQYQNRRADYISSFWNIINWKKVEENYKKSIE

>D0LUJ8_502025_Proteobacteria_B_Mar

MAFELPKLPYAEDALEPHISKETIQYHYGKHHQGYVNKVNAAVENT-PNADLSIDELVRKVAG--DAGSKKLFNSAAQVWNHTFYWNCLSPNGGGEPTGAIAEAINKSFGSFADFKSKFSDAAAGQFGSGWAWLIKGDD-GGVSIVTTSDAETPMAEGKTCVLTIDVWEHAYYVDYRNARPKYIEAFWNIVNWDFVNQQLA----

>D0RR50_684719_Proteobacteria_B_Mar

MAFELPKLNYANDALAPVMSQETLDLHHGKHHQTYVTNLNNLVKDT-DLADASLEDIIKKTAK--DSSKAGIFNNAGQHWNHILFWKCMKPKGGGAIPSELEKRIVSDFGSVDKFKEDFIQAGVTQFGSGWAWLAID-N-GKLVITKSANASNPLVDNMKPILGCDVWEHSYYVDYRNRRPDYLKAFIDLVNWEHVASLLD----

>D2RVX0_543526_Halobacteria_A_Arc

TDHELPPLPYDYDALEPALSEQVLTWHHDTHHQGYVNGLNSAEETLAENREEGDY---SSTP----GALSDVTHNGCGHYLHTLFWENMSPNGGGEPEGDLADRIEEDFGSYEGWKGEFQKAAG--AAGGWALLVYDPVAKQLRNLAVDKHDQGALWGAHPVLALDVWEHSYYYDYGPDRGEFIDAFFDVVNWEKAEEEYQTCLD

>D2S0I8_543526_Halobacteria_A_Arc

GRYALPELPYEYGALEPHIDERIMELHHSEHHQGYVDGANDALDAFEEMRSDSNF---EDIK----AVKRDFSFNLSGHVNHTIFWQNMSPDGGGAPDGEFRSALEDQFGSFEAFQNEFSTAAENVESNGWAMLFYEPIADLLVIGQVESQNGLAHQGAIPILTLDVWEHAYYLQYENERASYIEEWWNVVDWTDVCQRYEFLTQ

>D3FDM4_469383_Actinobacteria_B_Ter

MAYELPALPYAYDALEPHIDEATQKFHHDKHHATYVARANEALEG-TEWADKPVEELLKNLDKLPADKLNPVRNNAGGHYNHTLWWESLSPNGGGAPTGDLAAAIDAAFGSFDEFKAKVEAAGAGRFGSGWAWLVKG-D-GGLEVTSTPNQDTPLADGKTPLFGIDVWEHAYYLKYQNLRPAYLKAVWNVVDWNKVAERFAAAS-

>D3SY04_547559_Halobacteria_A_Arc

TDHELPPLPYDYDALEPSISEQVVTWHHDTHHQGYVNGLNSAEETLAENRESGDF---DSTP----GALSNVTHNGCGHYLHTLFWENMSPDGGGEPAGDIADRIEEDFGSYEGWKGEFEAAAG--AAGGWALLVYDPVAKQLRNVAVDKHDQGALWGSHPILALDVWEHSYYYDYGPDRGSFIEGFFDVINWDSVEDEYQKCLD

>D5BTR8_488538_Proteobacteria_B_Mar

MAFTLPDLPYSHDALAGLMSAETLEYHHDLHHNAYVVNGNKLVAGT-EWENKSLEDIITGTYQADAVAQNGIFNNASQHWNHMQFWEMMGPGKT-AMPSELEKALVESFGSVDAFNDAFKAAGASQFGSGWCWLVKNAD-GGLQVTKTENGVNPLCFGQTALLGCDVWEHSYYIDFRNKRPDYLSNFLNLVNWEFVAAQL-----

>D5VB54_1236608_Proteobacteria_B_Mar

MAFTLPELGYSYDALEPHFDKETMEIHHSRHHQAYVNNANGLLEG-TQWADKSAEEVIANLDQIPADKRTGVRNNAGGHANHSLFWTILKT--GTTLGGSLKDAIVRDFGSVEAFQEQFEKAAASRFGSGWAWLVLD-E-GSLKVVSTANQDSPLMGKGYPIIGLDVWEHAYYLKYQNKRPDYIKAFWEVVNWDEAQKRFDSAN-

>D6TCG4_485913_Chloroflexi_B_Ter

KGFQLPPLGYAYNALEPYIDAQTMQLHHDKHHATYVKNLNDALKNHP-FASLPIEEVLRRINELPENIRTAVRNNGGGHANHSMFWRIMKPKGGGEPAGELASAIKANFGSFASFKEAFNTTGSKVFGSGWVWLVSDKS-GKLQITSAANQDSPLLNGLYPVMGNDVWEHAYYLKHQNRRADYLNAWWNVTNWDEIAKRYGQAKN

>D6TP22_485913_Chloroflexi_B_Ter

MAFELPPLPYDYKALEPYIDTQTMQLHHDKHHATYVTNLNNALQGH-EFANLPVDEVLRRINEVPESARTAVRNNGGGHSNHTMFWQIMGPNAGGQPTGEIANAINAKFGSFDNFKNAFNDAGAKRFGSGWAWLVIDRN-GNLEVISTANQDSPLMESLFPVMGNDVWEHAYYLKYQNRRPEYLNAWWNVVNWNEVNKRYAQARG

>D6U4H7_485913_Chloroflexi_B_Ter

MAFQLPPLPYDYQALEPYIDTQTMQIHHDKHHATYVSNLNAALEGH-EFASLAVEQVLARLNEVPEAKRTAVRNNGGGHANHTLFWETMTPGGSKEPTGTLAQAITSTFGSFDAFKAAFNDAGAKRFGSGWAWLVLNKN-GQLEVTSTPNQDSPLLDSNVPLLGNDVWEHAYYLKYQNRRPEYLSAWWNVVNWDIVGRRFEQATN

>D6YU13_716544_Chlamydiae_B_Mar

NTYTLPDLPYDLGDLEPVINAEIMDLHYNKHHKTYINNLNNLLEQLEEAQSKKDI---SKEI----SLQSGINFNGGGYINHSIFWTNLAPKGGGEPSGPLADAIMKDFGSLDKLKETMTAKTVAVQGSGWGWLGYNKSEDRLEIAICQNQDPLAAKGLVPLLGIDVWEHAYYLQYKNVRPDYVKAIWEIVNWKNVSERYEAAL-

>D7CR17_649638_Deinococcus-Thermus_B_Ter

YPFSLPELGYAYGALEPVIDAETMTLHHQEHHQSYVDALNAALADYPELQGRTLGELLTTLPELPEEVRQDVQNQGGGHLNHALWWRWVAPGGSREPVGRSAERIAETFGDLEGLKEPFNAAADARFGSGWAWLVVDES-GRLSVLSTPNQDHPISQGLVPLLGLDVWEHAYYLSYRNRRPEYINAFWEVVNWDAVEEQHGVAAA

>D7CSR0_649638_Deinococcus-Thermus_B_Ter

MAFNLPDLPYPTDALEPHIDAKTMEIHHDKHHGTYTTKLNDAVQG-TELEGLSIEEILRKAENLP----AAVRNNGGGYYNHNLFWEWLSPQGGGEPTGELAEAIARDFGSFAAFKEKFSEAAANRFGSGWAWLVSE-N-GKLSITSTPNQDNPMMEGKHAILGLDVWEHAYYLKYQNRRPEYIQAFWNVVNWPKVSELYAQTK-

>D7CXL9_649638_Deinococcus-Thermus_B_Ter

YPFALPELPYPYDALAPAIDAETMRLHHEAHHRSYVERLNAALADVPEFHGLPFSRLLIDLTALPTALGTAVRNHGGGHLNHTLWWGWLEPGGPAAPPSALRRALEGAFGSTESFRERLLGAAAAHFGSGWAWLVLDAA-GRLRVRTTRNQDSPVMDGELPVLGVDVWEHAYYLTYRNRRGEYLENLWRLVNWDAVARAYERAEA

>D7JFA1_575590_Bacteroidetes_B_Mar

MKHELIVLPYPANALEPVISETTINLHHGKHLATYVNNLNNLIQGT-KFENADLETIVRES-------DGALFNNAGQTLNHNYYFTQFAPKSEGRPSGRLLKAIEEAWGSFENFRSEFETAATSLFGSGWAWLAADKE-GKLQITKEQNAGNPVAKGLKPILGIDVWEHAYYLDYQNRRADHLKELWKIISWAEVEKRY-----

>D8J2G3_795797_Halobacteria_A_Arc

SNAELPPLPYDYDALEPSISEQVVTWHHDTHHQGYVNGLNSAEETLAENRESGDY---SSTG----GALGNVTHNGSGHYLHTLFWENMSPEGGDEPSGDLRERIEEDFGSYEGWKGEFEAAAK--AAGGWALLVYDPVAKQLRNLAVDKHDQGALWGAHPVLALDVWEHSYYYDYGPDRGDFIDAFFDVVNWDEAEDQYQKSVD

>D8JZF0_552811_Chloroflexi_B_Ter

-------MGYEASGLTG-FSDKLLSNHF-TLYQGYVKNVNKLLETLQSLAGEAKG-GTPEYA----ELKRRFGFEWNGLRLHELYFGNLGGNGTPIPGGRLVKELTEQWGSFDAWETEFRATGS-LRGVGWAVLYQDTENGRLFNVWVDQHESGHLAGCRSILVMDVWEHAFMLDYGLKRPDYIAAFFKNINWSVCEKRLA----

>D9Q0R7_666510_Thermoprotei_A_Arc

KRYELPPLPYNYDALEPIISAETLRYHHDKHHLGYVNGANAALDKLEKYLNGQLTD--IDVR----AVSRDFEFNYGGHILHTLYWLNMAPKGGGTPGGAIGDAINKFFGSFDKFKKLFGDAAKNVEGVGWAILAYDPVTGDLRILQVEKHNNVVTTNLIPLLAVDVFEHAYYIDYRNDRAKYVDSWWDLINWDDVEARYQKALN

>E0TI36_314260_Proteobacteria_B_Mar

MAISLMDLPYEKTALAPHISEDTLNYHHGKHHQAYVTKTNDAIKGT-ALDDADLEAIVKEAKK---TANQGLFNNSAQVWNHNIYWQSMSPNGGGAPKAKIAEAIDKSFGSYDEFKAKFKDAGGTQFGSGWAWLVAKKD-GSLEILKTLNADCPLTDSVTTLLTMDVWEHAYYLDYQNARPDYMTHFLNLVNWDFAEERLAAA--

>E1QRN4_572478_Thermoprotei_A_Arc

KRYELPPLPYSINALEPHISGQVIDVHYNGHHKGYVNGANATIERLEKIIKGDVTS--YDIQ----GLLRSLFFNVNGHKLHTLYWYSMAPPGGGTPGGYLGDLIKKQFGSFDRFKALFTEVMRSLPGCGWTVLYYDPETGNLEFTTFENHYNQHIAELPILLIVDEFEHAYYLQYRSNRNGYIDAIWNVLNWEEAENRLRKYIK

>E1QXA5_633147_Actinobacteria_B_Ter

--FEQIKLPYAYDALEPYVDALTMETHYSKHHTTYTNALNAAAEK-AGVADQPIERLLSSLDGIDEPLRGTIRNNGGGFYNHNLYFSIMSPAPAAEPAGVLRERIEEAFGSVGELKAELKKAAVGRFGSGWAWLNAAPD-GTLSVTSSPNQDNPFSEGVTPILGIDVWEHAYYLKYKNLRGDYVDAFFSVLDWDAVAANYAKATA

>E1SRJ0_550540_Proteobacteria_B_Mar

MAFELPALPYAKDALEPHISAETLEFHHGKHHATYVTKLNGLVEGT-ELAGKSLEEIVKTS-------TGGVFNNAAQIWNHTFYWHCLSPNGGGAATGAIADAINSAFGSFDAFKAQFTESAINNFGSSWTWLVKNAD-GSLAIVNTSNAATPLTEGVTPLLTVDLWEHAYYIDYRNARPNYMDAFWNLVNWDFVNENFA----

>E1VHB8_83406_Proteobacteria_B_Mar

MSFELPALPYAKNALAPHISEETLEFHYGKHHNAYVTNLNNLVKGT-EFEGKSLEEVVRKS-------SGGIFNNAAQVWNHTFYWNSLSPKGGGAATGAVAEGINKAFGSFDKFKEEFSKSAAGNFGSGWTWLVKKAD-GSLAIVNTSNAATPITEGVTPLITVDVWEHAYYIDYRNLRPKYLEAFWALVNWDFANANLAK---

>E1X0Y3_862908_Proteobacteria_B_Mar

MAHELPKLPWADNALEPHISAETISFHYGKHHNAYLTKLNAAIPGT-EYESMTLEETIMKS-------EGGLFNNAAQVWNHSFYWNCLAPNAGGAATGAVAEKINAKWGSFEKFQEDFTNAAATNFGSGWTWLVEE-G-GELEIFNTANADTPMKHGKKALMTIDVWEHAYYVDYRNARPNYIEAFWKLVNWDFVNSNL-----

>E4NT27_469382_Halobacteria_A_Arc

MSYELDPLPYEYDALEPHISEQVLTWHHDTHHQGYVNGWNSAEETLESNREEGDF---GSSP----GAIRNVTHNGCGHILHDLFWNSMSPEGGSEPTGSLADRIEEDFGSYEAWKGEFEAAAG--NAGGWALLVYDSFSNQLRNVVVDKHDQGALWGSHPILALDVWEHSYYHDYGPARGDFIDAFFEVVDWEEPSARYEQAVE

>E4T0E9_694427_Bacteroidetes_B_Mar

MKFSLPELPYAHNALEPVISEKTISFHYGKHHQTYVNNLNGLVEGT-EFENSDLESIVKKS-------EGPIFNNAAQIWNHTFYFLTLTPNKGSVPSEKLAKAIDAAWGSLDNFKAEFNKAAVSVFGSGWAWLVKDAE-GKLSIVKESNAGNPITRGLTPLLTFDVWEHAYYLDYQNRRPDYVAALWDLVDWKTVSERY-----

>E4T355_694427_Bacteroidetes_B_Mar

GQYKLPNLPYKYDALEPYIDSVTMHIHHDLHHATYVSNLNKALEKNPELYKKSLVELIQTLNTLPADIQTAVRNNGGGVYNHSFFWEIMAPAGTATMSPKLEKILSENFGSVDAFKAEFEKAAAGRFGSGWAWLIKDPA-GKLRIISTPNQDNSLMPKGKPVLALDVWEHAYYLKYQNKRAAYAKAFWSVVNWTKVEQLIEQ---

>E6SLN6_644966_Firmicutes_B_Ter

MAYQLPPLPYDYNALEPHIDEQTMRIHHDRHHATYVNNVNAALEKYPALQNKPIEELLRQIDQVPEDIRTAVRNNGGGHANHSLFWEIMSPRGGGQPSGALAEAINRSFGSFDAFKDQFTKTATTHFGSGWAWLVVDEK-GELQVYSLPNQDSPYMKGHTPILGLDVWEHAYYLKYQNKRPDYIAAWWNVVNWDEVARRYQQARG

>E8QXM5_575540_Planctomycetes_B_Mer

MAYTLPALPYAYDALEPHIDARTMEIHHTKHHQAYITNLNNAIKDQPALQDLPIEKLIADLGAIPEAIRTTVRNNGGGHANHALFWQIMSPNGGGQPVGKLAAAIEGELHGFDSFKDAFSKAGLNRFGSGWAWLALDPS-KKLVITSTPNQDSPIMEGMIPLLGMDVWEHAYYLKYQNRRADYIAAFFNVINWPMVDELYVKAMG

>F0TCH4_877455_Methanobacteria_A_Arc

KNYQLPELPYGYKDLEPYISEEQLKIHHTKHHKAYVDGANAILKKFDN-R-GSEE---FDVK----AVSKELTFHVGGYVLHKFFWENMGPACGGEPTGTIAEYIKKDFGTFERFKKEFSQAATGVEGSGWAVLTLCRMTNRIFIMQVEKHNVNIIPGFRIMLALDVWEHAYYLDYQNRRPDYVEAFWNLVNWEEVNRKMDVWLS

>F2IV67_991905_Proteobacteria_B_Mar

MSFQLPDLPYAYDALGPYMSAETLEYHHDKHHLAYVSNGNNLLKDS-GLEGKSVEEVVKESFG----KNPGLFNNAGQHYNHIHFWKWMKPNGGGKLPGALASKIDSDLGGYDKFRADFINAGITQFGSGWAWLALK-D-GKLAIMKTPNGENPLVHGAAPLLGVDVWEHSYYIDYRNLRPKYLEAFVNLVNWDYVLEMYEAAA-

>F2KQT8_693661_Archaeoglobi_A_Arc

SRYVLPELPYEYNALEPYISEEILKLHHDKHHSAYVKGANAALEKLEKARKGEIE---VDIK----AVLKELSFHVGGHILHTIFWNCMTPE-KGEPSGVLAEKIKEEFGSVERFKDEFSKAANSVEGSGWAALMYCPLTGRLIIQQIEKHNVNLAPGLHILACIDVWEHAYYLQYKNDRASFVKNWWNVVNWNFIEERLKEAMK

>F4B3P9_933801_Thermoprotei_A_Arc

KKYELPPLPYNLDALEPYISKEIIDVHYNGHHRGYVNGANSFVDRVNKILKGEISSGQYDIQ----GLLRGLVFNINGHKLHSLYWQNMAPAGGGKPGGVIGDLIEKQYGSFEKFKALFTEAANSLPGTGWAVLYYEVENGNLQIMTFENHFQNHIAELPILLILDEFEHAYYLQYKNKRADYVNNWWNLVNWDFADKKLQQYMK

>F4BZF7_990316_Methanomicrobia_A_Arc

KKYTLPELPYAGNALEPHITQAQLVLHHDKHHAAYVNGANAILERLDKARESGSD---IDMK----ATLKELSFQAGGHVLHSLFWANLAPAAKKEPEGALAEALKKEFGSIERFKKEFTTAAVSTEGSGWAALSWCGMTGRPIIMQIEKHNVNVYPMFRILMVLDVWEHAYYLDYKNERAKFVEAFWNIVNWDEVNRRLEAVIK

>F7PIA8_1033806_Halobacteria_A_Arc

SNPELPSLPYDYDALEPHISEQVLEWHHDTHHQGYVNGLDAAEETLAENRASGDH---STTG----GALGNVTHNGSGHYLHTLFWENMSPNGGGEPEGALLERIEEDFGSYDAWKAEFEAAAG--AAGGWALLVYDPVAKQLRNVAVDKHDNGALWGSHPILALDVWEHSYYYDYGPDRGSFIDAFFEVLNWEKVAEEYQKSLD

>F7XLC9_679901_Methanomicrobia_A_Arc

EYYKLPPLAYGYDELEPYISEEQLRLHHDKHHQSYVDNLNSIIKMVEKAREEGKD---YDYK----AATKAASFNAGGNVLHDYFWWEMMPAAGEEPVGELLEQIKKDFGSFERFKKEFSQVALTVEGSGWAALTYCGDTHRLSPIQIEKHNVNVYPDYPIIMVLDMWEHAYYIDYRNEKAKFVDGFWNIINWEELDKHFKKYL-

>F8D6A5_797210_Halobacteria_A_Arc

ADHELPPLPYDYDALEPSISEQVLTWHHDTHHQGYVNGLNSAEETLAENREEGDY---GSTP----GALKDVTHNGCGHYLHTLFWENMSPDGGGEPEGEVADRIEEDFGSYEGWKGEFQKAAG--AAGGWALLVYDPVAKQLRNLAVDKHDQGALWGAHPILALDVWEHSYYYDYGPDRGEFIDAFFDVVNWDSVEEEYQKCVD

>F8DDU6_797210_Halobacteria_A_Arc

SDYELPPLPYDYDALEPHISEQVLTWHHDTHHQGYVNGWNSAEETLAENREEGDF---AGSA----GAIRNVTHNGSGHILHDLFWQSMSPEGGDEPEGDLADRIEEDFGSYEAWKGEFEAAAS--NAGGWALLVYDSFSNQLRNVVVDKHDQGALWGSHPILALDVWEHSYYHDYGPARGDFVDAFFEVVDWEEPSARYEQAVE

>F8KWM1_765952_Chlamydiae_B_Mar

LQYKLPDLPYDLGDLEPVISKEIMSLHYNKHHATYVANLNKALEQYAEAEAKNDL---PALI----TLQSAINFNGGGHINHSIFWTNLAPQGGAPPEGAIAEAIQKQFGSLEKFIETMNTKTAAIQGSGWGWLGYCKNKKQLEIVTCSNQDPLAAKGLVPLLGIDVWEHAYYLQYKNVRADYLKAIWNIVNWKNVEERFTKAKA

>F8L0F2_765952_Chlamydiae_B_Mar

MA--M-TKKYEVDHLLGKLNDDLLKMHF-KLYQGYVNNTNTLLQKIGELDQTGKS-QSPEFA----GFKHMLGWEFDGMLLHEYYFENLGGQTHLKQDDPLFLKMVQDFGGYDQWKSDFQATGA-IRGIGWVITYVDPKQGRLVNTWINEHDVGHLSGGKPLLVMDVFEHAYITQFGLDRAKYIQVFFDNIDWNAVSQRYKKTL-

>F8L7W5_331113_Chlamydiae_B_Mar

--FSLPPINIEASHLLGHIDDDLLKMHF-TLYNGYVKNASALLTTLVQMRQNGKD-TTLEYG----AIERRFVWEFDGMILHELYFENLGPKPFLDRKDPLLLKMTMDFGSFEQWKKNFVATGL-IKGVGWVILYQSPKTGHLNNIWVDEHNINLVPGGKPLLIMDVWEHAYITEYGLDRAGYIEAFMQNIDWEVVSKRFNDMES

>F8L8N3_331113_Chlamydiae_B_Mar

MAFKQPDLPYDLSALAPFVSEEQMHYHYNKHHAAYFNKLNGLVEGK-KEAQLSLEEVVVQS-------SGGVFNNAAQAWNHTFFWHCMSPNGGGQPKGELKEAIERDFGGLEPFMKQFSDAAATLFGSGWAWLASDGQ-GKLEIMALSNADTPLKHNKTPILTLDVWEHAYYIDYRNERPRFIEQFKDVIHWDFAQKCYLDAK-

>G2E543_765913_Proteobacteria_B_Mar

MAHELPALPYEKNALEPVISAETIEYHYGKHHQTYVTNLNNLIAGT-EFETMSLTDIIMKS-------SGGLFNNAAQVWNHSFYWKCLSPNGGGAPSGALASAIDAKFGSFDEFKKQFSQSAATNFGSGWTWLVKNAD-GSIEIFNTANAGTPMTEGKTALLTIDVWEHAYYIDYRNARPKYLESIWNKIDWSFVEANFS----

>G2E6T6_765913_Proteobacteria_B_Mar

MPHRLPDLPYPTDALEPRMSSETLSLHHGKHHATYVDKLNALIEGT-AFGDAVLSDIVAKA-------HGGIFNNAAQAWNHAFFWNCLRPDGGGNPQGDLAAAIDRDFGSVEALRKEFSTQLTTLFGSGWVWLARDDD-GNLSVESHSNAGNPITEGKLPILTCDMWEHAYYVDYRNQKKDYVDAFWHLVNWDFAAANFDRNTP

>G2FFW9_1049564_Proteobacteria_B_Mar

MAHELPALPFAIDALEPVISQETLEFHHGKHHNTYVTNLNNLIPGT-EFENASLEEIIMKS-------EGGVFNNAAQVWNHTFYWNCLSPGDENAPTGALADAINSTFGSFDEFKQKFATSGATNFGSGWTWLVQNED-GSLEIYNTSNAGTPMTAGKKALLTADVWEHAYYIDYRNARPAYLDAFWKLVNWDFVASNMG----

>G2KRC6_856793_Proteobacteria_B_Mar

MALILPELPYAYDSLQPFMSSETLEFHHDKHHKAYVDKGNELLAGS-GLENLSLEEAMVAAFK--DKSKAGLFNQLGQHYNHIHFWNWMKKNGGGSIPGKLDAQITSDLGGFDTFRGNFIQAGMTQFGSGWCWLAVDAT-GKLEITKTANGENPLVYGKTPILGCDVWEHSYYIDYRNARQKYLEAFVSLINWDYVGQLFEKGPL

>G6A1M0_909943_Proteobacteria_B_Mar

MAFELPELPYAYDALAAGMSQETMEFHHDLHHKAYVDNGNKLIAGT-EWENSSLEQIITGTYQSGAVAQNGIFNNASQHWNHIQFWEMMGPSGR-QMPSELDAALNAQFGSIDAFKEQFVAAGVGQFGSGWCWLVQNAD-GSLAITKTENGVNPLCFGQTALLGCDVWEHSYYIDFRNKRPAYLTNFLNLVNWENVAARMAG---

>G6FT84_372787_Cyanobacteria_B_Ter

GAIQLPPLPYDYNALEPHIDEATMRFHHDKHHATYVKNLNAALDKYPVLKNKNVEDLLRNLNSVPEDIRTTVRNNGGGHINHSMFWLIMKPDGGGEPTGAIANAIQDNFGSFANFKKQFNDAGSKLFGSGWVWLVRNPD-GKLEVVTTPNQDTPLSEGKYPIIGNDVWEHAYYLKYQNRRADYLDAWWNVLNWDEINKRFAAAT-

>G6G070_372787_Cyanobacteria_B_Ter

MAFTQPPLPFAFDALEPYMKAETFEYHYGKHHKAYVDNLNKLTEGT-ELADKSLEEVIQLSFK--DSSKTGVFNNAAQVWNHTFFWNCLKPAGGGAPTGDLATKIEKDFGSFDKFKEEFSNAAATQFGSGWAWLIDD-G-GTLKVMKTPNAENPLAHGKKALLTLDVWEHAYYIDFRNARPAFIKNFLQLVNWDFVAEQYATA--

>G7LTJ2_598467_Proteobacteria_B_Mar

MSYSLPSLPYAYDALEPHFDKQTMEIHHTKHHQTYVNNANAALESLPEFAELSAEELLTKLDKVPAEKKTVLRNNVGGHANHSLFWKGLKL--GTTLAGDLKAAIERDFGSIDTFKEKFEQAAATRFGSGWAWLVLKDD-GKLAVVSTANQDSPLSGSGYPILGLDVWEHAYYLKYQNRRPDYIKAFWNVVNWDEAAKRFAEAKK

>G7VBY8_1104324_Thermoprotei_A_Arc

KKYTLPELPYAYNALEPYIAEEIMKLHHQKHHQGYVNGANAALEKLEKFRKGEAQ---IDIR----AVLRDFSFHLNGHVLHSIFWPNMAPPGGGKPGGKTADLINQFFGSFEKFKEEFSQAAKNVEGVGWAILVYEPLAEQLVILQVEKHNLMHAADAQVLLALDVWEHAYYLQYKNDRGSYVDNWWNVVNWDDVERRLQKALN

>G7WL45_1110509_Methanomicrobia_A_Arc

KFYSLPELPYAYNALEPQISEAQLRLHHDKHHAAYVNGANAILERLDKAREAGAD---LDMK----ATLKELSFQAGGHLLHSLFWRNLAPAAAEEPKGALADALKEEFGSFARFKKEFSAAAATAEGSGWAALAWCGMTNRPVIMQIEKHNVNVYPMFRILMVLDVWEHAYYLDYKNERPKFVEAFWKIVDWDEVSRRLEAAIK

>G8NWQ2_682795_Acidobacteria_B_Mar

MAFELPPLPYDYAALEPTIDEATMKLHHDKHHQTYVTNLNGAVEKHPDLGKKTPEELIKDLDSIPEDVRGVVRNNGGGHVNHTMFWQIMGPNGGGEPTGEIAAQIKADFGSFEDFKKKFNETTAKQFGSGWGWLVFK-G-GKLEIVTTANQDNPLSQGLYPILGNDVWEHAYYLKYQNKRPDYLAAWWNVINWAEVNKRFEHAKK

>G8R729_926562_Bacteroidetes_B_Mar

MSFELPDLPYSKSALEPYIDAQTMEIHHDKHHAGYTTKLNDAIEG-TELEKQSIEDILKNVSKHS----GGVRNNGGGYYNHSLFWSIMGPDAGGDPTGDVGAAIDDAFGSYENFKTEFSNAAATRFGSGWAWLIVNGE-GKLEVTSSPNQDNPLMDKGTPILGLDVWEHAYYLKYQNKRPDYISAFFNVINWDEVNRRFAEAK-

>H0PZQ1_748247_Proteobacteria_B_Mar

MEHTLPPLPYSKDALAPHISAETMEFHYGKHHQAYVTNLNNLIKGT-EYENLDLEAIVKKAP------AGGVYNNSAQVWNHTFFWNSMKPNGGGEPTGALGDAIKAKWGSFEDFKKAFTASAVGNFGSGWTWLVKKAD-GAVDIVNMGAAGTPLTTGDKALLCIDVWEHAYYIDYRNRRPDFVATFLSLANWDFAAKNFAG---

>H0TES2_551947_Proteobacteria_B_Mar

MTFTLPPLPYDYEALAPYMSKETLEYHHDKHHQAYVTNGNNAIKGT-EFEGKSLEEIVKGSYG----KNAAVFNNAGQHYNHIHFWQWMKKDGGDKVPGRLEKKINEDLGGFEKFKADFAAAGVGQFGSGWCWLQVK-N-GKLEISKTPNGENPLVHGATPILGCDVWEHSYYIDYRNRRPDYLKAFVNLINWDYVDELFDKTV-

>H2C9I7_671065_Thermoprotei_A_Arc

KKFELPPLPYKVDSLEPYISKDIIDVHYNGHHKGYVNGANQFMERFNKVIKGELQAGQYDVQ----GLMRGLVFNINGHKLHALYWDNMAPAGGGKPGGALADLIEKQYGGVERFKQVFTETANSLPGTGWTVLYYDTENGNLEIMTFENHFMNHIAELPILLILDEFEHAYYLQYKNKRADYVTNWWNVVNWDYAEKKLQKYLR

>H6L6A2_984262_Bacteroidetes_B_Mar

SEFVLPALPYAADALLPHIDTETMNIHHGKHHAGYVKKLNAALADNPQKEANNLEDLLGRLED--KEEHTALRNNGGGHYNHSLFWTNMSPEGGGTPEGEFGQALAQAFGSFENFAKEFKAAAASRFGSGWAWLAVNAK-KELYICSTPNQDNPLMKAGQPILGLDVWEHAYYLNYQNQRKAYINSFFNIINWSAVSQNWSNIVK

>H6L8Y0_984262_Bacteroidetes_B_Mar

MAFELPKLPYAHDALEPHIDARTMEIHHGKHHAGYTNKLNAAIVG-TDLEGKSIEDILANVSAAG----AGVRNNGGGFYNHSLFWSVMSPNGGGEPTGEVAEAIKAAFGDYETFKDKFAAAAKTRFGSGWAWLCVK-D-GKLEVCSTPNQDNPLMDKGTPILGLDVWEHAYYLNYQNRRPDYVEAFFNVINWEEVNKRYAAAK-

>I0AK38_945713_Ignavibacteriae_B_Mar

GKFELPPLPYSYDALEPYIDKMTMEIHHTKHHQAYINNLNKAIEG-TDMEKMTMEEMFASVSKLP----VAVRNNGGGHWNHSLFWTLMKPNGGGKPSGALADAISAQFGSFDDFKKKFSDAAAGRFGSGWAWLVKA-D-NKLIVTSTPNQDNPLMDKGSPILGLDVWEHAYYLKYQNRRPEYIENWWNVVNWDKVAELFSKAK-

>I0I4H8_926550_Chloroflexi_B_Ter

MAFTLPDLPYPEDALEPYIDARTMNIHRTKHHQAYVTNLNNAIAG-TEWEKLSIEEILRRIDEVPENIRTTVRNNGGGHANHSLFWKIMAPNAGGAPTGALAEAINAKWGSFDEFKAQFKAIAVGRFGSGWAWLVVKPD-GSLDAYSLPNQDSPYMQGDTPILGLDVWEHAYYLHYQNRRPDYVDAWWNVVNWDEVAKNYAAAKG

>I0IDH9_1142394_Planctomycetes_B_Mar

MAFSLPSLTYAYDALEPAIDARTMEVHYSKHFAGYASKLTAALEG-TEWADRSVEEILAKLDQLPEGKQTAVRNNGGGYFNHDLFFTTLAPSENNAPSGELAEAIERDFGGFADFKKSFSDAAASRFGSGWAWLCVQPG-GKLHVTSTANQDTTFMPGHHPVLGLDVWEHAYYLHYQNRRPDYIEAFFSVIDWDKVGEKHAAAKG

>I0IFX1_1142394_Planctomycetes_B_Mar

VGHALPDLPYAADALMPHIDAETMRIHHGKHHAGYVRKLNDALAAYPDLAERDPAELVAMAGELPADLQRPVVNNGGGHVNHTMFWRMMSPDGGGEPRGELAGAIDAAFGTFAGFQAAFSGAASGRFGSGWAWLVVGRD-GELAVTSTANQDSPLMGGQTPVLGLDVWEHAYYLKYQNRRADYVEAWWNVVNWDEASERYARATD

>I2JH08_1168065_Proteobacteria_B_Mar

MAFELPALPYAKDALQPHISAETLDFHHGKHHNAYVTKLNELLPGS-EFEGKTLEEIIKAS-------SGVMFNQAAQVWNHTFYWNSLSPNGGGAPTGDLAAAIDKAFGSFDAFKTAFNAKAVGNFGSGWTWLVKNSD-GSLEIVNTDDADTPIAEGQTPLITADVWEHAYYIDYRNARPKYLEEFWNLVNWEFAAANFA----

>I2K993_1165841_Proteobacteria_B_Mar

MTHTLMDLPFDENALEPYISKETLQYHHGKHHAGYINKLNILIEGT-VYAEMKLEEIVTKA-------DGGIFNNAAQVFNHNFYFNGMSKK-ATSPSKELLALFERDFGSIESFKEKFLDMAAGLFGSGWVWLSIKDS-GTLSIESFSNAGNPLLLGHTPLLTCDVWEHAYYIDYRNARADYLEKWWELVNWDFVSKNLEASTQ

>I3R8L9_523841_Halobacteria_A_Arc

MSYELDPLPYEYDALEPHISEQVLTWHHDTHHQGYVNGWNAAEETLEANREAGEF---DSSA----GAIRNVTHNGCGHILHDLFWQNMSPEGGDEPAGALADRIAEDFGSYDAWKGEFEAAAG--AAGGWALLVYDSFSNQLRNVVVDKHDQGALWGSHPILALDVWEHSYYHDYGPARGDFISAFFEVVDWDVPAARYEQALE

>I3RA82_523841_Halobacteria_A_Arc

SEAELPPLPYDYDALEPHIDEQILAWHHDTHHQGYVNGLNAAEETLRENRSSGDM---DGSA----AAMRSVSHNGCGHYLHHLFWRCMTPNGGGEPTGDLRDRIESDFGSYEGWEAEFEAASW--PAGGWALLVYDPVTKQLRNLAVQKHNDGALWSAHPILALDVWEHSYYFQYGPDRGGFVDAFFEVIDWDTIASEFEKTTS

>I3RBB8_523841_Halobacteria_A_Arc

SDYELDPLPYEYDALEPHISEQVLTWHHDTHHQGYVNGWNAAEETLEANREAGEF---DSSA----GAIRNVTHNGCGHILHDLFWQNMSPEGGDEPTGALADRIAEDFGSYEAWKGEFEAAAG--AAGGWALLVYDSFSNQLRNVVVDKHDQGALWGSHPILALDVWEHSYYHDYGPARGDFISAFFEVVDWDVPAARYEQALE

>I4AH06_880071_Bacteroidetes_B_Mar

SPFQLPTLPYDLAALAPNIDKQTMEIHHGKHHQGYVNKLNKAIEG-TDFANQNLLEILENVTE----NDTAVRNNGGGHYNHSLFWESLSPK-NQVATGNIKAALEKDFGSYETFQKEFSAEAKGVFGSGWAWLCKAND-GKLFITSTPNQDNPLMSTGTPILCLDVWEHAYYLNYQNKRAEYIENFFKIINWEKIEERFNA---

>I4APS4_880071_Bacteroidetes_B_Mar

MAFELPKLPYAYNALEPNIDEQTMTIHHTKHHQGYTNNLNAAIEG-TDLEGKSIEEILKSVGSAS----KAVRNNGGGYYNHDLFWKVMSPNGGGEPSGELAEAINKSFGSFAEFKTQFETAAKTQFGSGWAWLIVKAD-GSLAVTGTPNQDNPLMDKGTPIFGIDVWEHAYYLKYQNKRPDYVGAFWNVANWEQVTENFKKAK-

>I4EEM3_1129897_Chloroflexi_B_Ter

MAFTLPPLPYAYDALEPYIDEQTMHLHHDRHHAGYVNNLNAALESAPEFFNQSIEDILRNINDVPEAIRTAVRNNGGGAENHTMFWEIMGPNGGGEPTGDLANAINSAFGDFATFKDKFSKAGATRFGSGWAWLVAR-D-GTLDVISTANQDSPFMDGLYPVMGIDVWEHAYYLKYQNRRPEYISAWWNVVNWPAIADRYAKAIG

>I6ZUA8_1191523_Ignavibacteriae_B_Mar

SKFELPALPYAFDALEPYIDARTMEIHHDKHHAGYVNNLNKAVEG-TEYEGKSLEELFKTVSKLP----VAIRNNGGGHYNHSMFWQIMGPNKGGEPTGALADAINGTFDSFEKFKETFNNAAATRFGSGWAWLVLS-N-GKLVVTSTPNQDNPLMDQGFPVMGLDVWEHAYYLKYQNRRPEYITNWWNVVNWDEVAKRFDSVK-

>I7KYD2_1201294_Methanomicrobia_A_Arc

KKYELPPLPYAPDALEPHISKEQLSLHHDKHHQAYVTGANANLERLEKARQEGTD---VDMK----ALLKELSFNIGGHILHTLFWPGMAPAGGGTPGGALADLIDREWGSFDRFKAEFSKAAASVEGSGWAALAYCTMTDRPMIMQIEKHNNNVYPTFQILMVLDVWEHAYYVDYKNNRGQFVDAFWNVANWDEVNRRLEKI--

>K0AYP0_1128398_Firmicutes_B_Ter

NTINIKQFNFNN------ISRNQLDQHY-ELYKGYVRNINKIWDILDQTPENDSNPTYSPLR----CLRLGETYALDGVKLHELYFENLNSN-YNRPFGPIVDLIIRDFYSFERWENLFKQTGL--AMRGWVVLAIDHIDKRLHIYGQDEHDKGSIWMAHPLLVLDVYEHAYMIDFGIDRKKYIDIFMENIDWNIVNQRLEMFML

>K1JSW8_742823_Proteobacteria_B_Mar

-MFTLPALPYALDALEPAMSRETLEFHWGKHHQTYVNNLNGLIAGT-DFEGKSLEEIIRTS-------TGGVFNNAAQVFNHTFFWQGLKPQGGGMPEGALFEAINAIWGSYDAFRKAFTASAAGNFGSGWTWLVKNAD-GTLAIVNTSNAGTPLTGDQKPLLALDVWEHAYYVDYRNARPKFIDAFFSLVNWEFAQQNFAA---

>K2JEV8_745411_Proteobacteria_B_Mar

MAFELPALPYEKNALEPHISAETLEYHYGKHHNTYVVNLNNLTAGT-ENEGKSLEDIIKSS-------TGGLFNNAAQVWNHTFYWHCLSPNGGGAATGAVADAINAAFGSFDAFKEEFTKNAIGNFGSGWTWLVKKAD-GSVGIHNTSNAGCPITEGVTPLLTVDVWEHAYYIDYRNARPKYMEAFWALVNWDFVNKNLA----

>K4MC62_1094980_Methanomicrobia_A_Arc

ELYKLPALKYGYADLEPYISEEQLRIHHDKHHQSYVNNANSLLQMMDKAREEGTD---FDYK----ATAKAFAFNLGGHVLHDYFWWEMTPAASKEPTGELSDAVKENFGGFERFKKEFSQVASGVEGSGWAALTFCTDTNRLGIMQIEKHNVNLIPDYPIIMALDVWEHAYYLDYKNERGKFIDAFWNIVDWEELDKWFKKVQN

>K9PE47_99598_Cyanobacteria_B_Ter

MAFVQAPLPYDFNALEPYMKAETFEYHYGKHHKAYVDNLNKLTDGT-ELADKSLEEVIQISYK--DSSKVGIFNNAAQVWNHTFFWSSLAPAGGGTPTDELAAKIDKDFGSLDKLKEEFSNAAATQFGSGWAWLIDD-G-GTLKVIKTPNAENPLAYGQKALLTLDVWEHAYYIDYRNARPGYIKNFLQLINWDFAAENLAKA--

>K9PU33_99598_Cyanobacteria_B_Ter

GSVKLPPLPYAYEALEPHIDAKTMQFHHDKHHAAYVKNLNAALEKHPELKNKSVEELLRKLDTVPEDIRKVVRNNGGGHVNHSMFWQIMKPKGGGDPTGEIATAINQNFGSFAAFKKQFNEAGASRFGSGWAWLVRTKD-GKLEVTSTANQDSPLSAGKYPILGNDVWEHAYYLNYQNRRADYLEAWWNVVNWEEINKRFAAASK

>K9SBX7_1173025_Cyanobacteria_B_Ter

MSYEFPNLPYAQDALEPHISANTLSFHHGKHHAKYVSTYNEMVQGT-ENESKSIEEVIKASYD--PSAKSGLFNNAAQSWNHTFYWYCMKPGGGGEPTGELAEKIKADFGSFEKFKEEFKAAGGSQFGSGWAWLVLD-N-GTLKVTKTPDAVNPIAQGQTPLLTMDVWEHAYYLDYQNLRPSYANTFLSLVNWDFVAEQYAKAK-

>K9T440_118163_Cyanobacteria_B_Ter

MAYELPALPYDYTALEPVISKKTLEFHHDKHHAAYVNNYNSAVKGT-EFENKSIEEVIKAVAG--DSSKTAIFNNGAQAWNHTFYWNCMKPNGGGTPSGALADKIKADFGSFDKFVEEFKTAGATQFGSGWAWLVLD-N-GTLKVTKTPNADNPIVAGQVPLLTMDVWEHAYYLDYQNRRPDYINEFIKLINWDFVAQNFAAAS-

>K9T7F8_118163_Cyanobacteria_B_Ter

KPFKAIPLPYAYDALEPYIDAETMRFHHDKHYVTYTKNFNAAISKYPQLANQSAEEIISDLDRLPQDIRTTVRNNGGGYVNHTMFWEIMSPKGGDRPTGELARAIDKTFGSFDKFKTAFNDTGSKQFGSGWAWLVLDRN-KQLKIMGTPNQDSPLMMGMYPVMGNDVWEHAYYLKYRNERGKYLDAWWNVVNWDEVNKRYQQAMA

>K9ULY3_1173020_Cyanobacteria_B_Ter

DVFALPPLTYDYKALEPHIDAATMKFHHDKHHAAYVKNLNAAVNKYPELKTKSVEQLLTSLSALPKDIQTTVRNNGGGHYNHTMFWRIMGPKGGGMPTGSIATAINSQFGTFDTFKTQFNQAGTKLFGSGWVWLVSD-K-NKLKIITTPNQDSPISQGLYPIMGNDVWEHAYYLNYQNRRPEYLSAWWNVINWTEVNKRFAQAQK

>K9UQB8_1173020_Cyanobacteria_B_Ter

MAFVQEALPYDVSALEPHMSAKTFEFHYGKHHATYVTNLNNLTKDT-PMADMSIEDVVRGSFG--DASKAGIFNNAAQVWNHAFFWKSMKPNGGGAPTGELAAKIDADFGSFDQFKTDFKAAATTQFGSGWAWLVND-G-GTLKITKTGNAENPLVHGQTPLLTLDVWEHAYYLDFQNRRPDFISTYLSLVNWDFAAANFK----

>K9VXC7_1173022_Cyanobacteria_B_Ter

MAFEQPPLPYDFNALEPHMSAKTFEFHYGKHHAAYVTNLNKLVQDT-EMADKSLEEVIKASFG--DSSKTGIFNNAAQVWNHTFFWNCMKPNGGGQPTGELADKINTAFGSFDKFKEEFKNAAVTQFGSGWAWLVKD-G-DTLKITKTPNAENPLAHGQTALLTVDVWEHAYYLDYQNRRPDFVQTVLNLINWDFVTQNLSA---

>K9XDJ4_1173026_Cyanobacteria_B_Ter

MAFELSALPYNYDALEPYIDAQTMQLHHDMHHQAYVNNLNAAVEKHAQLQSKSLEDLVRELDSIPDDVRTAVRNNGGGHVNHTMFWEIMGPNSGGEPTGAVGEAIQDTFGDFETFKQRFNDAGTKQFGSGWVWLVRSPD-GKLEVMSTPNQDSPITQGYFPIMGNDVWEHAYYLKYQNRRAEYLKQWWNVVNWDEINKRFEMSTR

>K9XKI9_1173026_Cyanobacteria_B_Ter

GSFELPPLPYAYNALEPHIDAATMRFHHDRHHATYVKNLNAALEKHPQLKGRSAEQLLSNLNSVPEDIRTSVRNNGGGHVNHSMFWRIMSPDGGGEPTGQIATVINQNFGSFAEFKKQFNSAGEGRFGSGWAWLVRTRD-GNYQITSTANQDSPFMEGNYPIMGNDVWEHAYYLKYQNRRAEYLNAWWNVVNWNEINQRLAQATK

>K9XY88_111780_Cyanobacteria_B_Ter

MAYELPSLPYDYTALEPYISKSTLEFHHDKHHAAYVNKYNDAVKGT-ELDSKSIEEVIKAIAG--DSSKTGLFNNAAQAWNHTFYWQCMKPNGGGTPTRELAKKIEADFGGFDQFVEAFKDAGATQFGSGWAWLVLD-G-DKLKVTKTLNADNPLTSGQVPLLTMDVWEHAYYLDYQNKRPSYIDEFVHLINWDFVAQNLSAA--

>K9XZ62_111780_Cyanobacteria_B_Ter

KPFQLPPLPYAYDALEPYIDAETMRFHHDKHHAAYTKKLNQAVNQYPELANQSAEDILRNMDTVPEDILTTVRNNGGGYVNHAMFWQIMSPDGGGNPTGAIATAITETFGSFDAFKEQFNEAGSKQFGSGWAWLVLDTN-NQLQVMSTPNQDSPLMDGMYPIMGNDVWEHAYYLKYRNKRDEYLTQWWNVVNWEEVNKRYEQAIA

>K9Z4N7_755178_Cyanobacteria_B_Ter

MAYQLPNLPYEYNALEPYISKSTLEFHHDKHHAAYVNKFNDAVAGT-ELDNQPIETIIKKFAE--DTSKQGIFNNAAQAWNHSFYWQCMKPNGGGNPTGVLADKINTDFGSFEKFIEAFKNAGATQFGSGWAWLVLD-G-DTLKVTKTPNADNPFTKNQIPLLTMDVWEHAYYLDYQNRRPDYINDFIKLVNWDFVAQNFSEAL-

>L0AA53_1056495_Thermoprotei_A_Arc

KRYELPPLPYSYDALEPVLSRDILTYHHDKHHLAYVNGANAAMEKLEKYLNGQEQS--IDIR----AVSRDFEFNYGGHLLHTLYWLNMAPTGGGTPGGTIADAINKNFGSFDKFKKVFGDAAKLVEGVGWAILALDPVTGDLKITQVEKHNAVITMNLVPLLACDVFEHAYYLQYKNDRGSYVDKWWDVVNWDDVEKRYQKALT

>L0AKN7_797304_Halobacteria_A_Arc

TDHELPPLPYEYDALEPAISEQVVTWHHDTHHQGYVDGLNSAEAELAENRESGEF---GSTP----GALSNVTHNGCGHYLHTLFWENMSPDGGGEPEGELADRIEEDFGSYEGWKGEFEAAAG--AAGGWALLVYDPVSKQLRNVAVDKHDQGALWGAHPILACDVWEHSYYYDYGPDRGSFIEGFFDVVDWDKVDEEYQKCLG

>L0HEM7_593750_Methanomicrobia_A_Arc

KLYTLPKLPYEYKALAPYISEEQLTLHHTKHHQAYVTGANAVFGKLDKARKDKAD---LDMK----ATLKELSFHIAGFRLHNIFWENLAPAGGGLPGGELAKAIDAEFGSFDRFKKEFTQAASSAEGSGWAVLTHCVKTNRLIIMQIEKHNVNLVPGFRILMALDVWEHAYYVDYKNDRAKFIEGFWNIVNWDSVNARFVTPLK

>L0HJ20_593750_Methanomicrobia_A_Arc

RLYSLPKLPYEYNALSPAISEEQLKLHHQKHHQAYVNGANAIYEKLDKARKENAT---ADMK----AVLKELSFHIGGFKLHNLFWENLAPAGGGAPKGELAKAITGEFGTFDRFKTEFTQAAVSAEGSGWAALTFCHKTKRPLVMQIEKHNTNVYPGFSILMVLDVWEHAYYLDYKNDRAKFVDAFWTIVNWDVVAQRFEAARK

>L0IC00_797302_Halobacteria_A_Arc

TEHELPPLPYDYDALEPSISEQVVTWHHDTHHQGYVNGLNAAEETLAENRESGDF---GSSP----GAIGNVTHNGCGHYLHTMFWENMSPNGGGEPEGDLADRIEEDFGSYEGWKGEFEAAAG--AAGGWALLVYDPVAKQLRNIVVDKHDQGALWGSHPILALDVWEHSYYYDYGPDRGDFIDSFFDVINWDSVAEEYQKCLD

>L0JLZ0_797303_Halobacteria_A_Arc

ADHELPPLPYDYDALEPALSEQVLTWHHDTHHQGYVNGLNSAEETLAQNREEGDF---GSTP----GALSDVTHNGCGHYLHTLFWENMSPNGGGEPEGDLADRIEEDFGSYEGWKGEFEAAAG--AAGGWALLVYDPVAKQLRNVAVDKHDQGALWGSHPILALDVWEHSYYYDYGPDRGEFIDGFFDVVNWEKAEEEYQTCLD

>L0JX01_694430_Halobacteria_A_Arc

TDHELPELPYDYDALEPAISEQVVTWHHDTHHQGYVDGLNSAEKTLEENRESGDF---SSSG----AAMENVTHNGSGHYLHTLFWENMSPDGGGEPEGDLADRIEEDFGSYEAWKGEFEAAAS--GAGGWALLVYDPVAKQLRNLKVDRHDLHALWGAHPILACDVWEHSYYYDYGPDRGEFIDGFFDVVNWDKAAEEYEKCLD

>L0JXL3_694430_Halobacteria_A_Arc

GRYALPELPYEYDALEPHIDERIMELHHSVHHQGYVDGANAALDTFEEMRSEGDY---EDVK----AAKRDFSFNLSGHINHTIFWQNMSPDGGGEPDGELGAAIDEQFGSFEAFREEFTAAAENVESNGWAMLFYEPVADLLVIGQIESQNGLAHQGAIPILTLDVWEHAYYLQYENERGSYVEEWWNVVDWDDVCRRYEFLTQ

>L0KZ58_867904_Methanomicrobia_A_Arc

ELYKLPALKYGYADLEPYISEEQLRIHHEKHHQAYVTNVNSLLQMMEKARREGTD---FDYK----ATAKAVAFNLGGHVLHDYFWWEMTPAATKEPVGELAEVIKEDFGNFERFKKEFTQTAASVEGSGWAALTFCNDTKRLGIMQIEKHNVNLFPDFPIIMAIDVWEHAYYLDYKNERGKFIDAFWNIVNWEEIDRYFRKVQQ

>L8D9X1_1268239_Proteobacteria_B_Mar

MAFELPSLPYAINALEPHMSQETLEFHHGKHHNTYVVKLNGLITGT-QFEGKTLEEIVCSS-------EGGVFNNAAQIWNHTFFWNSLSPNGGGEPSGKVAELINAKWGSFAAFKDAFNDKAVNNFGSSWTWLVQLAD-GSLDIVNTSNAATPLTQGVTPILTVDLWEHAYYIDYRNVRPNYLGGFWALVNWDFANSNLA----

>L8DAQ3_1268239_Proteobacteria_B_Mar

MAFVLPELNYSYDALEPHLDAKTMEIHYTLHHQTYVNKANDALLGTP-YDDASDEWLLKNIHTLPTNVQAAVRDHVGGHHNHSLFWTVMSAHGGHLNIGPLADEIQHAFGSFEAFRELFVKAALSRFGSGWAWLVIDTN-GKLSVTSTANQDSPLMQQLTPILGLDVWEHAYYLKYQNRRPEYINAFFNVIDWQEVERRYLAVM-

>L8LWB9_102125_Cyanobacteria_B_Ter

MAYQLPSLPYDYTALEPTISKSTLEFHHDKHHAAYVSKYNNAVQGT-EFDSKSIEEVIKAIAK--DESKTGLFNNAAQAWNHTFYWQCMKPNGGGTPTGELAKKIDADFGSFEKFAEAFKNAGATQFGSGWAWLVLD-G-DTLKVTKTLNADNPLTSNQVPLLTMDVWEHAYYLDYQNKRPAYIDDFLKLVNWDFVAQNLAAA--

>L8XU36_1261130_Proteobacteria_B_Mar

MAYTLPDLPYAHDALEPHIDTETMHLHHDKHHNTYVNNLNNAIANHPELAQLPIDELIAKLDVVPEEIRNAVRNNGGGHANHTLFWEIMAPNAGGEPTGEIKAAIEKAFGSFDAFKEKFAAAATGQFGSGWAWLVVNKD-GDLEVMSTSNQDSPLTIGKTPVLTIDVWEHAYYKKFSNVRPDYIKAFWNVVNWNEVNRRFLAAK-

>L9PNM3_1198452_Proteobacteria_B_Mar

MEHTLPPLPYAIDALAPHISQETLEYHYGKHHQTYVTNLNNLIKGT-EFENLSLEEIVKKS-------SGGIFNNSAQVWNHTFYWNGLTPNGKGAPDGALADAINAKWGSFDKFKEEFTKSCVGNFGSSWTWLVKKAD-GSLDIVNTSNAATPLTTDAKALLTCDLWEHAYYIDYRNVRPKYVETFFKLANWDFAAANFA----

>L9VRX1_1114856_Halobacteria_A_Arc

TDHELPPLPYDYDALEPSISEQVVTWHHDTHHQGYVNGLNAAEETLAENRESGDF---GSTP----GALSNVTHNGCGHYLHTLFWENMSPNGGGEPEGDLADRIEEDFGSYEGWKGEFEAAAG--AAGGWALLVYDPVAKQLRNVAVDKHDQGALWGSHPILACDVWEHSYYYDYGPDRGEFIDGFFDVVNWDSAAEEYQKCLD

>L9WFH7_1230460_Halobacteria_A_Arc

TDHELPPLPYDYDALEPSISEQVVTWHHDTHHQGYVNGLNAAEETLAENRESGDF---GSTP----GALGSVTHNGCGHYLHTLFWENMSPNGGGEPEGDLADRIEEDFGSYEGWKGEFEAAAG--AAGGWALLVYDPVAKQLRNVAVDKHDQGALWGSHPILALDVWEHSYYYDYGPDRGDFIDAFFDVVNWEKAAEEYEKCLD

>L9WKK3_1227499_Halobacteria_A_Arc

TDHELPPLPYDYDALEPSISEQVVTWHHDTHHQGYVNGLNSAEETLEENRESGDF---GSTP----GALGNVTHNGCGHYLHTLFWENMSPDGGGEPEGDLADRIEEDFGSYEGWKGEFEAAAG--AAGGWALLVYDPVAKQLRNLAVDKHDQGALWGAHPILALDVWEHSYYYDYGPDRGEFIDGFFEVVNWDSAAEEYQKCLD

>L9XNL7_1227499_Halobacteria_A_Arc

PSYQLPELPYEYDALEPHIDGRIMELHHGEHHQAYVDGANEALEEFESMREAGEF---DDIR----AAKRDFSFNYSGHVNHTVFWENMSPDGGGEPEGDLASAIDEQFGSFEDFQAEFTATANNVEGDGWAMLFYEPLADALVIGQVEGQNELAHQRAIPILTLDVWEHAYYLQYENDRGAYVDEWWNVVDWEDVGERYELLTD

>L9ZI72_1227494_Halobacteria_A_Arc

TDHELPPLPYDYDALEPALSEQVLTWHHDTHHQGYVNGLNAAEETLAENREEGDF---GSTP----GALKNVTHNGCGHYLHTLFWENMSPNGGGEPDGDLADRIEEDFGSYEGWKGEFEAAAG--AAGGWALLVYDPVAKQLRNVAVDKHDQGALWGAHPVLALDVWEHSYYYDYGPDRGDFIDAFFDVVNWEKAEEEYQTCLD

>M0AV65_29540_Halobacteria_A_Arc

PDYRLPELPYAYDALEPHIDARIMELHHGKHHQSYVDSANAAVEELDEMRANEDF---EGIR----STKRDLSFNLSGHVNHSVFWASMSPDGGGRPDGALATAIENQFGSFEAFQAEFTATATAVEGAGWAMLFYEPLADALVIGQVEDQNELAHQGATPLLPLDVWEHAYYLQYENDRGAYVDAWWNVVDWTAVGQRYEILAE

>M0AWE7_29540_Halobacteria_A_Arc

TNHELPPLPYDYDALEPSISEQVLTWHHDTHHQGYVNGLNSAEEELEQNRENGDF---ESTP----AAMENVTHNGCGHYLHTMFWENMSPNGGGEPEGDLATRIEADFGSYEAWKGEFEAAAG--AAGGWALLVYDPVAKQLRNLKVDRHDLHALWGSHPILALDVWEHSYYYDYGPDRGSFIDAFFDVINWESVEDEYQTCLD

>M0B3T7_29540_Halobacteria_A_Arc

SNHELPPLPYDYDALEPHISEQVLNWHHDTHHQGYVNGWNSAEETLEANREAGDF---SSSA----GAIRNVTHNGSGHVLHDLFWQSMSPEGGAEPTGDLAARIEADFGSYEAWKGEFEAAAS--AAGGWALLVYDSFSNQLRNIVVDKHDQGALWGSHPILALDVWEHSYYHDYGPDRGDFVDNFFEVVDWDEPSTRYEQAVE

>M0CBS2_1230457_Halobacteria_A_Arc

TDHELPPLPYDYDALEPSISEQTLTWHHDTHHQGYVNGLNAAEETLSENREEGDF---STTP----GALSNVTHNGCGHYLHTLFWENMSPNGGGEPEGDLADRIEEDFGSYEGWKGEFEAAAG--AAGGWALLVYDPVAKQLRNIAVDKHDQGALWGSHPVLALDVWEHSYYYDYGPDRGSFIDSFFDVVNWEKAEEEFQKCLD

>M0CPB2_797114_Halobacteria_A_Arc

SDPELPPLPYDYDALEPHISEQVLTWHHDTHHQGYVNGLESAEETLAENREAGDF---GSSA----GALGNVTHNGCGHYLHTLFWDNMDPNGGGEPDGDLADRIEEDFGSYEGWKGEFEAAAG--AAGGWALLVYDPVAKQLRNVAVDKHDQGALWGAHPVLALDVWEHSYYYDYGPDRGDFIDNFFEVVDWDEVADQYETAVG

>M0CQV9_797114_Halobacteria_A_Arc

SNYELDPLPYDADALEPHVSEQVLTWHHDTHHQGYVDGWNAAEDTLEDNREDGDT---DGSA----GAIRSVTHNSSGHILHSLFWQNMSPEGGAEPEGDLRERIEDDFGSYEAWKAEFEAAAS--DASGWALLVYDSFSERLRNVVVDKHDQGAIWGGHPVLALDVWEHSYYYDYGPARGDFVDAFFEVVDWDEPSERYAEAVE

>M0DHS9_1227487_Halobacteria_A_Arc

MSYELDPLPYDYDALEPHISEQVLTWHHDTHHQGYVNGWNSAEETLESNREEGDF---SSSG----SALRNVTHNGSGHILHDLFWQNMSPEGGEEPSGDLADRIEEDFGSYDAWKGEFEAAAK--NASGWALLVYDSFSNQLHNVVVDKHDQGALWGSHPILALDVWEHSYYHDYGPARGDFIDAFFEVVDWEESSSRYNEAVQ

>M0L696_358396_Halobacteria_A_Arc

TDHELPPLPYDYDALEPAISEQVVTWHHDTHHQGYVNGLNSAEETLAENRESGDF---DSTP----GALSNVTHNGCGHYLHTLFWENMSPDGGGEPEGDLADRIEEDFGSYEGWKGEFEAAAG--AAGGWALLVYDPVAKQLRNVAVDKHDQGALWGSHPILALDVWEHSYYYDYGPDRGSFIDAFFDVVDWDKAAEEYQKCLD

>M0LBE9_1227454_Halobacteria_A_Arc

RRYRLPDLPYEYGALEPHIDERIMELHHGEHHQAYVDGANEALEEFDTRRATDDF---AEIR----APKREFSFNYSGHVNHTIFWENLGPDGGGTPEGEFATAIDERFGSFETFQLEFSATADQVEGDGWAMLFYEPLADTLVIGQLEDQHELAHQAAVPILTLDVWEHAYYLQYENDREAYVEAWWNVVDWADVARRYDFVTD

>M0M2F6_1132509_Halobacteria_A_Arc

SHPELPPLPYDYDALEPHISEQVLEWHHDTHHQGYVNGLESAEETLAENRESGDH---STTG----GALNNVTHNGSGHYLHTMFWENMSPNGGGEPDGDLADRIAEDFGSYEGWKDEFEAAAS--AASGWALLVYDPVADQLRNVAVDKHDDGALWGAHPVLALDVWEHSYYYDYGPDRGSFVDAFFEVVDWDAAADNYEQTVS

>M0M5P3_1227454_Halobacteria_A_Arc

TDHELPPLPYDYDALEPAISEQVVTWHHDTHHQGYVNGLNAAEETLAENRESGDF---DSTP----GALSNVTHNGCGHYLHTLFWKNMSPDGGGEPEGDLADRIKEDFGSYEGWKGEFEAAAG--AAGGWALLVYDPVAKQLRNVAVDKHDQGALWGSHPILALDVWEHSYYYDYGPDRGSFIDGFFDVVNWDKAEEEYQTCLD

>M0MDJ5_931277_Halobacteria_A_Arc

SNPELPPLPYDYDALEPHISEQVLNWHHDTHHQGYVNGLDSAEETLAENRESGDY---SSTG----GALGDVTHNGSGHYLHTMFWENMSPNGGGEPSGDLADRIEEDFGSYEGWKGEFEAAAS--AASGWALLIYDPVADQLRNVAVDNHDEGALWGAHPVLALDVWEHSYYYDYGPDRGSFIDAFFEVVDWDAAEDNYQKTVS

>M0MM71_1227455_Halobacteria_A_Arc

SNAELPPLPYDYDALEPHISEQVLTWHHDTHHQGYVNGLNSAEETLAENRESGDY---SSTG----GALGNVTHNGCGHYLHTMFWENMDPNGGGEPSGELADRIEEDFGSYEGWKGEFEAAAS--AASGWALLVYDPVANQLRNVAVDNHDEGALWGAHPVLALDVWEHSYYYDYGPDRGSFIDAFFEVVDWDAAAENFEKTTS

>M1L4D0_1208918_Proteobacteria_B_Mar

MIHVLPDLPYKIDSLSPYISKETLEFHYGKHHQTYINNLNSLIQNT-EFQEMSLEEIIKKS-------SGAIFNNAAQVWNHNFYWNSICPDSTKKPEGNLADAINQKWGNFESFKLEFNKQAASNFGSGWTWLVKKSD-RSLEILNTSNAGTIVNSNDKAMITCDVWEHAYYIDYRNARVKYLENFWSIINWKFAEQNFSL---

>M1M096_1208922_Proteobacteria_B_Mar

MSYNLPPLPYDASSLSPYISKETIEFHYGKHHKTYVDNLNNLISDN-ELSKLNLDDIIKNS-------TGAIFNNAAQIWNHNFYWNSLSPIFNQHPSDILLKSIELKWGNFENFKESFTRSALSNFGSGWTWLVKKTD-GSLDIVNTSNANTPIITEDKALLTCDVWEHAYYIDYRNSRAKYLENFWEIINWNFVSSNFSG---

>M1XSP2_268739_Halobacteria_A_Arc

SNPELPPLPYEYDALEPSISEQVVTWHHDTHHQGYVNGLEAAEETLAENREAGEF---GSSG----AAIRNVTHNGSGHYLHTLFWENMDPNGGGEPSGELADRIEADFGSYEGWKGEFEAAAG--EASGWALLVYDPVSDQLRNLVVDKHDQGALWGSHPVLALDVWEHSYYYDYGPDRGSFIDGFFEVVDWDNVAEQYEKAIQ

>M1Z874_1288971_Firmicutes_B_Ter

AKYELPALPYAYDALEPYIDEATMKVHHTGHHAAYVNNLNAALDKHPELYEKSLEDLLASLDQVPEDIRTAVRNNAGGHWNHSFFWPLMKKNGGGTPSGELAKAIDAQFGSFDAFKEEFGKVAAGRFGSGWAWLLVEQG-GKLSLTSTPNQDNPLMEGKKPILGLDVWEHAYYLKYQNKRPEYIKAFWNVVNWEQVEKNFNQYK-

>M1ZG58_1288971_Firmicutes_B_Ter

MAFKLPELKYSYDALEPHIDGLTMETHYSKHHKGYVDNLNKALEGHSKFQKMSIEEILKSLDELPEEIRTAVRNNGGGHYNHTLFWEIMSPDGGGKPDGELAKKIDEDLGGFDKFKEELKKAALARFGSGWAWLVLN-N-GKLEIVSTPNQDNPISDGKPPILGIDVWEHAYYLKYKNLRADYIDAWWNVVDWKKVSEVFDKVK-

>M7Y218_1239962_Bacteroidetes_B_Mar

MAFELPKLPYDFNALEPHIDARTMEIHHGKHHNAYVTNLNNAIAG-TALEGKSLEELMK-VAGSN----TPVRNNGGGHYNHSLFWNLLSPNGGGKPTGELAAAIDAKFGSFDAFKEEFNKAAATRFGSGWAWLSVA-N-GQLVVSSTPNQDNPLMDKGFPILGLDVWEHAYYLHYQNRRPDYIAAFWNVINWDEVAKRYAAAK-

>M7YBF0_1239962_Bacteroidetes_B_Mar

TGFAQTPLAYAYNALEPHIDAMTMEIHYTKHAAAYANNLKDAARDEK-VTSKPLEDVMKNISKY----SVKMRNNGGGHYNHELFWKIMSPKGGGEPAGDLAKAIAADFGSFAAFKTKFEDAAKARFGSGWAWLSVDAN-KKLVVSSTPNQDNPLMDKGTPILGLDVWEHAYYLKYQNRRPDYITAFWNLVDWNAVAERYKMAVK

>O84296_272561_Chlamydiae_B_Mar

SSYMLPALPYDYDALEPVISAEIMQLHHQKHHQGYINNLNEALKSLDVANATQDL---ARLI----AINPALRFNGGSHINHSLFWEMLAPQGKGPPRHELLKLIEKFWGSFDNFLKNFITSSAAVQGSGWGWLAFCPQKQELMVQTTANQDPLATTGMIPLLGVDVWEHAYYLQYKNARMDYLKSFPSIINWDYIENRFVEMSK

>O93724_178306_Thermoprotei_A_Arc

KRYTLPPLPYAYNALEPYISAEIMQLHHQKHHQGYVNGANAALEKLEKFRKGEAQ---IDIR----AVLRDLSFHLNGHILHSIFWPNMAPPGGGKPGGKIADLINKFFGSFEKFKEEFSQAAKNVEGVGWAILVYEPLEEQLLILQIEKHNLMHAADAQVLLALDVWEHAYYLQYKNDRGSYVDNWWNVVNWDDVERRLQKALN

>P09224_64091_Halobacteria_A_Arc

SQHELPSLPYDYDALEPHISEQVVTWHHDTHHQSYVDGLNSAEETLAENRETGDH---ASTA----GALGDVTHNGCGHYLHTMFWEHMSPDGGGEPSGALADRIAADFGSYENWRAEFEVAAG--AASGWALLVYDPVAKQLRNVAVDNHDEGALWGSHPILALDVWEHSYYYDYGPDRGSFVDAFFEVIDWDPIAANYDDVVS

>P09737_64091_Halobacteria_A_Arc

SEYELPPLPYDYDALEPHISEQVLTWHHDTHHQGYVNGWNDAEETLAENRETGDH---ASTA----GALGDVTHNGSGHILHTLFWQSMSPAGGDEPSGALADRIAADFGSYENWRAEFEAAAS--AASGWALLVYDSHSNTLRNVAVDNHDEGALWGSHPILALDVWEHSYYYDYGPDRGSFVDAFFEVVDWDEPTERFEQAAE

>P18868_187420_Methanobacteria_A_Arc

KFYELPELPYPYDALEPHISREQLTIHHQKHHQAYVDGANALLRKLDEARESDTD---VDIK----AALKELSFHVGGYVLHLFFWGNMGPACGGEPSGKLAEYIEKDFGSFERFRKEFSQAAISAEGSGWAVLTYCQRTDRLFIMQVEKHNVNVIPHFRILLVLDVWEHAYYIDYRNVRPDYVEAFWNIVNWKEVEKRFEDIL-

>P77968_1111708_Cyanobacteria_B_Ter

MAYALPNLPYDYTALEPCISKSTLEFHHDKHHAAYVNNFNNAVAGT-DLDNQSIEDVIKAVAG--DASKAGIFNNAAQAWNHSFYWNCMKPGGGGQPSGALADKINADFGSFDAFVEAFKQAGATQFGSGWAWLVLD-N-GTLKVTKTGNAENPMTAGQTPLLTMDVWEHAYYLDYQNRRPDYIADFLKLVNWDFVAANLAAA--

>P80857_273057_Thermoprotei_A_Arc

KKYELPPLPYKIDALEPYISKDIIDVHYNGHHKGYVNGANSLLERLEKVVKGDLQTGQYDIQ----GIIRGLTFNINGHKLHALYWENMAPSGGGKPGGALADLINKQYGSFDRFKQVFTETANSLPGTGWAVLYYDTESGNLQIMTFENHFQNHIAEIPIILILDEFEHAYYLQYKNKRADYVNAWWNVVNWDAAEKKLQKYLT

>Q02A56_234267_Acidobacteria_B_Mar

MPFTLPPLPYAPDALEPHIDKMTMEIHHGKHHNAYVTNLNKALESAPELADKNIEELLANCAIVPEKIRTAVRNNGGGHINHSMFWKIMGPNGGGAPVGNVAQAITGAFGSFDAFKEKFNAAGVGRFGSGWAWLLKT-S-SGVEITSTANQDSPIMEGKVPVFGCDVWEHAYYLKYQNRRPDYLAAWWNVVNWKEIEDRFNAR--

>Q03300_309800_Halobacteria_A_Arc

SDYELDPLPYEYDALEPHISEQVLTWHHDTHHQGYVNGWNAAEETLAENREAGEF---GSSA----GALRNVTHNGSGHILHDLFWQNMSPEGGDEPEGALAERIAEDFGSYEAWKGEFEAAAG--AAGGWALLVYDSFSNQLRNVVVDKHDQGALWGSHPILALDVWEHSYYHDYGPARGDFVSAFFEVVDWDEPAARYEQAVE

>Q03301_309800_Halobacteria_A_Arc

MSYELDPLPYEYDALEPHISEQVLTWHHDTHHQGYVNGWNAAEETLAENREAGEF---GSSA----GALRNVTHNGSGHILHDLFWQNMSPEGGDEPEGALAERIAEDFGSYEAWKGEFEAAAG--AAGGWALLVYDSFSNQLRNVVVDKHDQGALWGSHPILALDVWEHSYYHDYGPARGDFVSAFFEVVDWDEPAARYEQAVE

>Q03302_272569_Halobacteria_A_Arc

SNPELPPLPYDYDALEPHISEQVLTWHHDTHHQGYVNGLESAEETLAENRDAGDF---GSSA----AAMGNVTHNGCGHYLHTLFWENMDPNGGGEPEGELLDRIEEDFGSYEGWKGEFEAAAS--AAGGWALLVYDPVAKQLRNVPVDKHDQGALWGSHPILALDVWEHSYYYDYGPARGDFIDAFFEVVDWDKAAEEYEKSVS

>Q08713_330779_Thermoprotei_A_Arc

KRYEFPQLPYKVDALEPYISKDIIDVHYNGHHKGYVNGANSLLDRLEKLIKGDLPQGQYDLQ----GILRGLTFNINGHKLHAIYWNNMAPAGGGKPGGALADLINKQYGSFDRFKQVFSESANSLPGSGWTVLYYDNESGNLQIMTVENHFMNHIAELPVILIVDEFEHAYYLQYKNKRGDYLNAWWNVVNWDDAEKRLQKYLN

>Q08ZM8_378806_Proteobacteria_B_Mar

APFTLDDLPYAYEALEPVIDAETMRIHHGAHHKAYVDNLNKAVAANSALAGQSLDKILANVSRYD----AAVRNNAGGHYNHTLFWKLMAPPGGGAPSKALADQITKDFKSIDEFKKAFAEAGTKRFGSGWAWLVWT-G-DKLQVGSTPNQDNPLMDKGTPVIANDVWEHAYYLKHRNKRDSYLSGWWEVLNWNEANRLFDEARA

>Q09C50_378806_Proteobacteria_B_Mar

------DKKYTPPELKG-ISDAVLETHF-KLYEGYVNRANKLTESLSGLATQGEASTNPVYA----ELTRRLGFEYNGMVLHEYYFGNLKPGG-TPPGDRLKKAFEASFGSFENWLTDFKAVAT-MPGIGWAVTFQDPRNGWLSNHWITLHETNNIAGFTPIIVLDAWEHAFVPDYANERAKYVDAYFSNIDYEAAQARLNVK--

>Q09CK0_378806_Proteobacteria_B_Mar

MPFMLPELPYPKDALAPHMSAETLDYHHGKHHATYVNKLNELLAGK-PEADKSLVDIILSS-------DGPVFNNAAQVWNHTFFWHCMKPSGGGAPAGELADAINRDFGSYDNFKKAFSEAATTQFGSGWAWLVQD-G-SKLAVMKTANADLPMKHGKKALLTIDVWEHAYYIDYRNARPKFIETFLSLVNWDFVAGNLKNP--

>Q0AW95_335541_Firmicutes_B_Ter

MAHTLPDLPYAYDALEPFYDEQTVRLHHDMHHKAYVDGLNNAESKLAEALEKGDF---ALIK----HIERELSFHGSGHILHTMFWENMKPGGGGPADGAVAELIDRDFGSFDNFKKLFSASALAVEGSGWTILACNPIFEKLVVLQAEKHQDLTQWGAVPLLIVDVWEHAYYLKYQNKRAAWIEAWWNLINWDDVNRRVALMQQ

>Q0F8L9_367336_Proteobacteria_B_Mar

MAFELPELPYSHDALAKGMSAETLEFHHDLHHKAYVDNGNKLISGT-EWENKSLEEITIGTYNSTSVSQNGIFNNISQLWNHNQFWEMMGPDGR-AMPSELEAAIVESFGSVEAFKSDFGAAGVGQFGSGWCWLVKDTD-GSLKITKTENGVNPLCFGQKTLLGCDVWEHSYYIDFRNKRPVYISNFLNLVNWENVAARLASS--

>Q18HG3_362976_Halobacteria_A_Arc

MSYELDPLPYEYDALEPQLSEQVLTWHHDTHQQGYVNGWNNAEAELEENRDSHDF---SSSA----GAIRDVTHNSSGHLLHDLFWQCMSPEGGDEPTGDLGDRIEEDFGSYDAWRGEFEAAAG--DASGWALLVYDSFSNQLRNVVVDNHDEGAFWGAHPVLSLDVWEHSYYHDYGPARGDFVDAFFEVVDWEEPSQRYAQAVE

>Q1ARZ8_266117_Actinobacteria_B_Ter

MAYELPPLPYDYNALEPYIDEATMRFHHDNHHNTYVTNLNAALEKHPDVDPGNVDELIADLNAIPEDIRRAVRNNGGQHSNHSIFWQIMAPSGGGEPTGELGEAINGTFGSFDAFKEQFAAAAAALFGSGWCWLVVTSG-GQLAIKTTPNGDSPYMEGEIPVIGLDCWEHSYYLKYQYRRPEYVQNWWNVVNWEEANRRYQAARS

>Q1LSZ4_374463_Proteobacteria_B_Mar

MIFMLPTLPYRFDAFEPFIDQTTMEIHYTKHHQNYITNANLILESLPQFAQLSVEELIQQLDQVPHNKKMALRNYAGGHANHSLFWKLL--KHGTKIQGKLKNAIESDFGSIIIFQELFEQVAMNCFGSGWAWLVKK-N-NQLLVVSTANQDNPLMGSGYPILGLDVWEHAYYLKYQNRRSDYIKSFWNIVNWDEASIRFNQCED

>Q26GK1_156586_Bacteroidetes_B_Mar

MAFELPKLNYAYDALEPHIDARTMEIHHSKHHNGYTTKLNNAIEG-TDLEGKSIEDILANLDMDN----KAVRNNGGGFYNHRLFWEVMSPNGGGKPTGDLAAAIDSAFGSYDAFQDKFATAAKTQFGSGWAFLCVK-D-GALEVCGTPNQDNPLMPGGTPILGLDVWEHAYYLNYQNRRPDYVSAFFNVIDWDAVSEKYNAAK-

>Q2FSC2_323259_Methanomicrobia_A_Arc

KKYTLPALSFEYGALAPFITEKQLTLHHQKHHQAYVTGANAIFEKLEMARREGGD---LDQK----ALLKELSFHIGGHRLHTLFWENLAPAGGGVPSGILADWINRDFGSLDRFKKEFTQTASSVEGSGWAVLSVCLGTQRLLLMQVEKHNVHVYPGFRILMVLDVWEHAYYLDYMNDRAKFIENFWNIIRWDMVNQRLEAALK

>Q2S1T9_309807_Bacteroidetes_B_Mar

MAFDLPDLPYDYDALEPHIDEQTMRIHHDKHHAGYTRKLNNALEGHDDLQEHSIEELLAGLDTLPTDVQTPVRQNGGGFYNHRLFWNVMSPDGGGTPDGDLADAIHDAFGSYEDFKDAFADAATGQFGSGWGWLVAQPN-GDVTVTSTPNQDNPLLEGHTPILGIDVWEHSYYLNYQNERGTYVDEWWNVVDWDAVGENYDEIVG

>Q3SGN8_292415_Proteobacteria_B_Mar

FPYELPPLPYGPDALRPLISGNTIRFHHREHHQRYIDGLNRGIDDT-EFGAMPLRAVIRATAG--KPETAVIFNNAAQAWNHAFYWDSLTPQGGGLPPPALRKMIDASFGSVDACLRQLAHAAIAQFGTGWVWLARD-D-KKIRVVRTCGADNPLTRELTPLLAIDLWEHAYYLDYQNRLEDHVSGVIRLVNWDFAAKNLV----

>Q3SK26_292415_Proteobacteria_B_Mar

MEHTLPALPFAMDALEPHMSKETFEYHYAKHHQAYVTNLNNLIKGG-EFESKSLEDIVKTAP------AGGIYNNAAQVWNHTFFWNCLTPNGGGAPSGALADAINKKWGSLDEFKKAFQTSAVGNFGSGWTWLVKKAD-GSVDIVNMGAAGTPLTTGDKALLCVDVWEHAYYIDYRNLRPKFVETFLNMVNWKFAEANFA----

>Q3YRT4_269484_Proteobacteria_B_Mar

--FTLPELPYQQSDLVPYLSPEILGYHYNKHHQGYVNTLNSLVVGT-DFSEEDLPKIIEATAG--DLGSRSIFNNAGQIWNHNFYWESMKKNGGGAPTGKLLDKINEDFGSIDDFNNAFTNAGKSHFGSGWVWLVFDSE-QKLKILCTSNGDTPITQETHPLLTMDVWEHAYYLDYFNVRQNYVETFLHLVNWDFAAQRFLEV--

>Q3Z7W8_243164_Chloroflexi_B_Ter

--------MYTASALLG-FSDTLLKNHF-TLYQGYVNNTNKLSDTLKTMLAEGKT-AAPEYA----ELKRRFGFEFDGMRLHEYYFSNLGKSAPLSNTGKLYQALSAEFGSYQLWETDFKATAS-MRGIGWVILYKDSQTGRLFNQWINEHETGHLAGITPILVIDIFEHAFMTDYGLKRADYIAAFLKNINWDEAEKRFQN---

>Q67QL1_292459_Firmicutes_B_Ter

MAFQLPALPYPTNALEPYIDAQTMEIHHGKHHAAYVNNLNAALEGHPELQSKSIEELLRGIDSVPESIRTAVRNNGGGHANHTLYWEIMTPGGAKEPGGELAEAINAAFGSFQNFKDEFAKAGAGRFGSGWAWLVVTKD-GKLAVYSTANQDSPLMQGDTPILGMDVWEHAYYLKYQNRRPEYIQAFMNVINWDKVAERYAAARK

>Q67T03_292459_Firmicutes_B_Ter

GGHRLPPLPYPYDALEPYIDAETMRLHHDRHHRSYVEGLNRAERALAEARAAGDW---ALVK----HWERELAFNGAGHYLHTLFWESMAPGAGGEPGGEVLAQIRQDFGDFRRFREQFSKAAENVEGGGWAVWVWAPRANRTEILTAEKHQNLSQWDVVPLLPLDVWEHAYYLKYRNDRAAYIEQWWNVVNWPAVERRLREARR

>Q6L1T7_263820_Thermoplasmata_A_Arc

MAETWE----VKEKLKPRISDQQIDYHFDFHYKGYVTKLNEIWSKLPDVDLSKANQNYSDLR----EMKLEETFNYDGSMLHEYYFESLTKD-HTEIPASVKEQIEKDFGSYEKFVALFKATGT--AFRGWAHLIFDLNYGKLRIVGADIHSAGAIWNAIMILPLDVYEHAYYTDYGAKRAPYLDAFMKNVNWKVVEKRLEKAKK

>Q6MHD3_264462_Proteobacteria_B_Mar

--FKLPNLPYAKTGIAPLFNEEQMTYHYDKHHKAYIDNLNKFMEDA-SLKGKSLEEITLTS-------TGGIFNNAAQAWNHTFFWFGMSPAGGGQPSAELSAAITRDFGSMDELKAKFVDGGVKTFGSGWIWLCMDAA-GKLSLVSTSNAAVPFTNGPTPLLVADVWEHAYYVDYRNLRAKYLETFWAQVNWNFVSENFASKKV

>Q6MKI3_264462_Proteobacteria_B_Mar

MTFELPALPYAKDALAPHMSAETLEYHHGKHHKTYVDNLNKLVPGT-EHEGKTLEQIIMSS-------SGGVFNNAAQIWNHTFFWNCLSPKGGGEPTGELAQAIVRDFGSIEKFKELFADASIKQFGSGWGWLVKNKE-GKLEILSTSNAETPMTKGHTAILTCDVWEHAYYIDYRNSRPNFLAAFWKLVNWEFAAKNFKG---

>Q6YQD2_262768_Tenericutes_B_Ter

MNFTLLSLPYQYDALEPFFDTQTMQLHHLKHHQTYINNLNDALKKHPQL-NLSLEQMLTDLSLVPQDIRQTVRNNGGGHFNHSFFWNILKVNNGNTPQGLLKEMIDCEFGSIDSFKDKFANAAKTIFGSGWAWLVLTPQ-QKLAITFTPNQDVVLNQ-GTPLLGLDVWEHAYYLSYQNRRVDYIEAFFSVLDWEKVQNNLTQTLK

>Q7M864_273121_Proteobacteria_B_Mar

NPFTLAPLPYAFDALEPAIDKETMQIHHGKHHQAYVNNLNAQVATYPELAKMSLEQIMAKISSY----NMAVRNNGGGHYNHALFWTLMAPVGGGTPSSALAKAIDRDFGSLDKMKEAFEKAGATRFGSGWAWVVVTAD-KKLAITSTPNQDNPLMDKGTPILALDVWEHAYYLKYQNRRGDYLKEWWKVVNWNEVNKLFDQAVK

>Q7M8L0_273121_Proteobacteria_B_Mar

--FTLRKLPFEAQQVASFISQETLDYHHGKHHAAYVNNLNNLTKEG-EFSASSLWDIIQKS-------QGGIFNNAAQVFNHDFYWDCIATAPQEIP--KLKLALKAGFGSVEAFKEAYLKAATTLFGSGWAWLVLDLE-GKLEIVQTQNAATPLTSGKIPLLVCDVWEHAYYVDFRNARPGYLDKFWESINWDFVTKNYELGLH

>Q8TQG9_188937_Methanomicrobia_A_Arc

DLYKLPPLKYGYADLEPYISEEQLRIHHDKHHQGYVNNTNALLEMMDKARKEDTD---FDYK----ATAKALSFNLSGHVLHDFFWWEMTPAASKEPVGEFAEAIKEDFGSFERFKKEFSKVASSVEGSGWAALTFCKGTKRLGIVQIEKHNVNLVPDFPIIMDLDVWEHAYYIDYKNDRGKFIEGFWNIIDWEELDKYFKKIQK

>Q96Y84_273063_Thermoprotei_A_Arc

KKYELPPLPYKVDALEPYISKDIIDVHYNGHHKGYVNGANSFLERLEKIIRGEITSGQYDIQ----GLLRGLVFNINGHKLHALYWQNMAPAGGGKPGGALADLIDKQYGSFDKFKQLFTEAANSLPGTGWTVLYYDTESGNLEIMTFENHFQNHIAELPIILILDEFEHAYYLQYKNKRADYVNAWWNLVNWDEADKKLQKYLN

>Q9HM56_273075_Thermoplasmata_A_Arc

MAETWE----IKEKLKPRISDVQIDNHFDVHYKGYVNKLNEIWSRLPDVDRSKANQNYSEFR----ALKLEETFNYGGSLLHELYFEGLTPK-HSEVPKEFKDAVAKDFGSYEKWLEDFKATGT--AFRGWAILVFDLNYGKLRNIGSDAHNVGLIWNSIAILTMDVYEHAYYVDYGAKRAPYLDAFLKNVNWPVVLDRLNRAKK

>Q9RUV2_243230_Deinococcus-Thermus_B_Ter

MAYTLPQLPYAYDALEPHIDARTMEIHHTKHHQTYVDNANKALEG-TEFADLPVEQLIQQLDRVPADKKGALRNNAGGHANHSMFWQIMGQNGANQPSGELLDAINSAFGSFDAFKQKFEDAAKTRFGSGWAWLVVK-D-GKLDVVSTANQDNPLMGSGTPILGVDVWEHAYYLNYQNRRPDYLAAFWNVVNWDEVSKRYAAAK-

>Q9Y8H8_272557_Thermoprotei_A_Arc

KRYELPPLPYNYNALEPYIIEEIMKLHHQKHHNTYVKGANAALEKIEKHLKGEIQ---IDVR----AVMRDFSFNYAGHIMHTIFWPNMAPPGGGTPGGRVADLIEKQFGGFEKFKALFSAAAKTVEGVGWGVLAFDPLTEELRILQVEKHNVLMTAGLVPILVIDVWEHAYYLQYKNDRGSYVENWWNVVNWDDVEKRLEQALN

>Q9ZD15_272947_Proteobacteria_B_Mar

YPFILPDLPYDKESFKPHFTRETFDYHHGKHHNSYVQNLNNLIKDREELQKKDLEEIIEWSSQ---NAEVAILNNASQIWNHTFFWYSIKPHGGGKPSGKVFEQISKDFGSFEQFCAQFKQEAVGQFGSGWTWVVYH-D-NKLQIIKTSNAGTPIVNFMKPILACDVWEHAYYIDYRNKRSDYIDIFIHMINWKFVEDNLIQ---

>R4W031_1333523_Halobacteria_A_Arc

SNPELPPLPYDYDALEPSISEQVLHWHHDTHHQGYVNGLDAAEETLADARESGDY---SATA----GALGDVTHNGSGHYLHTLFWENMDPNGGGEPDGDLRERIEADFGSYEGWKGEFEAAAS--AAGGWALLVYDPVAKQLRTVAVDKHDQGALWGAHPILALDVWEHSYYYDYGPDRGSFVDAFFEVVDWNAVADEYATVVE

>R4W6K3_1333523_Halobacteria_A_Arc

SNPELPPLPYDYDALEPHISEQVVTWHHDTHHQGYVNGLDSAEETLTENRSEGDF---GSSA----AAIRNVTHNGCGHYLHTLFWENMDPNGGGEPDGDLRERIEEDFGSYEAWKGEFEAAAS--AAGGWALLVYDPVAKQLRNLVVDKHDQGALWGSHPVLALDVWEHSYYHDYGPARGDFVDNFFEVVDWDNVASQYDDAVS

>R5HFY3_1263001_Firmicutes_B_Ter

YPFALPPLPYAYDALEPYVDEATMHFHHDKHLKTYVDNLNKALEAYPQYHTWTLETLLTKLEELPDGLRTAVRNNGGGVYNHDLFFDLMAPAGQ-----KISPAVAERFGGEEAWQKEMKAAALGQFGSGFAWLVADSS-GDLHIIALPNQDNPLSQGLTPILPLDVWEHAYYLKYQNLRADYIDAWFHVINWDGVNKRLHRG--

>R5JCK6_1262737_Bacteroidetes_B_Mar

MTYEMPKLPYANNALEPVISQQTIDYHYGKHLQTYVNNLNSLAPGT-EFEGKTVEEIVAKA------PDGAIFNNAGQVLNHTLYFLQFAPKSKKEPSGKLAEAIKRDFGSFENFKKEFNAAAVGLFGSGWAWLSVDKN-GKLHITKEANGSNPVRAGLKPLLGFDVWEHAYYLDFQNRRADHVNALWDIIDWEVVDKRM-----

>R5L6H9_1262760_Spirochaetes_B_Mar

--FELIKLPYGKEDLAPYMSSNTLDFHHGKHLNAYVTAVNDFVSDK-SLEGKSIEELILLSHN--NADKQGLFNNAGQVYNHEEFFKVLKKSEKPSIPSELESKIKSDFGSFDAFKEAFTTAGKTQFGSGWAWLVLA-N-GKLEVRKYPNAMNPIADKVHGLLTCDVWEHAYYLDYQNRRPDFLNTFVHLVNWEYVAEKLKNAK-

>R5NVX0_1262909_Bacteroidetes_B_Mar

MKFKLLPTLYPEDALEPYISKKTVEFHYGKHLAGYIQTTNNLKADT-EYKDLSIEEIMLRA-------DGKLFNNAAQVYNHYFQFEALAPKKDNAPEGKIKKNIEGTFGNLDTFKQKFAEAATTLFGSGYVWLVASAD-GKLELVQTKNAENPLTAGKTPVLNLDVWEHAYYLDVQNLRAKYVENFWNIVDWEKVNKRLECIK-

>R5P756_1262909_Bacteroidetes_B_Mar

KKIDMPHLPYAMNALEPIISEETMNYHYGKHLQAYVDNVNRLIIGT-PFENSDLETIVKYA-------TGPIYNNGSQAYNHTIYFLTFSPDAVHQPSGDLLKAIEKKWGSFENFKNVFSQSAASLFGSGWVWLAKNNQ-GELFIFQEPNGGNPLSRGYIPLMGMDVWEHAYYLDYQNRRPEHIEKLWTIIDWNTVGQRYGS---

>R5PAP5_1262909_Bacteroidetes_B_Mar

KQFTLPQLPYSADALAPVISKETIDYHYGKHLQTYINNLNKLIAGT-EFENADLETIVKKS-------DGAIFNNAAQTWNHTIYFNTFSPNARQYPEGKLRAAIEKEWGSFDNFKKEFTAAGSAIFGSGWVWLAKTPE-GQLVILKESNAGNPLTKGYTPLLGIDVWEHSYYLDYQNRRADHLEALWTIIDWKSIESRF-----

>R6A170_1262986_Proteobacteria_B_Mar

MAFTVPPLPFAMDALEPYMSSKTLSYHYGKHNKAYNDTLNNLIKGT-DYETMPLEEIIRTT------QSGPIFNNAAQCFNHTFFWNSLKPNGGGQPTDVIAAQINAKWGSYDAFADAFTKSAVGNFGSGWTWLVEKED-GTLDIVNTSNAGTPITGTDVPLITCDVWEHAYYLDYYNMRVTFVQTFLHLVNWDFAAKNYK----

>R6EU58_1262994_Firmicutes_B_Ter

YPFVVQPLPYEYDALLPVLDEETLHFHHDKHYQTYVDKLNAILADYPQLQQMTLTELLTSLASLQEEARESIHNNGGGVYNHQLYFDSMRSPVGQEPCGALEEALIRDFGSVRQWKEQMSQSAIGVFGSGWAWLVSDQD-GTLMILTTANQDVPDLRLYAPILLIDVWEHAYYLQYRNRRPDYVQGWHKLLHWKKAERRYEQVLC

>R6GYH2_1262911_Firmicutes_B_Ter

YPFILPPLPYPPAALEPNLGRSSVRLHHDAFFAAYVDRLNGALAPWLQYQDWPLERLLLHWCQLPRALGQAVRRYGGGTYNHTLYFSSLAPARTTKPSPVLLAEIEHSFGSMEGLHRSMKNAAASIFGSGWVWLVCC-S-GGLQIVCTANQDTPLP--LWPLVNMDLWEHAFLPDYENRRDDYMEAALAIIDWDAASRRFEERTA

>R7QTT1_1262942_Firmicutes_B_Ter

YPFQNPPLPYAYDALEPYIDEKTMHLHHDRHLQTYVDNLNAALLPHPELHDKSLVFLLTHQNLLPCGTQIPIRNNAGGVYNHIFYFSGLTPDADR-PPDPLSAFLIESFQSPEKFKEKFKAATLSVFGSGYAWLTIEPT-GRLCIFVTKNQDTPLPAGLFPLLNIDVWEHAYYLKHYNERAAYIDDWFHIADFARANEHLLSFLH

>S0AS08_333146_Thermoplasmata_A_Arc

ATETWE----VKENAKPRISDKQIDYHFDVHYKGYVSKLNEIWSKLPDVDLTKANQNYSDLR----EMKLEETFNYDGSMLHEYYFESLSKD-HVAMPESVKAQIEKDFGSYEKFVALFKATGT--AFRGWAHLVFDLNCGKLRVLGADIHNASAIWNALMILPLDVYEHAYYTDYGAKRAPYLDAFMNNVNWKVVEKRLDRAKR

>S0L434_1140003_Firmicutes_B_Ter

MAYELPELPYAYDALEPQIDVETMHLHHDKHHNTYVTNLNAAVEKYPELAEKSVEDLIADMDAIPSDIQTAVRNNGGGHANHSFFWEILTPNATEEPVGELKTAIEDTFGSLDALKEEFKKAATGRFGSGWAWLVVK-D-GKLAVTSTANQDSPLMEGQTPVLGLDVWEHAYYLNYKNVRPDYIDAFWKLVNWEKANELFVAAK-

>S8FFI4_888054_Bacteroidetes_B_Mar

MKVELPRLPYAANALEPVISAQTILLHHGKHFQNYINTLASLVKGT-EFENKSIEEIVQTV------PDGPIFNNAGQSLNHAFYFSQFSPVSNNVPHGKMAKAIDVAFGSFEEFKKQFSQAAATLFGSGWAWLSQDKD-GNLVITKESNAGNPFRRGNNPLFGLDVWEHAYYLDYQNRRADHIAAVWEIVDWEIVEKRLK----

>S9QPA8_1123237_Proteobacteria_B_Mar

MAFELPDLPYAHDALASKMSKETLEYHHDIHHNTYVTTLNKLIDGT-EWADKSLEEIIKGTYDSGAVAQSGLFNNASQHWNHTQFWEMMGPGDN-SVPGALEKAIVEAFGSFDKFKEDFAAAGAGQFGSGWAWLVKDTD-GSLKVTKTENGVNPVCFGQTALLGCDVWEHSYYIDFRNKRPAYLSNFLNLVNWENVASRM-----

>S9RPC2_1123237_Proteobacteria_B_Mar

MAFELPALNFAHSALAGRMSQETLELHHDKHHQAYVTALNGFVENA-DLQGKTLEEIVTATYA--DAEREGIFNQAGQHWNHIHFWNALSPQGGG-IPGTLEARLVADFGSVADFKKAFKTAATGQFGSGWAWLIQKPG-GSLGVTKTPNGVNPLATGETALLGLDVWEHSYYVNFRNRRPDYVDNFLHLANYEFAESNLA----

>T0LM88_667138_Thermoplasmata_A_Arc

KKYELPQLPYRIDGLEPHISKDIVDVHYNGHHKGYVNTANNLIDRLNGIVKEEVKS--YDIH----GVLRNLTFNINGDKLHTLYWNNMAPEGGDKPGGKLGDLIEQQYGSYEKFRKLFTEAANSNPGTGWAVLTYDKENSNLNVMTVENHFMNHLAEMPIVLILDEFEHAYYLQYKNKRADYVGAWWHIVNWDEANKKLEKLL-

>T0M4N9_667135_Thermoplasmata_A_Arc

MAENWQ----IKDKLKPSISDKQIDYHFETHYKGYVNKLNEIWEKLPNADRSKANQNYSEFR----ELKLEETFNFDGSLLHETYFANLAKE-HAPVSESFKKQVEKDFGSYEKWVEDFKATGV--AFRGWSLCVFDLNTGKLRNIGADVHNTNGIWNAILVLALDVYEHAYYTDYGPKRAPYLDAFLKNVKWSDVEQRLEKAKK

>T0MPA5_667137_Thermoplasmata_A_Arc

MTEKWE----KKTTLKPKISDQQIEYHFETHYNGYVTKLNEIWEKMQTVDRSKANQNYSEFR----ELKLEETFNYDGSLLHELYFENLHSG-SSSAPESFKSQVAKDFGSYEKWLEDFKATGI--AFRGWSLLVFDLNTGKLRNIGADVHNTNGIWNAIVILSLDVYEHAYYTDYGPKRAPYLDAFMKNVNWSEVEKRLQKAQK

>T0MZU0_261391_Thermoplasmata_A_Arc

MVEKWE----KKNQFKPKISDQQIEYHFETHYNGYVTKLNEIWEKLPNADRSKANQNYSEFR----ELKLEETFNYDGSLLHEIYFESLKKDGLKNLSEELKKKISEDFGSYEKWVEDFNATGT--AFRGWALLVYDLNTGKLRNIGADVHNTNGIWNAIVVMALDVYEHAYYVDYGAKRAPYLDAFMKNVDWASVNKRFEKAHK

>U1MGY5_1325472_Halobacteria_A_Arc

SQPQLPELPYEYDALEPHISEQVVNWHHDTHHQSYVNNLAAAEETLAENREAGDY---DGTA----AAIRDVTHNTGGHYLHTLFWENMHPDGGGEPSGELRDRIETDFGSYEGWKGEFETAAS--DASGWALLVYDPVTKQLRNATVDNHDEGAIWGAHPILSLDVWEHSYYYDYGPDRGGLIDGFFEVVNWDYVADQYDTVVS

>U1QK35_1070774_Halobacteria_A_Arc

MSYELDPLPYEYDALEPHLDEQVLTWHHDTHHQGYVDGWNSAEETLAENRENHDF---GSSA----GAIRDVTHNSSGHILHDLFWQNMSSEGGDEPSGDLADRIAEDFGSYEAWKGEFEAAAG--SASGWALLVYDTFSNQLRNVVVDNHDEGAVWGGHPVLALDVWEHSYYYDYGPNRGEFVDNFFEVVDWDEPSARYQQAVE

>U1QN78_1085028_Halobacteria_A_Arc

SNPELPSLPYDYDALEPSISEQVVTWHHDTHHQSYVDNLTAAEETLAENRESGDY---GGSA----AAIRNVTHNTGGHYLHTLFWENMHPNGGGEPEGELRDRIEADFGSYEGWKGEFETAAG--DASGWALLVYDPVSKQLRNAVVDNHDEGAIWGSHPILSLDVWEHSYYYDYGPDRGSFVDGFFEVVDWSKVSEEFQTCLD

>U2EL32_1033802_Proteobacteria_B_Mar

MAFELPDLPYDYDALEPYIDGRTMEIHYEKHHNAYLTKFKKAIEG-TELEDQDLETILSKAGQHG----PGVRNQGGGFYNHILFWDSMSPSGGGKPTGGLGTAIDSSFGSFDSFKEEFTNAATGLFGSGFVWLVPQ-G-NQLKIVSTQNQDNPIMDSGKPVMGLDVWEHAFYLKYQNRKPEYVDAWWNVVNWDGIAKRYDEQVG

>U2QZH4_1321779_Fusobacteria_B_Anc

--FEQVKLPYAFDALEPNIDTKTMEIHYGKHHAAYTNNLNDALKNAPEFLEKPIEEILSNLDALPEAIRGAVRNNGGGFYNHNLYFTVMGPNAGGEPTGELAEKINEKFGSFEEFKVEFSKAAATRFGSGWAWLVVNKK-GELRVTSTANQDNPLMPEGTPILGIDVWEHAYYLNYQNRRPDYITAFFNVINWDAVAERYSKAK-

>U2YRD9_1261545_Halobacteria_A_Arc

TDYELPPLPYDYDALEPSISEQVLNWHHDTHHQGYVNGWNAAEETLEEAREEGDF---SGSP----GAIRNVTHNGSGHVLHTLFWESMGPNGGDEPSGALAARIQEDFGSYEAWKGEFEAAAS--AAGGWALLVYDTHSNQLRNLVVDKHDQGALWGAQPVLALDVWEHSYYYDYGPARGDFIDAFFDVVDWETPADRYEEAVA

>U5C1V6_1123057_Bacteroidetes_B_Mar

TGFEQTPLGYDYTSLEPNIDAMTMEIHYTKHAAAYAKNLGEAVSEEGD-ASKPLEDVLMNISKY----STKMRNNGGGHYNHELYWKIMKPNAGGKPEGVLAEAINSSFGSFEAFVEQFETAGKTRFGSGWAWLVLDKS-NKLAVGSTPNQDNPLMDQGIPLMGIDVWEHAYYLNYTSDRAGYISNWWNVINWDLVSERYEALV-

>U5C2N4_1123057_Bacteroidetes_B_Mar

MAFELPKLPYDFNALEPHIDARTMEIHHGKHHNAYVTNLNKAIEG-TDLADKSLEELMK-VAGAN----TPVRNNGGGHYNHSLFWTILSPKGGGLPSGDLAKAIDAKFGSFDAFKEEFNKAATTRFGSGWAWLGLDES-NELFVSSTPNQDNPLMDKGTPILGLDVWEHAYYLHYQNRRPDYISSFWNLVDWDAVSKRFTAAK-

>U5Q5M1_1400053_Bacteroidetes_B_Mar

NTLEFPQLPYSTGALEPFIDQQTVEIHYGKHQKAYFDNFLAAVKG-TDAEKLTILEVFKNISKYP----AAVKNNGGGFYNHVIYWESIKPN-SGKPSAKLAEAINKKFGSMDELKKQFTDAGKTRFGSGWAWLSVDDK-GELFVSSTANQESPLMDQGTPILAMDVWEHAYYLKYQNRRPDYIESFWQIINWDVVSGKYEAIVK

>U5QI99_1183438_Cyanobacteria_B_Ter

MAFTLPPLPYDESALAPYISAQTLSFHYGKHHKGYVDTLNKLVAGS-EAENTPLEELIKSVHG--QPDKAAIFNNAAQIWNHTFYWNSLKPGGGGEPTGTIAELIKDAFGSYDEFKKQFITAGTTQFGSGYAWLVKDKS-GKLSVIKTPNAETPLTDPTVPVLTFDVWEHAYYLDYQNLRPKYEEAVVHLLNWEFAEKNLASA--

>U6EAT7_1379702_Methanobacteria_A_Arc

KKYELPSLPYGYKDLEPYISEEQLRIHHDKHHQAYVDGANALLDKFDS-R-PGVE---FDVK----AVAKELSFHVGGFVLHKMFWENLAPAGGGEPTGTLAKYIEKDFGNLERFKEEFSQAAISTEGSGWAALTICRRTDRLFITQIEKHNVNVIPHFRVLMVLDVWEHAYYLDYKNVRPDYVAAFWNIVNWEEVNRRLEIELL

>U9VUJ7_1385935_Cyanobacteria_B_Ter

GEFTLPPLPYDYDALDDYIDSETMTIHHDRHHAGYVRNLNAAIANYPELQNDSLEELISKVHELPLDIRANVRKNGGGHANHTMFWEIMTPNGQGRPTGAIAAAIDDTFGDFEQFKQIFKSAGLSQFGSGWVWLTLTKN-GQLRITRTANQDSPLMEGNYPILGNDVWEHAYYLKYQNRRGDYLDAWWNVVNWDEVNQRLERAQA

>V4HNI7_1324957_Halobacteria_A_Arc

SEYELPPLPYDYDALEPHISEQVLTWHHDTHHQGYVNGWNSAEETLEQNREEGEF---GSSG----SALRNVTHNGSGHMLHTLFWQSMSPEGGDEPSGELADRIEEDFGSYDAWKGEFEAAAK--NASGWALLVYDSFSNELHNVVVDKHDQGALWGSHPVLALDVWEHSYYYDYGPARGDFIDAFFEVVDWTEPAERYEQATQ

>V5ADP9_1408164_Proteobacteria_B_Mar

MEHTLPALPYAIDALAPHYSQEAFEYHHGKHHNAYVVNLNNLQKGT-EFEAMDLESIVKQS-------AGGVYNNAAQVWNHSFFWNCMKPNGGAAPTGALADAINTKFGSYDGFKEAFVKSATGNFGSGWTWLVKKAD-GSVDIVNTGPAGTPLTTDDKALMTVDVWEHAYYIDYRNARPKFVEAFFKLVNWEFAEKNFA----

>V6DRS8_1173487_Halobacteria_A_Arc

MSYELDPLPYDYDALEPHISEQVLEWHHDTHHQGYVNGWNSAEETLEENRESHDF---SSSG----GAIRNVTHNSSGHILHDLFWQNMSPEGGDEPEGALADRIAEDFGSYEAWKGEFEAAAG--NASGWALLVYDTFSNQLRNVVVDKHDQGAVWGGHPILALDVWEHSYYHDYGPARGEFVDNFFEVVDWNEPSTRYEQAVE

>V6DS43_1173487_Halobacteria_A_Arc

MSYELDPLPYDYDALEPHISEQVLEWHHDTHHQGYVNGWNSAEETLEANRESHDF---SSSG----GAIRNVTHNSSGHILHDLFWQNMSPEGGDEPEGALADRIEEDFGSYEAWKGEFEAAAS--AAGGWALLVYDTFSNQLRNVVVDKHDQGAVWGGHPILALDVWEHSYYHDYGPARGEFVDNFFEVVDWEEPSSRYEQAVE

>V6IV34_1395513_Firmicutes_B_Ter

MPFVLPPLGFSYNALEPYIGRETMRIHYTKHHQAYVDNLNKAIDQHPEYRNWTLTELLTNLHQLPKDIRTAVRNNGGGHYNHSLFWEVLKPGGAKKPTGTLEKALNRELNGFEAFKVQFTNAATGQFGSGWAWLVLNPN-KKLEVISMNNQDNPIMVGKTPLFGLDVWEHAYYLDYQNRRPDYIKNSFNLYNWDVISRRYEWAMR

>V6J6E9_1395513_Firmicutes_B_Ter

SQFELPELEFAPDALEPYIDKETMTIHHDKHHQTYITNLNAALDKHPELKDHSLTDLIAHLEKVPEDIRTAVRNNGGGHYNHSLFWKVLIPGGSSQPTGALAKAIDEQLGGFEAFKEKFSAAAAGQFGSGWAWLTVNQL-NRLKVTNTPNQDSPLMDSETPLLGLDVWEHAYYLKYQNKRPEYIKNFFNVINWDYVSDVYDKVLA

>V9H8J0_641147_Proteobacteria_B_Mar

MAYTLPELGYAYNALEPHFDAQTLEIHHSKHHQTYVNNANGVRETLPEFQDLPVEEMMKRIGELPADKQMPARNNIGGHANHSFFWTLLKT--GTELKGSLKEAIERDFGSVDAFKEAFEKAAQTRFASGWAWLVVE-N-GKLAIVSTPNQDSPVMGSGTPLLTLDVWEHAYYLKFQNRRPEYIKTFWNVVNWDEVQRLYDSAN-

>W0ERB5_880074_Bacteroidetes_B_Mar

MIYQLPPLPYGTNDLAPVISQETIEYHYGKHEQTYLDNLNRLIDGT-PYAEKPLEEVIREA-------EGALFNNASQAWNHIFYFFTFAPDGRRKPQGKLAEAIDRQWGSLENFQKEFEQAGVTLFGSGWVWLSKDPE-GKLVISQESNAGSPLKKGYTPLLTFDVWEHAYYIDYRNRRAEHLHRLWEIVDWSVVEARYL----

>W0JR24_797299_Halobacteria_A_Arc

TDNELPPLPYDYDALEPSISEQVVTWHHDTHHQGYVNGLNSAEETLAENRESGDF---GSTP----GALSNVTHNGCGHYLHTLFWENMSPNGGGEPSGDLADRIEEDFGSYEAWKGEFEAAAG--AAGGWALLVYDPVAKQLRNIAVDKHDQGALWGSHPILALDVWEHSYYYDYGPDRGSFIDSFFDVVNWDKAAEEYQTCLD

>W0K3A4_751944_Halobacteria_A_Arc

SQHELPPLPYDYDALEPHISEQVLTWHHDTHHQGYVNGLNAAEGTLAENRESGEY---GSTA----GALGSVTHNGSGHYLHTMFWENMSENGGGEPSGELADRIEEDFGSYEGWKGEFKAAAS--AAGGWALLVYDPVSKQLRNLAVDKHDQGALWGSHPILALDVWEHSYYYDYGPKRGDFIDAFFEVVDWDDVAENYQKTVS

>W0K3U0_751944_Halobacteria_A_Arc

SNYELPPLPYDYDALEPHISEQVLTWHHDTHHQGYVSGWNSAEETLEANRESKDF---SDSA----GAIRNVTHNGSGHVLHTLFWQSMSPEGGDEPGGDLRDRIEADFGSYEGWKGEVEAAAS--AAGGWALLVYDSHSEQLRNVVVDKHDQGALWGSHPILALDVWEHSYYYDYGPARGDFVDAFFEVVDWEEPSARYEQAVD

>W0TLC1_1076588_Proteobacteria_B_Mar

TTHTLPVLPYAKDALQPHISVETLDYHHDKHHATYVDNLNNLIPGT-EFENASLEEIVMKA-------EGGIFNNGAQVWNHTFYFNCMSPNGGGEPGGALADAINSAFGSFADFKEKFSTSAATNFGCGWTWLVKNSD-GSLEIVNTSGAGNPMRDGQTPLLTIDVWEHAYYIDKRNVRPQYIADFFNVVNWDFVADNLG----

>W3TXZ6_1402976_Proteobacteria_B_Mar

MAFELAKLPYDYDALSPYMSRETLEYHHDKHHLAYLTNTNNFVKDL-GLENESLEHIVKKSFG----QNIGLFNNAAQYYNHNHFWHWMKKGGGGKLPEKLAKAIESDLGGYNKFRADFIAAAVAQFGSGWAWVAVK-D-GKLEIMKTSNSENPLVHDAQPILGVDVWEHSYYIDYRNARPKYLEAFVNLINWDYVLKLYEDCGF

>W3Y1G4_936589_Firmicutes_B_Ter

KPFELAPLPYAYDFLEPVIDKETMQIHHDKHHQAYVNNLNAAIAKYPDLPYGCVDCILGDIANVPEDIRQAVINNGGGHKNHTLFWEIMTKPDSSTLSGDLKDAIDRDLGGYDAFVEAFSNAAATRFGSGWAWLVVNKD-RKLEVTSTANQDNPLLEGKTGILGLDVWEHAYYLHYQNRRPDYIKEFFCVINWDKVSENYTRALN

>W6M2C8_1400863_Proteobacteria_B_Mar

MVHQLPELPYAKNALEPHISAETLEYHYGKHHQTYVTNLNNLIKGT-EFENLSLEDIVLKS-------SGGIFNNAAQVWNHTFYWNCLKPAGGGEPTGALAQAINQKFGSFAAFKEEFTKVTVGTFGSGWGWLVKNPD-GSVELLSTSNAGTPATAGKKALLTCDVWEHAYYIDYRNLRPKYVEAFWNLVNWDFVAHQYAG---

>W6M9I9_1400863_Proteobacteria_B_Mar

GPFTLPPLPYANDALSPVISANTIGFHYGKHHQGYATKLNELVAGS-PMADQSLEAIIKATAG--KADQAAIFNNAAQVWNHTFYWNSLKPKGGGKPTGALAEWIDKSFGDYDKFKAEFTKAATGQFGSGWAWLIKD-G-DKLAVTKTGNADTPIAHEQKPLLTVDVWEHAYYLDYQNRRADYVAAVLNLLNWEFAAKNLVG---

>A0A011MHB8_1454001_Proteobacteria_B_Mar

MEHKLPPLPFAMDALAPHMSQETLEYHYGKHHQAYVTNLNNLIKGT-EYEELDLEAIVRKAP------AGGIYNNSAQVWNHSFFWNCLRAGGGGAPGGALAAAIDARWGSFAEFAKAFQASAVGNFGSAWTWLVRKPD-GTVDIVNTGAAGTPLTTADKALLCIDVWEHAYYIDYRNLRPKFVETFLNLVNWHFAEQNYAA---

>A0A031LPG5_1160895_Thermoprotei_A_Arc

KKYELPPLPYNLDALEPYISKDIIDVHYNGHHKGYVNGANAFLDRVNKVLKGELASGQYDMM----GLMRGLVFNINGHKLHDLYWRNMTPSGGGKPGGSLADLIDKQYGSFEKFKQIFTEAANSLPGTGWTVLYYDVENGNLQIMTFENHFQNHIAELPILLILDEFEHAYYLQYKNKRADYVNNWWNVVNWDFADKKLQNYIK

>A0A062V7A0_1392998_Methanomicrobia_A_Mar

-------MAYELEDLLG-FSNQLLRNHF-TLYHGYVNNTNKLSDVLSTMVREGMA-GTPQYA----ELKRRFGWEWNGMRLHEYYFGNMIKGGRIDKNSDLYKKIVKDFGSYENWEKDFKAIGM-MRGIGWAMLYYDTAAGRLLNVWINEHDVGHLSGASPILIMDVFEHAFMIDYGVKKADYIESFFKAIDWKIDITMARNKLP

>A0A062V9F8_1392998_Methanomicrobia_A_Mar

-------MAYEAEHLLG-LSDKLLKNHF-TLYQGYVTNTNKLIDELGKLEKEGKA-GTPEYA----ELRRRFGWEWNGMRLHEYYFGKMTRGGKIDKNTNLYRKIVKDFGSYENWEKDFKATGM-MRGIGWAVLYYDITAGRLLNVWINEHDGGHLSGASPILIMDVFEHAFMIDYGTKRADYIEAFFKAIDWTIDPELDSALFG

>A0A080M9P3_1453999_Proteobacteria_B_Mar

MEHQLPPLPFAIDALAPHMSQETLEYHYGKHHQAYVTNLNNLIKGS-EYEALDLEAIVRKAP------AGGIYNNSAQVWNHTFFWNCLAPNGGGAPTGALAAAIDAKWGSFADFAKAFQASAVGNFGSGWTWLVRKAD-GSVDIVNMGAAGTPLTTGDQALLCVDVWEHAYYIDYRNLRPKFVETFLSLANWRFAEQNFVG---

>A0A081NMU1_1137799_Proteobacteria_B_Mar

MAFELPALPYARDALAPHISEETLNYHYGKHHKTYVDKLNGLVAGT-ELESKSLEEIIKTS-------EGGIFNNAAQIWNHTFYWDCLSPNGGGAPSGELADAINKAFGSFEEFVAAFNDKAVNNFGSSWTWLVKNSD-GSVEIVNTSNAGTPLTTDQKPLLTCDLWEHAYYIDYRNVRPEYLKNFWALVNWNFAAANYAK---

>A0A085HJD6_1005999_Proteobacteria_B_Mar

MSFELPALPYAKNALEPHISAETLEYHYGKHHNTYVVNLNNLVKGG-EFEGKSLEEIIMKS-------SGGIFNNAAQVWNHTFYWNCLAPNAGGEPTGALADAINKDFGSFAAFKEQFTDSAVKNFGSGWTWLVKKAD-GKLAIVNTSNAANPMTDGDKPLLTADVWEHAYYIDYRNARPKYMENFWALVNWAFVAKNLG----

>A0A097R2J1_1453496_Proteobacteria_B_Mar

MSFELPALPYEKNALEPHISAETLEYHYGKHHNTYVVNLNNLIKGT-EFEGKSLEEIIKTS-------TGGIFNNAAQVWNHTFYWHCLSPKGGNAPTGAVAEAINKAFGSFDAFKEELTKSAIGNFGSGWTWLVKKAD-GSLAIVNTSNAATPLTNGDKPLLTVDVWEHAYYIDYRNARPKYLENFWALVNWEFVAKNLA----

>A0A090CXU8_1437425_Chlamydiae_B_Mar

KKYELPKLPYELNALEPVISSQIMDLHYNKHHLAYVNNLNAALEKYEEAQNKGDL---DGMI----SAQGAIKFNGGGHINHSIFWTNLAPIGGGEPKGALLEAINKEFGSLDAFIQEFSAQTGAIQGSGWGWLGYNKKRKRLEIAVCPNQDPLVLQNLIPLLGIDVWEHAYYLQYKNVRPDYLKNIWKIINWKNVEERYAKAQS

>A0A090IF69_80854_Proteobacteria_B_Mar

MSITLPALPYAQDALEPHISAETLSFHYGKHHNTYVVKLNGLIEGT-PLAEKSLEEIVKSS-------EGPVFNNAAQVWNHTFYWNSLTPNAKGQPEGALADAINAKFGSFEAFQEAFNDKAVNNFGSSWTWLVKNAE-GELEIVNTSNAGTPITEGVTPLITVDLWEHAYYIDYRNLRPSYLKGFWALANWDFAAANFAA---

>A0A091FCF4_1499107_Proteobacteria_B_Mar

MAISLPELPYGKDALAPFITANTLDFHYGKHHKAYVDNLNKLIAGT-GLEQKSLEEIIKIAAK--DPAKAGIFNNAAQIWNHSFYWQCLKKAGGGVATGAIAAKINAVWGNYDKFAEELKNAGVTQFGSGWAWLVLE-G-DQLKITKTANADTPMAHGQKALLTIDVWEHAYYLDYQNRRPDYLAAVINLINWDFVNANLS----

>A0A094J7J3_1517416_Proteobacteria_B_Mar

MAFELPALPYEKNALEPHISAETLEYHYGKHHNAYVTKLNDAVKGT-DMESKSLEEIIKTS-------SGGVFNNAAQVWNHTFYWHCLSPNGGGEPTGALADAINSAFGSFDKFKEEFNAQAAGNFGSGWTWLVKKAD-GSVAIVNTSNAETPLTDSVTPILTVDVWEHAYYIDYRNARPNYLSAFWNLVNWEFVAKNFA----

>W7KP27_1326980_Thermoprotei_A_Arc

RKYELPPLPYKVDALEPYISKDIIDVHYNGHHKGYVNGANSFLDRLNKVIKGEVQAGQYDIQ----GILRGLVFNINGHKLHALYWENMAPNGGGKPGGALADLIDKQFGSFDKFKAVFTEAANSLPGTGWTVLYYDTESGNLEIMTFENHFQNHIAELPIILILDEFEHAYYLQYKNKRADYVNNWWNIVNWDWADKKLQKYLN

>X5MEY9_1458461_Proteobacteria_B_Mar

MTITLPDLPYAHDALEPHMSKTTFEFHHDKHHQKYVDTLNGLIDGT-NLEGKSLEDIIAAAKG--DDSKKKLFNQAAQVWNHTFFWNSMSPDGGGEPVGKLADAIKRGFGSFDDFKTQFSEAAAGEFGSGWAWLVSDKD-GKLSVMSTHDADLPLAHGAIALLTIDVWEHAYYLDYQNKRPDYISTYLKLVNWEFAAENYAAIS-
