# Supplementary material for: Characterization of ancestral Fe/Mn superoxide dismutases indicates their cambialistic origin
Source: Protein Sci. 2022 Sep 21;31(10):e4423. doi: 10.1002/pro.4423 (PMC9490801; doi:10.1002/pro.4423)
Supplement: Supplementary file 3 — Dataset S3 [file PRO-31-e4423-s006.zip › PRO_4423_Dataset3_PhylogeneticTree.docx]

((((O84296_272561_Chlamydiae_B_Mar:0.63262000,D6YU13_716544_Chlamydiae_B_Mar:0.45033000):0.11436000,(((A0A095XIK9_1230730_Firmicutes_B_Ter:0.29483000,A0A2P2EAB2_1445552_Proteobacteria_B_Mar:0.54056000):0.09543000,((A0A2U1E3U8_46507_Firmicutes_B_Ter:0.50701000,(((((A0A149W187_1789004_Proteobacteria_B_Mar:0.50798000,(A0A142LJU4_1690485_Proteobacteria_B_Mar:0.34897000,A0A2U2AEQ9_472582_Proteobacteria_B_Mar:0.21218000):0.07104000):0.05414000,(K1JSW8_742823_Proteobacteria_B_Mar:0.31294000,((R6A170_1262986_Proteobacteria_B_Mar:0.24857000,A0A1T4W9E9_1121442_Proteobacteria_B_Mar:0.94715000):0.16202000,((L9PNM3_1198452_Proteobacteria_B_Mar:0.10820000,((A0A0N0JCV7_1523424_Proteobacteria_B_Mar:0.04945000,(V5ADP9_1408164_Proteobacteria_B_Mar:0.13434000,A0A0U3DNG9_1768242_Proteobacteria_B_Mar:0.08110000):0.01484000):0.07616000,(((H0PZQ1_748247_Proteobacteria_B_Mar:0.07956000,A0A0K6IWK3_876478_Proteobacteria_B_Mar:0.15628000):0.03487000,(((A0A1G8BH66_83767_Proteobacteria_B_Mar:0.02161000,(A0A011QCI9_1454004_Proteobacteria_B_Mar:0.04103000,((A0A080M9P3_1453999_Proteobacteria_B_Mar:0.03811000,(A0A011NKF6_1454000_Proteobacteria_B_Mar:0.02378000,A0A011MHB8_1454001_Proteobacteria_B_Mar:0.04146000):0.06550000):0.01217000,(A0A1A8XY42_1860102_Proteobacteria_B_Mar:0.05260000,C7RIZ7_522306_Proteobacteria_B_Mar:0.02029000):0.04641000):0.01608000):0.05625000):0.04058000,A0A1R1I0D0_418702_Proteobacteria_B_Mar:0.06578000):0.02989000,A0A497X9T4_1381557_Proteobacteria_B_Mar:0.02161000):0.04406000):0.05192000,A0A0Q6BJ75_1736373_Proteobacteria_B_Mar:0.05353000):0.06407000):0.07587000):0.02643000,(((M1L4D0_1208918_Proteobacteria_B_Mar:0.23634000,M1M096_1208922_Proteobacteria_B_Mar:0.35566000):0.18204000,A0A2T0XJH1_323426_Proteobacteria_B_Mar:0.09083000):0.02812000,(A0A0N1LB93_1523428_Proteobacteria_B_Mar:0.11029000,A0A2G3K1V1_1559339_Proteobacteria_B_Mar:0.25064000):0.06372000):0.02874000):0.03995000):0.02174000):0.05206000):0.04997000,(((A0A1V3PIV2_1945854_Proteobacteria_B_Mar:0.19136000,C7RD31_523791_Proteobacteria_B_Mar:0.15992000):0.07195000,((((((A0A081NMU1_1137799_Proteobacteria_B_Mar:0.17033000,A0A162GML5_1822219_Proteobacteria_B_Mar:0.10901000):0.04569000,(A4BGS7_314283_Proteobacteria_B_Mar:0.11947000,A0A1H6CZE7_568106_Proteobacteria_B_Mar:0.11937000):0.02235000):0.05620000,(A0A1H4F8V2_152573_Proteobacteria_B_Mar:0.12334000,(A0A2N0WW65_2058089_Proteobacteria_B_Mar:0.17198000,(A0A0S2JHC6_58049_Proteobacteria_B_Mar:0.14684000,A0A090IF69_80854_Proteobacteria_B_Mar:0.13559000):0.03297000):0.03743000):0.03855000):0.03452000,((((A0A1J4QJC3_1414654_Proteobacteria_B_Mar:0.12887000,(E1VHB8_83406_Proteobacteria_B_Mar:0.15655000,K2JEV8_745411_Proteobacteria_B_Mar:0.09286000):0.03554000):0.03534000,(A0A2S1JPT9_359370_Proteobacteria_B_Mar:0.12095000,(A0A4Q5V9J9_1913989_Proteobacteria_B_Mar:0.09195000,A0A1Y0FVA0_1987723_Proteobacteria_B_Mar:0.06072000):0.06321000):0.03953000):0.02632000,(A8H3Z8_398579_Proteobacteria_B_Mar:0.12264000,E1SRJ0_550540_Proteobacteria_B_Mar:0.08999000):0.03427000):0.03486000,(A0A1E2V9J2_197479_Proteobacteria_B_Mar:0.17202000,(A0A094J7J3_1517416_Proteobacteria_B_Mar:0.09857000,A0A2A5LZ91_2039467_Proteobacteria_B_Mar:0.12963000):0.03109000):0.03645000):0.03850000):0.02861000,(I2JH08_1168065_Proteobacteria_B_Mar:0.15745000,(A0A1R3VN56_233100_Proteobacteria_B_Mar:0.23300000,B5JWC0_391615_Proteobacteria_B_Mar:0.14040000):0.09851000):0.05939000):0.02224000,A0A0B4XLG0_391936_Proteobacteria_B_Mar:0.20514000):0.02334000):0.03166000,(((((A0A1E2ZEY7_1655433_Proteobacteria_B_Mar:0.02996000,(G2FFW9_1049564_Proteobacteria_B_Mar:0.07761000,A0A1E2V0Q9_1818881_Proteobacteria_B_Mar:0.01865000):0.01578000):0.13756000,(A0A0F7K213_1543721_Proteobacteria_B_Mar:0.14618000,A0A1T2KSF0_1918948_Proteobacteria_B_Mar:0.10762000):0.03476000):0.03399000,(A0A251X438_1570016_Proteobacteria_B_Mar:0.12317000,(D0LUJ8_502025_Proteobacteria_B_Mar:0.37393000,E1X0Y3_862908_Proteobacteria_B_Mar:0.27286000):0.06079000):0.08903000):0.03907000,(W0TLC1_1076588_Proteobacteria_B_Mar:0.17311000,(A0A1Z4VNQ5_585455_Proteobacteria_B_Mar:0.12731000,(A0A1T2L771_1918949_Proteobacteria_B_Mar:0.03774000,A0A0B0HBS3_2340_Proteobacteria_B_Mar:0.00656000):0.23591000):0.07605000):0.08197000):0.03807000,((((A0A0A6PC03_1003181_Proteobacteria_B_Mar:0.13288000,(A0A0D0S5X1_1199154_Proteobacteria_B_Mar:0.19381000,A0A2P1PVM9_2021234_Proteobacteria_B_Mar:0.11498000):0.08250000):0.03604000,A0A1B1YPZ1_1810504_Proteobacteria_B_Mar:0.19259000):0.03673000,(((A0A0F6YK52_927083_Proteobacteria_B_Mar:0.32518000,A0A4Q6B980_1977087_Proteobacteria_B_Mar:0.21962000):0.06763000,(A0A0K0XVJ8_1579979_Proteobacteria_B_Mar:0.13638000,(A0A1Y2K534_1434232_Proteobacteria_B_Mar:0.19897000,A0A1Y6CDA5_1513793_Proteobacteria_B_Mar:0.20737000):0.03173000):0.07530000):0.04777000,(A0A2K8LAI0_1921087_Proteobacteria_B_Mar:0.23608000,A0A0W0TNM8_453_Proteobacteria_B_Mar:0.18613000):0.04771000):0.04404000):0.03095000,(A0A1Z5HC40_113268_Proteobacteria_B_Mar:0.15511000,A0A139SPB8_1680762_Proteobacteria_B_Mar:0.22402000):0.03567000):0.03546000):0.01381000):0.01308000):0.09330000,(((A0A2X0WVT3_179995_Proteobacteria_B_Mar:0.67674000,((W0ERB5_880074_Bacteroidetes_B_Mar:0.44709000,((A0A1I2LRE0_1855325_Bacteroidetes_B_Mar:0.23819000,S8FFI4_888054_Bacteroidetes_B_Mar:0.23520000):0.14950000,(R5JCK6_1262737_Bacteroidetes_B_Mar:0.14303000,(A0A2V3PRT9_1605892_Bacteroidetes_B_Mar:0.21513000,(A0A0F5IRT0_1203610_Bacteroidetes_B_Mar:0.06626000,D7JFA1_575590_Bacteroidetes_B_Mar:0.30851000):0.05747000):0.21339000):0.09227000):0.13331000):0.12691000,A0A1Y4C9N7_1965623_Bacteroidetes_B_Mar:0.42103000):0.14804000):0.13178000,I2K993_1165841_Proteobacteria_B_Mar:0.56251000):0.13613000,((A0A2D3WM99_2015906_Proteobacteria_B_Mar:0.44933000,A8PPX3_59196_Proteobacteria_B_Mar:0.32587000):0.17249000,(A0A1V0RDW6_28898_Proteobacteria_B_Mar:0.61301000,A0A2Z2L2V6_549298_Proteobacteria_B_Mar:0.29194000):0.06362000):0.04355000):0.07763000):0.04524000,(((((A0A0S2KFC6_1249552_Proteobacteria_B_Mar:0.05632000,A0A1E8CFB9_1524254_Proteobacteria_B_Mar:0.05480000):0.35214000,(E0TI36_314260_Proteobacteria_B_Mar:0.43764000,(A0A4Q3S5H0_1978230_Proteobacteria_B_Mar:0.59579000,A0A193LCR6_1548547_Proteobacteria_B_Mar:0.53241000):0.12686000):0.04937000):0.08888000,Q3YRT4_269484_Proteobacteria_B_Mar:0.61214000):0.06010000,((((((A0A0M2R958_1549748_Proteobacteria_B_Mar:0.11458000,(A0A4R2PEU5_1188247_Proteobacteria_B_Mar:0.40054000,(G2KRC6_856793_Proteobacteria_B_Mar:0.36599000,((A0A1I4SFU3_1166257_Proteobacteria_B_Mar:0.19541000,F2IV67_991905_Proteobacteria_B_Mar:0.08842000):0.08729000,(A0A231UUB0_1876515_Proteobacteria_B_Mar:0.37643000,(A0A1E3VXW9_1774968_Proteobacteria_B_Mar:0.26888000,((A0A256FXP0_571255_Proteobacteria_B_Mar:0.07668000,(H0TES2_551947_Proteobacteria_B_Mar:0.13497000,A0A4R2GX74_659006_Proteobacteria_B_Mar:0.22766000):0.10831000):0.05565000,(A0A090FD11_1505946_Proteobacteria_B_Mar:0.14274000,W3TXZ6_1402976_Proteobacteria_B_Mar:0.28466000):0.03295000):0.02525000):0.02501000):0.02563000):0.08067000):0.11551000):0.07667000):0.04212000,(A8U258_331869_Proteobacteria_B_Mar:0.18774000,(D5BTR8_488538_Proteobacteria_B_Mar:0.12282000,(Q0F8L9_367336_Proteobacteria_B_Mar:0.13355000,G6A1M0_909943_Proteobacteria_B_Mar:0.12047000):0.05686000):0.37220000):0.03960000):0.07213000,(A0A4R7K0K2_332522_Proteobacteria_B_Mar:0.48586000,D0RR50_684719_Proteobacteria_B_Mar:0.25288000):0.08334000):0.03970000,A0A366ENT2_1473586_Proteobacteria_B_Mar:0.34237000):0.06038000,(R5L6H9_1262760_Spirochaetes_B_Mar:0.55215000,A0A061Q889_1492281_Proteobacteria_B_Mar:0.35731000):0.14002000):0.20796000,(((A0A3M0CGB4_911205_Proteobacteria_B_Mar:0.23559000,(((A0A1K1LIX2_1855339_Proteobacteria_B_Mar:0.11096000,A0A4R8ITH7_381308_Proteobacteria_B_Mar:0.36938000):0.18258000,(A0A257EMF7_2015572_Proteobacteria_B_Mar:0.61975000,B3EJL9_331678_Chlorobi_B_Mar:0.27252000):0.13546000):0.07928000,(U5QI99_1183438_Cyanobacteria_B_Ter:0.31368000,A0A4Q5XXF3_1913988_Proteobacteria_B_Mar:0.25963000):0.10723000):0.05421000):0.06355000,((A0YJL0_313612_Cyanobacteria_B_Ter:0.16716000,(A0A1C0VXQ7_1880991_Cyanobacteria_B_Ter:0.17750000,A0A073CMZ0_388467_Cyanobacteria_B_Ter:0.09117000):0.05634000):0.07974000,(K9SBX7_1173025_Cyanobacteria_B_Ter:0.26637000,((P77968_1111708_Cyanobacteria_B_Ter:0.05017000,(A0A0M2Q238_317619_Cyanobacteria_B_Ter:0.11186000,B0JGF5_449447_Cyanobacteria_B_Ter:0.08105000):0.04485000):0.03115000,(L8LWB9_102125_Cyanobacteria_B_Ter:0.11728000,K9Z4N7_755178_Cyanobacteria_B_Ter:0.13285000):0.07776000):0.14269000):0.04473000):0.03877000):0.06327000,((((A0A0H4WNU9_1297742_Proteobacteria_B_Mar:0.26713000,A0A4P8L1J4_980445_Proteobacteria_B_Mar:0.38847000):0.03410000,(A0A091FCF4_1499107_Proteobacteria_B_Mar:0.22278000,A0A0M4CZS5_1603606_Proteobacteria_B_Mar:0.32692000):0.12483000):0.04462000,(X5MEY9_1458461_Proteobacteria_B_Mar:0.43137000,C8Q0X7_553217_Proteobacteria_B_Mar:0.28942000):0.10031000):0.02417000,((A0A077FIP1_1528098_Proteobacteria_B_Mar:0.19950000,A0A0C1QWB0_86105_Proteobacteria_B_Mar:0.34124000):0.14853000,(A0A3N1MAT8_94_Proteobacteria_B_Mar:0.20779000,K9VXC7_1173022_Cyanobacteria_B_Ter:0.15207000):0.06509000):0.04917000):0.02046000):0.03342000):0.03748000):0.07550000,Q9ZD15_272947_Proteobacteria_B_Mar:0.73520000):0.05193000):0.26744000):0.19281000,(R7QTT1_1262942_Firmicutes_B_Ter:0.71159000,(R6EU58_1262994_Firmicutes_B_Ter:0.78529000,(R6GYH2_1262911_Firmicutes_B_Ter:1.02064000,R5HFY3_1263001_Firmicutes_B_Ter:0.37052000):0.06253000):0.06918000):0.31247000):0.06978000):0.05514000,(((A0A1I1DPZ5_34097_Spirochaetes_B_Mar:0.37566000,Q6YQD2_262768_Tenericutes_B_Ter:0.53660000):0.04300000,A0A1B4XH23_1620215_Proteobacteria_B_Mar:0.70521000):0.03896000,((A0A1G6AIZ5_439219_Firmicutes_B_Ter:0.35887000,W3Y1G4_936589_Firmicutes_B_Ter:0.42611000):0.07191000,((A0A4Q2ZMN6_2044944_Bacteroidetes_B_Mar:0.29275000,(Q9RUV2_243230_Deinococcus-Thermus_B_Ter:0.17916000,((A0A242PAB3_1970738_Proteobacteria_B_Mar:0.23436000,V9H8J0_641147_Proteobacteria_B_Mar:0.27190000):0.04682000,(((Q1LSZ4_374463_Proteobacteria_B_Mar:0.43912000,((A0A085JMH6_1005995_Proteobacteria_B_Mar:0.03046000,A0A2I0FW47_2025587_Proteobacteria_B_Mar:0.11151000):0.03732000,A0A0J8VRJ1_435910_Proteobacteria_B_Mar:0.02485000):0.04672000):0.01624000,(A0A085G4I0_910964_Proteobacteria_B_Mar:0.04868000,(G7LTJ2_598467_Proteobacteria_B_Mar:0.05266000,A0A1I0EA93_1123402_Proteobacteria_B_Mar:0.12860000):0.02679000):0.01256000):0.03700000,(D5VB54_1236608_Proteobacteria_B_Mar:0.19535000,A0A1V3TPU9_1924934_Proteobacteria_B_Mar:0.19078000):0.08563000):0.05299000):0.10463000):0.08198000):0.09077000,(((((((Q2S1T9_309807_Bacteroidetes_B_Mar:0.40129000,A0A259TZ62_716817_Rhodothermaeota_B_Mar:0.29329000):0.05341000,A0A1B6B8G0_1048380_Firmicutes_B_Ter:0.42084000):0.05725000,((A0A4R1R4E5_1469948_Firmicutes_B_Ter:0.41495000,E1QXA5_633147_Actinobacteria_B_Ter:0.28666000):0.22774000,U2QZH4_1321779_Fusobacteria_B_Anc:0.18684000):0.13575000):0.02275000,(A0A1H3A4M8_1123352_Firmicutes_B_Ter:0.34101000,A0A0C7NIR4_1006576_Thermotogae_B_Anc:0.36937000):0.14719000):0.02372000,(((I6ZUA8_1191523_Ignavibacteriae_B_Mar:0.21031000,I0AK38_945713_Ignavibacteriae_B_Mar:0.21395000):0.11011000,((A0A380N0R1_13276_Proteobacteria_B_Mar:0.38884000,(U5Q5M1_1400053_Bacteroidetes_B_Mar:0.57415000,A0A2Z2P4N8_1192854_Proteobacteria_B_Mar:0.32398000):0.06802000):0.10695000,(A0A3T0EBQ7_1434191_Proteobacteria_B_Mar:0.32942000,A0A0X3T641_1685380_Proteobacteria_B_Mar:0.27428000):0.06791000):0.08855000):0.01601000,(A0A191ZFD3_1860122_Proteobacteria_B_Mar:0.31527000,(A0A085L1P1_1453500_Bacteroidetes_B_Mar:0.23906000,(A0A1I5ZIY7_1227077_Bacteroidetes_B_Mar:0.23978000,(((A0A098SDX5_1524460_Bacteroidetes_B_Mar:0.23282000,(A0A3E1EU86_1737063_Bacteroidetes_B_Mar:0.09056000,(C0BHJ2_487796_Bacteroidetes_B_Mar:0.24802000,(Q26GK1_156586_Bacteroidetes_B_Mar:0.06898000,A6EQH5_50743_Bacteroidetes_B_Mar:0.21203000):0.05871000):0.09774000):0.03852000):0.03416000,(U2EL32_1033802_Proteobacteria_B_Mar:0.53756000,(B3ER22_452471_Bacteroidetes_B_Mar:0.33472000,D0J9E3_600809_Bacteroidetes_B_Mar:0.60371000):0.11178000):0.05118000):0.03055000,(G8R729_926562_Bacteroidetes_B_Mar:0.19475000,A0A0D3LFT2_1257021_Bacteroidetes_B_Mar:0.24014000):0.06462000):0.05921000):0.02879000):0.03216000):0.04010000):0.08826000):0.02461000,(((A0A142X6W1_1632864_Planctomycetes_B_Mar:0.28341000,(E8QXM5_575540_Planctomycetes_B_Mer:0.31226000,(A0A086D0E9_1492922_Proteobacteria_B_Mar:0.13599000,A0A2N7UR03_1684789_Proteobacteria_B_Mar:0.13658000):0.16268000):0.06766000):0.04066000,A0A212PXD1_877466_Chloroflexi_B_Ter:0.30453000):0.05987000,((((((A0A328VKE5_1825093_Chloroflexi_B_Ter:0.19419000,A0A401ZTK7_2014871_Chloroflexi_B_Ter:0.20480000):0.10735000,((U9VUJ7_1385935_Cyanobacteria_B_Ter:0.31875000,A0A2W1JHI3_1764569_Cyanobacteria_B_Ter:0.21157000):0.10867000,G8NWQ2_682795_Acidobacteria_B_Mar:0.37551000):0.04912000):0.09950000,((A0A1M6L352_1830138_Firmicutes_B_Ter:0.20572000,A0A418MLK8_2048547_Firmicutes_B_Ter:0.17767000):0.08041000,(A0A0K2SFV8_1555112_Firmicutes_B_Ter:0.25217000,Q1ARZ8_266117_Actinobacteria_B_Ter:0.34872000):0.04886000):0.03149000):0.04586000,E6SLN6_644966_Firmicutes_B_Ter:0.18525000):0.01581000,(I4EEM3_1129897_Chloroflexi_B_Ter:0.28230000,(A0A399EPG5_2026184_Deinococcus-Thermus_B_Ter:0.25103000,A5UY95_357808_Chloroflexi_B_Ter:0.22705000):0.07245000):0.07199000):0.07906000,(A0A0M9UDM4_872965_Chloroflexi_B_Ter:0.16815000,(I0I4H8_926550_Chloroflexi_B_Ter:0.24875000,A0A192WU48_1660251_Acidobacteria_B_Mar:0.39286000):0.06225000):0.02736000):0.03322000):0.02269000):0.05038000,((A0A031IK26_1470592_Firmicutes_B_Ter:0.22195000,A0A4R6BX88_198484_Firmicutes_B_Ter:0.16299000):0.07180000,((A0A099W947_1552123_Firmicutes_B_Ter:0.31220000,(A0A2P8H4Q1_1176648_Firmicutes_B_Ter:0.11310000,A0A0K0GDK0_1637974_Firmicutes_B_Ter:0.12662000):0.10359000):0.02624000,((L8XU36_1261130_Proteobacteria_B_Mar:0.24542000,(S0L434_1140003_Firmicutes_B_Ter:0.23486000,A0A0R2HP56_1449336_Firmicutes_B_Ter:0.14292000):0.02556000):0.04065000,((A0A143PHC2_1855912_Acidobacteria_B_Mar:0.21438000,Q02A56_234267_Acidobacteria_B_Mar:0.32084000):0.07029000,(D3FDM4_469383_Actinobacteria_B_Ter:0.33137000,(A0A2A6RIS4_2024553_Chloroflexi_B_Ter:0.18719000,(A0A2H3KUE0_1506545_Chloroflexi_B_Ter:0.17754000,A9WJC9_324602_Chloroflexi_B_Ter:0.09092000):0.05006000):0.12183000):0.04735000):0.06193000):0.05924000):0.02351000):0.01931000):0.01030000):0.06566000):0.02666000):0.02311000):0.40190000):0.19461000,((A0A0M5KZK2_1528099_Actinobacteria_B_Ter:0.41088000,A0A2A9DLZ4_1724_Actinobacteria_B_Ter:0.23455000):0.05625000,((((A0A2T0GV44_33906_Actinobacteria_B_Ter:0.11266000,A0A1I1CI47_490629_Actinobacteria_B_Ter:0.21202000):0.16546000,(((A0A166QH71_683316_Actinobacteria_B_Ter:0.12838000,(A0A1V2RP88_1857892_Actinobacteria_B_Ter:0.06237000,(A0A0N1G8F9_1592327_Actinobacteria_B_Ter:0.06722000,A0A0N1H7L0_1592329_Actinobacteria_B_Ter:0.06875000):0.05802000):0.24592000):0.08377000,(A0A3D9SUE6_111806_Actinobacteria_B_Ter:0.08791000,(A0A2T0ZW47_1629062_Actinobacteria_B_Ter:0.18829000,D3PZ20_446470_Actinobacteria_B_Ter:0.08041000):0.07419000):0.15582000):0.06675000,K6W4W1_1184607_Actinobacteria_B_Ter:0.20721000):0.03814000):0.12254000,(((((A0A386UD88_1389713_Actinobacteria_B_Ter:0.10153000,A0A378YCD3_1823_Actinobacteria_B_Ter:0.05728000):0.12403000,((A0A166PIY3_37915_Actinobacteria_B_Ter:0.07898000,D5UQ12_521096_Actinobacteria_B_Ter:0.06950000):0.09117000,(K6WAV5_1108045_Actinobacteria_B_Ter:0.08715000,A0A0Q5R2U1_1736349_Actinobacteria_B_Ter:0.05869000):0.08831000):0.09496000):0.06091000,((A0A1H6UCM7_1043493_Actinobacteria_B_Ter:0.17460000,(((A0A1I3W540_1855324_Actinobacteria_B_Ter:0.09507000,((A0A168EFF5_1300344_Actinobacteria_B_Ter:0.04240000,A0A3N5AAM9_154117_Actinobacteria_B_Ter:0.14730000):0.10190000,(A0A2A9CZS9_556530_Actinobacteria_B_Ter:0.21278000,(A0A1G6RCD0_1814289_Actinobacteria_B_Ter:0.06990000,C7R4P3_471856_Actinobacteria_B_Ter:0.06522000):0.08852000):0.09017000):0.07637000):0.05548000,C0W267_525245_Actinobacteria_B_Ter:0.19881000):0.04944000,A0A1I5SFS4_1523247_Actinobacteria_B_Ter:0.22209000):0.03867000):0.03682000,A0A1H0IML9_1090615_Actinobacteria_B_Ter:0.24959000):0.06776000):0.00448000,(((A0A1H8D259_1424661_Actinobacteria_B_Ter:0.10274000,A4AJQ1_312284_Actinobacteria_B_Ter:0.11479000):0.16369000,(A0A0H0ZNJ8_1652545_Actinobacteria_B_Ter:0.10460000,A0A239QSP2_1945888_Actinobacteria_B_Ter:0.22925000):0.08149000):0.04558000,(A0A4R7JC83_993414_Actinobacteria_B_Ter:0.31459000,D4YMB9_585530_Actinobacteria_B_Ter:0.13537000):0.05385000):0.04104000):0.03591000,((A0A2T0PUC9_1144618_Actinobacteria_B_Ter:0.12364000,A0A329QP63_1981511_Actinobacteria_B_Ter:0.18362000):0.06355000,A0A223S566_1235441_Actinobacteria_B_Ter:0.12397000):0.05654000):0.07471000):0.10103000,Q83GI4_203267_Actinobacteria_B_Ter:0.50513000):0.08593000):0.13374000):0.01849000,((((((U2YRD9_1261545_Halobacteria_A_Arc:0.15712000,((M0DHS9_1227487_Halobacteria_A_Arc:0.03263000,((((A0A368N5W7_1126245_Halobacteria_A_Arc:0.04432000,A0A345E419_1547899_Halobacteria_A_Arc:0.01951000):0.01881000,(E4NT27_469382_Halobacteria_A_Arc:0.04329000,A0A1H3W6N9_555874_Halobacteria_A_Arc:0.01133000):0.01144000):0.01503000,((A0A1H3ILX3_660517_Halobacteria_A_Arc:0.00561000,(U1QK35_1070774_Halobacteria_A_Arc:0.09011000,Q18HG3_362976_Halobacteria_A_Arc:0.09997000):0.04235000):0.03722000,A0A1H6AL08_699433_Halobacteria_A_Arc:0.01050000):0.02499000):0.01752000,(A0A256IHQ1_1383851_Halobacteria_A_Arc:0.00644000,A0A238VTU4_63740_Halobacteria_A_Arc:0.04765000):0.03596000):0.05724000):0.05268000,V4HNI7_1324957_Halobacteria_A_Arc:0.05584000):0.05552000):0.04075000,((A0A1H6J821_1267564_Halobacteria_A_Arc:0.09841000,(A0A1D8S4L0_1873524_Halobacteria_A_Arc:0.21897000,(A0A2R4X0R1_1679096_Halobacteria_A_Arc:0.18623000,(((Q03302_272569_Halobacteria_A_Arc:0.05328000,(A0A4D6HF93_1457250_Halobacteria_A_Arc:0.04219000,(A0A1Q1FM06_1932360_Halobacteria_A_Arc:0.03587000,(M1XSP2_268739_Halobacteria_A_Arc:0.04366000,A0A1U7EY38_348780_Halobacteria_A_Arc:0.02892000):0.02236000):0.05162000):0.02802000):0.01557000,(((D8J2G3_795797_Halobacteria_A_Arc:0.08645000,(A0A202E7D5_253108_Halobacteria_A_Arc:0.06448000,(((A0A3N6LM69_1679091_Halobacteria_A_Arc:0.01638000,(A0A3N6M5P3_1679083_Halobacteria_A_Arc:0.00689000,(D3SY04_547559_Halobacteria_A_Arc:0.01430000,(A0A2Z2HUJ4_745377_Halobacteria_A_Arc:0.02193000,L0AKN7_797304_Halobacteria_A_Arc:0.07633000):0.01936000):0.02148000):0.01547000):0.00637000,(M0L696_358396_Halobacteria_A_Arc:0.02344000,((A0A063ZJ32_1495067_Halobacteria_A_Arc:0.04844000,W0JR24_797299_Halobacteria_A_Arc:0.00604000):0.02494000,(M0CBS2_1230457_Halobacteria_A_Arc:0.04280000,(A0A1H9BZT2_1186196_Halobacteria_A_Arc:0.00517000,(L0JLZ0_797303_Halobacteria_A_Arc:0.02729000,(L9ZI72_1227494_Halobacteria_A_Arc:0.02315000,A0A1I0M6P7_1202768_Halobacteria_A_Arc:0.02723000):0.00581000):0.00656000):0.03007000):0.02843000):0.00055000):0.02225000):0.01088000,(L0IC00_797302_Halobacteria_A_Arc:0.04423000,(L9VRX1_1114856_Halobacteria_A_Arc:0.01724000,L9WFH7_1230460_Halobacteria_A_Arc:0.02708000):0.00538000):0.00055000):0.01712000):0.05512000):0.02744000,F7PIA8_1033806_Halobacteria_A_Arc:0.09942000):0.02128000,(M0M2F6_1132509_Halobacteria_A_Arc:0.06549000,(M0MM71_1227455_Halobacteria_A_Arc:0.04743000,M0MDJ5_931277_Halobacteria_A_Arc:0.02780000):0.01003000):0.07040000):0.04036000):0.01931000,(A0A1G8W4P7_890420_Halobacteria_A_Arc:0.05995000,(U1QN78_1085028_Halobacteria_A_Arc:0.07948000,U1MGY5_1325472_Halobacteria_A_Arc:0.12718000):0.13035000):0.04732000):0.02365000):0.02298000):0.02574000):0.04755000,A0A0F7PDN8_1604004_Halobacteria_A_Arc:0.13714000):0.05355000):0.32348000,((K0AYP0_1128398_Firmicutes_B_Ter:1.23865000,((C1DW98_204536_Aquificae_B_Anc:0.22323000,B4U6J9_380749_Aquificae_B_Anc:0.18866000):0.30671000,((A0A218ZVX8_1961136_Thermoplasmata_A_Arc:0.07628000,(T0MPA5_667137_Thermoplasmata_A_Arc:0.13022000,((Q9HM56_273075_Thermoplasmata_A_Arc:0.19940000,(Q6L1T7_263820_Thermoplasmata_A_Arc:0.00748000,(S0AS08_333146_Thermoplasmata_A_Arc:0.08319000,A0A0N8VL92_312540_Thermoplasmata_A_Arc:0.03783000):0.05325000):0.15985000):0.12207000,T0M4N9_667135_Thermoplasmata_A_Arc:0.10835000):0.01766000):0.06423000):0.06928000,T0MZU0_261391_Thermoplasmata_A_Arc:0.11141000):0.16644000):0.17483000):0.19766000,(A0A1L4CZX9_1915309_Proteobacteria_B_Mar:0.77318000,(A7HBL1_404589_Proteobacteria_B_Mar:0.57663000,((D8JZF0_552811_Chloroflexi_B_Ter:0.29460000,(A0A0W0GJW2_1217799_Chloroflexi_B_Ter:0.19523000,A0A1P8F872_1839801_Chloroflexi_B_Ter:0.16165000):0.06589000):0.09253000,(A0A0C9Q165_1197129_Planctomycetes_B_Mar:0.23440000,Q3Z7W8_243164_Chloroflexi_B_Ter:0.34562000):0.06478000):0.29905000):0.52673000):0.18880000):0.73340000):0.23365000,(A0A2Z4FGN2_1548548_Proteobacteria_B_Mar:0.39492000,(Q0AW95_335541_Firmicutes_B_Ter:0.32463000,(A0A259UH52_1123289_Firmicutes_B_Ter:0.34914000,A0A0B7MKY0_499207_Firmicutes_B_Ter:0.25079000):0.31294000):0.20652000):0.22261000):0.08732000,(((A8MAM7_397948_Thermoprotei_A_Arc:0.07381000,E1QRN4_572478_Thermoprotei_A_Arc:0.10387000):0.24623000,(Q08713_330779_Thermoprotei_A_Arc:0.07400000,((A0A348B0W0_1670455_Thermoprotei_A_Arc:0.12995000,((((A4YHX7_399549_Thermoprotei_A_Arc:0.02753000,H2C9I7_671065_Thermoprotei_A_Arc:0.03677000):0.09688000,W7KP27_1326980_Thermoprotei_A_Arc:0.04656000):0.01311000,((A0A2U9IFV2_41673_Thermoprotei_A_Arc:0.05325000,(A0A031LPG5_1160895_Thermoprotei_A_Arc:0.08128000,F4B3P9_933801_Thermoprotei_A_Arc:0.06743000):0.06445000):0.04668000,Q96Y84_273063_Thermoprotei_A_Arc:0.03247000):0.02723000):0.03292000,P80857_273057_Thermoprotei_A_Arc:0.06543000):0.02085000):0.01438000,T0LM88_667138_Thermoplasmata_A_Arc:0.32906000):0.02898000):0.08441000):0.41629000,(((O93724_178306_Thermoprotei_A_Arc:0.01729000,(G7VBY8_1104324_Thermoprotei_A_Arc:0.03203000,A1RW25_384616_Thermoprotei_A_Arc:0.04855000):0.02142000):0.10940000,(Q9Y8H8_272557_Thermoprotei_A_Arc:0.15654000,(L0AA53_1056495_Thermoprotei_A_Arc:0.12463000,D9Q0R7_666510_Thermoprotei_A_Arc:0.13732000):0.24169000):0.11700000):0.11338000,(F2KQT8_693661_Archaeoglobi_A_Arc:0.25184000,(((A0A498GYD6_1550565_Methanomicrobia_A_Mar:0.21151000,(I7KYD2_1201294_Methanomicrobia_A_Arc:0.00468000,A0A0X3BJM4_86622_Methanomicrobia_A_Mar:0.00598000):0.10753000):0.13802000,(((A0B701_349307_Methanomicrobia_A_Arc:0.22170000,(G7WL45_1110509_Methanomicrobia_A_Arc:0.10339000,F4BZF7_990316_Methanomicrobia_A_Arc:0.08599000):0.11131000):0.05242000,((F7XLC9_679901_Methanomicrobia_A_Arc:0.22873000,((((A0A0E3NRG6_1434102_Methanomicrobia_A_Mar:0.04512000,Q8TQG9_188937_Methanomicrobia_A_Arc:0.06084000):0.01944000,A0A0E3WWB9_1434107_Methanomicrobia_A_Mar:0.11831000):0.04411000,(K4MC62_1094980_Methanomicrobia_A_Arc:0.09708000,L0KZ58_867904_Methanomicrobia_A_Arc:0.10324000):0.01267000):0.02065000,A0A0E3NWV4_1434100_Methanomicrobia_A_Mar:0.09690000):0.12605000):0.26910000,(P18868_187420_Methanobacteria_A_Arc:0.19057000,(U6EAT7_1379702_Methanobacteria_A_Arc:0.16812000,(A0A1D2W9T7_1860100_Methanobacteria_A_Arc:0.27730000,(A0A1D3L2G7_118062_Methanobacteria_A_Arc:0.13210000,F0TCH4_877455_Methanobacteria_A_Arc:0.14356000):0.06092000):0.03237000):0.06650000):0.15593000):0.08141000):0.04659000,A0A0Q4BCN0_1713724_Thermoplasmata_A_Arc:0.56380000):0.06231000):0.02558000,Q2FSC2_323259_Methanomicrobia_A_Arc:0.35117000):0.16813000):0.14061000):0.07168000):0.32674000):0.01941000,A0A1I2QXX4_553467_Halobacteria_A_Arc:0.33895000):0.16076000);
