# Supplementary material for: Characterization of ancestral Fe/Mn superoxide dismutases indicates their cambialistic origin
Source: Protein Sci. 2022 Sep 21;31(10):e4423. doi: 10.1002/pro.4423 (PMC9490801; doi:10.1002/pro.4423)
Supplement: Supplementary file 3 — Dataset S3 [file PRO-31-e4423-s006.zip › PRO_4423_Dataset3_SequenceAlignment.docx]

>A0A0A6PC03_1003181_Proteobacteria_B_Mar

M-HTLPELPYAKNALEPHISAETLEYHYGKHHQTYVTNLNNLIKGT-EFENLSLEETIRKA-------SGGLFNNAAQIWNHTFYWNCLSPQGGGEPSGALADAIKRDFGSFESFKENFSKTAITTFGSGWGWLVKKPD-GTLALESTSNAATPLTGENKPLLTCDVWEHAYYIDYRNARPKYVEAFWNLVNWDFVAENFAA---

>A0A085JMH6_1005995_Proteobacteria_B_Mar

MSYSLPSLPYAYDALEPHFDKETMEIHHTKHHQAYVNNANAALEG-TEFASLPVEELITKLDQVPADKKTALRNNAGGHANHSLFWKGLKT--GTTLQGELKAAIEKDFGSVEKFQAEFEKAAATRFGSGWAWLVKQGD--KLAVVSTANQDSPLMGSGFPILGLDVWEHAYYLKFQNKRPDYIKAFWNVVNWDEAAARFASAK-

>A0A0C7NIR4_1006576_Thermotogae_B_Anc

MRFELPKLNYSYDSLEPYIDSRTMEIHYTKHHATYVNKLNEALEKHPELMNKNLEDLLKDLDNIPSDIRTTVRNNGGGHYNHTLFWSIMGPNGSRKPIGKLAQRIDEVFGSFDKFKLEFSDSALNRFGSGWAWLVLDKY-GHLSIISTPNQDNPIMIGLYPILGIDVWEHAYYLKYQNRRAEYIESWWNVVNWEEVEKNYLNIV-

>L8LWB9_102125_Cyanobacteria_B_Ter

MAYQLPSLPYDYTALEPTISKSTLEFHHDKHHAAYVSKYNNAVQGT-EFDSKSIEEVIKAIAK--DESKTGLFNNAAQAWNHTFYWQCMKPNGGGTPTGELAKKIDADFGSFEKFAEAFKNAGATQFGSGWAWLVLD-G-DTLKVTKTLNADNPLTSNQVPLLTMDVWEHAYYLDYQNKRPAYIDDFLKLVNWDFVAQNLAAA--

>U2EL32_1033802_Proteobacteria_B_Mar

MAFELPDLPYDYDALEPYIDGRTMEIHYEKHHNAYLTKFKKAIEG-TELEDQDLETILSKAGQHG----PGVRNQGGGFYNHILFWDSMSPSGGGKPTGGLGTAIDSSFGSFDSFKEEFTNAATGLFGSGFVWLVPQ-G-NQLKIVSTQNQDNPIMDSGKPVMGLDVWEHAFYLKYQNRKPEYVDAWWNVVNWDGIAKRYDEQVG

>F7PIA8_1033806_Halobacteria_A_Arc

SNPELPSLPYDYDALEPHISEQVLEWHHDTHHQGYVNGLDAAEETLAENRASGDH---STTG----GALGNVTHNGSGHYLHTLFWENMSPNGGGEPEGALLERIEEDFGSYDAWKAEFEAAAG--AAGGWALLVYDPVAKQLRNVAVDKHDNGALWGSHPILALDVWEHSYYYDYGPDRGSFIDAFFEVLNWEKVAEEYQKSLD

>A0A1H6UCM7_1043493_Actinobacteria_B_Ter

ADYTLPDLSYDYGALDPHIAGEIMELHHSKHHAAYVAGANTALEKMAGARESGDF---GAIA----GLEKALAFNLGGHTNHSVFWTNMSPDGGDKPEGELAAAIDEHFGSFDGFRAHFTNNAMTIMGSGWSILAWDTIGKKLIIVQLYDQQGNVPVGIVPLLMLDMWEHAFYLQYKNVKADYVNAWWNVVDWANVQQRFITATA

>A0A1B6B8G0_1048380_Firmicutes_B_Ter

MKFQLPNLTYAYDALEPHFDKQTMEIHHTKHHAGYTNNLNAALEG-KDI-DLSIEAILKGLEAIPEDVRVAVRNNGGGFYNHSLFWTLLSPNGGGEPKGKLKEALVKQFGSFETFKENFNEAAAKRFGSGWAWLTIK-D-GVLGIESTANQDSPLSDGKDVLLGLDVWEHAYYLKFQNKRPDYISAFWHLVNWDEVESRFEQFK-

>G2FFW9_1049564_Proteobacteria_B_Mar

MAHELPALPFAIDALEPVISQETLEFHHGKHHNTYVTNLNNLIPGT-EFENASLEEIIMKS-------EGGVFNNAAQVWNHTFYWNCLSPGDENAPTGALADAINSTFGSFDEFKQKFATSGATNFGSGWTWLVQNED-GSLEIYNTSNAGTPMTAGKKALLTADVWEHAYYIDYRNARPAYLDAFWKLVNWDFVASNMG----

>L0AA53_1056495_Thermoprotei_A_Arc

KRYELPPLPYSYDALEPVLSRDILTYHHDKHHLAYVNGANAAMEKLEKYLNGQEQS--IDIR----AVSRDFEFNYGGHLLHTLYWLNMAPTGGGTPGGTIADAINKNFGSFDKFKKVFGDAAKLVEGVGWAILALDPVTGDLKITQVEKHNAVITMNLVPLLACDVFEHAYYLQYKNDRGSYVDKWWDVVNWDDVEKRYQKALT

>U1QK35_1070774_Halobacteria_A_Arc

MSYELDPLPYEYDALEPHLDEQVLTWHHDTHHQGYVDGWNSAEETLAENRENHDF---GSSA----GAIRDVTHNSSGHILHDLFWQNMSSEGGDEPSGDLADRIAEDFGSYEAWKGEFEAAAG--SASGWALLVYDTFSNQLRNVVVDNHDEGAVWGGHPVLALDVWEHSYYYDYGPNRGEFVDNFFEVVDWDEPSARYQQAVE

>W0TLC1_1076588_Proteobacteria_B_Mar

TTHTLPVLPYAKDALQPHISVETLDYHHDKHHATYVDNLNNLIPGT-EFENASLEEIVMKA-------EGGIFNNGAQVWNHTFYFNCMSPNGGGEPGGALADAINSAFGSFADFKEKFSTSAATNFGCGWTWLVKNSD-GSLEIVNTSGAGNPMRDGQTPLLTIDVWEHAYYIDKRNVRPQYIADFFNVVNWDFVADNLG----

>U1QN78_1085028_Halobacteria_A_Arc

SNPELPSLPYDYDALEPSISEQVVTWHHDTHHQSYVDNLTAAEETLAENRESGDY---GGSA----AAIRNVTHNTGGHYLHTLFWENMHPNGGGEPEGELRDRIEADFGSYEGWKGEFETAAG--DASGWALLVYDPVSKQLRNAVVDNHDEGAIWGSHPILSLDVWEHSYYYDYGPDRGSFVDGFFEVVDWSKVSEEFQTCLD

>A0A1H0IML9_1090615_Actinobacteria_B_Ter

AQYTLPELPYDYAALEPFISGQIMELHHSKHHAAYVAGANTALDQMAEAREKDAF---GTIN----LLEKNLAFHLGGHINHSVFWPNLAPGGGDKPDGELGAAIDEFFGSWDGFRKQFSANANAIQGSGWSILAWDTLGQRLNIMQLFDQQGNLPAAQIPIVLLDMWEHAFYLQYKNVKADYVTAWWNVINWADATARFTAARD

>K4MC62_1094980_Methanomicrobia_A_Arc

ELYKLPALKYGYADLEPYISEEQLRIHHDKHHQSYVNNANSLLQMMDKAREEGTD---FDYK----ATAKAFAFNLGGHVLHDYFWWEMTPAASKEPTGELSDAVKENFGGFERFKKEFSQVASGVEGSGWAALTFCTDTNRLGIMQIEKHNVNLIPDYPIIMALDVWEHAYYLDYKNERGKFIDAFWNIVDWEELDKWFKKVQN

>G7VBY8_1104324_Thermoprotei_A_Arc

KKYTLPELPYAYNALEPYIAEEIMKLHHQKHHQGYVNGANAALEKLEKFRKGEAQ---IDIR----AVLRDFSFHLNGHVLHSIFWPNMAPPGGGKPGGKTADLINQFFGSFEKFKEEFSQAAKNVEGVGWAILVYEPLAEQLVILQVEKHNLMHAADAQVLLALDVWEHAYYLQYKNDRGSYVDNWWNVVNWDDVERRLQKALN

>K6WAV5_1108045_Actinobacteria_B_Ter

AEYTLPDLPYDYAALEPHISGRIMELHHDKHHATYVKGANDTLEKLAAAREDGTI---AKVY----GLSATLSFHLGGHTNHSIFWKNLSPNGGDKPEGDLAAAIDDQFGGFDKFQAHFTAAATTLQGSGWAILGYDTIGKNLVILQLTDQSGNIPAAIIPVVMLDMWEHAFYLDYQNVKPDYVKAWWNVVNWADAAERFATASS

>G7WL45_1110509_Methanomicrobia_A_Arc

KFYSLPELPYAYNALEPQISEAQLRLHHDKHHAAYVNGANAILERLDKAREAGAD---LDMK----ATLKELSFQAGGHLLHSLFWRNLAPAAAEEPKGALADALKEEFGSFARFKKEFSAAAATAEGSGWAALAWCGMTNRPVIMQIEKHNVNVYPMFRILMVLDVWEHAYYLDYKNERPKFVEAFWKIVDWDEVSRRLEAAIK

>P77968_1111708_Cyanobacteria_B_Ter

MAYALPNLPYDYTALEPCISKSTLEFHHDKHHAAYVNNFNNAVAGT-DLDNQSIEDVIKAVAG--DASKAGIFNNAAQAWNHSFYWNCMKPGGGGQPSGALADKINADFGSFDAFVEAFKQAGATQFGSGWAWLVLD-N-GTLKVTKTGNAENPMTAGQTPLLTMDVWEHAYYLDYQNRRPDYIADFLKLVNWDFVAANLAAA--

>L9VRX1_1114856_Halobacteria_A_Arc

TDHELPPLPYDYDALEPSISEQVVTWHHDTHHQGYVNGLNAAEETLAENRESGDF---GSTP----GALSNVTHNGCGHYLHTLFWENMSPNGGGEPEGDLADRIEEDFGSYEGWKGEFEAAAG--AAGGWALLVYDPVAKQLRNVAVDKHDQGALWGSHPILACDVWEHSYYYDYGPDRGEFIDGFFDVVNWDSAAEEYQKCLD

>A0A3D9SUE6_111806_Actinobacteria_B_Ter

ATYSLPDLPYDYAALEPAITGEILELHHAKHHAAYVKGANDTLERLEEARDKEQY---GGLV----GLEKTLAFNLSGHVLHSIFWDNLSPDGGDRPDGELAAAIDEHFGSFEAFRKQLSTATATVQGSGWGVLGWEPLGRRLIVQQVYDHHGNVGLGATPLLVFDAWEHAYYLQYRNVRPDYVEKLWSLVNWTDVSARFTAAKG

>A0A1T4W9E9_1121442_Proteobacteria_B_Mar

AAPKAPQLPYAKGSLAPAISQKTLDFHYGKHTLGYYSKLAALISGT-KYAKMPLEDIIRSTAS--NKSTRSIYNNAAQAWNHTFYWQGLKAKGGGMPPDAFGADLMETYSTLAHFQDVFIATAASVFGSGWAWIVKDKA-GKIDIIGTQGADTPLTMNKTPLFVVDVWEHAYYLDYQNRRSDYVQAFYYLANWDVVSKRYNAK--

>A0A259UH52_1123289_Firmicutes_B_Ter

GKHRLPPLPYAYDALEPIISEDTLRIHHDHHHKAYVDGLNKAEVALAQARAGKDY---TFIK----YWENELAFNGSGHILHSIYWTILTQKGGGEPGPDTVREITRRFVSFDAFLEQLIAATIKVEASGWGILAWNPSWNYLEILIAEKHQNLTQWGSIPILVLDVWEHAYYLDYQYQREEYVRRILELINWNEVETRLDLAIR

>A0A1H3A4M8_1123352_Firmicutes_B_Ter

MVFKLPELKYSYNALEPHIDALTMETHHSKHHKAYVDNLNKALEGHAKSQEMDIEEILKSLTELPEEIRTAVRNNGGGHYNHTLFWEFMSPDGGGKPEGELANKIDEDLGGFDKFKEDFKKAALDQFGSGWAWLVLN-N-GRIEIVSTPNQDNPISQGKIPILGIDVWEHAYYLKYKNLRGDYVDAWWNVVNWKKVEEIFNKVK-

>A0A1I0EA93_1123402_Proteobacteria_B_Mar

MSYSLPELPYAYDALEPHFDKETMEIHHSKHHQAYVNNANNALADLPDLAKLSVEALIADLDKVPAEKRPVLRNNAGGHANHSLFWKGLKV--GTTLAGDLKAAIERDFGSVDAFKEKFEQAAATRFGSGWAWLVYQ-D-GKLAVVSTANQDSPLMGSGFPIIGLDVWEHAYYLKFQNRRPDYIKAFWNVVNWDEAANRFAQVK-

>A0A368N5W7_1126245_Halobacteria_A_Arc

MSYELDPLPYDYDALEPHISEQVLTWHHDTHHQGYVNGWNSAEETLEANRGEDDF---GSSA----GAIRNVTHNGCGHILHDLFWNCMSPEGGDEPSGDLADRIAEDFGSYEAWKGEFEAAAG--DASGWALLVYDSFSNQLRNVVVDEHDQGALWGSHPVLALDVWEHSYYHDYGPARGDFVDNFFQVVDWSEPSTRYEQAVE

>K0AYP0_1128398_Firmicutes_B_Ter

NTINIKQFNFNN------ISRNQLDQHY-ELYKGYVRNINKIWDILDQTPENDSNPTYSPLR----CLRLGETYALDGVKLHELYFENLNSN-YNRPFGPIVDLIIRDFYSFERWENLFKQTGL--AMRGWVVLAIDHIDKRLHIYGQDEHDKGSIWMAHPLLVLDVYEHAYMIDFGIDRKKYIDIFMENIDWNIVNQRLEMFML

>I4EEM3_1129897_Chloroflexi_B_Ter

MAFTLPPLPYAYDALEPYIDEQTMHLHHDRHHAGYVNNLNAALESAPEFFNQSIEDILRNINDVPEAIRTAVRNNGGGAENHTMFWEIMGPNGGGEPTGDLANAINSAFGDFATFKDKFSKAGATRFGSGWAWLVAR-D-GTLDVISTANQDSPFMDGLYPVMGIDVWEHAYYLKYQNRRPEYISAWWNVVNWPAIADRYAKAIG

>M0M2F6_1132509_Halobacteria_A_Arc

SHPELPPLPYDYDALEPHISEQVLEWHHDTHHQGYVNGLESAEETLAENRESGDH---STTG----GALNNVTHNGSGHYLHTMFWENMSPNGGGEPDGDLADRIAEDFGSYEGWKDEFEAAAS--AASGWALLVYDPVADQLRNVAVDKHDDGALWGAHPVLALDVWEHSYYYDYGPDRGSFVDAFFEVVDWDAAADNYEQTVS

>A0A1Z5HC40_113268_Proteobacteria_B_Mar

MAYELPALPYAKDALEPHISEETLEFHYGKHHQTYVTNLNNLVPGS-EFEGASVENLMMRA-------SGGIFNNAAQVWNHTFYWHSLSPNGGGEPTGALADGINAAFGSFAEFKEAFTKCAVTTFGSGWAWLVKNAD-GSLALVSTSNAACPLTEGQTPLLTCDVWEHAYYIDYRNARPKYLEAYWALVNWEFAAENYAG---

>A0A081NMU1_1137799_Proteobacteria_B_Mar

MAFELPALPYARDALAPHISEETLNYHYGKHHKTYVDKLNGLVAGT-ELESKSLEEIIKTS-------EGGIFNNAAQIWNHTFYWDCLSPNGGGAPSGELADAINKAFGSFEEFVAAFNDKAVNNFGSSWTWLVKNSD-GSVEIVNTSNAGTPLTTDQKPLLTCDLWEHAYYIDYRNVRPEYLKNFWALVNWNFAAANYAK---

>S0L434_1140003_Firmicutes_B_Ter

MAYELPELPYAYDALEPQIDVETMHLHHDKHHNTYVTNLNAAVEKYPELAEKSVEDLIADMDAIPSDIQTAVRNNGGGHANHSFFWEILTPNATEEPVGELKTAIEDTFGSLDALKEEFKKAATGRFGSGWAWLVVK-D-GKLAVTSTANQDSPLMEGQTPVLGLDVWEHAYYLNYKNVRPDYIDAFWKLVNWEKANELFVAAK-

>A0A2T0PUC9_1144618_Actinobacteria_B_Ter

SAYELPDLPYDYSALEPHISGAIMELHHSKHHATYVKGTNDAIEQLAEAREKQTL---GTVN----LLQKNLAFNLAGHVNHSVFWPNMSPDGGDKPEGGLAAAIEEFFGSFDAFRAHFTANALGIQGSGWSILAWDSIGRRLILEQLYDHQGNLAVGTYPLLMLDMWEHAFYLQYRNVKADYVKAFWNIVNWADVQERFERAQG

>A0A031LPG5_1160895_Thermoprotei_A_Arc

KKYELPPLPYNLDALEPYISKDIIDVHYNGHHKGYVNGANAFLDRVNKVLKGELASGQYDMM----GLMRGLVFNINGHKLHDLYWRNMTPSGGGKPGGSLADLIDKQYGSFEKFKQIFTEAANSLPGTGWTVLYYDVENGNLQIMTFENHFQNHIAELPILLILDEFEHAYYLQYKNKRADYVNNWWNVVNWDFADKKLQNYIK

>I2K993_1165841_Proteobacteria_B_Mar

MTHTLMDLPFDENALEPYISKETLQYHHGKHHAGYINKLNILIEGT-VYAEMKLEEIVTKA-------DGGIFNNAAQVFNHNFYFNGMSKK-ATSPSKELLALFERDFGSIESFKEKFLDMAAGLFGSGWVWLSIKDS-GTLSIESFSNAGNPLLLGHTPLLTCDVWEHAYYIDYRNARADYLEKWWELVNWDFVSKNLEASTQ

>A0A1I4SFU3_1166257_Proteobacteria_B_Mar

MAFELNDLPYAYDALGPYMSRETLEYHHDKHHLAYVNNGNNLIKGT-EWEGKSIEEVVKGSFG----KNPALFNNVAQHYNHFYFWQWMKPNGGGSIPGELLNKINEDLGGLDKFRADFIQAGMTQFGSGWAWLSLK-D-GKLAISKTPNGENPLVSGATPLLGVDVWEHSYYIDYRNLRQKYLEAFFNLVNWEFVAELYSKAG-

>I2JH08_1168065_Proteobacteria_B_Mar

MAFELPALPYAKDALQPHISAETLDFHHGKHHNAYVTKLNELLPGS-EFEGKTLEEIIKAS-------SGVMFNQAAQVWNHTFYWNSLSPNGGGAPTGDLAAAIDKAFGSFDAFKTAFNAKAVGNFGSGWTWLVKNSD-GSLEIVNTDDADTPIAEGQTPLITADVWEHAYYIDYRNARPKYLEEFWNLVNWEFAAANFA----

>K9VXC7_1173022_Cyanobacteria_B_Ter

MAFEQPPLPYDFNALEPHMSAKTFEFHYGKHHAAYVTNLNKLVQDT-EMADKSLEEVIKASFG--DSSKTGIFNNAAQVWNHTFFWNCMKPNGGGQPTGELADKINTAFGSFDKFKEEFKNAAVTQFGSGWAWLVKD-G-DTLKITKTPNAENPLAHGQTALLTVDVWEHAYYLDYQNRRPDFVQTVLNLINWDFVTQNLSA---

>K9SBX7_1173025_Cyanobacteria_B_Ter

MSYEFPNLPYAQDALEPHISANTLSFHHGKHHAKYVSTYNEMVQGT-ENESKSIEEVIKASYD--PSAKSGLFNNAAQSWNHTFYWYCMKPGGGGEPTGELAEKIKADFGSFEKFKEEFKAAGGSQFGSGWAWLVLD-N-GTLKVTKTPDAVNPIAQGQTPLLTMDVWEHAYYLDYQNLRPSYANTFLSLVNWDFVAEQYAKAK-

>A0A2P8H4Q1_1176648_Firmicutes_B_Ter

MAYQLPELPYAYDALEPHIDKETMNIHHTKHHNTYVTNVNAALEGNEELASKSVEELISDLSSVPEEIRTAVRNNGGGHANHSLFWQLLSPNGGGNPTGALAEAIDSKFGSFDEFKTQFENAGKTRFGSGWAWLVSN-N-GELEVTSTQNQDSPLMEGQTPLLGVDVWEHAYYLKYQNRRPDYLAAFWNVVNWEEVSRRFDATK-

>A0A1D3L2G7_118062_Methanobacteria_A_Arc

NFYELPELPYGYKDLEPYISEEQLKIHHKKHHQAYVDGANALLKKFDA-RAEEEE---FDLK----AVSKELSFHVGGFILHKLFWANMGPSCGGEPTGLIADYIEKDFGSFERFKKEFSQTAVSTEGSGWAALTLCRGTDRIFIMQIEKHNVNTVPGFRLMMVLDVWEHAYYLEYQNRRPDYVEAFWNLVNWDEVNRRIKVWLD

>U5QI99_1183438_Cyanobacteria_B_Ter

MAFTLPPLPYDESALAPYISAQTLSFHYGKHHKGYVDTLNKLVAGS-EAENTPLEELIKSVHG--QPDKAAIFNNAAQIWNHTFYWNSLKPGGGGEPTGTIAELIKDAFGSYDEFKKQFITAGTTQFGSGYAWLVKDKS-GKLSVIKTPNAETPLTDPTVPVLTFDVWEHAYYLDYQNLRPKYEEAVVHLLNWEFAEKNLASA--

>K6W4W1_1184607_Actinobacteria_B_Ter

-MYTLPDLPYDYAALEPAISGQIMELHHAKHHATYVKGCNDTLEKLADARDKNDF---GSIV----GLEKTLAFNLAGHALHTIFWENLSPQGGGEPTGPLAEAIQKNFGSFEAFKAHMTAASTTIQGSGWGMLAYEPLDGRLIVSQHYDHQSNVNFGAAPLLVFDVWEHAFYLQYLNVKADYVSALWNIVNWDDVSRRFAAARG

>A0A1H9BZT2_1186196_Halobacteria_A_Arc

ADHELPPLPYDYDALEPALSEQVLTWHHDTHHQGYVNGLNSAEETLAENREEGDF---GSTP----GALSNVTHNGCGHYLHTLFWENMSPNGGGEPEGDLADRIEEDFGSYEGWKGEFEAAAG--AAGGWALLVYDPVAKQLRNLAVDKHDQGALWGAHPVLALDVWEHSYYYDYGPDRGSFIDAFFDVVNWEKAEEEYQTCLD

>A0A4R2PEU5_1188247_Proteobacteria_B_Mar

-MLTLPDLPYDYKALEPYMSAETLEYHHDKHHNAYVTAGNKLMEGT-PYADMDLESAIRESFK----SDPKLFNQIAQHWNHMEFWHWMSPKGGGAIPGNLESQIKKDFGSVDDFKEAFVQAGVTQFGSGWCWLVWK-D-GKLAVTQTPNGENPLVHGGTALLGCDVWEHSYYIDYRNARPKYLEAFFHLVNWERVTEIYEQAS-

>I6ZUA8_1191523_Ignavibacteriae_B_Mar

SKFELPALPYAFDALEPYIDARTMEIHHDKHHAGYVNNLNKAVEG-TEYEGKSLEELFKTVSKLP----VAIRNNGGGHYNHSMFWQIMGPNKGGEPTGALADAINGTFDSFEKFKETFNNAAATRFGSGWAWLVLS-N-GKLVVTSTPNQDNPLMDQGFPVMGLDVWEHAYYLKYQNRRPEYITNWWNVVNWDEVAKRFDSVK-

>A0A2Z2P4N8_1192854_Proteobacteria_B_Mar

MSFTFPDLPYDFTALEPHVDAQTMEIHYDRHHRTYFNNFTGAVNDTP-LADKSLEEVMAQVDS---STSPAIRNNGGGYYNHILYWNSMC-NGGSAPSSELATAIDSTFGSMAELKTGLKQAGITRFGSGFAWLIVK-N-GKLEITSTPNQDNPLMSQGTPILALDVWEHAYYLKYQNKRPDYIDGWLEVVDWAKTSERFAAASA

>A0A0C9Q165_1197129_Planctomycetes_B_Mar

AAFGIPTMSYNASKLIG-FSDTLLKNHF-TLYQGYVTNTNKVLDTLSQMLKEGKT-ANPEFA----ELKRRLGWEFNGMRLHEYYFENLGGKAGINKEGKLAKKMADAFGDYATWEKDFRAVGA-MRGIGWAVLYQDSANGQLINFWVNEHDVSHPAGCNPILIMDVFEHAFMIDYGLKRADYIEAFFKNIDWSAAEARLK----

>L9PNM3_1198452_Proteobacteria_B_Mar

MEHTLPPLPYAIDALAPHISQETLEYHYGKHHQTYVTNLNNLIKGT-EFENLSLEEIVKKS-------SGGIFNNSAQVWNHTFYWNGLTPNGKGAPDGALADAINAKWGSFDKFKEEFTKSCVGNFGSSWTWLVKKAD-GSLDIVNTSNAATPLTTDAKALLTCDLWEHAYYIDYRNVRPKYVETFFKLANWDFAAANFA----

>A0A0D0S5X1_1199154_Proteobacteria_B_Mar

MAIELPPLPYDRTALEPHISGETIDFHYGKHHKSYVDNLNKMIEGT-EFADMPLEDIIRKS-------QGGMFNNAAQVWNHTFYWNCLSPNGGGEPTGKLAEAISKSFGDFATFKEQFSDTAVKTFGSGWGWLVQRTD-GSLALVSTSNANTPLTGSDTPLLTCDVWEHAYYIDYRNARPKYVETFWKLVNWDFVASNLK----

>I7KYD2_1201294_Methanomicrobia_A_Arc

KKYELPPLPYAPDALEPHISKEQLSLHHDKHHQAYVTGANANLERLEKARQEGTD---VDMK----ALLKELSFNIGGHILHTLFWPGMAPAGGGTPGGALADLIDREWGSFDRFKAEFSKAAASVEGSGWAALAYCTMTDRPMIMQIEKHNNNVYPTFQILMVLDVWEHAYYVDYKNNRGQFVDAFWNVANWDEVNRRLEKI--

>A0A1I0M6P7_1202768_Halobacteria_A_Arc

ADHELPPLPYDYDALEPALSEQVLTWHHDTHHQGYVNGLNSAEETLAENREEGDY---GSTP----GALKDVTHNGCGHYLHTLFWENMSPNGGGEPEGDLADRIEEDFGSYEGWKGEFQKAAG--AAGGWALLVYDPVAKQLRNLAVDKHDQGALWGAHPVLALDVWEHSYYYDYGPDRGEFIDAFFDVVNWEKAEEEYQTCLD

>A0A0F5IRT0_1203610_Bacteroidetes_B_Mar

MKFELIQLPYAANALEPVISKETIEFHHGKHLQAYVNNLNNLIPGT-KFENAPLEQIVAES-------DGAIFNNAGQILNHNLYFTQFSPKGGGKPSGKLAEAIDKTWGSFENFQKEFNTAGAGLFGSGWVWLAKDKD-GKLSITKEANGSNPVAKGLTPILGFDVWEHAYYLDYQNRRPDHLAALWKIVDWDAVGKRY-----

>M1L4D0_1208918_Proteobacteria_B_Mar

MIHVLPDLPYKIDSLSPYISKETLEFHYGKHHQTYINNLNSLIQNT-EFQEMSLEEIIKKS-------SGAIFNNAAQVWNHNFYWNSICPDSTKKPEGNLADAINQKWGNFESFKLEFNKQAASNFGSGWTWLVKKSD-RSLEILNTSNAGTIVNSNDKAMITCDVWEHAYYIDYRNARVKYLENFWSIINWKFAEQNFSL---

>M1M096_1208922_Proteobacteria_B_Mar

MSYNLPPLPYDASSLSPYISKETIEFHYGKHHKTYVDNLNNLISDN-ELSKLNLDDIIKNS-------TGAIFNNAAQIWNHNFYWNSLSPIFNQHPSDILLKSIELKWGNFENFKESFTRSALSNFGSGWTWLVKKTD-GSLDIVNTSNANTPIITEDKALLTCDVWEHAYYIDYRNSRAKYLENFWEIINWNFVSSNFSG---

>A0A0W0GJW2_1217799_Chloroflexi_B_Ter

-------MAYDTKKLLG-FSDNLLNNHF-TLYQGYVKNLNRLVETIDALSKAGKS-DTPEYA----ELQRRFGFEWNGMRLHEYYFDNLGGTGLAVSGGKLAQNLAATWGSREAWEKDFRATGA-MRGVGWVVLYQDIQNGRLFNTWINLHESGHLTGCQPVLVMDVWEHAFMIDYGLKRAEYIEAFFRNIDWSVCELRIK----

>A0A1I5ZIY7_1227077_Bacteroidetes_B_Mar

MAFELPKLPYAYDALEPTFDAQTMEIHHSKHHQAYVTNLNAAITG-TEMENQSIEEIMHNIAKAP----APVRNNGGGHFNHSLFWTILSPNGGGQPSGAIGEAITSAFGSYDKFKEEFTKAATTRFGSGWAWLCKQAD-GSVQICSTPNQDNPLMPKGLPVLGLDVWEHAYYLKYQNRRPDYIAAFYNLINWDEVNKRFAEATP

>M0MM71_1227455_Halobacteria_A_Arc

SNAELPPLPYDYDALEPHISEQVLTWHHDTHHQGYVNGLNSAEETLAENRESGDY---SSTG----GALGNVTHNGCGHYLHTMFWENMDPNGGGEPSGELADRIEEDFGSYEGWKGEFEAAAS--AASGWALLVYDPVANQLRNVAVDNHDEGALWGAHPVLALDVWEHSYYYDYGPDRGSFIDAFFEVVDWDAAAENFEKTTS

>M0DHS9_1227487_Halobacteria_A_Arc

MSYELDPLPYDYDALEPHISEQVLTWHHDTHHQGYVNGWNSAEETLESNREEGDF---SSSG----SALRNVTHNGSGHILHDLFWQNMSPEGGEEPSGDLADRIEEDFGSYDAWKGEFEAAAK--NASGWALLVYDSFSNQLHNVVVDKHDQGALWGSHPILALDVWEHSYYHDYGPARGDFIDAFFEVVDWEESSSRYNEAVQ

>L9ZI72_1227494_Halobacteria_A_Arc

TDHELPPLPYDYDALEPALSEQVLTWHHDTHHQGYVNGLNAAEETLAENREEGDF---GSTP----GALKNVTHNGCGHYLHTLFWENMSPNGGGEPDGDLADRIEEDFGSYEGWKGEFEAAAG--AAGGWALLVYDPVAKQLRNVAVDKHDQGALWGAHPVLALDVWEHSYYYDYGPDRGDFIDAFFDVVNWEKAEEEYQTCLD

>M0CBS2_1230457_Halobacteria_A_Arc

TDHELPPLPYDYDALEPSISEQTLTWHHDTHHQGYVNGLNAAEETLSENREEGDF---STTP----GALSNVTHNGCGHYLHTLFWENMSPNGGGEPEGDLADRIEEDFGSYEGWKGEFEAAAG--AAGGWALLVYDPVAKQLRNIAVDKHDQGALWGSHPVLALDVWEHSYYYDYGPDRGSFIDSFFDVVNWEKAEEEFQKCLD

>L9WFH7_1230460_Halobacteria_A_Arc

TDHELPPLPYDYDALEPSISEQVVTWHHDTHHQGYVNGLNAAEETLAENRESGDF---GSTP----GALGSVTHNGCGHYLHTLFWENMSPNGGGEPEGDLADRIEEDFGSYEGWKGEFEAAAG--AAGGWALLVYDPVAKQLRNVAVDKHDQGALWGSHPILALDVWEHSYYYDYGPDRGDFIDAFFDVVNWEKAAEEYEKCLD

>A0A095XIK9_1230730_Firmicutes_B_Ter

MAFTLPELPYAYDALEPVIDKETMQFHHDKHHDTYVNNLNKALEKAPEWAAKDIEEIVADWDNAPEEVRQAIRNNGGGHYNHSLFWQMMTPVKDSKLEGAIQAQIEKDFGSFDAFKKEFGAAATGRFGSGWAWLVWN-G-KKLAVVSTPNQDNPLADGQKPLLGIDVWEHAYYLKYQNKRADYVDNFFKVINWKFVNELFAAAKK

>A0A223S566_1235441_Actinobacteria_B_Ter

APYSLPELPYDYAALEPWISGQIMELHHDKHHAAYVGGANTALEKLAEAREKGDF---GTVN----MLEKNLAFNLAGHVNHSVFWPNLSPDGGDKPEGELGAAIDDQFGSFDAFRAHFSAVATGIQGSGWAILAWDILGQKLIIEQLYDHQGNLAAGSYPLLLLDMWEHAFYLQYKNVKADYVKAFWNVVNWADVQRRFEEARK

>D5VB54_1236608_Proteobacteria_B_Mar

MAFTLPELGYSYDALEPHFDKETMEIHHSRHHQAYVNNANGLLEG-TQWADKSAEEVIANLDQIPADKRTGVRNNAGGHANHSLFWTILKT--GTTLGGSLKDAIVRDFGSVEAFQEQFEKAAASRFGSGWAWLVLD-E-GSLKVVSTANQDSPLMGKGYPIIGLDVWEHAYYLKYQNKRPDYIKAFWEVVNWDEAQKRFDSAN-

>A0A0S2KFC6_1249552_Proteobacteria_B_Mar

MKLELPELPYAANALEPHMSAETFSFHHGKHHNAYVVKGNELLADA-GIDADNLEDLVRESAK----VGGPLFNNVAQVFNHNFFWKSMKPNGGGAPTGAIADKINADFGSYDNFKKEFVNGGVGQFGSGWVWLVLD-G-GKLKIAKTPNAECPLTTSAKPILVCDVWEHAYYLDYQNRRPDFLTSFLNLVNWDFANENLA----

>A0A0D3LFT2_1257021_Bacteroidetes_B_Mar

MAFELPNLPYPSNALEPNIDQQTMEIHHGRHHKGYTDNLNKAIQG-TDMENKSIEEILKNVSGN-----NAVRNNGGGYYNHNLFWEVMSPNGGGQPTGAVAEAINKKFGSFDKFKEEFSNAAATRFGSGWAWLIVTGN-GEVDVTSTPNQDNPLMDKGTPILGLDVWEHAYYLKYQNKRVDYVGNWWNVVNWEAVNRRFEQARN

>L8XU36_1261130_Proteobacteria_B_Mar

MAYTLPDLPYAHDALEPHIDTETMHLHHDKHHNTYVNNLNNAIANHPELAQLPIDELIAKLDVVPEEIRNAVRNNGGGHANHTLFWEIMAPNAGGEPTGEIKAAIEKAFGSFDAFKEKFAAAATGQFGSGWAWLVVNKD-GDLEVMSTSNQDSPLTIGKTPVLTIDVWEHAYYKKFSNVRPDYIKAFWNVVNWNEVNRRFLAAK-

>U2YRD9_1261545_Halobacteria_A_Arc

TDYELPPLPYDYDALEPSISEQVLNWHHDTHHQGYVNGWNAAEETLEEAREEGDF---SGSP----GAIRNVTHNGSGHVLHTLFWESMGPNGGDEPSGALAARIQEDFGSYEAWKGEFEAAAS--AAGGWALLVYDTHSNQLRNLVVDKHDQGALWGAQPVLALDVWEHSYYYDYGPARGDFIDAFFDVVDWETPADRYEEAVA

>R5JCK6_1262737_Bacteroidetes_B_Mar

MTYEMPKLPYANNALEPVISQQTIDYHYGKHLQTYVNNLNSLAPGT-EFEGKTVEEIVAKA------PDGAIFNNAGQVLNHTLYFLQFAPKSKKEPSGKLAEAIKRDFGSFENFKKEFNAAAVGLFGSGWAWLSVDKN-GKLHITKEANGSNPVRAGLKPLLGFDVWEHAYYLDFQNRRADHVNALWDIIDWEVVDKRM-----

>R5L6H9_1262760_Spirochaetes_B_Mar

--FELIKLPYGKEDLAPYMSSNTLDFHHGKHLNAYVTAVNDFVSDK-SLEGKSIEELILLSHN--NADKQGLFNNAGQVYNHEEFFKVLKKSEKPSIPSELESKIKSDFGSFDAFKEAFTTAGKTQFGSGWAWLVLA-N-GKLEVRKYPNAMNPIADKVHGLLTCDVWEHAYYLDYQNRRPDFLNTFVHLVNWEYVAEKLKNAK-

>R6GYH2_1262911_Firmicutes_B_Ter

YPFILPPLPYPPAALEPNLGRSSVRLHHDAFFAAYVDRLNGALAPWLQYQDWPLERLLLHWCQLPRALGQAVRRYGGGTYNHTLYFSSLAPARTTKPSPVLLAEIEHSFGSMEGLHRSMKNAAASIFGSGWVWLVCC-S-GGLQIVCTANQDTPLP--LWPLVNMDLWEHAFLPDYENRRDDYMEAALAIIDWDAASRRFEERTA

>R7QTT1_1262942_Firmicutes_B_Ter

YPFQNPPLPYAYDALEPYIDEKTMHLHHDRHLQTYVDNLNAALLPHPELHDKSLVFLLTHQNLLPCGTQIPIRNNAGGVYNHIFYFSGLTPDADR-PPDPLSAFLIESFQSPEKFKEKFKAATLSVFGSGYAWLTIEPT-GRLCIFVTKNQDTPLPAGLFPLLNIDVWEHAYYLKHYNERAAYIDDWFHIADFARANEHLLSFLH

>R6A170_1262986_Proteobacteria_B_Mar

MAFTVPPLPFAMDALEPYMSSKTLSYHYGKHNKAYNDTLNNLIKGT-DYETMPLEEIIRTT------QSGPIFNNAAQCFNHTFFWNSLKPNGGGQPTDVIAAQINAKWGSYDAFADAFTKSAVGNFGSGWTWLVEKED-GTLDIVNTSNAGTPITGTDVPLITCDVWEHAYYLDYYNMRVTFVQTFLHLVNWDFAAKNYK----

>R6EU58_1262994_Firmicutes_B_Ter

YPFVVQPLPYEYDALLPVLDEETLHFHHDKHYQTYVDKLNAILADYPQLQQMTLTELLTSLASLQEEARESIHNNGGGVYNHQLYFDSMRSPVGQEPCGALEEALIRDFGSVRQWKEQMSQSAIGVFGSGWAWLVSDQD-GTLMILTTANQDVPDLRLYAPILLIDVWEHAYYLQYRNRRPDYVQGWHKLLHWKKAERRYEQVLC

>R5HFY3_1263001_Firmicutes_B_Ter

YPFALPPLPYAYDALEPYVDEATMHFHHDKHLKTYVDNLNKALEAYPQYHTWTLETLLTKLEELPDGLRTAVRNNGGGVYNHDLFFDLMAPAGQ-----KISPAVAERFGGEEAWQKEMKAAALGQFGSGFAWLVADSS-GDLHIIALPNQDNPLSQGLTPILPLDVWEHAYYLKYQNLRADYIDAWFHVINWDGVNKRLHRG--

>A0A1H6J821_1267564_Halobacteria_A_Arc

SEHELDPLPYDYDALEPHISEQVLTWHHDTHHQGYVNGLNAAEETLAENRESGDY---GSTA----SALGNVTHNGSGHYLHTLFWNNMSPNGGGEPAGDLADRIAEDFGSYEAWKGEFEAAAG--AAGGWALLVYDPVSKQLRNLKVDKHDQGALWGAHPILALDVWEHSYYHDYGPARGEFIDAFFEVVDWGEVADNYENVVS

>A0A0H4WNU9_1297742_Proteobacteria_B_Mar

MPFTLPELPYKKDALQPHMSAETLEFHHDKHHAAYVNNLNKLLDGK-AEANKSLEEIILSS-------DGGVFNNAAQVWNHTFFWNCMKPAGGGKPTGELAAAIDRDFGSFDKFKEEFSTAAATQFGSGWAWLVLE-G-GKLKVTKTGNADLPMKHGQKALLTIDVWEHAYYIDYRNLRPKFIETFLHLVNWDFVAQNLKG---

>A0A168EFF5_1300344_Actinobacteria_B_Ter

AVYTLPDLPYDYSALEPHISGKIMELHHDKHHAAYVTGANTALDKLAEARESGDL---AAVN----LHEKNLAFNLGGHVNHTVFWNNLSPEGGGEPEGELAEAIKDQFGSFAAFQAQFTAVATGIQGSGWSVLGWDSIGGRLAIFQLFDQQANVPVGITPILMLDMWEHAFYLDYLNVKADYVKAFWNIANWQDVAARLERART

>U2QZH4_1321779_Fusobacteria_B_Anc

--FEQVKLPYAFDALEPNIDTKTMEIHYGKHHAAYTNNLNDALKNAPEFLEKPIEEILSNLDALPEAIRGAVRNNGGGFYNHNLYFTVMGPNAGGEPTGELAEKINEKFGSFEEFKVEFSKAAATRFGSGWAWLVVNKK-GELRVTSTANQDNPLMPEGTPILGIDVWEHAYYLNYQNRRPDYITAFFNVINWDAVAERYSKAK-

>V4HNI7_1324957_Halobacteria_A_Arc

SEYELPPLPYDYDALEPHISEQVLTWHHDTHHQGYVNGWNSAEETLEQNREEGEF---GSSG----SALRNVTHNGSGHMLHTLFWQSMSPEGGDEPSGELADRIEEDFGSYDAWKGEFEAAAK--NASGWALLVYDSFSNELHNVVVDKHDQGALWGSHPVLALDVWEHSYYYDYGPARGDFIDAFFEVVDWTEPAERYEQATQ

>U1MGY5_1325472_Halobacteria_A_Arc

SQPQLPELPYEYDALEPHISEQVVNWHHDTHHQSYVNNLAAAEETLAENREAGDY---DGTA----AAIRDVTHNTGGHYLHTLFWENMHPDGGGEPSGELRDRIETDFGSYEGWKGEFETAAS--DASGWALLVYDPVTKQLRNATVDNHDEGAIWGAHPILSLDVWEHSYYYDYGPDRGGLIDGFFEVVNWDYVADQYDTVVS

>W7KP27_1326980_Thermoprotei_A_Arc

RKYELPPLPYKVDALEPYISKDIIDVHYNGHHKGYVNGANSFLDRLNKVIKGEVQAGQYDIQ----GILRGLVFNINGHKLHALYWENMAPNGGGKPGGALADLIDKQFGSFDKFKAVFTEAANSLPGTGWTVLYYDTESGNLEIMTFENHFQNHIAELPIILILDEFEHAYYLQYKNKRADYVNNWWNIVNWDWADKKLQKYLN

>A0A380N0R1_13276_Proteobacteria_B_Mar

MSYTFQDLPYAYDALEPVIDEATMHLHKDKHHKTYFDKFVAAIAE-TDFNGKSLPEIFANISAL----SPAIRNNGGGYYNHDLFLNGMAPQENNTPSGELANAIKAAFGSFEDFQKAFADAAVNQFGSGWAWLVLQ-D-GKLVVSSTPNQDNPLMDRGTPILGLDVWEHAYYLNYQNKRPDYVSNWWKVVNWDYVSEQYAAAK-

>U6EAT7_1379702_Methanobacteria_A_Arc

KKYELPSLPYGYKDLEPYISEEQLRIHHDKHHQAYVDGANALLDKFDS-R-PGVE---FDVK----AVAKELSFHVGGFVLHKMFWENLAPAGGGEPTGTLAKYIEKDFGNLERFKEEFSQAAISTEGSGWAALTICRRTDRLFITQIEKHNVNVIPHFRVLMVLDVWEHAYYLDYKNVRPDYVAAFWNIVNWEEVNRRLEIELL

>A0A497X9T4_1381557_Proteobacteria_B_Mar

MEHTLPALPYAMDALAPHMSKETFEYHYAKHHQAYVTNLNNLIKGT-EYEALDLEAIVKKAP------AGGVYNNAAQVWNHTFFWNCMKPNGGGAPTGALADAINKKWGSLDEFKKAFQTSAVGNFGSGWTWLVKKAD-GSVDIVNMGAAGTPLTTGDKALLCVDVWEHAYYIDYRNLRPKFVETFLNLVNWDFAAKNFA----

>A0A256IHQ1_1383851_Halobacteria_A_Arc

MSYELDPLPYDYDALEPHISEQVLEWHHDTHHQGYVNGWNAAEETLEANREEGDF---SSSG----GAIRNVTHNSSGHILHDLFWQNMSPEGGDEPEGDLADRIEEDFGSYEAWKGEFEAAAS--AASGWALLVYDTFSNQLRNVVVDKHDQGAIWGGHPILALDVWEHSYYHDYGPARGEFVDNFFEVVDWEEPSARYEQAVE

>U9VUJ7_1385935_Cyanobacteria_B_Ter

GEFTLPPLPYDYDALDDYIDSETMTIHHDRHHAGYVRNLNAAIANYPELQNDSLEELISKVHELPLDIRANVRKNGGGHANHTMFWEIMTPNGQGRPTGAIAAAIDDTFGDFEQFKQIFKSAGLSQFGSGWVWLTLTKN-GQLRITRTANQDSPLMEGNYPILGNDVWEHAYYLKYQNRRGDYLDAWWNVVNWDEVNQRLERAQA

>A0A386UD88_1389713_Actinobacteria_B_Ter

AEYTLPDLDWDYAALEPHISGQINEIHHSKHHATYVKGVNDAVAKLEEARAKGDH---SAIF----LNEKNLAFHLGGHVNHSIWWKNLSPDGGDKPTGDLAAAIDDQFGSFDKFQAQFSAAANGLQGSGWAVLGYDTLGGRLLTFQLYDQQANVPLGVIPLLQVDMWEHAFYLQYKNVKADYVKAFWNVVNWADVQSRYEAATS

>U5Q5M1_1400053_Bacteroidetes_B_Mar

NTLEFPQLPYSTGALEPFIDQQTVEIHYGKHQKAYFDNFLAAVKG-TDAEKLTILEVFKNISKYP----AAVKNNGGGFYNHVIYWESIKPN-SGKPSAKLAEAINKKFGSMDELKKQFTDAGKTRFGSGWAWLSVDDK-GELFVSSTANQESPLMDQGTPILAMDVWEHAYYLKYQNRRPDYIESFWQIINWDVVSGKYEAIVK

>W3TXZ6_1402976_Proteobacteria_B_Mar

MAFELAKLPYDYDALSPYMSRETLEYHHDKHHLAYLTNTNNFVKDL-GLENESLEHIVKKSFG----QNIGLFNNAAQYYNHNHFWHWMKKGGGGKLPEKLAKAIESDLGGYNKFRADFIAAAVAQFGSGWAWVAVK-D-GKLEIMKTSNSENPLVHDAQPILGVDVWEHSYYIDYRNARPKYLEAFVNLINWDYVLKLYEDCGF

>V5ADP9_1408164_Proteobacteria_B_Mar

MEHTLPALPYAIDALAPHYSQEAFEYHHGKHHNAYVVNLNNLQKGT-EFEAMDLESIVKQS-------AGGVYNNAAQVWNHSFFWNCMKPNGGAAPTGALADAINTKFGSYDGFKEAFVKSATGNFGSGWTWLVKKAD-GSVDIVNTGPAGTPLTTDDKALMTVDVWEHAYYIDYRNARPKFVEAFFKLVNWEFAEKNFA----

>A0A1J4QJC3_1414654_Proteobacteria_B_Mar

MAFELPALPYAKNALEPHISQETLEFHHGKHHNSYVVNLNNLVPGT-EFEGKSLEDIIKTS-------SGGIFNNAAQIWNHTFYWNCLAPNGGGEPTGALADAIKAAFGSFDAFKEEFTKSCVTNFGSGWTWLVKKAD-GSVAIANTSNAGCPLTDNATPLLTCDVWEHAYYIDYRNVRPDYVKAFWALVNWEFVAENFAK---

>A0A1H8D259_1424661_Actinobacteria_B_Ter

AEYTLPTLDYDYAALEPSISGTIMELHHGKHHQAYVTGANTALAQLAEARDSGNL---ANVN----KLQKDLAFNLGGHVNHSIFWTNLSPNGGDKPVGELAAAIDDNFGSFDKFQAHFTAAALGVQGSGWAVLAWDSIGQRLIIQQFFDQQSNFAAGTVPVLMLDVWEHAYYLDYRNVRADYVKAFWNIANWANVQKRFDVARE

>A0A0E3NWV4_1434100_Methanomicrobia_A_Mar

ELYKLPPLKFGYADLEPYISEEQLRIHHDKHHQGYVNNTNSILQMMEKARKDGTD---FDYK----ATAKALAFNMGGHVLHSYFWWELTPAASKEPVGELAEAIKEDFGTFERFKKEFSQVAASVEGSGWAALTFCRDTKRLGFMQIEKHNVNLIPDFPVLLVVDVWEHAYYLDYKNERGKFIDAFWNIIDWEEIDKYFKTIR-

>A0A0E3NRG6_1434102_Methanomicrobia_A_Mar

ELYKLPPLKYGYADLAPYISEEQLRIHHDKHHQGYVNNTNALLEMMDKARKEGTD---FDYK----TTAKALSFNLGGHVLHDYFWWEMTPAASKEPVGELADVIKDDFGSFERFKKEFSQVASSVEGSGWAALTFCNDTKRLGIMQIEKHNVNLVPDFPILMDLDVWEHAYYIDYKNDRSKFIEGFWNIVDWEEIDKYFKKTQK

>A0A0E3WWB9_1434107_Methanomicrobia_A_Mar

GLYKLPDLKYGYSDLEPYISEEQLRIHHDKHHQAYVNNANSLIEMMDKARKEGTD---FDYK----ATAKALSFNLAGHVLHDYFWWEMTPESSKEPVGELSEVIKEDFGGFDRFKKEFSQVASSVEGSGWAALTYCKDTERLMIMQIEKHNVNLVPDYPIIMDLDTWEHAYYIDYRNDRAKFIEAFWNIVDWEEIDKYFKKMRK

>A0A3T0EBQ7_1434191_Proteobacteria_B_Mar

SAFSLPGLPYAYDALEPVIDAETMELHHSRHHQTYVNGLNSALEGRSDLQGTSLEDILARVSDLP----KAFRNHGGGHWNHTFFWESMTPADSGAPSGALAEAINAKFGSLDEMKSQFNSAGAGQFGSGWAWLIVNGN-GELEITATPNQDNPLMDRGTPILGNDVWEHAYYVTYRNRRADYLDAWWQVVNWDVASERYDAAVA

>A0A1Y2K534_1434232_Proteobacteria_B_Mar

MAFELPALPYAKDALEPHISKETLEYHYGKHHQTYVTNLNGLIEGG-PDADKSLEEIIHSA-------QGGLFNNAAQVWNHTFYWNCLSPHGGGAPGGALADAINAKWGSFDAFKEAFTKCAVTTFGSGWAWLVKKPD-GTLDLLSTSNACCPLTGDAKPLLTCDVWEHAYYVDYRNARPKYVEAFWNVVNWAFVADNFNG---

>A0A2P2EAB2_1445552_Proteobacteria_B_Mar

PAFVQAALPYGFEALEPVIDTATMRLHYERHHGAQVAGLNTAVAANPTYAGKSLEALLAGASTAP----AAIRNNAGGHWNHSFYWQIMAPVGGGAPSPELAAAITRDFGSLDAFKAQFRAASMGRFGSGWAWVVVGKD-GKLAIGSTPNQDNPLMDRGKPILGNDVWEHAYYLKYQNKRADYVDAWWSLINWNKVSSLYAEAIK

>A0A0R2HP56_1449336_Firmicutes_B_Ter

MTYQLPELPYAYDALTPYIDEETMHLHHDKHHNTYVTNLNAAIEKHPELGSKTIEELMSNLDAIPEDIRTAVRNNGGGHVNHTFFWEILAPNAGGVPTGEVKEAIDATFGSFDKFKEEFAAAATTRFGSGWAWLVLD-G-GKLSIISTPNQDSPLSEGKTPILGLDVWEHAYYLNYKNVRPEYIKAFWNIVNWDEVAKRYAAAK-

>A0A085L1P1_1453500_Bacteroidetes_B_Mar

MAFTLPSLPYDKAALEPHIDAMTMEIHHGKHHAAYVNNLNAAIQG-TEYENKSIEELMSMVSKLS----VAVRNNGGGHYNHTLFWTIMAPNGGGTPSGELAAAIDSQFGSFDKMKEDFNKAAATRFGSGWAWLCVGAD-KKLFITSTPNQDNPLMDKGTPILGLDVWEHAYYLKYQNRRPEYIAAFWNVVNWPEVARRYQEAVK

>A0A080M9P3_1453999_Proteobacteria_B_Mar

MEHQLPPLPFAIDALAPHMSQETLEYHYGKHHQAYVTNLNNLIKGS-EYEALDLEAIVRKAP------AGGIYNNSAQVWNHTFFWNCLAPNGGGAPTGALAAAIDAKWGSFADFAKAFQASAVGNFGSGWTWLVRKAD-GSVDIVNMGAAGTPLTTGDQALLCVDVWEHAYYIDYRNLRPKFVETFLSLANWRFAEQNFVG---

>A0A011NKF6_1454000_Proteobacteria_B_Mar

MEHKLPPLPFAIDALAPHMSQETLEYHHGKHHQAYVTNLNNLIKGS-EYETLDLEAIVRKAP------AGGIYNNSAQVWNHSFFWNCLRANGGGAPGGALAAAIDARWGSYAEFAKTFQASAVGNFGSGWTWLVRKPD-GTVDIVNMGAAGTPLTTADKALLCIDVWEHAYYIDYRNLRPKFVETFLNLVNWSFAEQNFAA---

>A0A011MHB8_1454001_Proteobacteria_B_Mar

MEHKLPPLPFAMDALAPHMSQETLEYHYGKHHQAYVTNLNNLIKGT-EYEELDLEAIVRKAP------AGGIYNNSAQVWNHSFFWNCLRAGGGGAPGGALAAAIDARWGSFAEFAKAFQASAVGNFGSAWTWLVRKPD-GTVDIVNTGAAGTPLTTADKALLCIDVWEHAYYIDYRNLRPKFVETFLNLVNWHFAEQNYAA---

>A0A011QCI9_1454004_Proteobacteria_B_Mar

MEHQLPQLPFAIDALAPHMSQETLEYHHGKHHQAYVTNLNNLIKGS-EYEALDLEAIVKKAP------AGGIYNNSAQVWNHTFFWNCLKPAGGGAPGGALAAAIDAKWGSFAEFAKAFQASAVGNFGSGWTWLVKKAD-GSVDIVNMGAAGTPLTTGDTALLCIDVWEHAYYVDYRNQRPKFVETFLQLANWTFAEKNFAG---

>A0A4D6HF93_1457250_Halobacteria_A_Arc

SEPELPPLPYDYDALEPHISEQVLTWHHDTHHQGYVNGLESAEATLAENRESGDF---GSSA----AAIGNVTHNGCGHYLHTLFWDNMDPNGGGEPSGDLADRIEEDFGSYEGWKGEFKAAAG--AAGGWALLVYDPVAKQLRNVKVDKHDQGALWGAHPILALDVWEHSYYYDYGPDRGSFIDAFFEVVDWDNVAEQYEKTVG

>X5MEY9_1458461_Proteobacteria_B_Mar

MTITLPDLPYAHDALEPHMSKTTFEFHHDKHHQKYVDTLNGLIDGT-NLEGKSLEDIIAAAKG--DDSKKKLFNQAAQVWNHTFFWNSMSPDGGGEPVGKLADAIKRGFGSFDDFKTQFSEAAAGEFGSGWAWLVSDKD-GKLSVMSTHDADLPLAHGAIALLTIDVWEHAYYLDYQNKRPDYISTYLKLVNWEFAAENYAAIS-

>A0A4R1R4E5_1469948_Firmicutes_B_Ter

--YKQYQLTYDFNSLEPYIDALTMETHYSKHHAAYTKNLNDAVEK-AGIENKSIETLLASLGKIGDDLRTAIRNNGGGFYNHNLYFSTMSPNGGGEPEGDLAKAIADSFENFSLFKEKLTALALGQFGSGWAWLSAGPN-GELKLSASPNQDNPIIE-YIPILGIDVWEHAYYLKYKNIRADYVKEYFNVIDWKSVAEYYKRARD

>A0A031IK26_1470592_Firmicutes_B_Ter

SKFELPELGYAYDALEPHIDARTMEIHHTKHHNTYVTNVNTALEG-TEHEGKSLEELLQNLDALPANIQTAVRNNGGGHWNHSFFWKLLKKNDGAAPTGELATAIDEAFGSFDAFKDAFAKAATTRFGSGWAWLIVD-G-GKLAVVSTPNQDTPVMEGKTPILGLDVWEHAYYLNYQNRRPDYINAFFNVVDWDHVAKLYADAK-

>A0A366ENT2_1473586_Proteobacteria_B_Mar

MTFSLPELPYAYDALGPYMSRETLEYHHDKHHLAYVNNGNNAIKGT-EWEGKPIEEIIKGSFG----KTPAVFNNIGQHYNHSLYWLSMKPNGGGAIPGELEKALVESFGSVDKAKEDLAQAAVTQFGSGWAWLSLK-D-GKLEITKTGNAESPLVHGAQPLITVDVWEHAYYIDYRNRRPDFVKAVLNLINWEFAAEQFAKA--

>A0A061Q889_1492281_Proteobacteria_B_Mar

--FELPKLPYEKNALEPHMSAQTLDFHHGKHHAKYVDVANDLVKGT-AHEGQSLEQVMVESFG----KNDKLFNNVGQIYNHNEFWKCMKPNGGGAVPEALKAKLVEAFGSVEAFAEEFVAAGTGQFGSGWVWLVVK-D-GKLAIMKTANAENPVVHGATPLLVADVWEHAYYLDYQNRRPDFLKTFIKLVNWDYVNERLSKA--

>A0A086D0E9_1492922_Proteobacteria_B_Mar

MAHSLPSLPYAYDALEPKIDAMTMEIHHSRHHQTYVNNLNAALEG-TGLEDVPVDKLMADIDKVPQDKRQAVINNGGGHANHSLFWTVMSKDGGGAPTGKVAEAIDSELGGYANFQEEFKKAATGRFGSGWAWLVVTPE-KKLKVINTLNQDSPLMDGLTPILGLDVWEHAYYLKYQNKRPDYIAAFFDVINWDEVNRRYAEATA

>A0A063ZJ32_1495067_Halobacteria_A_Arc

TDHELPPLPYDYDALEPALSEQVLTWHHDTHHQGYVNGLNSAEETLAENRENGDF---DSTP----GALSNVTHNGCGHYLHTLFWENMAPDGGGEPSGELADRIEEDFGSYEAWKGEFEAAAG--AAGGWALLVYDPVSKQLRNIAVDKHDQGALWGSHPILALDVWEHSYYYDYGPDRGSFIDSFFDVVNWDKAAEEYQTCLD

>A0A091FCF4_1499107_Proteobacteria_B_Mar

MAISLPELPYGKDALAPFITANTLDFHYGKHHKAYVDNLNKLIAGT-GLEQKSLEEIIKIAAK--DPAKAGIFNNAAQIWNHSFYWQCLKKAGGGVATGAIAAKINAVWGNYDKFAEELKNAGVTQFGSGWAWLVLE-G-DQLKITKTANADTPMAHGQKALLTIDVWEHAYYLDYQNRRPDYLAAVINLINWDFVNANLS----

>A0A090FD11_1505946_Proteobacteria_B_Mar

MAFELPALPYDYEALQPYMSKETLEYHHDKHHKAYVDNGNKLAAEA-GMGDLSVEEVVKQSFG----KNAGLFNNAAQHYNHIHFWKWMKKGGGGKLPGALQKAFDSDLGGYDKFKADFTAAGTTQFGSGWAWVSVK-N-GKLEISKTPNGENPLVHGGSPILGVDVWEHSYYIDYRNARPKYLEAFVSLINWDYVLELYEKAKG

>A0A2H3KUE0_1506545_Chloroflexi_B_Ter

MPFELPALPYAYTALEPHIDEATMHFHHDNHHNTYVTNLNNALANYPDLQDKSLEALIGDLDALPEAIRMVVRNNGGGHWNHALFWETMGPNAGGEPTGALGTAIAAAFGSFAEFKEKFKAAAIGRFGSGWAWLVVNPD-GTVAITSTPNQDTPVMEGKKAILGLDVWEHAYYLKYQYKRAGYVDAWWNVVNWAKVAELYDAARA

>A0A1Y6CDA5_1513793_Proteobacteria_B_Mar

MAFTLPELPYPKDALSPHISAETLDFHHGKHHNAYVTKLNGLVDGT-PNAEKSLEDIIKSS-------EGGLFNNAAQVWNHTFYWNCLSPNGGGEPSGAVADAINKNFGSFAEFKEKFTNAALTQFGSGWAWLVKKSD-GGLDIMQTANAGCPITENVTPLLTCDVWEHAYYVDYRNARPKYVDAFWSLVNWDFVASQMS----

>A0A094J7J3_1517416_Proteobacteria_B_Mar

MAFELPALPYEKNALEPHISAETLEYHYGKHHNAYVTKLNDAVKGT-DMESKSLEEIIKTS-------SGGVFNNAAQVWNHTFYWHCLSPNGGGEPTGALADAINSAFGSFDKFKEEFNAQAAGNFGSGWTWLVKKAD-GSVAIVNTSNAETPLTDSVTPILTVDVWEHAYYIDYRNARPNYLSAFWNLVNWEFVAKNFA----

>A0A1I5SFS4_1523247_Actinobacteria_B_Ter

PEYVLPDLPYDYGALEPHISGEIMQLHHDKHHQTYVTGANTALEKLAEARANDSL---GTAN----LHEKNLAFNLGGHVNHSVFWPNMSPDGGERPDGELAAAIDDQFGSFEAFQAHFTAVAGGVQGSGWSILAWDSVGQKLLICQLHDHQGNLTVGLVPLLMLDMWEHAFYLQYRNVKNDYVAAWWNVVNWADVTTRFTTART

>A0A0N0JCV7_1523424_Proteobacteria_B_Mar

MEHTLPALPYAIDALAPHYSQETLEFHHGKHHNAYVVNLNNLQKGT-EFESLSLEEIIKKS-------SGGIYNNAAQIWNHTFFWNCMKPAGGGEPTGALAAAINAKWGSYAAFREAFVKSAVGNFGSGWTWLVKKAD-GSVDIVNTGAAGTPLTTADKALLTVDVWEHAYYIDYRNMRPKFVETFLKLVNWGFAEANFA----

>A0A0N1LB93_1523428_Proteobacteria_B_Mar

MEHKLPELPYAMDALAPHISKETLEFHYGKHHQTYVTNLNNLIKGT-EFESMSLEDIVRKS-------SGGMFNNAAQIWNHTFYWFGFKPNGGGDATGAVAAAINAKWGSFAAFKEAFNKSALGNFGSGWTWLVKKAD-GTLDIVNTSNAATPLTTSDKPLLTCDVWEHAYYIDFRNRRADYLGSFWSLVNWDFVNQQFA----

>A0A1E8CFB9_1524254_Proteobacteria_B_Mar

MKLELPDLPYAANALEPHMSAETLGFHHGKHHNTYVVKGNELLADA-GVDADNLEDLVRATAK----VGGPLFNNVAQVYNHNFFWQSMKPSGGGMPSGAIADKINADFGSYDDFKKEFSAGGVGQFGSGWVWLVLD-G-GKLKIAKTPNAETPLTTSAKPILVCDVWEHAYYLDYQNRRPDFLAAFLNLVNWDFANQNLA----

>A0A098SDX5_1524460_Bacteroidetes_B_Mar

MAFELPKLPYAYDALEPHIDARTMEIHHTKHHAGYTSKLNAAIEG-TDLEKKSIKEILAHVSKHS----AGVRNNGGGFYNHKLFWEVMSPDGGGEPSERIHKAIMRDFGSFEKFKDEFANAAATQFGSGWAWLCVDRD-DKLFVTSTPNQDNPLMDDGTPILGLDVWEHAYYLNYQNRRPDYIEAFFNVIDWGKVSENYNDANE

>A0A1H4F8V2_152573_Proteobacteria_B_Mar

MAFELPALPYAKNALAPHISEETLEYHYGKHHNTYVVKLNGLIEGT-DFAGKSLEEIVRTS-------EGGVFNNAAQVWNHTFYWHCLSPNGGGEPTGALADAINAKWGSFADFKAAFNDKAVNNFGSSWTWLVKKAD-GSLDIVNTSNADTPLAHGLTPVLTVDLWEHAYYIDYRNARPTYLDAFWALVNWDFASKNLAA---

>A0A077FIP1_1528098_Proteobacteria_B_Mar

MTFVLPNLPYNNNALEPYITFNTLNFHHGKHHNTYVTNLNNLIKDS-ELSSKSLEEIILSTAK--DTSKSGIFNNAAQVWNHTFYWHSMKASGGGMPSPTLLDKINEDFGSFDKFKEEFKNAGTTQFGSGWSWLVLE-G-NQLKITKTGNADLPLIHNQVALLTCDVWEHAYYLDYQNRRPDYITTFLKLVNWEFVEENFLNSQK

>A0A0M5KZK2_1528099_Actinobacteria_B_Ter

QKYVLPELDYEYSALEPFISGEIMELHHAKHHAGYVSGANAALEALDEARKAGTA---EKVA----TISKNLAFNLGGHINHSLFWKNLAPQQDARPEGELAAAIDEEFGSFEQFQKEFSASATSIQGSGWAVLAWDMLSQRLVILQVTNHQDNHALTLIPLLLLDMWEHAFYLQYKNVKADYVKAFWNVINWEEVQKRFAGTAK

>A0A3N5AAM9_154117_Actinobacteria_B_Ter

AVYTLPELPYDYSALEPHISGKIMELHHDKHHATYVDGANAALDKLAAARESGDL---AAVN----LYEKNLAFNLGGHINHTIFWNNLSPEGGGEPDGEVAEAIKDSFGSFEGFKSHFTANATSIQGSGWSVLAYDSVSGRLVIFQMFDQQNNVPVGTLPLLQLDMWEHAFYLDYLNVKADYVKAFWNIVNWQDVAARLSRATT

>A0A0F7K213_1543721_Proteobacteria_B_Mar

MAHELPPLPYAQDALEPVISKETLEYHYGKHHQTYVTNLNNLVKGT-DLENASLEEIIMKA-------SGGQFNNAAQVWNHTFYWNCLCP-GGSAPSSQLSDAINSAFGSMDEFKKQFSASAAGNFGSGWTWLVKNSD-GGLEIVNTSNAANPMTDGKTPLLTIDVWEHAYYIDYRNARPKYLEAVWNIVNWEYVSANFAG---

>A0A345E419_1547899_Halobacteria_A_Arc

MSYELDPLPYDYDALEPHISEQVLTWHHDTHHQGYVNGWNSADETLEANREDGDF---GSSA----GAIRNVTHNGCGHILHDLFWNCMSPEGGDEPSGDLADRIEEDFGSYDAWKGEFEAAAG--AASGWALLVYDSFSNQLRNVVVDKHDQGALWGSHPILALDVWEHSYYHDYGPARGDFVDNYFEVVDWSEPSARYEQAVE

>A0A193LCR6_1548547_Proteobacteria_B_Mar

MKFEMKPLPYAYDALQPHISEQTVKFHYDKHHTGYMTKLQGQLAGT-PDEQKSLQEIVETS-------SGGVFNLAAQIWNHDFYWQSLTPNGGGAPGGELAEMIAAEFGDFATMRGKLKDEALGHFGSGWAWLVLGKD-NKLQICSTHDADNPLRAGATPLLTVDVWEHAYYLDTQNDRGGYLDRVLNLLNWQFAEDNLSAARK

>A0A2Z4FGN2_1548548_Proteobacteria_B_Mar

GEHKLPELPYAYDALEPFIDEQTMRLHHDIHHNGYVNGLNKAEADLAAARESGDF---SKIA----DIQRRLAFHGSGHVNHTLFWRNMCPPGDKDPSGDLAKQIEKDFGSLENLKAQFAAAAKTVEGNGWGFLVWSPEGGYLTTVAAENHQKNFVNNQIPLLVLDVWEHAYYLKYQNKRGDYVENFFKIVNWENVAQNFANAKA

>A0A0M2R958_1549748_Proteobacteria_B_Mar

MALELPALPYAYDALGPYMSAETLEFHHDKHHNAYVVNGNKLLEGS-DLEGKSLEDIVKGSFG--DASKAGIFNNAAQHWNHIEFWNMMKPNGGGAIPGELEKKIIEDFGSVDQFKADFVNAGVTQFGSGWCWLVLDDS-GKLAVTKTANGENPLVTGGHALLGCDVWEHSYYIDYRNARPDYVKAFVSLVNWEYVAERFSKAG-

>A0A498GYD6_1550565_Methanomicrobia_A_Mar

KKYELPPLPYEANALEPYISQEQLSLHHDKHHQAYVKGANADLEKIEQARRDNAS---IDEK----AILKELSFNIGGHILHSLFWPTMAPAGGGTPGGALADALDREFGSFERFKSEFSQAASSVEGSGWAALAIDEITGRPMVMQIEKHSNNVYPSAPIIMVLDMWEHAYYVDYRNSRADFIDAFWNVVNWDEVNRRIEQRV-

>A0A099W947_1552123_Firmicutes_B_Ter

MTYELPKLAYTYDALEPNFDKETMEIHYSKHHNTYVTKLNEALEGHDDLAAKSIEDLVADLNSVPENIRTAVRNNGGGHANHSFFWTILSPQGGGEPTGALADAINAEFGSFEEFKTQFANSGVARFGSGWAWLVVK-D-GKLSIISTPNQDSPLTEGYTPVIGLDVWEHAYYLKFQNLRPKYIETFWNVVDWDKAAEYFNAAK-

>A0A0K2SFV8_1555112_Firmicutes_B_Ter

MAHELPPLPYGFDALEPYIDAQTMQIHHDKHHGTYVNNLNAALESADALRALGVEQLLQRINEVPESIRTAVRNNGGGHANHSMFWEIMAPNAGGAPSGELGSAITSAFGSFDSFKETFAKAALGRFGSGWAWLVLK-G-GRLEVSSTANQDSPYMEGQVPILGLDVWEHAYYLKYQNRRPEYVQAWWNVVNWPEVNQRFLKARS

>A0A2G3K1V1_1559339_Proteobacteria_B_Mar

MEHKLPELPYAIDALAPHMSKETLEYHYGKHHQTYITNLNNLIKGT-EHENASLEEIVKKAP------AGGLFNNAAQVWNHTFFWMGFKPNAGDAPTGKLLAAITAKWGTVDEFKKAFNTSAAGNFGSGWTWLVKKPD-GSLDIVNTSAAATPLTTADTALLTADVWEHAYYIDYRNSRPNYLEAFWKLVDWDAVAARLG----

>Q26GK1_156586_Bacteroidetes_B_Mar

MAFELPKLNYAYDALEPHIDARTMEIHHSKHHNGYTTKLNNAIEG-TDLEGKSIEDILANLDMDN----KAVRNNGGGFYNHRLFWEVMSPNGGGKPTGDLAAAIDSAFGSYDAFQDKFATAAKTQFGSGWAFLCVK-D-GALEVCGTPNQDNPLMPGGTPILGLDVWEHAYYLNYQNRRPDYVSAFFNVIDWDAVSEKYNAAK-

>A0A251X438_1570016_Proteobacteria_B_Mar

MAHQLPALPYAMDALAPHISKETLEFHYGKHHQTYVDKLNALIPNT-EFTDLSLEEIVKKS-------SGGIFNNAAQVWNHTFYWNCLSPNGGGAPTGAIAEAINAKFGSFDKFKEEFTATAINTFGSGWGWLVKNPD-GSLALMSTSNAGTPMTEGKTALLTCDVWEHAYYVDYRNARPKYVEAFWNLVNWDFVNANLAA---

>A0A0K0XVJ8_1579979_Proteobacteria_B_Mar

MAFTLPELPYAKDALEPHISAETLEYHYGKHHQTYVDKLNGLVEGT-ADADKSLEDIIRSS-------SGGLFNNAAQVWNHTFYWHCLAPNGGGEPGGALAELIDRDFGSFAAFKEQFTQTAINTFGSGWAWLVQNPD-GKLAITSTSNAETPLTGSAKPLLTCDVWEHAYYIDYRNARPKYMDAFWNLVNWDHVAAQLS----

>A0A0N1G8F9_1592327_Actinobacteria_B_Ter

PVRTLPDLPYDYSALAPVISPEIVELHHDKHHAAYVKGANDTLEQLAEARDKESW---GSVN----GLEKNLAFHLSGHVLHSIYWHNMTGEGGGEPVGELADAITESFGSFAAFRAQLSKAAATTQGSGWGVLAYEPLSDRLIVEQVYDHQGNVGQGATPILVFDAWEHAFYLQYRNQKVDFVEAMWQVVNWQDVARRYEAARS

>A0A0N1H7L0_1592329_Actinobacteria_B_Ter

PVYTLPELPYDYSALAPVISPEIIELHHDKHHAAYVKGANDTLEQLAEARDKEQW---AALN----GLEKNLAFHLSGHILHSIYWANMTGDGGGEPVGDLADAITESFGSFAGFKAQLTKASATTQGSGWGVLAYEPLSGRLIVEQVYDHQGNVGQGSVPVLVFDAWEHAFYLQYKNQKVDFIEAMWAVVNWQDVARRHEAAKS

>A0A0M4CZS5_1603606_Proteobacteria_B_Mar

MTIALPELPFPIAALEPHISARTFEFHHGKHHKAYVDNTNKLIEGT-PLAGKDLESIILAAAG--DQAKKGLFNNAAQVWNHSFFWKCLKPGGGGKPTGKVAARIDADLGGYEKFAADFKNAGATQFGSGWAWLVLK-D-GKLEIAQTANAETPLTKGHKPLLVVDVWEHGYYLDYQNRRPDFLQAFLHLVNWDFVNANLG----

>A0A0F7PDN8_1604004_Halobacteria_A_Arc

SDYELPPLPYDYDALEPHISEQVLTWHHDTHHQGYVNGWNNAEETLAENREAGTT---DDSA----AAIRNVTHNGSGHILHTLFWNNMSPNGGGEPEGDLRARIEEDFGSFEAWQDEFEAAAG--AASGWALLIYDTHAERLRNVVVDKHDQGALWGSHPILALDVWEHSYYFDYGPDRGSFIDAFFEVVDWDDVAENYEDVIA

>A0A2V3PRT9_1605892_Bacteroidetes_B_Mar

MKFEMPKLPYATNALEPVISQQTVELHYGKHLQTYVNNLNNLVPGT-KFENSDLITIIKES-------DGAIFNNAGQILNHDLYFASFTPGTGGSPKGKLAEAITAKFGSFENFQKEFNTAGAGLFGSGWVWLAKDAS-GNLSIEKEPNGSNPVVKGLTPILGIDVWEHAYYLDYQNRRADHLAEVWKIINWDVVSARY-----

>A0A1B4XH23_1620215_Proteobacteria_B_Mar

YPFALAPLPFAYDALEPYIDAATMRIHHDKHHQTYITRLNAALETYPEWHGLSIEKLLLERSELPIAIRQTVHDQGGGHLHHQLFWEILKPGAGDRPTRALAQAIDRSFGSFDAFKARFVDTGARHFASGWVFLLVNPADGKLEILSCHDHDSAVLENKTALLLNDLWEHAYYLKYQSGRVDYLKVFWNVVNWEHVGHRLESALA

>A0A2T0ZW47_1629062_Actinobacteria_B_Ter

AVYSLPDLAYDYGALEPAMTGEILELHHSKHHAAYVKGANDTLEQLEEAADKATP---TQTV----GLEKTLAFNISGHVLHSIFWENLSPEGGGRPEGELASAIDEYFGSFEKFHSRLSTATAAVQGSGWGVLGYEPLSKRLIIEQVYDHHGNVGQGTTPLLAFDAWEHAYYLQYRNVRPDYVKRLWDLVNWSDVAKRFDKARK

>A0A142X6W1_1632864_Planctomycetes_B_Mar

MAYTLPALPYAYNALEPHIDARTMEIHHTKHHQAYINNVNKALEGHADLAALPVDDLMKKLSSVPEAIRTVVRNNGGGHSNHTLFWTVLAPNAGGAPNGAVASAIDGAFGNFDTFKTQFNDAATKRFGSGWAWLSVD-K-GKLVVESTANQDTPLSEGRTPILGLDVWEHAYYLNYQNRRPDYITAFWNVVNWAEVNKRLAAAK-

>A0A0K0GDK0_1637974_Firmicutes_B_Ter

AKFELPELPYAYDALEPTIDKETMNIHHTKHHNTYVTNLNAAVEGISELEGKSVEELISNLDAVPEAKRTAVRNNGGGHANHSLFWKVLSPNGGGEPTGELAEKINAKFGSFDKFKEEFENAAKTRFGSGWAWLVSN-N-GELEVMSTPNQDSPLMEGKTPLLGVDVWEHAYYLKYQNRRPEYLAAFWNVVNWDEVAKNYKG---

>A0A0H0ZNJ8_1652545_Actinobacteria_B_Ter

AVYSLPELQYDYAALEPNISARIMELHHSKHHATYVAGANSALEQLAEAREKGEF---GNIP----KLSKDLAFHLGGHTNHSIFWNNLSPEGGDKPEGELAAAIDDFFGSFDAFRGHFTASAMSLQGSGWAVLAYDAIGGQLVIEQLYDQQGNVPVATIPLLMLDMWEHAFYLDYVNVKADYVKAFWNIVNWADVQARFEAARN

>A0A1E2ZEY7_1655433_Proteobacteria_B_Mar

MAHELPALPYAIDALEPVISKETLEFHHGKHHNTYVTNLNNLIPGT-EFENASLEDTIMKS-------SGGVFNNAAQIWNHTFYWNCLSPNDDNAPGGALAGAIDAAFGSFDEFKKQFATSGATNFGSGWTWLVQNDD-GSLEIFNTSNAGTPMTSGKKALLTADVWEHAYYIDYRNARPAYLEAFWKVVNWDFVASNMS----

>A0A192WU48_1660251_Acidobacteria_B_Mar

MAHELPSLPYAFDALEPHIDARTMEIHHGKHHAAYVSKLNAALEGHDGLAAKSIEDLMASLDEVPEDIRGAVRNNGGGHCNHSLFWKIMKPGGGGEPGGELADAINSVFGSFADMKKTLINVGVGQFGSGWAWLGIK-D-GKLHAGGTANQDSPLMFGGTPILGVDVWEHAYYLNYQNRRPDYLEAWFNTVNWDEVAANLKNA--

>A0A348B0W0_1670455_Thermoprotei_A_Arc

RKYELPQLPYKLDALEPYISKDIMDVHYNGHHKGYVNGANSLVDRLEKVVKGEVTS--YDVQ----GIVRGLAFNINGHKLHDLFWKNMAPAGGGKPGGALADLIQKQYGGFDRFKQVFTEVANSLPGTGWTVLYYDTENGNLQLMTFENHFMNHIAELPIIMIVDEFEHAYYLQYKNKRADYVSAWWNVVNWDYAEEKLKKYMK

>A0A3N6M5P3_1679083_Halobacteria_A_Arc

TDHELPPLPYDYDALEPAISEQVVTWHHDTHHQGYVNGLNSAEETLAENRESGDF---DSTP----GALGNVTHNGCGHYLHTLFWENMSPDGGGEPDGDLADRIEEDFGSYEGWKGEFEAAAG--AAGGWALLVYDPVAKQLRNVAVDKHDQGALWGSHPILALDVWEHSYYYDYGPDRGSFIDGFFDVVNWDSVADEYQKCLD

>A0A3N6LM69_1679091_Halobacteria_A_Arc

TDHELPPLPYDYDALEPSISEQVVTWHHDTHHQGYVNGLNSAEETLAENRESGEF---GSTP----GALGNVTHNGCGHYLHTLFWENMAPNGGGEPDGDLADRIEEDFGSYEGWKGEFEAAAG--AAGGWALLVYDPVAKQLRNVAVDKHDQGALWGSHPILALDVWEHSYYYDYGPDRGSFIDGFFDVVNWDSVDEEYQKCLD

>A0A2R4X0R1_1679096_Halobacteria_A_Arc

SHPELPDLPYAYDALAPSISEQVLTWHHDTHHQGYVNGLDAAEQTLAENRANGDF---ASSG----AALRSVTHNGCGAYLHELFWTNMSPNGGGEPSGSLRERIEEDFGSYEGWKGEFEAAAS--AAGGWALLVYDSKAGQLRNVVVDKHDQGALWGAHPILALDVWEHSYYHDYGPARGDFVDAFFDVVDWDAVAEEYSSVVG

>A0A139SPB8_1680762_Proteobacteria_B_Mar

MAFVLPPLPYEKNALEPHISSETIEYHYGKHHNTYVVNLNNLVPGT-EFEGKSLEDIIKTS-------SGGIFNNAAQIWNHSFYWECLSPSGGGEPGGELGKAIDTAFGSFAKFKEEFTKTAIGTFGSGWAWLVKKAD-GSLALASTIGAGCPLTSGDTPLLTCDVWEHAYYIDYRNLRPKYVEAFWNLVNWDFVAKNYAA---

>A0A2N7UR03_1684789_Proteobacteria_B_Mar

MAHTLPELPYAYNALEPHIDATTMEIHHSRHHQTYVNNLNGALEG-TGLEDVPVDELVANLDRVPEEKRQAVINNGGGHSNHSMFWQVMSPNGGGAPKGKVGEAIDSELGGFDDFKDAFVKAALGRFGSGWAWLSVTPE-KKLVVENTLNQDNPLMHGNTPVLGLDVWEHAYYLKYQNKRPDYVAAFFNVVDWDEVERRYQAAVA

>A0A0X3T641_1685380_Proteobacteria_B_Mar

MAFTLPDLPYDYNALDAAIDAQTMEIHHTKHHQTYVTKLNDAIKDSPN-ADKSLDELIANAGSIS----PAVRNNGGGHWNHSFFWEIMTGDSMGAPSGALADDIKATFGSFEDMKAQFNNAGATQFGSGWAWLTVSAE-GKLEISSTPNQDNPLMDKGTPILGLDVWEHAYYLRYQNRRPDYMQAWWDVVNWNKVEALYNAAK-

>A0A142LJU4_1690485_Proteobacteria_B_Mar

TVFAQVALPYAKDALVPHVSAETIDFHYGKHHLTYVTNLNNLAKGT-EFENLTLEEVVKKA------PAGAIFNNAAQIWNHDFYFLGFKPGGGGKPSGALAAAIDKQFGSFEEFQKQFDAKAAGTFGSGWAWLCKKAD-GSLSLESTSNAATPLTQGMTPLLTCDVWEHAYYIDYRNSRPNYLKGFWAIVNWDFVAANFAK---

>A0A0Q4BCN0_1713724_Thermoplasmata_A_Arc

GFYSLPELKYGYGDLAPFISEQLLKIHHDGHHQKYVNQANALLEKLDKARQDGNM---PPMK----CDVQALSFNVGGHYLHSLFWDNMAPAGGGKPGGLIADLLDKEFGSFDRFKKEFTEVANSVESSGWAVLVMCLQTGRPLLVQVKDHHLYSIPGFRILMVLDVWEHAYYLDYRNEKARYNQAFWDVVNWDKVDERAENIVG

>A0A2A9DLZ4_1724_Actinobacteria_B_Ter

AVYTLPELDYAYDALEPHISAEIMEIHHTKHHQTYVDGANATLEALEKAREEGDQ---NQIR----ALSKNLAFNLGGHTNHSIFWKNLSPNGGGEPTGELAEAINRDFGSFEKFKDHFSNAALSLQGSGWAVLGYDHVAGRLVIEQMTDQAGNTSINLTPILMLDMWEHAFYLQYKNVKADYVKAVWNVFNWDDVAERYAAAAK

>A0A0Q5R2U1_1736349_Actinobacteria_B_Ter

SEYTLPDLDYDYAALEPHISGRIMELHHSKHHATYVKGANTALEKAAAAREDGTI---AKVF----QLSANLAFHLGGHTNHSIFWKNLSPNGGDKPEGDLAAAIDDQFGGFDKFQAHFTAAATTLQGSGWAILGFDTIEGKLVIQQLTDQHGNTSVGLIPIVMLDMWEHAFYLDYQNVKPDYVKAWWNVVNWADAGERFAKAQS

>A0A0Q6BJ75_1736373_Proteobacteria_B_Mar

MEHTLPPLPFAKDALAPHMSEETFEYHYGKHHQAYVTNLNNLIKGT-EFENLSLEDIIKKS-------SGGIYNNSAQIWNHTFFWNCLTPNGGGEPTGALADAIKAKWGSYEEFKKAFQTSAVGNFGSGWTWLVKKAD-GSVDIVNMGAAGTPLTTGDKALLTIDVWEHAYYIDHRNARPKFVETFLNLVNWEFAAKNFAA---

>A0A3E1EU86_1737063_Bacteroidetes_B_Mar

MAFELPKLPYAHDALEPNIDAKTMEIHHGKHHQGYTTKLNAAIEG-TDLEGKSIEEILK-AGKDK----AAVRNNGGGYYNHNLFWTVMSPNGGGNPIGDIKDAIDSAFGSFDAFKDEFSKAAATQFGSGWAWLCVK-D-GKLEVCSTPNQDNPIM-EGTPVLGLDVWEHAYYLNYQNRRPDYIEAFFNVINWEEVNRRYNEAK-

>A0A2W1JHI3_1764569_Cyanobacteria_B_Ter

EEFVLPPLPYAYDALSAYVDEQTMMIHHDKHHAGYVKNLNDAIAKHPDLKGKSVEALLQDLDNIPEDIRVTVRNNGGGHANHTMFWETMTPDSKGQPTGKLAKAINSSFGSFDAFKEAFNTAGTKQFGSGWAWLVLTPD-NKLEVTSTANQDSPLLDGNTPIMGNDVWEHAYYLKYQNKRGDYLNAWWNVVNWDQVGQRYEQALS

>A0A0U3DNG9_1768242_Proteobacteria_B_Mar

MEHLLPALPYAKDALAPHYSAETLEFHHGKHHNAYVVNLNNLQKGT-EFEALSLEEIVKKA-------SGPIYNNAAQIWNHTFFWNCMKPNGGGAPTGALADAINAKFGSFEAFKEAFTKSAVGNFGSGWTWLVKKAD-GTVDIVNTGAAGTPLTTADKALLTIDVWEHAYYIDYRNLRPKFVETFLSLANWEFAQANFA----

>A0A1E3VXW9_1774968_Proteobacteria_B_Mar

MAFELAPLPYAYDSLQPYMSAETLEYHHDKHHKAYVDNLNKLIEGT-DYEGKDLETIIKESFG----KDAGVFNNAAQNFNHIHFWPWMTKDGGGKVPGAILALIDRDLGGFDKFRADFLAAGATQFGSGWAWLTLK-D-GKLEVTKTPNGENPLVHGGWPLLGCDVWEHSYYIDYRNARPKYLEAWFNLINWDHVEELYAQAPG

>O93724_178306_Thermoprotei_A_Arc

KRYTLPPLPYAYNALEPYISAEIMQLHHQKHHQGYVNGANAALEKLEKFRKGEAQ---IDIR----AVLRDLSFHLNGHILHSIFWPNMAPPGGGKPGGKIADLINKFFGSFEKFKEEFSQAAKNVEGVGWAILVYEPLEEQLLILQIEKHNLMHAADAQVLLALDVWEHAYYLQYKNDRGSYVDNWWNVVNWDDVERRLQKALN

>A0A149W187_1789004_Proteobacteria_B_Mar

VTHELMTLPFAMDALAPHISKETLEYHYGKHHRAYVNKLNELIKGT-EFEGLSLSDLVRKT-------TGPTFNNAAQVWNHNFYWYGLNPRGSDKPTGELAAAINKNFGSFDQFAAQFKTAAATKFGAGWTWLVKTED-GQLAIRNSNDAENPLQWNQIPLLTCDVWEHAYYIDYRNERPRYIETFWALVDWDFVARNLEGEGI

>A0A2X0WVT3_179995_Proteobacteria_B_Mar

MSFAITPLKFEKTAV-PFLSENTFNFHHGKHFQTYIDTANKLVAGT-DYEGKTLEEIITTA-------SGPLFNNAAQAWNHAFYFNCIGTAKKEVP-AKLLELINANFDSFESFAEKFIASATTNFGSGWTWLVQTGP-NFLKIVNTSNAGNPMVDGFTPLLTVDVWEHAYYLDYQNRRADYLKDFVEYIDWEFVASNLK----

>A0A1B1YPZ1_1810504_Proteobacteria_B_Mar

MTHELPPLPYAIDALAPHISAETLEFHHGKHHKTYVDNLNKLIPGT-EFENLSLEDIVRKS-------SGGIFNNAAQIWNHTFYWNCLAPKAGGKPTGALAAAIDTAFGSFDTFKEKFSQTAITTFGSGWGWLVKNAA-GGVELVSTSNAGCPLTAGQTPLLTCDVWEHAYYIDYRNARPKYVESFWNLVNWDFVARNFG----

>A0A1G6RCD0_1814289_Actinobacteria_B_Ter

AVYTLPELGYDYGALEPHISGRIMELHHSKHHQAYVTGANTALDKLAEARENGDF---AAIN----LHEKNLAFNLGGHVNHSAFWTNLSPAGGGEPTGDLAAAIAEDFGSFDAFKKHFGAVAAGVQGSGWAILAWDSLGSKLIIVQLYDQQGNIPMGLTPIVLLDVWEHAYYLDYQNVRADYVAAWWNIVNWADADARFTRART

>A0A1E2V0Q9_1818881_Proteobacteria_B_Mar

MAHELPALPYAIDALEPVISKETLEFHHGKHHNTYVTNLNNLIPGT-EFENASLEDIIMKS-------SGGVFNNAAQIWNHTFYWNCLSPSDDNAPTGALADAINNTFGSFDEFKKKFATSGATNFGSGWTWLVQNAD-GSLEIFNTSNAGSPMTSGKKALLTADVWEHAYYIDYRNARPAYLEAFWKVVNWDFVAGNMS----

>A0A162GML5_1822219_Proteobacteria_B_Mar

MAIELPALPYAIDALAPHISQETLEFHHGKHHKTYVDKLNGMIPGT-EYEGKTLEEIITSS-------SGPVFNNAAQIWNHTFYWNCLSPNGGGAPTGKVADAINEAFGSFEEFQAAFNDKAVNNFGSSWTWLVKNAD-GSLEIVNTSNAGTPMTEGQTALLTVDLWEHAYYIDYRNVRPTYLNAFWALVNWGFVAENLG----

>A0A378YCD3_1823_Actinobacteria_B_Ter

AEYTLPDLDYDYSALEPHISGQINELHHSKHHAAYVAGANTALEKLEEAREKGDH---GAIF----LYEKNLAFHLGGHVNHSIWWKNLSPNGGDKPVGELAAAIDDQFGSFDKFRAQFTAAANGLQGSGWAVLGYDTLGQKLLTFQLYDQQANVPLGIIPLLQVDMWEHAFYLQYKNVKADYVTAFWNVVNWEDVQERFARAVS

>A0A328VKE5_1825093_Chloroflexi_B_Ter

MAFELPPLPYSFDALEPHIDAQTMQIHHDKHHGTYVANLNSALEGHP-FANLPVEEVLRRINEVPENVRTAVRNNGGGHANHSMFWRIMKPNGGGQPSGDLAKAIEQTFGSFENFKAQFNDTGLKRFGSGWVWLVLDKQ-GKLQVISTANQDSPYMDGLYPVMGNDVWEHAYYLKYQNRRADYLNAWWNVVNWEEIERRYAEGLS

>A0A1M6L352_1830138_Firmicutes_B_Ter

MAFQLPPLPYAFNALEPHIDALTMEIHHDRHHGTYVNNLNAALEGHADLQSKSIEELLSHLDAVPENIRTAVRNNGGGHANHSMFWEILSPNGGGAPTGEIAKAIDETFGSFEKFQEEFTKAATGRFGSGWAWLVVD-G-GKLSIMSTPNQDNPMMEGKKPVLGLDVWEHAYYLKYQNKRPDYIKAFWNLINWAEVNKRFEAAK-

>A0A1P8F872_1839801_Chloroflexi_B_Ter

--------MYTANHLKG-FSETLLNNHF-TLYQGYIKNTNKLFETLDALSKAGKS-DTPEFA----ELQRRFGFEWNGMRLHELYFGNLGGDGLLAPAVKLEKKLAEAWGSREAWEKDFRAVGS-LRGVGWAVLYQDPVSGALFNCWINLHESGHLAGCNPLLVMDVWEHAFMIDYGLKRADYIDAFFKNIDWAACEKRLK----

>A0A1I3W540_1855324_Actinobacteria_B_Ter

ADYTLPDLPYDYAALEPHISGRIMELHHDKHHAAYVAGANTALEKLAAARETGDF---AAVN----LHEKNLAFNLGGHVNHSVFWENLSPDGGDKPVGELAAAIDEFFGSFEAFQKHFAANAAGIQGSGWSILGWDSVGQKLAIFQLYDQQSNYPLGLVPIVVLDMWEHAFYLDYVNVKADYIKAWWNIVNWADAAERFTRART

>A0A1I2LRE0_1855325_Bacteroidetes_B_Mar

MNIPMPVLPYAPNALEPVISEQTISFHYGKHLQGYVNTINKLVEGT-EFAGKSLEDLVRTV------PEGPMYNNAGQALNHKFYFLQFSPRKDNVPTGRIAAAINSDFGSFEAFKKQFAQAATSLFGSGWAWLSQDES-GKLVITKEANGGNPLRSGLNPLMGIDVWEHAYYLDYQNRRADHVEAVWEIIDWKVVESRLK----

>A0A1K1LIX2_1855339_Proteobacteria_B_Mar

ITHVLPPLPYADDALDPVISAHTLSFHYGKHHKAYVDNLNKLVAGT-ELADRSLEEIIAATAG--QADKAGVFNNAAQIWNHTFYWNSLKPGGGGQPPAALRERIEASFGSVDACKQELAAAATTQFGSGWAWLVRD-G-DTLKVVKTGNADVPLTKGLTPLLTLDVWEHAYYLDYQNRRADYVNAVLKLINWGFAADNHA----

>A0A143PHC2_1855912_Acidobacteria_B_Mar

MPYELPPLPYAHNALEPFIDEQTMQIHHGKHHQTYVNNVNAALEKYPELQGKPIDQLIADLNAIPEDIRTAVRNNGGGHANHTFFWAVMAPNAGGSPTGKIAEAINAKFGSFDAFKEAFAKAGTTRFGSGWAWLIKS-G-SGVEVTSTPNQDSPLMEGKMPLLGLDVWEHAYYLKYQNKRPDYIAAWWNVVNWDAVNNAF-----

>A0A1V2RP88_1857892_Actinobacteria_B_Ter

ATYTLPDLPYDYSALEPVISGEIIELHHDKHHAGYVKGANDTLEQLAQARETDQW---GTIN----GLEKNLAFHLSGHILHSIYWHNMTGDGGGEPTGELADAVAESFGSFARFKAQLSKAAATTQGSGWGVLAYEPVSGRLIVEQIYDHQGNVGQGSVPILVFDAWEHAFYLQYRNQKVDFVEAMWRVVNWQDVAARYTAAKQ

>A0A1D2W9T7_1860100_Methanobacteria_A_Arc

GFYELPELDYGYKDLEPHISEQQLKIHHQKHHQAYVDAANAIFKLYDEAREKGED---FDVK----AKAKELAFNAGGHQLHTLFWKNMGPANGGEPTGTIAEYIKKDFGSFERFKKEFSQAAITTEGSGWAVVTLCKSTDRLIILQLEKHSVNTAPRWPPLMVLDVWEHAYYLDYKNVRPDFVAAFWNIVNWDEVNTRVDAWLK

>A0A1A8XY42_1860102_Proteobacteria_B_Mar

MEHQLPQLPFALDALAPHMSRETLEYHHGKHHQAYVTNLNNLIKGT-EYEALDLESIIRKAP------AGGVYNNSAQVWNHSFFWNCLTVNGGGAPGGALAAAIDARWGSFAEFSKAFQASAVGNFGSAWTWLVKKAD-GSVDIVNMGAAGTPLTTGDQALLCIDVWEHAYYVDYRNLRPKYVETFLNLANWGFAEQNFAG---

>A0A191ZFD3_1860122_Proteobacteria_B_Mar

MAFQLPELSYDYGALDKSIDAQTMEIHYSKHHGAYVTNCNNAIAG-TEWDDKPIEDIMANVSKLT----PAVRNNGGGHFNHTLFWSILSPNGGGEPTGALADDIASTFGSFSAFRDQVNTAGATRFGSGWAWLVVTPE-GKLAVCSTPNQDNPVMDKGTPILGIDVWEHAYYLRYQNRRPDYLKAIWDVISWDAVAQRYADAK-

>A0A1D8S4L0_1873524_Halobacteria_A_Arc

SHPELPELPYDYDALEPHISEQVLEWHHDTHHQGYVNGLDSAEEALAEARETGET---HGTQ----AALRSVSHNGSGHYLHTMFWDNMSPNGGGEPEGALRERIEEDFGSYEAWRQEFEEAAG-YSAGGWALLVYDPVAKQLRNLAVQKHNDGALWGAHPILALDVWEHSYYFDYGPARGEFVDAFFEVIDWDDVAENFEKVSS

>P18868_187420_Methanobacteria_A_Arc

KFYELPELPYPYDALEPHISREQLTIHHQKHHQAYVDGANALLRKLDEARESDTD---VDIK----AALKELSFHVGGYVLHLFFWGNMGPACGGEPSGKLAEYIEKDFGSFERFRKEFSQAAISAEGSGWAVLTYCQRTDRLFIMQVEKHNVNVIPHFRILLVLDVWEHAYYIDYRNVRPDYVEAFWNIVNWKEVEKRFEDIL-

>A0A231UUB0_1876515_Proteobacteria_B_Mar

MAFSLPDLPYAYDALAPHMSAETLEYHHDKHHNAYLETMNGIIEGT-DYESMELEEIVKKSHK----DDSKLFNQSAQFYNHVHFWKWMKPEGGGQLPEKLQQAFESDLGGYDKFREDFINAGKTQFGSGWAWVFVK-D-GKLHIDNGLNGENPLIMGGTPILGVDVWEHSYYIDYRNARPKYLEAFVHLINWDYVLERYEVATS

>A0A1C0VXQ7_1880991_Cyanobacteria_B_Ter

MAFELPPLPFAADALESAMSANTFSFHHDKHHAAYVTNLNKLIEGT-ELADKSLEEIVSISFK--DPSKVGIFNNAAQVWNHTFFWSSLKPNGGGIPTGTLADKIAADFGSFDKFKDDFKAAAATQFGSGWAWLVLD-N-GALKVTKTPNAENPVVHGQIPLLTLDVWEHAYYLDFQNRRPDFIANFLNLANWDFAAENFAKAA-

>Q8TQG9_188937_Methanomicrobia_A_Arc

DLYKLPPLKYGYADLEPYISEEQLRIHHDKHHQGYVNNTNALLEMMDKARKEDTD---FDYK----ATAKALSFNLSGHVLHDFFWWEMTPAASKEPVGEFAEAIKEDFGSFERFKKEFSKVASSVEGSGWAALTFCKGTKRLGIVQIEKHNVNLVPDFPIIMDLDVWEHAYYIDYKNDRGKFIEGFWNIIDWEELDKYFKKIQK

>A0A4Q5XXF3_1913988_Proteobacteria_B_Mar

MAFTLPALPYARDALAPHISENTLNFHYGKHHQAYVDNLNKLVAGT-PLENAPLEEVVKQSWA--E-KKAPIFNNSGQVWNHTFYWHSMKPGGGGKPTGLVADRINKDFGSYDEFKRLFAEAGATQFGSGWAWLVLK-D-GKLSVTKSPNAETPLTEGVTPILTMDVWEHAYYLDTQNARPKYIETFVELVNWDFANQNLSDAK-

>A0A4Q5V9J9_1913989_Proteobacteria_B_Mar

MAFELPALPYAKDALAPHISAETLDFHHGKHHKTYVDKLNGLVPGT-EFEGKSLEEVIKSS-------SGPVFNNAAQIWNHTFYWHCLSPNGGGAPNGPVAEAINKAFGSFDKFKEDFNTSAVNNFGSSWTWLVKKAD-GSVAIVNTSNAGTPLTDSVTPILTVDLWEHAYYIDYRNARPTYLNAFWSLVNWEFVNANFAK---

>A0A1L4CZX9_1915309_Proteobacteria_B_Mar

MAKEIS------KHLLGKISDPQLEAHFGL-YEGYVKKLNEIEEKLEKTDKGLTNYSFGEFS----ELKRRHCVPYNGTYLHEMYFENLIST--ESPSPQFENLAKAAFGSVDNWKADVKATGL--AVPGWVVTCVETTTGKLKNVQIMEHHIGFPLNHVPVLVMDTWEHAFFLDFKANRGAYIDVFFKNINWSVVNARVTQCAK

>A0A1T2KSF0_1918948_Proteobacteria_B_Mar

MAHELPALPYDMNALEPTISQETLEFHYGKHHQTYATNLNNLVPGT-EFEDASLDEIVMKS-------SGGIFNNAAQVWNHTFYWNCLSPNGGGAPSGTLAVAINSAFGSFEEFKKQFAQSAATNFGSGWTWLVKNGD-GSVEIVNTSNAANPMTDGKTPLLTIDVWEHAYYIDYRNARPKYLEEIWNIVNWDFVGSNYDA---

>A0A1T2L771_1918949_Proteobacteria_B_Mar

MQHQLPELPYEKSALEPHISAETLEFHHDKHHATYVTNLNNLINGT-EFADKSLEEIVTSAP------AGGIFNNAAQVWNHTFYFNCMAPGAGGAPTGEIAAAIDAAFGSFEDFKSAFSTSAVTNFGSGWTWLVKNAE-GGVEIFNTSNAGCPLTEGLTPLLTIDVWEHAYYVDKRNARPAYVESWWTLVNWDFVNAQL-----

>A0A2K8LAI0_1921087_Proteobacteria_B_Mar

MPFELPELPYARNALEPHISAETLDYHYGKHHNAYVTNLNNLIAGT-GHENSSLEEIIRSS-------TGPLFNNAAQVWNHTFYWNCLSPQGGGEPSGELGDAINRTFGSFGAFKEKFTASAATNFGSGWTWLVKNED-GTIEIVNTSNAGNPLTNGQKPLLTCDVWEHAYYIDYRNARPSYLEAFWHLVNWNFADNNL-----

>A0A1V3TPU9_1924934_Proteobacteria_B_Mar

MAYTLPELGYAYDALEPHFDAKTMEIHHSKHHQAYINNANAALEAHPELLEQCPGKLISNLSQVPADKLAAVRNNVGGHVNHTLFWKGLKT--GTTLQGALKDAIVRDFSSVENFQAEFEKAAATRFGSGWAWLVAN-E-GKLSVVSTANQDSPLMGAGYPILGLDVWEHAYYLNYQNRRPDYIKAFWNVVNWDEAARRFEEKVA

>A0A1Q1FM06_1932360_Halobacteria_A_Arc

SNPELPPLPYDYDALEPSISEQVLEWHHDTHHQGYVNGLESAEETLAENRENGDF---GSSG----SAIRNVTHNGSGHYLHTLFWENMDPNGGGEPSGELADRIEEDFGSYEGWKGEFEAAAS--AAGGWALLVYDPVADQLRNLVVDKHDQGALWGSHPILALDVWEHSYYYDYGPDRGSFIDGFFDVVDWDNVAEQYEKALS

>A0A1V3PIV2_1945854_Proteobacteria_B_Mar

MAIELPPLPYEKNALEPHISAETLEYHYGKHHQAYVTNLNKLIEGT-EFADAALEDIIRKS-------SGGVFNNAAQIWNHTFYWNSLGPKGGKEPNGKLADAISKAFGSFEKFKEEFTKSAAGNFGSGWTWLVQRPD-GALGIVNTSNAATPITGSDKPLLNVDVWEHAYYIDYRNARPKYLEAFWNLVNWEFAAKNLA----

>A0A239QSP2_1945888_Actinobacteria_B_Ter

AIYTLPELPYDYAALEPHISATIMELHHSKHHAAYVAGANTALEQLAEARENGAF---GAIP----KLEKDLAFHVGGHVNHSIFWKNLAPNAGGEPEGEVAAAIQDSFGSFAGFKGQFTAAAMSLQGSGWAVLAYEPLSQKLVTYQLFDQQGNVPVTTLPLLQLDMWEHAFYLDYKNVKADYVTAFWNIINWENVAERLEAAKA

>A0A218ZVX8_1961136_Thermoplasmata_A_Arc

MAEKWE----AKDKLKPKISDQQIEYHFETHYKGYVNKLNEIWEKLSTADRSKANQNYSEFR----ELKLEETFNFDGSLLHELYFDNLSKG-QGQIPEDLRKHLEKDFGSYEKWVEDFKATGV--AFRGWSLLVYDLNTGKLRNIGADVHNTNGIWNAIVLMALDVYEHAYYTDYGPKRAPYLDAFMKNVNWPAVSKRYEKAKK

>A0A1Y4C9N7_1965623_Bacteroidetes_B_Mar

MKHLMPELPYGKEAFAPVLSEESFDYHYGKHLQTYIDTLNKLIEGT-GYENMSMEEIIMKA-------DGPVYNNAAQAWNHTLFFLTLTPAKKTMPE-KLSSLVRRDFGSEEQFRDQFIAKATAQFGSGWGWLSVDKE-GKLVITTESNAGNPLRGGLRPIMTLDVWEHAYYIDYRNRRADYLKAVWEHIDWDKVAERL-----

>A0A242PAB3_1970738_Proteobacteria_B_Mar

MAYTLPPLPYAYDALEPYIDTETMHLHHDKHHQTYVNNANALLESLPEVKDFCPETLLKNLDKVPADKVTGVKNNVGGHVNHTLFWELLKV--GTELKGELKAAIEKDFGSVDAFKEKFSAAAATRFGSGWAWLVLK-N-DKLEVVSTANQDSPVMGSGTPILALDVWEHAYYLKYQNRRADYIGAFWNVINWDKVAELYAEAKK

>A0A1E2V9J2_197479_Proteobacteria_B_Mar

MAFELPALPYEKNALEPHISQETLEYHYGKHHQTYVTKLNGLVEGT-DNAGKSLEELIKTAP------AGGLFNNAAQVWNHTFYWNCLSPNGGGEAKGAIADAINSKWGSFADFQKDFDEKAAANFGSGWTWLVKNSD-GSIEIVNTSNAGNPLTDGQTPLLTVDVWEHAYYIDYRNSRPNYLNAFWSLVNWDFVNQNFA----

>A0A4Q6B980_1977087_Proteobacteria_B_Mar

MAFKLPELPYAKDALAPHISAETLEYHYGKHHQAYVDNLNKLVAGT-PNENKSLEELVKTT-------EGGIFNNAAQVWNHTFYWYCLKPKGGGAPTGKIGDAITKNFGSFDTFKEQFSEAAKTQFGSGWAWLVKKAD-GSLAIEKTANAGNPMTSGLKPLMTCDVWEHAYYIDYRNARPKYVESFWNLVNWDFVEQQLG----

>A0A4Q3S5H0_1978230_Proteobacteria_B_Mar

--FILPDLPYARDALSPIVSGETLDYHHGKHHKTYVETLNKLLAEK-GETPESLEAVVKSA------GPGKLFNNAAQAWNHGFFWESMTPS-PAQPSGALAEAIEA-FGGHAALGEKFVETGVGQFGSGWAWLVWK-D-GKIAVAPSHDADTPLANGAFPLLVCDVWEHAYYLDHQNDRKGFLTAWFKLANWEFAGVQLAAAQG

>A0A329QP63_1981511_Actinobacteria_B_Ter

ATYSLPDLPYDYGALEPHISGQIMELHHSKHHQTYVNGTNTALEQMEEARASNKF---DIVN----QLQKNLAFNLAGHVNHTVFWNNMSPEGGDKPDGELGAAIDEFFGSFDGFRGHFTAAALGIQGSGWAILAWESLGERLVIEQLYDHQGNLAAGTVPLLMLDMWEHAFYLQYKNVKPDYVAAFWNIVNWADVADRFSKARS

>A0A4R6BX88_198484_Firmicutes_B_Ter

MAFELPQLPYAYDALEPHIDKETMEIHHTKHHNTYVTNLNAAVAG-TEFENQSIEELMANINSVPEDKQTAVRNNGGGHYNHSLFWQLLSKD-GGTPNGEVAEAINAKFGSFEAFQEAFAEAATKRFGSGWAWLVVN-N-GELEVVSTPNQDNPLMEDKTPILGLDVWEHAYYLNYQNKRPDYIKAFWNVVNWDKVNELYTAAK-

>A0A1Y0FVA0_1987723_Proteobacteria_B_Mar

MAFELPALPYAKDALAPHISAETLEYHYGKHHKTYVDKLNGLVPGT-EYEGKSLEDIIKTS-------SGGVFNNAAQIWNHTFYWNCLSPNGGGEATGAVAEAINKAFGSFDKFKEEFTNSAINNFGSAWTWLVKKAD-GSVAIVNTSNAQTPLTDSVKAILTVDVWEHAYYIDYRNSRPNYLNAFWSLVNWEFVNAKYAE---

>A0A401ZTK7_2014871_Chloroflexi_B_Ter

MAFELPKLPYDYSALEPYIDTQTMQLHHDKHHATYVTNLNNALQGH-DFANLPIEQVIRRLNEVPESARTAVRNNGGGHINHTMFWEIMTPGGSKTPTGELASAIDATFGSFDAFKTAFNDAGVKRFGSGWAWLVLDKS-GKLAITSTANQDSPFIDGNYPVLGNDVWEHAYYLKYQNRRPEYLGAWWNVVNWDIVGDRYKTALS

>A0A257EMF7_2015572_Proteobacteria_B_Mar

SPLEQPPLPFARDALAPAISADTIGVHYDKHHKGYFDTLVKLVAGS-PLAGQTLEQIIRATTD--DPAQVKIFNNAAQAWNHNFYWASLSPK-ALTPGGALAAAIARDFGDLAAVQAALIKAGTEQFGSGWAWLVAD-G-DTLKVVATHDAGVRFTSGQRPLLTVDVWEHAYYLDVQNRRADYLKAVVGHLNWDFAAANFAAD--

>A0A2D3WM99_2015906_Proteobacteria_B_Mar

MKHRLPQLPYKMDALAPYISEETLRYHHGKHHAGYVNKLNALIEGT-EYEEMSLEQIIHQS-------DGAIFNNAAQTFNHNFYWHSLSPM-KTEPSDPFRRAIEETFGSMDAFKETFVQAATGQFGSGWAWLVLDQH-DRLLIETTSNAHTPIEHHRTPLFVCDVWEHAYYIDYRNERPKYVETFWDLINWDYASKIFEDKEH

>A0A2P1PVM9_2021234_Proteobacteria_B_Mar

MAIELPALPYARDALAPHISAETIDFHYGKHHQTYVTNLNNQIKGT-EFENLSLEEIIRKS-------SGGMFNNAAQIWNHTFYWNCMKPSGGGEPTGKLADAINKSFGSFQAFKDQFTQTALTTFGSGWAWLVQRAD-GSLALVSTSNAATPLTGPDRALLTCDVWEHAYYIDFRNARAKYVETFWNLVNWDYVSAQMA----

>A0A2A6RIS4_2024553_Chloroflexi_B_Ter

MPFELAPLPYAYDALEPHIDEATMRFHHDNHHNTYVTNLNNALAKYPQLADQSLEQLLTKLDELPDDIRMVVRNNGGGHWNHTFFWQIMAA-GGGAPSGALAEAINASFGSFETFKEQFKAAGLGRFGSGWAWLVSDAA-GTLSIVSTPNQDNPLMEGKTAILGVDVWEHAYYLKYQFKRPAYLDAWWNVVNWPKVAELYAAAQK

>A0A2I0FW47_2025587_Proteobacteria_B_Mar

MSYSLPSLPYAYDALEPHFDKETMEIHHSKHHQAYVNNANAALEG-TEFANLPAEELITKLDQLPADKKGPLRNNAGGHVNHSFFWKGLKT--GTTLQGDLKAAIEKEFGSVDAFKEAFEKAAATRFGSGWAWLVKK-D-GKLAVVSTANQDSPLMGSGTPIIGLDVWEHAYYLKYQNKRPDYAKAFWNVVNWDVAAENFAKA--

>A0A399EPG5_2026184_Deinococcus-Thermus_B_Ter

MSFKLPELPYPKDALEPHIDAQTMEIHHGKHHATYVNNLNAALEKHPELHSWSIEDLLTKIAQVPEDIRTAVRNNGGGHHNHTLFWDILTPGGAKEPTGRLAEAIQATFGSFEELKNKMTQAGLTRFGSGWAWLVKDKD-GKLLVYSTANQDSPLMEGHTPLLGIDVWEHAYYLKYQNRRPDYLAAIWNVINWDKVAERF-----

>Q83GI4_203267_Actinobacteria_B_Ter

LLYTLPDLPYGYSALEPYISGKIMELHHSKHHKAYVDGANQALEQLCEARDKGDF---SRVP----QLQKNLAFNLAGHKNHSVFWSVLTPEVGQAPAGPFLEVIERDFGSFDSFMNHFSAAALSIQGSGWAVLSWEPVASRLIIQQFYDHQNNAV-SARNLLLLDMWEHAYYLDYLNVKADYVKAFWSIVNWHGVTDKFLKIAG

>A0A2A5LZ91_2039467_Proteobacteria_B_Mar

MAFELPALPYEKNALEPHISAETLEYHYGKHHNTYVTKLNGLVEGT-DNANKSLEEIIKSA-------SGPVFNNAAQVWNHTFYWHCLSPNGGGEPTGAAKEAIEKAFGSVEEFKKEFSDKAANNFGSGWTWLVKKAD-GSVAIVNTSNAETPLTGADKPVLTVDVWEHAYYIDYRNSRPNYLEAFWKLVNWDFVNENLA----

>A0A4Q2ZMN6_2044944_Bacteroidetes_B_Mar

MAYTLPALPYDFSALEPHIDAQTMQIHHDKHHQAYVDNLNKALEG-SEKADMPIEELMATIDQYP----AAVRNNGGGHYNHSLFWEVLGAN-GGEPTGALKTAIDDAFGSLDTLKEKMADAGAKRFGSGWAWLIVK-D-GKLAVTSTPNQDNPLMPQGTPIFGIDVWEHAYYLNYQNKRPDYLKAIWNVVNWNEVARRYEAASG

>C1DW98_204536_Aquificae_B_Anc

KVVKLQ----PKDHLKPKISDEQIEVHFEAHYKGYVAKYNEIQEKLASADRSKANQNYSEYR----ALKVEESFNYMGVVLHELYFENLVAGGKGEPSPELKKMIEEYFGSVNNCINEIKATGI--ACRGWATLSYDLYNKILVVNGFDAHNQYGFVYSVPLIVLDVYEHAYYVDQKNKRPPYIDAFFKNLNWEVVNERFNKAVK

>A0A418MLK8_2048547_Firmicutes_B_Ter

MAHELPPLPYAYDALEPYIDEMTMKVHHDGHHGTYVKNLNAALEGHPELQSKSIEDLLHDINTVPESIRTAVRNNGGGHANHSMFWQIMSPNGGGQPSGALADALSTTFGGFEKFKEEFGKAAATRFGSGWAWLVVK-D-SKLSIISTANQDSPYMENQLPIFGLDVWEHAYYLKYQNRRPEYISAFWNVINWAEVEKRYQSAK-

>A0A2N0WW65_2058089_Proteobacteria_B_Mar

MAIELPALPFEKNALEPHISAETLEFHYEKHHATYVVKLNGLIEGS-DLANSSLEEIVKNS-------EGPVFNNAAQVWNHTFYWNCLAPNAGGEPTGPIADAINQTFGSFDAFKAAWNDKAVNNFGSSWTWLVKKSD-GALEIVNTSNAATPLTDSVTVLLTVDLWEHAYYIDYRNVRPNYLNGFWALVNWDFVNSNYA----

>A0A1R3VN56_233100_Proteobacteria_B_Mar

MSIELPPLPYAKNALEPHISAETLEYHHDKHHATYVANLNKMIAGT-EFENMGLDDIVKKAP------AGGMFNNAAQIWNHTFYFEGLTPNGGGEPSGALADAIDKAFGSFEGFKEKFTAAGAGNFGSGWTWLVKKAD-GSVEIVNTDDAETPITDGVTPLLTMDVWEHAYYVDYRNARPKYIENFFNVVNWDFVAKNYGA---

>A0A0B0HBS3_2340_Proteobacteria_B_Mar

MQHQLPELPYEKSALEPHISAETLEFHHDKHHATYVTNLNNLIEGT-EFADKSLEEIVTSAP------AGGIFNNAAQVWNHTFYFNCMAPEAGGAPSGEIAAAIDAAFGSFEDFKTAFSTSAATNFGSGWTWLVKNAD-GGVEIFNTSNAGCPLTEGLTPLLTIDVWEHAYYVDKRNARPAYVESWWNLVNWEFVNAQL-----

>Q02A56_234267_Acidobacteria_B_Mar

MPFTLPPLPYAPDALEPHIDKMTMEIHHGKHHNAYVTNLNKALESAPELADKNIEELLANCAIVPEKIRTAVRNNGGGHINHSMFWKIMGPNGGGAPVGNVAQAITGAFGSFDAFKEKFNAAGVGRFGSGWAWLLKT-S-SGVEITSTANQDSPIMEGKVPVFGCDVWEHAYYLKYQNRRPDYLAAWWNVVNWKEIEDRFNAR--

>Q3Z7W8_243164_Chloroflexi_B_Ter

--------MYTASALLG-FSDTLLKNHF-TLYQGYVNNTNKLSDTLKTMLAEGKT-AAPEYA----ELKRRFGFEFDGMRLHEYYFSNLGKSAPLSNTGKLYQALSAEFGSYQLWETDFKATAS-MRGIGWVILYKDSQTGRLFNQWINEHETGHLAGITPILVIDIFEHAFMTDYGLKRADYIAAFLKNINWDEAEKRFQN---

>Q9RUV2_243230_Deinococcus-Thermus_B_Ter

MAYTLPQLPYAYDALEPHIDARTMEIHHTKHHQTYVDNANKALEG-TEFADLPVEQLIQQLDRVPADKKGALRNNAGGHANHSMFWQIMGQNGANQPSGELLDAINSAFGSFDAFKQKFEDAAKTRFGSGWAWLVVK-D-GKLDVVSTANQDNPLMGSGTPILGVDVWEHAYYLNYQNRRPDYLAAFWNVVNWDEVSKRYAAAK-

>A0A202E7D5_253108_Halobacteria_A_Arc

ADHELPPLPYDYDALEPSISEQVVTWHHDTHHQGYVNGLNAAEETLEENRESGDY---GSTA----GALGNVTHNGSGHYLHTLFWENMSPNGGGEPAGDIADRIEEDFGSYEGWKGEFEAAAS--AAGGWALLVYDPVSKQLRNLAVDKHDQGALWGSHPILALDVWEHSYYYDYGPDRGEFIDGFFDVINWDSVDEEFQKCLD

>T0MZU0_261391_Thermoplasmata_A_Arc

MVEKWE----KKNQFKPKISDQQIEYHFETHYNGYVTKLNEIWEKLPNADRSKANQNYSEFR----ELKLEETFNYDGSLLHEIYFESLKKDGLKNLSEELKKKISEDFGSYEKWVEDFNATGT--AFRGWALLVYDLNTGKLRNIGADVHNTNGIWNAIVVMALDVYEHAYYVDYGAKRAPYLDAFMKNVDWASVNKRFEKAHK

>Q6YQD2_262768_Tenericutes_B_Ter

MNFTLLSLPYQYDALEPFFDTQTMQLHHLKHHQTYINNLNDALKKHPQL-NLSLEQMLTDLSLVPQDIRQTVRNNGGGHFNHSFFWNILKVNNGNTPQGLLKEMIDCEFGSIDSFKDKFANAAKTIFGSGWAWLVLTPQ-QKLAITFTPNQDVVLNQ-GTPLLGLDVWEHAYYLSYQNRRVDYIEAFFSVLDWEKVQNNLTQTLK

>Q6L1T7_263820_Thermoplasmata_A_Arc

MAETWE----VKEKLKPRISDQQIDYHFDFHYKGYVTKLNEIWSKLPDVDLSKANQNYSDLR----EMKLEETFNYDGSMLHEYYFESLTKD-HTEIPASVKEQIEKDFGSYEKFVALFKATGT--AFRGWAHLIFDLNYGKLRIVGADIHSAGAIWNAIMILPLDVYEHAYYTDYGAKRAPYLDAFMKNVNWKVVEKRLEKAKK

>Q1ARZ8_266117_Actinobacteria_B_Ter

MAYELPPLPYDYNALEPYIDEATMRFHHDNHHNTYVTNLNAALEKHPDVDPGNVDELIADLNAIPEDIRRAVRNNGGQHSNHSIFWQIMAPSGGGEPTGELGEAINGTFGSFDAFKEQFAAAAAALFGSGWCWLVVTSG-GQLAIKTTPNGDSPYMEGEIPVIGLDCWEHSYYLKYQYRRPEYVQNWWNVVNWEEANRRYQAARS

>M1XSP2_268739_Halobacteria_A_Arc

SNPELPPLPYEYDALEPSISEQVVTWHHDTHHQGYVNGLEAAEETLAENREAGEF---GSSG----AAIRNVTHNGSGHYLHTLFWENMDPNGGGEPSGELADRIEADFGSYEGWKGEFEAAAG--EASGWALLVYDPVSDQLRNLVVDKHDQGALWGSHPVLALDVWEHSYYYDYGPDRGSFIDGFFEVVDWDNVAEQYEKAIQ

>Q3YRT4_269484_Proteobacteria_B_Mar

--FTLPELPYQQSDLVPYLSPEILGYHYNKHHQGYVNTLNSLVVGT-DFSEEDLPKIIEATAG--DLGSRSIFNNAGQIWNHNFYWESMKKNGGGAPTGKLLDKINEDFGSIDDFNNAFTNAGKSHFGSGWVWLVFDSE-QKLKILCTSNGDTPITQETHPLLTMDVWEHAYYLDYFNVRQNYVETFLHLVNWDFAAQRFLEV--

>Q9Y8H8_272557_Thermoprotei_A_Arc

KRYELPPLPYNYNALEPYIIEEIMKLHHQKHHNTYVKGANAALEKIEKHLKGEIQ---IDVR----AVMRDFSFNYAGHIMHTIFWPNMAPPGGGTPGGRVADLIEKQFGGFEKFKALFSAAAKTVEGVGWGVLAFDPLTEELRILQVEKHNVLMTAGLVPILVIDVWEHAYYLQYKNDRGSYVENWWNVVNWDDVEKRLEQALN

>O84296_272561_Chlamydiae_B_Mar

SSYMLPALPYDYDALEPVISAEIMQLHHQKHHQGYINNLNEALKSLDVANATQDL---ARLI----AINPALRFNGGSHINHSLFWEMLAPQGKGPPRHELLKLIEKFWGSFDNFLKNFITSSAAVQGSGWGWLAFCPQKQELMVQTTANQDPLATTGMIPLLGVDVWEHAYYLQYKNARMDYLKSFPSIINWDYIENRFVEMSK

>Q03302_272569_Halobacteria_A_Arc

SNPELPPLPYDYDALEPHISEQVLTWHHDTHHQGYVNGLESAEETLAENRDAGDF---GSSA----AAMGNVTHNGCGHYLHTLFWENMDPNGGGEPEGELLDRIEEDFGSYEGWKGEFEAAAS--AAGGWALLVYDPVAKQLRNVPVDKHDQGALWGSHPILALDVWEHSYYYDYGPARGDFIDAFFEVVDWDKAAEEYEKSVS

>Q9ZD15_272947_Proteobacteria_B_Mar

YPFILPDLPYDKESFKPHFTRETFDYHHGKHHNSYVQNLNNLIKDREELQKKDLEEIIEWSSQ---NAEVAILNNASQIWNHTFFWYSIKPHGGGKPSGKVFEQISKDFGSFEQFCAQFKQEAVGQFGSGWTWVVYH-D-NKLQIIKTSNAGTPIVNFMKPILACDVWEHAYYIDYRNKRSDYIDIFIHMINWKFVEDNLIQ---

>P80857_273057_Thermoprotei_A_Arc

KKYELPPLPYKIDALEPYISKDIIDVHYNGHHKGYVNGANSLLERLEKVVKGDLQTGQYDIQ----GIIRGLTFNINGHKLHALYWENMAPSGGGKPGGALADLINKQYGSFDRFKQVFTETANSLPGTGWAVLYYDTESGNLQIMTFENHFQNHIAEIPIILILDEFEHAYYLQYKNKRADYVNAWWNVVNWDAAEKKLQKYLT

>Q96Y84_273063_Thermoprotei_A_Arc

KKYELPPLPYKVDALEPYISKDIIDVHYNGHHKGYVNGANSFLERLEKIIRGEITSGQYDIQ----GLLRGLVFNINGHKLHALYWQNMAPAGGGKPGGALADLIDKQYGSFDKFKQLFTEAANSLPGTGWTVLYYDTESGNLEIMTFENHFQNHIAELPIILILDEFEHAYYLQYKNKRADYVNAWWNLVNWDEADKKLQKYLN

>Q9HM56_273075_Thermoplasmata_A_Arc

MAETWE----IKEKLKPRISDVQIDNHFDVHYKGYVNKLNEIWSRLPDVDRSKANQNYSEFR----ALKLEETFNYGGSLLHELYFEGLTPK-HSEVPKEFKDAVAKDFGSYEKWLEDFKATGT--AFRGWAILVFDLNYGKLRNIGSDAHNVGLIWNSIAILTMDVYEHAYYVDYGAKRAPYLDAFLKNVNWPVVLDRLNRAKK

>A0A1V0RDW6_28898_Proteobacteria_B_Mar

--FELRKLPYNTNAFGDFLSAETFSYHHGKHHQTYVNNLNNLIQNT-EFAGKDLVSIIKNS-------SGGIFNNAAQVYNHDFYFDCISPSQSTCECPNLKAALEKNFGSLESFKDEFIKGATGVFGSGWFWLVLDSKTQKLEFVGTSNAATPICDDKIPLLVVDVWEHAYYVDHRNARPAYLEKFYAHINWAFVAKAYEWALK

>Q2S1T9_309807_Bacteroidetes_B_Mar

MAFDLPDLPYDYDALEPHIDEQTMRIHHDKHHAGYTRKLNNALEGHDDLQEHSIEELLAGLDTLPTDVQTPVRQNGGGFYNHRLFWNVMSPDGGGTPDGDLADAIHDAFGSYEDFKDAFADAATGQFGSGWGWLVAQPN-GDVTVTSTPNQDNPLLEGHTPILGIDVWEHSYYLNYQNERGTYVDEWWNVVDWDAVGENYDEIVG

>A4AJQ1_312284_Actinobacteria_B_Ter

AEYTLPELSYDYSALEPSISAMIMELHHSKHHQAYVTGANAAIAGLAEARDSGNL---SNVN----KLEKDLAFNLGGHINHSIFWTNMSPNGGDKPTGDLESAINDHFGSFDKFTAHFTATAMGVQGSGWAVLAWDSIGERLIIVQFFDQQGNLPAGIVPLLMLDVWEHAYYLDYKNVRADYVKAFWNIVDWANVQDRFSAARS

>A0A0N8VL92_312540_Thermoplasmata_A_Arc

ATETWE----VKEKLRPRISDTQIDYHFDAHYKGYVAKLNEIWSKLPSVDLSKANQNYSDLR----EMKLEETFNYDGSMLHEYYFESLTKD-HVEMPASVKEQIEKDFGSYENFVALFKATGT--AFRGWAHLIFDLNYGKLRVVGADIHSAAAIWNALMILPLDVYEHAYYVDYGAKRAPYLDAFMKNVNWKVVEKRLERAKR

>A0YJL0_313612_Cyanobacteria_B_Ter

MAFELPELPYPADALESSMSKQTFSFHHGKHHAAYVNKLNGMIEGT-DLANKSLEEIVKATAN--DPSKSGIFNNAAQVWNHSFFWKCMKPGGGGKPSGALAEKINADFGSYEKFAEEFKNAGATQFGSGWAWLVLE-N-GSLKVKKTLNAINPIIEGVTPLLTMDVWEHAYYLDFQNARPGYIDNFLKLVNWDFVAENLAAAQ-

>E0TI36_314260_Proteobacteria_B_Mar

MAISLMDLPYEKTALAPHISEDTLNYHHGKHHQAYVTKTNDAIKGT-ALDDADLEAIVKEAKK---TANQGLFNNSAQVWNHNIYWQSMSPNGGGAPKAKIAEAIDKSFGSYDEFKAKFKDAGGTQFGSGWAWLVAKKD-GSLEILKTLNADCPLTDSVTTLLTMDVWEHAYYLDYQNARPDYMTHFLNLVNWDFAEERLAAA--

>A4BGS7_314283_Proteobacteria_B_Mar

MAFELPELPYAKTALEPHISAETLEFHHGKHHATYVTKLNGLIPGT-EFEGKSLEDIIKSS-------SGGVFNNSAQIWNHTFYWHSLSPNGGGQPTGALAEAINAKWGSFEDFQKAFDEKAVNNFGSSWTWLVKNSD-GSLDIVNTSNAGTPITEGQTPIITVDLWEHAYYIDYRNARPKYLEAFWALANWEFAAENFAK---

>A0A0M2Q238_317619_Cyanobacteria_B_Ter

MAYELPALPYAYTALEPSITQATLEFHHDKHHAAYVNNYNTAVAGT-EYDSMALEAVIKAVAG--DASKAGLFNNAAQAWNHSFYWLCMKSGGGGAPTGALADKITADFGSFEEFVTAFKTAGATQFGSGWAWLVLD-N-GTLKVTKTGNAENPMTSGQVPLLTMDVWEHAYYLDYQNRRPDYMTDFLKLVDWDFVAANLAAA--

>Q2FSC2_323259_Methanomicrobia_A_Arc

KKYTLPALSFEYGALAPFITEKQLTLHHQKHHQAYVTGANAIFEKLEMARREGGD---LDQK----ALLKELSFHIGGHRLHTLFWENLAPAGGGVPSGILADWINRDFGSLDRFKKEFTQTASSVEGSGWAVLSVCLGTQRLLLMQVEKHNVHVYPGFRILMVLDVWEHAYYLDYMNDRAKFIENFWNIIRWDMVNQRLEAALK

>A0A2T0XJH1_323426_Proteobacteria_B_Mar

MAHTLPPLPYAMDALAPTISKETLEFHYGKHHQTYVTNLNNLIPGT-EFESASLEEIVKKS-------SGGVFNNAAQIWNHTFYWNSLSPNGGGEPTGKLADGITAKWGSVAAFKEAFNKSAAGNFGSGWTWLVKKAD-GSLDIVNTSNAGTPLTTTDVALLTCDVWEHAYYIDYRNARPKYLESFWSLANWSFAAQNLA----

>A9WJC9_324602_Chloroflexi_B_Ter

MAFEVPPLPYDYNALEPYIDEATMHYHHDNHHQTYVTNLNNALANYPELQSKTIEELLGNLDAIPEAIRTAVRNNGGGHWNHTFFWEIMAPNAGGAPTGDLAAAIDAAFGSFDAFKEKFKAAALGRFGSGWAWLVAAKD-GSLSIMSTPNQDNPLMEGKTAILGLDVWEHAYYLKYQYKRAAYVDAWWNVVNWAKVADHYAAAKG

>Q08713_330779_Thermoprotei_A_Arc

KRYEFPQLPYKVDALEPYISKDIIDVHYNGHHKGYVNGANSLLDRLEKLIKGDLPQGQYDLQ----GILRGLTFNINGHKLHAIYWNNMAPAGGGKPGGALADLINKQYGSFDRFKQVFSESANSLPGSGWTVLYYDNESGNLQIMTVENHFMNHIAELPVILIVDEFEHAYYLQYKNKRGDYLNAWWNVVNWDDAEKRLQKYLN

>B3EJL9_331678_Chlorobi_B_Mar

MAYTQPALPYAENALAPHISAETIGFHYGKHHATYISKYNGMVAGT-PFDDQTIEDVIVATAN--DPEKAGLFNNGAQAWNHSFYWNSLSPDGGGAPGGQIGEKINADFGGYDKFREELANAAATQFGSGWAWLVLD-K-GQLKIVKTANAQTPLTNGMFPILTIDVWEHAYYLDYQNRRPDYVSAVIELLNWNFAASNFEAAQV

>A8U258_331869_Proteobacteria_B_Mar

MAFELPPLPYAYDALAPYMSAETLEFHHDKHHNAYVVNGNNLLKDS-GLEGKSLEEVVKASYK--NDKLAGLFNNAAQHWNHIEFWQWMKPKGGGAMPGELEKRIAADFGSVAKFKEDFVNAGVTQFGSGWCWLAVGSD-GKLKVTKTANGVNPVALGETALLGCDVWEHSYYIDYRNRRPDYVKAFLSMVNWERVAELLGKAS-

>A0A4R7K0K2_332522_Proteobacteria_B_Mar

MAFELPKLPFSENALAEKMCQETLELHHGKHHNAYVTTLNTLVEDS-NLQGKSLEEIVKLSYG--RDDLQKVFNQAGQHWNHNLFWENLSPKGGG-IPGKLEQKINEDFGSVDTFKEEFKTAATGQFGSGWAWLIVGSD-GKLKVTKTPNAVNPLATGEKAILGLDVWEHSYYLDFRNRRPDYVMNFLNLANYEYAEELLNKA--

>S0AS08_333146_Thermoplasmata_A_Arc

ATETWE----VKENAKPRISDKQIDYHFDVHYKGYVSKLNEIWSKLPDVDLTKANQNYSDLR----EMKLEETFNYDGSMLHEYYFESLSKD-HVAMPESVKAQIEKDFGSYEKFVALFKATGT--AFRGWAHLVFDLNCGKLRVLGADIHNASAIWNALMILPLDVYEHAYYTDYGAKRAPYLDAFMNNVNWKVVEKRLDRAKR

>Q0AW95_335541_Firmicutes_B_Ter

MAHTLPDLPYAYDALEPFYDEQTVRLHHDMHHKAYVDGLNNAESKLAEALEKGDF---ALIK----HIERELSFHGSGHILHTMFWENMKPGGGGPADGAVAELIDRDFGSFDNFKKLFSASALAVEGSGWTILACNPIFEKLVVLQAEKHQDLTQWGAVPLLIVDVWEHAYYLKYQNKRAAWIEAWWNLINWDDVNRRVALMQQ

>A0A2T0GV44_33906_Actinobacteria_B_Ter

AQYVLPELDYDYSALEPAIAGEINELHHSKHHAAYVKGANDTIEKIAEAREKGDF---SSIV----GLETTLAFNLAGHSMHLVWWKVLSPDGGDKPTGELAAAIDEHFGSFDKFRAQMEAVSTTIQGNGWGVLAWDPVGQRLITQQLRDHHSNLSIATTPLLVFDIWEHAYYLQYKNAKADYVQQLWNVVNWDEVSRRFADARA

>A0A1I1DPZ5_34097_Spirochaetes_B_Mar

-MFTLPELGYSYDALEPYIDAKTMEIHHTKHHKGYIDNLNAALEKHPEWADKSLKEILANIESVPEDIRMAVRNNAGGHHNHTLFWKLLKKNNGAKPTGELLKLIERDFGSFEKFQEMFETAAKTRFGSGWAWLMMDKN-GKLSIISTPNQDSPIMDGFFPLLGLDVWEHAYYLKYQNRRPDYIKEFWNIIDWDIVSSRMNGSCG

>A0A1U7EY38_348780_Halobacteria_A_Arc

SNPELPPLPYDYDALEPSISEQVVTWHHDTHHQGYVNGLESAEETLAENREAGEF---GGSA----AAIRNVTHNGSGHYLHTLFWENMDPNGGGEPSGDLADRIEEDFGSYEGWKGEFKAAAG--AAGGWALLVYDPVADQLRNLVVDKHDQGALWGSHPILALDVWEHSYYYDYGPDRGEFIDGFFEVVDWDNVAEQYEKATQ

>A0B701_349307_Methanomicrobia_A_Arc

KFYTLPELPYGAKDLEPYISEAQLKLHHDKHHLAYVNGANAILEKLDKARREGAD---IDQK----STLKELSFHIGGHLLHSLFWANMRKPVG--PVGALADEIKDEFGSFERFRKEFTAAAVSVEGSGWAALAYCMQTRRPIIMQIEKHNTNICPMFRLLLVLDVWEHAYYLDYRNDRAKFVEAFWQIADWEEANRRFESLIG

>A5UY95_357808_Chloroflexi_B_Ter

MAFTLPPLPYDFSALEPHIDTMTMQIHHGKHHQAYVTNLNAALEGHPSLQNATIEEILSNINDVPESIRQAVINNGGGHHNHTLFWNIMGPNGGGEPTGALARAINDTFGSFAEFKAKIKDAGIKRFGSGWAWLVKDKD-GKLHIYSTPNQDSPLMQGHIPILGVDVWEHAYYLKYQNRRPDYIDAWWNTVNWEAVAARFEQ---

>M0L696_358396_Halobacteria_A_Arc

TDHELPPLPYDYDALEPAISEQVVTWHHDTHHQGYVNGLNSAEETLAENRESGDF---DSTP----GALSNVTHNGCGHYLHTLFWENMSPDGGGEPEGDLADRIEEDFGSYEGWKGEFEAAAG--AAGGWALLVYDPVAKQLRNVAVDKHDQGALWGSHPILALDVWEHSYYYDYGPDRGSFIDAFFDVVDWDKAAEEYQKCLD

>A0A2S1JPT9_359370_Proteobacteria_B_Mar

MAFELPELPYAKNALAPHISEETLEYHYGKHHKTYVDKLNGLLEGT-ADADKSLEDVIKSS-------SGGVFNNAAQVWNHTFYWNCLSPNGGGEATGAIAEAINAAFGSFDQFKEAFTTSAVNNFGSGWTWLVKNAD-GSVAIVNTSNAATPLTEGVTPLLTCDVWEHAYYIDYRNLRPKYMEAFWALVNWEFVNQNFA----

>Q18HG3_362976_Halobacteria_A_Arc

MSYELDPLPYEYDALEPQLSEQVLTWHHDTHQQGYVNGWNNAEAELEENRDSHDF---SSSA----GAIRDVTHNSSGHLLHDLFWQCMSPEGGDEPTGDLGDRIEEDFGSYDAWRGEFEAAAG--DASGWALLVYDSFSNQLRNVVVDNHDEGAFWGAHPVLSLDVWEHSYYHDYGPARGDFVDAFFEVVDWEEPSQRYAQAVE

>Q0F8L9_367336_Proteobacteria_B_Mar

MAFELPELPYSHDALAKGMSAETLEFHHDLHHKAYVDNGNKLISGT-EWENKSLEEITIGTYNSTSVSQNGIFNNISQLWNHNQFWEMMGPDGR-AMPSELEAAIVESFGSVEAFKSDFGAAGVGQFGSGWCWLVKDTD-GSLKITKTENGVNPLCFGQKTLLGCDVWEHSYYIDFRNKRPVYISNFLNLVNWENVAARLASS--

>Q1LSZ4_374463_Proteobacteria_B_Mar

MIFMLPTLPYRFDAFEPFIDQTTMEIHYTKHHQNYITNANLILESLPQFAQLSVEELIQQLDQVPHNKKMALRNYAGGHANHSLFWKLL--KHGTKIQGKLKNAIESDFGSIIIFQELFEQVAMNCFGSGWAWLVKK-N-NQLLVVSTANQDNPLMGSGYPILGLDVWEHAYYLKYQNRRSDYIKSFWNIVNWDEASIRFNQCED

>A0A166PIY3_37915_Actinobacteria_B_Ter

AEYVLPDLDYDYGALEPHISGEIMELHHSKHHATYVKGANDALEQLAAAREDGSI---SKAP----LLSKNLAFHLGGHTNHSIFWKNLSPEGGDRPEGELAAAIDAEFGSFDKFQAHFNAVATTLQGSGWSVLGWDHIGQRMVIQQLTDQQGNISVNITPLLMLDMWEHAFYLQYKNVKADYVKAFWNVVNWKDVAERYAAAKG

>B4U6J9_380749_Aquificae_B_Anc

--MKLE----PKNHLKPSISNEQIEPHFEAHYKGYVAKFNEIQDKLADSDRAKANQNYSEYR----ELKVEETFNYMGTVLHELYFGHLTP--KGTPSEALKKKVEEDFGSWDNCVTELKAAGI--AFRGWAILGLDIFSGKLMINGLDAHNLYNLTGLIPLIVLDTYEHAYYVDQKNKRPPYIDAFLNSLNWDVINERFEKAIK

>A0A4R8ITH7_381308_Proteobacteria_B_Mar

QIHVLEVLPYAVNALSPVISSNTVEVHYGKHHKGYVDKLNKQVEGT-EFSDMSLESLITGTAG--KPDHVGIFNNAAQAWNHTFYWRSLKQKGGGEPPAMLKDKIEASFDSLDACREELVKAATTRFGSGWAWLVQD-G-DQIKVVSTGNADNPIANGMTPLLTIDVWEHAYYLDYQNRREEYVRAVLKLINWEFAAENLD----

>A1RW25_384616_Thermoprotei_A_Arc

KKYTLPNLPYAYNALEPYIAEEIMRLHHQKHHQGYVNGANAALEKLEKFRRGEAQ---IDIR----AVLRDLSFNLNGHILHSIFWPNMAPPGGGKPGGRTADLINKFFGSFEKFKEEFSLAAKNVEGVGWAILVYEPLEEQLLVLQVEKHNLMHAAGAQVLLALDVWEHAYYLQYKNDRGSYVDNWWNVVNWDDVERRLEKALN

>A0A073CMZ0_388467_Cyanobacteria_B_Ter

MAFELPALPFADDALESSMSAKTFSFHHGKHHAAYVTNLNKLIDGT-GLADKSLEEIIKATAN--DPNKAGIFNNAAQVWNHTFFWNSLKAGGGGQPTGALADKINADFGSFDKFIEEFKAAATTQFGSGWAWLVLN-N-GTLKVTKTANAGTPIVDGLTPLLTLDVWEHAYYLDFQNARPAYIENFLQLVNWDFVAENFAAA--

>B5JWC0_391615_Proteobacteria_B_Mar

MSFELPTLPYEKNALEPTISAETLEFHHGKHHAAYVNKLNDMIQGT-EFENLSLEEIIQKS-------SGGMFNQAAQIWNHTFYFTGLSPNGGGEPTGALADAINAEFGSFDEFKAKFNAAGAGNFGSGWTWLVKNAE-GKLEIVNTDDAETPLTNGQTPVITVDVWEHAYYIDYRNARPKYLEEIWNIIDWSVAAERFGA---

>A0A0B4XLG0_391936_Proteobacteria_B_Mar

MAFELPALPYAKDALAPHMSAETLEFHYGKHHQTYVTKLNELVAGT-DKEGQSLEDIIKGAG------PGPLFNNAAQVWNHTFFWNSLSPNGGGEPTGALADAINAKWGSFDKFKEAFTNSAIGNFGSGWTWLVKNGN--ELEIVNTSNAATPLTDGKTALLTVDVWEHAYYIDYRNARPKYLEGFWALVNWEFAASNFA----

>A8MAM7_397948_Thermoprotei_A_Arc

KRYELPPLPYNVNALEPYISGQVIDVHYNGHHKGYVNGANAAIDRLEKIIKNEVTS--YDIQ----GLLRNLFFNINGHKLHTLYWNSMAPAGGGTPGGYLGDLVIKQFGSYDKFRNLFNEVMRSLPGSGWAVLYYDTETGNLVFTTFENHYNQHIAELPVILIIDEFEHAYYLQYKNNRNAYLDAIWNVLNWGEAENRLRKYIK

>A8H3Z8_398579_Proteobacteria_B_Mar

MAFELPALPYAKNALEPHISEETINFHYGKHHNTYVVKLNGLVEGT-ELEQKSLEEIIKTS-------TGGIFNNAAQIWNHTFYWNCLSPNGGGEATGPVAEAIIAAFGSFEAFKAQFTDSAVNNFGSAWTWLVKNAD-GTVAIVNTSNAATPLTDGVTPIMTVDVWEHAYYIDYRNVRPDYLAHFWQLVNWDFVNANFAA---

>A4YHX7_399549_Thermoprotei_A_Arc

KKFELPPLPYKVDALEPYISKDIIDVHYNGHHKGYVTGANTFMERFNKVIKGELQSGQYDVQ----GLMRGIVFNINGHKLHALYWDNMAPAGGGQPGGALADLIVKQYGSYDRFKQVFTETANSLPGTGWTVLYYDTENGNLEIMTFENHFMNHIAELPILLILDEFEHAYYLQYKNKRADYVTNWWNVVNWDYAEKKLQKYLK

>A7HBL1_404589_Proteobacteria_B_Mar

------VKRYTPSKVRG-ISDKQLEEHL-KLYEGYVKRTNALTEKLQAICADGKASADPVFA----ELTRRLGFEYSGMVNHEYYFDNMAPGAQAEPGGKLRKALEASFGKYETWLADFRAIAT-MPGIGWAMTFQDPTTGWLSNHWINLHQDNVPVGFKPVLVMDGWEHAYMRDYATERAKYVDAFFKNVSWEAVEKRIA----

>A0A2U9IFV2_41673_Thermoprotei_A_Arc

KKYELPPLPYKLDALEPYISKDIIDLHYNGHHKGYVNGANSFLDRMQKVTKGELSSGQYDIQ----GLLRGLVFNINGHKLHSLYWENMAPNGGGKPGGSLADLIEKQYGSFDKFKALFTEAANSLPGTGWTVLYYETENGNLQIMTFENHFQNHIAELPIVLILDEFEHAYYLQYKNKRADYVNAWWNLVNWDYANKKLEKYLN

>A0A1R1I0D0_418702_Proteobacteria_B_Mar

MEHQLPALPFAKDALAPHMSAETFDYHYSKHHNAYVVNLNNLIKGT-EYEALDLEAIVKKAP------AGGIYNNAAQVWNHTFFWNCLTPNGGGAPSGALADAINAKWGSLDAFKTAFQTSAVGNFGSGWTWLVKKAD-GSVDIVNMGAAGTPLTTGDKALLCVDVWEHAYYIDYRNLRPKFVETFLNLVNWSFAEANFAG---

>A0A0J8VRJ1_435910_Proteobacteria_B_Mar

MSYTLPSLPYAYDALEPHFDKQTMEIHHTKHHQTYVNNANAALESLPEFANLPVEELITKLDQVPADKKTVLRNNAGGHANHSLFWKGLKK--GTTLQGDLKAAIERDFGSVEKFKEEFEKAAATRFGSGWAWLVKQGD--KLAVVSTANQDSPLMGSGYPILGLDVWEHAYYLKFQNRRPDYIKEFWNVVNWDEAAERFASQK-

>A0A1G6AIZ5_439219_Firmicutes_B_Ter

MAIILPDLPYAYDALEPYIDAETMTLHHDKHHATYVANANAALEKHPE-IGEDLVALLSDVEQIPADIRQALINNGGGHLNHALFWELLSPE-KTEITEEVAAAINEAFGSFDAFQEAFTVAATTRFGSGWAWLVVNAE-GKLEVMSTANQDTPIMEGKQPILALDVWEHAYYLNYRNVRPNYIKAFFEIINWTKVAELYAAAKA

>D3PZ20_446470_Actinobacteria_B_Ter

GTYSLPDLSYDYGALEPAMSGDILELHHSKHHAAYVKGANDTLERIAEAREKGDY---ASLV----GLEKTMAFNLSGHVLHSIFWNNLSPEGGDRPDGELAAAIDEHFGSFEKFSAQLSAATTGVQGSGWGVLSWEPLGKRLIIEQVYDHHGNVGQGSTPLLAFDAWEHAYYLQYRNVRPDYVQRLWDLVNWNDVIARFEAARG

>B0JGF5_449447_Cyanobacteria_B_Ter

MAYTLPPLPYDYTALEPCITKSTLEFHHDKHHAAYVNNYNGLVKDT-ELDALSLEEVIVKVAG--DASKAGVFNNAAQAWNHSFYWDCMKPGGGGAPTGALADKIQADFGSFEKFVEAFKTAGATQFGSGWAWLVLD-N-GTLKVTKTGNADNPLTAGQVPLLTMDVWEHAYYLDYQNRRPDYISDFLKLVNWDFVAANLAAA--

>B3ER22_452471_Bacteroidetes_B_Mar

MAFELPTLPYAPEALEPHIDATTMQLHHAKHHQAYLDKFNKAIEG-TDLAARSIEDILKNVSNY----SVVVRNNGGGYYNHNLFWPMLTPNGGGEPTGAIVDAINQKFQGFAQFKEAFAQAALEQFGSGWAWLCKDIT-GELFICSTANQDNPIMDRGIPVLGLDVWEHGYYLKYQNRRAEYIDAFWNIVNWEEVNKRFQAPMP

>A0A0W0TNM8_453_Proteobacteria_B_Mar

MAFTLPQLPYAMNALEPHISQETLEYHYGKHHNAYVTNLNKLIPGT-EYENLSLEEIIKKS-------SGGIFNNAAQVWNHTFYWHCLSPNGGGEPTGKLADAIKKDFGSFAAFQEQFTQTAATTFGSGWAWLVQEKD-GSLKITSTSNAGTPMTAGQQALLTCDVWEHAYYIDYRNVRPDYIKAFWSLVNWEFVESNMR----

>A0A2U1E3U8_46507_Firmicutes_B_Ter

--IELIKLPYAYDALEPVISKETIEYHHDKHHQAYVNKLNELIEG-TELADMHPCEILKNLDKAPADKKQAIINQGGGVYNHNVYWTQLAKDGQKEPKGELKEAIDKAFGSFDEFKEKFEKAGAGQFGSGWAWLVEK-D-GKLEIMTTANQDAPISKGYKVLLNNDVWEHAYYIDYRNKRAEYLKKFWDIVDWSVVEERFVK---

>E4NT27_469382_Halobacteria_A_Arc

MSYELDPLPYEYDALEPHISEQVLTWHHDTHHQGYVNGWNSAEETLESNREEGDF---GSSP----GAIRNVTHNGCGHILHDLFWNSMSPEGGSEPTGSLADRIEEDFGSYEAWKGEFEAAAG--NAGGWALLVYDSFSNQLRNVVVDKHDQGALWGSHPILALDVWEHSYYHDYGPARGDFIDAFFEVVDWEEPSARYEQAVE

>D3FDM4_469383_Actinobacteria_B_Ter

MAYELPALPYAYDALEPHIDEATQKFHHDKHHATYVARANEALEG-TEWADKPVEELLKNLDKLPADKLNPVRNNAGGHYNHTLWWESLSPNGGGAPTGDLAAAIDAAFGSFDEFKAKVEAAGAGRFGSGWAWLVKG-D-GGLEVTSTPNQDTPLADGKTPLFGIDVWEHAYYLKYQNLRPAYLKAVWNVVDWNKVAERFAAAS-

>C7R4P3_471856_Actinobacteria_B_Ter

GVYTLPELSYDYGALEPHISGRIMELHHSKHHQAYVTGANTALEQLAEARDKGDF---ATVN----QLEKNLAFNLGGHVNHTAFWENLSPEGG-EPEGDLAAALAEQFGSFEAFKKHFAAVAAGVQGSGWAILAWDSLGKKLIIVQLYDQQGNIPMGLTPIVLLDVWEHAYYLDYQNVRADYVTAWWNIVNWKDAGARFERART

>A0A2U2AEQ9_472582_Proteobacteria_B_Mar

MSFELPKLPYAMDALAPHISKETLEYHYGKHHQTYVNTLNTLIEGT-EFANASLEEIILKA-------SGPMFNNAAQVWNHTFYWESLKPQGGGAATGKIAEEINKKWGSFEEFQKAFDKCAIGTFGSGWAWLVKNAD-GSIELVSTSNAATPLTAGQTPLLTCDVWEHAYYIDYRNSRPNYLENFWALVNWDKVNERLG----

>C0BHJ2_487796_Bacteroidetes_B_Mar

MAFELPSLSYAYDALEPNIDSRTMEIHHSKHHNGYTTNLNNAIAG-TPLADQSIEEILKGLDLNN----KAVRNNGGGYYNHRLFWNIMSPNGGGLPTGTLATAIDSAFGSFDNFKSEFAKAAATQFGSGWAWLCVHVD-GTVKVCATANQDNPLMPGGTPILGIDVWEHAYYLNYQNRRPDYIQAFFNVIDWNKVNELFTKGA-

>D5BTR8_488538_Proteobacteria_B_Mar

MAFTLPDLPYSHDALAGLMSAETLEYHHDLHHNAYVVNGNKLVAGT-EWENKSLEDIITGTYQADAVAQNGIFNNASQHWNHMQFWEMMGPGKT-AMPSELEKALVESFGSVDAFNDAFKAAGASQFGSGWCWLVKNAD-GGLQVTKTENGVNPLCFGQTALLGCDVWEHSYYIDFRNKRPDYLSNFLNLVNWEFVAAQL-----

>A0A1I1CI47_490629_Actinobacteria_B_Ter

ALYELPELDYDYGDLAPHISGEINELHHSKHHATYVKGANDTMEKLAAARDAGDF---GNIV----GLETTLAFNLAGHANHVVWWKILSPNGGDKPTGELAAAIDESFGSFDKFKAQFTAVCTTIQGNGWGALSWDPIGKTLITQQLRDHHNNLILPTQPILLVDVWEHAFYLDYKNVKPDYVKALWNVFNWDEISKRFENAKA

>A0A0B7MKY0_499207_Firmicutes_B_Ter

GGHKLPPLPYPYNALEPVISKRTLRIHHDRHHLAYVEGLNKAELRLVEARRMGDF---SLVK----HWERELAFHGSGHILHSIYWTVMAPGGLREPGYYTCLEIEKYFGGFAPFKEQFTEASIQVEASGWGVLCWVPSWMRLEILTAEKHQNLTQWGAIPLLVVDAWEHAYYLDYQNRRADYVRAWWSLVNWNEVERRLRLAMR

>D0LUJ8_502025_Proteobacteria_B_Mar

MAFELPKLPYAEDALEPHISKETIQYHYGKHHQGYVNKVNAAVENT-PNADLSIDELVRKVAG--DAGSKKLFNSAAQVWNHTFYWNCLSPNGGGEPTGAIAEAINKSFGSFADFKSKFSDAAAGQFGSGWAWLIKGDD-GGVSIVTTSDAETPMAEGKTCVLTIDVWEHAYYVDYRNARPKYIEAFWNIVNWDFVNQQLA----

>A6EQH5_50743_Bacteroidetes_B_Mar

MAFELPKLPYAHDALAPHIDEQTMQIHHGKHHQGYTNNLNAAIEG-TDLEGKTIENILINLDMDN----KAVRNNGGGFYNHSLFWEVMSPNGGGEPTGEIMSAIKSSFGSFADFKEKFSTAAKTQFGSGWAFLCVKED-GSLDICGCPNQDNPLMPGGQPILGLDVWEHAYYLNYQNKRPDYVSAFFNVINWDEVNARFLKSK-

>D5UQ12_521096_Actinobacteria_B_Ter

AVYTLPDLDYDYSALEPHISGEIMELHHSKHHATYVAGANAALEQLAEAREDGSI---AKAP----LLSKNLAFHLGGHTNHSIFWKNLSPNGGDKPVGDLAAAIDEYFGGFDKFQAHFTAVANSLQGSGWSVLAYDHIGQRLVIQQLTDQQGNISINLTPLLMLDMWEHAFYLQYKNVKADYVKAWWNVVNWEDVAARYAAAKG

>C7RIZ7_522306_Proteobacteria_B_Mar

MEHQLPQLPFAMDALAPHMSRETFEYHYAKHHQAYVTNLNNLIKGT-EYEALDLEAIIRKAP------AGGVYNNSAQVWNHTFFWNCLTPNGGGAPGGALAAAIDAKWGSFAEFSKAFQTSAVGNFGSAWTWLVKKAD-GSVDIVNMGAAGTPLTTGDKALLCIDVWEHAYYIDYRNLRPKYVETFLSLANWRFAEQNFAG---

>C7RD31_523791_Proteobacteria_B_Mar

MAIELPALPYDRDALAPHISEETINYHYGKHHQAYVTNLNKMIDGT-DFAGKSLEDIIRTS-------EGGVFNNAAQVWNHTFYWNSLSPNGGGEPSGALADAINSAFGSFADFKEKFTASAAGNFGSGWTWLVKNSS-GNLEIVNTSNAGTPITDGVTPLLTVDVWEHAYYVDYRNARPEYLKHFWELVNWDFAAKNFA----

>C0W267_525245_Actinobacteria_B_Ter

AIYTLPELGYDYGALEPHISARIMELHHSKHHAAYVAGANLALEKLEAAREAGDH---AAIN----LHEKNLAFNLGGHSNHSVFWTNMTGEGASRPEGELAAAIDEFFGSFEKFQAQFAATAMGIQGSGWAILAWDTLGQRLVTFQLFDQQGNIPVGTVPLLMLDMWEHAFYLDYVNVKADYVSAWWNVVDWANVAARFTAARS

>D3SY04_547559_Halobacteria_A_Arc

TDHELPPLPYDYDALEPSISEQVVTWHHDTHHQGYVNGLNSAEETLAENRESGDF---DSTP----GALSNVTHNGCGHYLHTLFWENMSPDGGGEPAGDIADRIEEDFGSYEGWKGEFEAAAG--AAGGWALLVYDPVAKQLRNVAVDKHDQGALWGSHPILALDVWEHSYYYDYGPDRGSFIEGFFDVINWDSVEDEYQKCLD

>A0A2Z2L2V6_549298_Proteobacteria_B_Mar

MKFKLPKLPYAQDALEPVISKETIEYHYGKHHQTYVTNLNNLIEGT-EHSGQTIEEIIKAS-------SGGLFNNAAQVYNHTFYWNCFSPN-KTKPSNQLKVAIVETFGSFDKFKEEFSKTAVTTFGSGWAWLVKDSS-GKLEILSTSNAGCPLTNNKHPLLTFDVWEHAYYIDHRNARPKHVKALWDIINWEFVSEQFAK---

>E1SRJ0_550540_Proteobacteria_B_Mar

MAFELPALPYAKDALEPHISAETLEFHHGKHHATYVTKLNGLVEGT-ELAGKSLEEIVKTS-------TGGVFNNAAQIWNHTFYWHCLSPNGGGAATGAIADAINSAFGSFDAFKAQFTESAINNFGSSWTWLVKNAD-GSLAIVNTSNAATPLTEGVTPLLTVDLWEHAYYIDYRNARPNYMDAFWNLVNWDFVNENFA----

>H0TES2_551947_Proteobacteria_B_Mar

MTFTLPPLPYDYEALAPYMSKETLEYHHDKHHQAYVTNGNNAIKGT-EFEGKSLEEIVKGSYG----KNAAVFNNAGQHYNHIHFWQWMKKDGGDKVPGRLEKKINEDLGGFEKFKADFAAAGVGQFGSGWCWLQVK-N-GKLEISKTPNGENPLVHGATPILGCDVWEHSYYIDYRNRRPDYLKAFVNLINWDYVDELFDKTV-

>D8JZF0_552811_Chloroflexi_B_Ter

-------MGYEASGLTG-FSDKLLSNHF-TLYQGYVKNVNKLLETLQSLAGEAKG-GTPEYA----ELKRRFGFEWNGLRLHELYFGNLGGNGTPIPGGRLVKELTEQWGSFDAWETEFRATGS-LRGVGWAVLYQDTENGRLFNVWVDQHESGHLAGCRSILVMDVWEHAFMLDYGLKRPDYIAAFFKNINWSVCEKRLA----

>C8Q0X7_553217_Proteobacteria_B_Mar

MSITLPDLPYAKNGLEPHISAETLEFHHDKHHAAYVNKLNELLPGS-GLEGKELDEIIKATAD--DSSKATIFNQAAQVWNHTFYWNCLTANGGGEPTGDLKAKIEEAFGSYAKFREEFKNAAVSQFGSGWAWLVADSG-GKLSIIKTANADTPLAHNQVAVLTCDVWEHAYYIDYRNRRPDYVDTFLKLVNWDYANAKYKGQEA

>A0A1I2QXX4_553467_Halobacteria_A_Arc

STYELPDLPYDYDALEPVIDERIMELHHDKHHQGYVDGANSALEQLEQMRESGDM---GNVK----SVKRNLAFNLSGHVNHTVFWENMSPDGGGEPEGELADALDEQFGGFEQFKSDFAAASKGVEGSGWGQLVYDHAADQLMVVANENHQNQQVSGSTPILVLDVWEHAYYLQYENNRGEYVDSFWDVVDWEDVAQRYEQAQS

>A0A1H3W6N9_555874_Halobacteria_A_Arc

MSYELDPLPYDYDALEPHISEQVLTWHHDTHHQGYVNGWNSAEETLEANREDGDF---GSSP----GAIRNVTHNGSGHILHDLFWNSMSPEGGDEPTGDLADRIEEDFGSYEAWKGEFEAAAG--AAGGWALLVYDSFSNQLRNVVVDKHDQGALWGSHPILALDVWEHSYYHDYGPARGDFVDNFFEVVDWEEPSARYEQAAE

>A0A2A9CZS9_556530_Actinobacteria_B_Ter

MTYTLPELGYDYAALEPHISGKIMQLHHDKHHQAYVTGANTALEKLAEARSAGDL---AAVN----LHSKNLAFNLGGHVNHTVFWENMSPEGGE-PAGELLAMLQDSFGGVEAFKKHFAAVAAGVQGSGWAVLAWDTVGANPVIVQLYDQQANLALGLVPLLMLDVWEHAYYLDYLNVRADYIAAWWNIVNWHDVAARLERATS

>A0A1H6CZE7_568106_Proteobacteria_B_Mar

MSFELPALPYAKDALEPHISAETLEFHHGKHHNTYVTKLNGLVPGT-EFEGKTLEEIITSA-------PAGVFNNAAQIWNHTFYWYSLSPNGGGAPTGAIADAINAKWGSFEKFQEEFNDKAVNNFASCWTWLVKNSD-GSLEIVNTSNAGTPMTNGQKALLTVDLWEHAYYIDYRNVRPDYLKGFWALVNWDFANENFA----

>A0A256FXP0_571255_Proteobacteria_B_Mar

MAFELPPLPYDYDALAPFMSRETLEFHHDKHHQAYVTNGNKLLEGS-GLEGKSLEEIVKESFG----KNQALFNNAGQHYNHLHFWNWMKKDGGGKLPGNLEKAIESDLGGYDKFREDFIAAGAGQFGSGWAWLSVK-N-GKLEISKTANGESPLVHGATPILGVDVWEHSYYIDYRNARPKYLEAFVSLINWDYVSELYEKA--

>E1QRN4_572478_Thermoprotei_A_Arc

KRYELPPLPYSINALEPHISGQVIDVHYNGHHKGYVNGANATIERLEKIIKGDVTS--YDIQ----GLLRSLFFNVNGHKLHTLYWYSMAPPGGGTPGGYLGDLIKKQFGSFDRFKALFTEVMRSLPGCGWTVLYYDPETGNLEFTTFENHYNQHIAELPILLIVDEFEHAYYLQYRSNRNGYIDAIWNVLNWEEAENRLRKYIK

>E8QXM5_575540_Planctomycetes_B_Mer

MAYTLPALPYAYDALEPHIDARTMEIHHTKHHQAYITNLNNAIKDQPALQDLPIEKLIADLGAIPEAIRTTVRNNGGGHANHALFWQIMSPNGGGQPVGKLAAAIEGELHGFDSFKDAFSKAGLNRFGSGWAWLALDPS-KKLVITSTPNQDSPIMEGMIPLLGMDVWEHAYYLKYQNRRADYIAAFFNVINWPMVDELYVKAMG

>D7JFA1_575590_Bacteroidetes_B_Mar

MKHELIVLPYPANALEPVISETTINLHHGKHLATYVNNLNNLIQGT-KFENADLETIVRES-------DGALFNNAGQTLNHNYYFTQFAPKSEGRPSGRLLKAIEEAWGSFENFRSEFETAATSLFGSGWAWLAADKE-GKLQITKEQNAGNPVAKGLKPILGIDVWEHAYYLDYQNRRADHLKELWKIISWAEVEKRY-----

>A0A0S2JHC6_58049_Proteobacteria_B_Mar

MAIELPALPYEQNALEPHISAETLSFHYGKHHNTYVVKLNGLIGGT-EFENKSLEEIVKSS-------SAGIFNNAAQIWNHTFYWNSLSPNGGGEPSGDLLAAINSNFGSFDEFKAKFTDSAINNFGSSWTWLVQNAD-GSLAIVNTSNAATPLTDGVTPLITVDLWEHAYYIDYRNVRPDYLKGFWALANWDFAQANFS----

>A0A1Z4VNQ5_585455_Proteobacteria_B_Mar

MTHTLPDLPYPGNALEPHISAETLEYHHGKHHATYVANLNKLIEGT-EYADKALEDIIRSAP------AGGLFNNAAQVWNHTFYWNCMSPNGGGEPAGALADAIKAAFGSFADFKEKFSTSGATNFGSGWTWLVQKSD-GSVDIMNTSNAGCPLTEGVTPLLTLDVWEHAYYIDYRNARPKYIEAFWNVVNWDFVAGNFQG---

>D4YMB9_585530_Actinobacteria_B_Ter

EQYTLPDLPYDYAALEPHISAKIMELHHDKHHATYVKGANTALEQMAEAREKGDF---STIG----KLSKDLSFNLGGHVNHSIFWNNMSPDGGDKPEGELAAAIDDQFGGFDKFQGQFTGVATSIQGSGWAILGWDMLGQRLTIEQLYDQQGNVQVGYVPLLQLDMWEHAFYLDYQNVKPDYVKAWWNVVNWEDVAARFDRARN

>A8PPX3_59196_Proteobacteria_B_Mar

MKHQLPPLPYSLEALAPIISKETLEYHYGKHHKAYVNKLNELIPNT-TFETLSLENIIKKA------PKGPIFNNAAQTWNHTFYWHCMTPLAKNKLKGALEESICKSFGSFSQFKEAFTNAAIAQFGSGWAWLIKDAH-G-LKIETTSNALTPLSEDKICLLTCDVWEHAYYIDYRNARPHYLEKFWDIVNWDFVAENFEKQ--

>G7LTJ2_598467_Proteobacteria_B_Mar

MSYSLPSLPYAYDALEPHFDKQTMEIHHTKHHQTYVNNANAALESLPEFAELSAEELLTKLDKVPAEKKTVLRNNVGGHANHSLFWKGLKL--GTTLAGDLKAAIERDFGSIDTFKEKFEQAAATRFGSGWAWLVLKDD-GKLAVVSTANQDSPLSGSGYPILGLDVWEHAYYLKYQNRRPDYIKAFWNVVNWDEAAKRFAEAKK

>D0J9E3_600809_Bacteroidetes_B_Mar

MSFKLPKLSYSYKDLEPYIDQKTMEIHYTKHHAGYTNNLNKAIEG-TDLTHLSIEEILRRSNI----EKPIIRNNGGGFYNHNLFWEILIPHDYTHPSKYLKNAIEKNFKSFDAFKEKFTSIAMNRFGSGWTWLCVK-E-KRLTICSTANQDNPVMLEGVPILGLDVWEHAYYLQYQNRRADYISSFWNIINWKKVEENYKKSIE

>E1QXA5_633147_Actinobacteria_B_Ter

--FEQIKLPYAYDALEPYVDALTMETHYSKHHTTYTNALNAAAEK-AGVADQPIERLLSSLDGIDEPLRGTIRNNGGGFYNHNLYFSIMSPAPAAEPAGVLRERIEEAFGSVGELKAELKKAAVGRFGSGWAWLNAAPD-GTLSVTSSPNQDNPFSEGVTPILGIDVWEHAYYLKYKNLRGDYVDAFFSVLDWDAVAANYAKATA

>A0A238VTU4_63740_Halobacteria_A_Arc

MSYELDPLPYDYDALEPHISEQVLTWHHDTHHQSYVNGWNAAEETLAANREEGDF---SSSG----GALRSVTHNSSGHILHDLFWQNMSPEGGDEPEGALADRIEEDFGSYEAWKGEFKAAGG--AASGWALLVYDTFSNQLRNVVVDKHDQGAIWGGHPILALDVWEHSYYHDYGPARGEFIDNFFEVVDWEEPSARYEQAVE

>V9H8J0_641147_Proteobacteria_B_Mar

MAYTLPELGYAYNALEPHFDAQTLEIHHSKHHQTYVNNANGVRETLPEFQDLPVEEMMKRIGELPADKQMPARNNIGGHANHSFFWTLLKT--GTELKGSLKEAIERDFGSVDAFKEAFEKAAQTRFASGWAWLVVE-N-GKLAIVSTPNQDSPVMGSGTPLLTLDVWEHAYYLKFQNRRPEYIKTFWNVVNWDEVQRLYDSAN-

>E6SLN6_644966_Firmicutes_B_Ter

MAYQLPPLPYDYNALEPHIDEQTMRIHHDRHHATYVNNVNAALEKYPALQNKPIEELLRQIDQVPEDIRTAVRNNGGGHANHSLFWEIMSPRGGGQPSGALAEAINRSFGSFDAFKDQFTKTATTHFGSGWAWLVVDEK-GELQVYSLPNQDSPYMKGHTPILGLDVWEHAYYLKYQNKRPDYIAAWWNVVNWDEVARRYQQARG

>A0A4R2GX74_659006_Proteobacteria_B_Mar

MTFKLPELPYAYDALQPYMSKETLEYHHDKHHQAYVDTGNKLLAGT-ELEGKSVEEIVKASYG----KNQPLFNNAGQHYNHLHFWNWMKPNGGGAIPGAIEKKIVSDIGSVEKFKEDFVQAGVGQFGSGWAWLALK-N-GKLEIMKSPNGENPLVHGATPILGVDVWEHSYYIDYRNRRADYLKAFVNLVNWEYVEKLFNEAT-

>A0A1H3ILX3_660517_Halobacteria_A_Arc

MSYELDPLPYDYDALEPHISEQVLTWHHDTHHQGYVNGWNAAEETLEENREAHDF---SSSA----GAIRNVTHNSSGHILHDLFWNSMSPEGGDEPTGALADRIEADFGSYEAWKGEFEAAAS--DASGWALLVYDSFSNQLRNVVVDNHDEGAVWGGHPVLALDVWEHSYYHDYGPARGDFVDNFFEVVDWEEPSARYEQAVE

>D9Q0R7_666510_Thermoprotei_A_Arc

KRYELPPLPYNYDALEPIISAETLRYHHDKHHLGYVNGANAALDKLEKYLNGQLTD--IDVR----AVSRDFEFNYGGHILHTLYWLNMAPKGGGTPGGAIGDAINKFFGSFDKFKKLFGDAAKNVEGVGWAILAYDPVTGDLRILQVEKHNNVVTTNLIPLLAVDVFEHAYYIDYRNDRAKYVDSWWDLINWDDVEARYQKALN

>T0M4N9_667135_Thermoplasmata_A_Arc

MAENWQ----IKDKLKPSISDKQIDYHFETHYKGYVNKLNEIWEKLPNADRSKANQNYSEFR----ELKLEETFNFDGSLLHETYFANLAKE-HAPVSESFKKQVEKDFGSYEKWVEDFKATGV--AFRGWSLCVFDLNTGKLRNIGADVHNTNGIWNAILVLALDVYEHAYYTDYGPKRAPYLDAFLKNVKWSDVEQRLEKAKK

>T0MPA5_667137_Thermoplasmata_A_Arc

MTEKWE----KKTTLKPKISDQQIEYHFETHYNGYVTKLNEIWEKMQTVDRSKANQNYSEFR----ELKLEETFNYDGSLLHELYFENLHSG-SSSAPESFKSQVAKDFGSYEKWLEDFKATGI--AFRGWSLLVFDLNTGKLRNIGADVHNTNGIWNAIVILSLDVYEHAYYTDYGPKRAPYLDAFMKNVNWSEVEKRLQKAQK

>T0LM88_667138_Thermoplasmata_A_Arc

KKYELPQLPYRIDGLEPHISKDIVDVHYNGHHKGYVNTANNLIDRLNGIVKEEVKS--YDIH----GVLRNLTFNINGDKLHTLYWNNMAPEGGDKPGGKLGDLIEQQYGSYEKFRKLFTEAANSNPGTGWAVLTYDKENSNLNVMTVENHFMNHLAEMPIVLILDEFEHAYYLQYKNKRADYVGAWWHIVNWDEANKKLEKLL-

>H2C9I7_671065_Thermoprotei_A_Arc

KKFELPPLPYKVDSLEPYISKDIIDVHYNGHHKGYVNGANQFMERFNKVIKGELQAGQYDVQ----GLMRGLVFNINGHKLHALYWDNMAPAGGGKPGGALADLIEKQYGGVERFKQVFTETANSLPGTGWTVLYYDTENGNLEIMTFENHFMNHIAELPILLILDEFEHAYYLQYKNKRADYVTNWWNVVNWDYAEKKLQKYLR

>F7XLC9_679901_Methanomicrobia_A_Arc

EYYKLPPLAYGYDELEPYISEEQLRLHHDKHHQSYVDNLNSIIKMVEKAREEGKD---YDYK----AATKAASFNAGGNVLHDYFWWEMMPAAGEEPVGELLEQIKKDFGSFERFKKEFSQVALTVEGSGWAALTYCGDTHRLSPIQIEKHNVNVYPDYPIIMVLDMWEHAYYIDYRNEKAKFVDGFWNIINWEELDKHFKKYL-

>G8NWQ2_682795_Acidobacteria_B_Mar

MAFELPPLPYDYAALEPTIDEATMKLHHDKHHQTYVTNLNGAVEKHPDLGKKTPEELIKDLDSIPEDVRGVVRNNGGGHVNHTMFWQIMGPNGGGEPTGEIAAQIKADFGSFEDFKKKFNETTAKQFGSGWGWLVFK-G-GKLEIVTTANQDNPLSQGLYPILGNDVWEHAYYLKYQNKRPDYLAAWWNVINWAEVNKRFEHAKK

>A0A166QH71_683316_Actinobacteria_B_Ter

AVYTLPDLQYDYAALEPAISGQIIELHHDKHHATYVAGANTTLEKLAEARDKGDF---GAIV----GLEKTLAFNVSGHILHSIYWQNLSPDGGDKPDGALADAIAESFGSFDNFKAQLTAATTTVQGSGWGILAYDQLSGRLLVEQVYDHQSNVGIGNTTLLAFDAWEHAWYLQYKNVKADYVTALWDIVNWADVAARYATAKQ

>D0RR50_684719_Proteobacteria_B_Mar

MAFELPKLNYANDALAPVMSQETLDLHHGKHHQTYVTNLNNLVKDT-DLADASLEDIIKKTAK--DSSKAGIFNNAGQHWNHILFWKCMKPKGGGAIPSELEKRIVSDFGSVDKFKEDFIQAGVTQFGSGWAWLAID-N-GKLVITKSANASNPLVDNMKPILGCDVWEHSYYVDYRNRRPDYLKAFIDLVNWEHVASLLD----

>F2KQT8_693661_Archaeoglobi_A_Arc

SRYVLPELPYEYNALEPYISEEILKLHHDKHHSAYVKGANAALEKLEKARKGEIE---VDIK----AVLKELSFHVGGHILHTIFWNCMTPE-KGEPSGVLAEKIKEEFGSVERFKDEFSKAANSVEGSGWAALMYCPLTGRLIIQQIEKHNVNLAPGLHILACIDVWEHAYYLQYKNDRASFVKNWWNVVNWNFIEERLKEAMK

>A0A1H6AL08_699433_Halobacteria_A_Arc

MSYELDPLPYDYDALEPHISEQVLTWHHDTHHQGYVNGWNAAEETLEENREAGDF---SSSA----GAIRNVTHNGSGHILHDLFWNSMSPEGGDEPTGALADRIEEDFGSYEAWKGEFEAAAS--AAGGWALLVYDSFSNQLRNVVVDKHDQGALWGSHPVLALDVWEHSYYHDYGPARGDFVENFFEVVDWEEPSARYEQAVE

>D6YU13_716544_Chlamydiae_B_Mar

NTYTLPDLPYDLGDLEPVINAEIMDLHYNKHHKTYINNLNNLLEQLEEAQSKKDI---SKEI----SLQSGINFNGGGYINHSIFWTNLAPKGGGEPSGPLADAIMKDFGSLDKLKETMTAKTVAVQGSGWGWLGYNKSEDRLEIAICQNQDPLAAKGLVPLLGIDVWEHAYYLQYKNVRPDYVKAIWEIVNWKNVSERYEAAL-

>A0A259TZ62_716817_Rhodothermaeota_B_Mar

MAFELPDLPYSYDALEPHIDEQTMRIHHGKHHAGYTTKLNAALEG-TEWADQPIEETLANLDALPEDKQTAVRNNGGGFLNHGIFWTTMSPSGGGEPTGDLAKAIDEAFGSFDAFKEKFSEAAKGQFGSGWAWLELASD-GSLQVHGHANQNNPVMHGAKPIIGLDVWEHAYYLKYQNKRPDYVSAWWNVVNWDEADRRYRSYNA

>K1JSW8_742823_Proteobacteria_B_Mar

-MFTLPALPYALDALEPAMSRETLEFHWGKHHQTYVNNLNGLIAGT-DFEGKSLEEIIRTS-------TGGVFNNAAQVFNHTFFWQGLKPQGGGMPEGALFEAINAIWGSYDAFRKAFTASAAGNFGSGWTWLVKNAD-GTLAIVNTSNAGTPLTGDQKPLLALDVWEHAYYVDYRNARPKFIDAFFSLVNWEFAQQNFAA---

>A0A2Z2HUJ4_745377_Halobacteria_A_Arc

TDHELPPLPYDYDALEPSISEQVVTWHHDTHHQGYVNGLNSAEETLAENRESGDF---DSTP----GALSSVTHNGCGHYLHTLFWENMSPDGGGEPAGDLAERIEEDFGSYEGWKGEFEAAAG--AAGGWALLVYDPVAKQLRNVAVDKHDQGALWGSHPILACDVWEHSYYYDYGPDRGSFVEGFFDVVNWDKVDEEYQKCID

>K2JEV8_745411_Proteobacteria_B_Mar

MAFELPALPYEKNALEPHISAETLEYHYGKHHNTYVVNLNNLTAGT-ENEGKSLEDIIKSS-------TGGLFNNAAQVWNHTFYWHCLSPNGGGAATGAVADAINAAFGSFDAFKEEFTKNAIGNFGSGWTWLVKKAD-GSVGIHNTSNAGCPITEGVTPLLTVDVWEHAYYIDYRNARPKYMEAFWALVNWDFVNKNLA----

>H0PZQ1_748247_Proteobacteria_B_Mar

MEHTLPPLPYSKDALAPHISAETMEFHYGKHHQAYVTNLNNLIKGT-EYENLDLEAIVKKAP------AGGVYNNSAQVWNHTFFWNSMKPNGGGEPTGALGDAIKAKWGSFEDFKKAFTASAVGNFGSGWTWLVKKAD-GAVDIVNMGAAGTPLTTGDKALLCIDVWEHAYYIDYRNRRPDFVATFLSLANWDFAAKNFAG---

>K9Z4N7_755178_Cyanobacteria_B_Ter

MAYQLPNLPYEYNALEPYISKSTLEFHHDKHHAAYVNKFNDAVAGT-ELDNQPIETIIKKFAE--DTSKQGIFNNAAQAWNHSFYWQCMKPNGGGNPTGVLADKINTDFGSFEKFIEAFKNAGATQFGSGWAWLVLD-G-DTLKVTKTPNADNPFTKNQIPLLTMDVWEHAYYLDYQNRRPDYINDFIKLVNWDFVAQNFSEAL-

>D8J2G3_795797_Halobacteria_A_Arc

SNAELPPLPYDYDALEPSISEQVVTWHHDTHHQGYVNGLNSAEETLAENRESGDY---SSTG----GALGNVTHNGSGHYLHTLFWENMSPEGGDEPSGDLRERIEEDFGSYEGWKGEFEAAAK--AAGGWALLVYDPVAKQLRNLAVDKHDQGALWGAHPVLALDVWEHSYYYDYGPDRGDFIDAFFDVVNWDEAEDQYQKSVD

>W0JR24_797299_Halobacteria_A_Arc

TDNELPPLPYDYDALEPSISEQVVTWHHDTHHQGYVNGLNSAEETLAENRESGDF---GSTP----GALSNVTHNGCGHYLHTLFWENMSPNGGGEPSGDLADRIEEDFGSYEAWKGEFEAAAG--AAGGWALLVYDPVAKQLRNIAVDKHDQGALWGSHPILALDVWEHSYYYDYGPDRGSFIDSFFDVVNWDKAAEEYQTCLD

>L0IC00_797302_Halobacteria_A_Arc

TEHELPPLPYDYDALEPSISEQVVTWHHDTHHQGYVNGLNAAEETLAENRESGDF---GSSP----GAIGNVTHNGCGHYLHTMFWENMSPNGGGEPEGDLADRIEEDFGSYEGWKGEFEAAAG--AAGGWALLVYDPVAKQLRNIVVDKHDQGALWGSHPILALDVWEHSYYYDYGPDRGDFIDSFFDVINWDSVAEEYQKCLD

>L0JLZ0_797303_Halobacteria_A_Arc

ADHELPPLPYDYDALEPALSEQVLTWHHDTHHQGYVNGLNSAEETLAQNREEGDF---GSTP----GALSDVTHNGCGHYLHTLFWENMSPNGGGEPEGDLADRIEEDFGSYEGWKGEFEAAAG--AAGGWALLVYDPVAKQLRNVAVDKHDQGALWGSHPILALDVWEHSYYYDYGPDRGEFIDGFFDVVNWEKAEEEYQTCLD

>L0AKN7_797304_Halobacteria_A_Arc

TDHELPPLPYEYDALEPAISEQVVTWHHDTHHQGYVDGLNSAEAELAENRESGEF---GSTP----GALSNVTHNGCGHYLHTLFWENMSPDGGGEPEGELADRIEEDFGSYEGWKGEFEAAAG--AAGGWALLVYDPVSKQLRNVAVDKHDQGALWGAHPILACDVWEHSYYYDYGPDRGSFIEGFFDVVDWDKVDEEYQKCLG

>A0A090IF69_80854_Proteobacteria_B_Mar

MSITLPALPYAQDALEPHISAETLSFHYGKHHNTYVVKLNGLIEGT-PLAEKSLEEIVKSS-------EGPVFNNAAQVWNHTFYWNSLTPNAKGQPEGALADAINAKFGSFEAFQEAFNDKAVNNFGSSWTWLVKNAE-GELEIVNTSNAGTPITEGVTPLITVDLWEHAYYIDYRNLRPSYLKGFWALANWDFAAANFAA---

>E1VHB8_83406_Proteobacteria_B_Mar

MSFELPALPYAKNALAPHISEETLEFHYGKHHNAYVTNLNNLVKGT-EFEGKSLEEVVRKS-------SGGIFNNAAQVWNHTFYWNSLSPKGGGAATGAVAEGINKAFGSFDKFKEEFSKSAAGNFGSGWTWLVKKAD-GSLAIVNTSNAATPITEGVTPLITVDVWEHAYYIDYRNLRPKYLEAFWALVNWDFANANLAK---

>A0A1G8BH66_83767_Proteobacteria_B_Mar

MEHQLPQLPYAIDALAPHMSKETFEYHYAKHHQAYVTNLNNLIKGT-EYEALDLEAIVKKAP------AGGIYNNSAQVWNHSFFWNCLKPNGGGAPSGALAEAINKKWGSFDEFKKAFQASAVGNFGSGWTWLVKKAD-GSVDIVNMGAAGTPLTTGDKALLCIDVWEHAYYIDYRNLRPKFVETFLSLANWSFAEKNFG----

>G2KRC6_856793_Proteobacteria_B_Mar

MALILPELPYAYDSLQPFMSSETLEFHHDKHHKAYVDKGNELLAGS-GLENLSLEEAMVAAFK--DKSKAGLFNQLGQHYNHIHFWNWMKKNGGGSIPGKLDAQITSDLGGFDTFRGNFIQAGMTQFGSGWCWLAVDAT-GKLEITKTANGENPLVYGKTPILGCDVWEHSYYIDYRNARQKYLEAFVSLINWDYVGQLFEKGPL

>A0A0C1QWB0_86105_Proteobacteria_B_Mar

NNFTLPQLPYAKNALEPYVSEETLSFHHGKHHQTYVTNLNNLIKDS-PFAEMGLEDIIEKSYQ--KADKVGIFNNAAQVWNHAFLWHCMKKDGGGLPSKTLQDKINEDFGSMEKFVEEFKNAAITQFGSGWAWLVLD-K-DKLKVIKTGNAELPMVLGQKALLTIDVWEHAYYLDYQNRRADYVNTFIKLINWQFVEENFNSR--

>E1X0Y3_862908_Proteobacteria_B_Mar

MAHELPKLPWADNALEPHISAETISFHYGKHHNAYLTKLNAAIPGT-EYESMTLEETIMKS-------EGGLFNNAAQVWNHSFYWNCLAPNAGGAATGAVAEKINAKWGSFEKFQEDFTNAAATNFGSGWTWLVEE-G-GELEIFNTANADTPMKHGKKALMTIDVWEHAYYVDYRNARPNYIEAFWKLVNWDFVNSNL-----

>A0A0X3BJM4_86622_Methanomicrobia_A_Mar

KKYELPPLPYAPDALEPHISKEQLSLHHDKHHQAYVTGANANLERLEKARREGTD---VDMK----ALLKELSFNIGGHILHTLFWPGMAPAGGGTPGGALADLIDREWGSFDRFKAEFSKAAASVEGSGWAALAYCTMTDRPMIMQIEKHNNNVYPTFQILMVLDVWEHAYYVDYKNNRGQFVDAFWNVANWGEVNRRLEKI--

>L0KZ58_867904_Methanomicrobia_A_Arc

ELYKLPALKYGYADLEPYISEEQLRIHHEKHHQAYVTNVNSLLQMMEKARREGTD---FDYK----ATAKAVAFNLGGHVLHDYFWWEMTPAATKEPVGELAEVIKEDFGNFERFKKEFTQTAASVEGSGWAALTFCNDTKRLGIMQIEKHNVNLFPDFPIIMAIDVWEHAYYLDYKNERGKFIDAFWNIVNWEEIDRYFRKVQQ

>A0A0M9UDM4_872965_Chloroflexi_B_Ter

MAHELPALPYAYDALEPYIDARTMEIHHTKHHQGYVNNLNKALEAYPHLQDKSVEELLRNIESVPEDIRTAVRNNGGGHANHSLFWTIMAPNAGGAPSGELAAAIDAAFGSFDAFKEAFSKAAATRFGSGWAWLVVTAF-GELKVYSTPNQDSPYMFGHTPILGLDVWEHAYYLNYQNRRGDYIGNWWNVVNWDKVAEYYAAAKK

>A0A0K6IWK3_876478_Proteobacteria_B_Mar

MEHKLPELPYPKNALAPYISEETMEYHYGKHHQAYVTNLNNLIKGT-EYENMDLESIVKKAP------AGPIYNNAAQTWNHTFFWHCMKPNGGGEPKGALLDAIKAKWGSFDEFKKVFHTSAVGNFGSGWTWLVKKAD-GSVDVVNMGAAGTPLTTGDTPLLCIDVWEHAYYIDYRNRRADFVTAFLHLVNWEFAERNYAA---

>F0TCH4_877455_Methanobacteria_A_Arc

KNYQLPELPYGYKDLEPYISEEQLKIHHTKHHKAYVDGANAILKKFDN-R-GSEE---FDVK----AVSKELTFHVGGYVLHKFFWENMGPACGGEPTGTIAEYIKKDFGTFERFKKEFSQAATGVEGSGWAVLTLCRMTNRIFIMQVEKHNVNIIPGFRIMLALDVWEHAYYLDYQNRRPDYVEAFWNLVNWEEVNRKMDVWLS

>A0A212PXD1_877466_Chloroflexi_B_Ter

MAHTLPPLPYPYDALEPYIDAQTMEIHHTRHHQAYVNNLNAALEKYPELQDVPVETLLRHLPSVPEDIREAVRNNGGGHLNHSLFWTLMKPGGGGEPSGALAEAIAKAFGSFANFKELFTKAAMGRFGSGWAWLSMTAF-GKLVVHSTPNQDNPVMEGLFPLLGLDVWEHAYYLKYQNRRADYVQAWWNVVNWDQVAANYAKALE

>W0ERB5_880074_Bacteroidetes_B_Mar

MIYQLPPLPYGTNDLAPVISQETIEYHYGKHEQTYLDNLNRLIDGT-PYAEKPLEEVIREA-------EGALFNNASQAWNHIFYFFTFAPDGRRKPQGKLAEAIDRQWGSLENFQKEFEQAGVTLFGSGWVWLSKDPE-GKLVISQESNAGSPLKKGYTPLLTFDVWEHAYYIDYRNRRAEHLHRLWEIVDWSVVEARYL----

>S8FFI4_888054_Bacteroidetes_B_Mar

MKVELPRLPYAANALEPVISAQTILLHHGKHFQNYINTLASLVKGT-EFENKSIEEIVQTV------PDGPIFNNAGQSLNHAFYFSQFSPVSNNVPHGKMAKAIDVAFGSFEEFKKQFSQAAATLFGSGWAWLSQDKD-GNLVITKESNAGNPFRRGNNPLFGLDVWEHAYYLDYQNRRADHIAAVWEIVDWEIVEKRLK----

>A0A1G8W4P7_890420_Halobacteria_A_Arc

SNPELPELPYEYDALEPHISEQVVTWHHDTHHQGYVNGLDSAEETLAENREDGDF---GSSG----SAMRDVTHNGCGHYLHTLFWENMHPDGGGEPEGELRDRIEEDFGSYEGWKGEFEAAAG--AAGGWALLVYDPVAKQLRNVVVDKHDQGALWGAHPVLALDVWEHSYYYDYGPDRGEFIDNFFEVVNWDKAAEEYETCLA

>G6A1M0_909943_Proteobacteria_B_Mar

MAFELPELPYAYDALAAGMSQETMEFHHDLHHKAYVDNGNKLIAGT-EWENSSLEQIITGTYQSGAVAQNGIFNNASQHWNHIQFWEMMGPSGR-QMPSELDAALNAQFGSIDAFKEQFVAAGVGQFGSGWCWLVQNAD-GSLAITKTENGVNPLCFGQTALLGCDVWEHSYYIDFRNKRPAYLTNFLNLVNWENVAARMAG---

>A0A085G4I0_910964_Proteobacteria_B_Mar

MSYSLPSLPYAYDALEPHFDKQTMEIHHTKHHQTYVNNANAALESLPELAKLSVEELIANLDKVPADKKVALRNNAGGHANHSLFWKGLKT--GTTLQGDLKAAIERDFGSVDAFKETFEKAATTRFGSGWAWLVLKTD-GKLAVVSTANQDSPLMGSGYPLLGLDVWEHAYYLKYQNKRPDYIKAFWDVVNWDEAAKRLAEAK-

>A0A3M0CGB4_911205_Proteobacteria_B_Mar

MSFELPALPYAQDALEPHISANTLSFHHGKHHNTYVVNLNKLVEGT-EYDGKSLEDIMKATAG--QADKAGIFNNAAQVWNHTFYWHSMSPNGGGSPSGDLAAKIDEDFGGYDAFADAFKAAGATQFGSGWAWLVLD-G-GKLAVVKTPNAECPLTDGAVPLITMDVWEHAYYLDYQNARPAYMETFLKLVNWEFAARNLAAAA-

>I0I4H8_926550_Chloroflexi_B_Ter

MAFTLPDLPYPEDALEPYIDARTMNIHRTKHHQAYVTNLNNAIAG-TEWEKLSIEEILRRIDEVPENIRTTVRNNGGGHANHSLFWKIMAPNAGGAPTGALAEAINAKWGSFDEFKAQFKAIAVGRFGSGWAWLVVKPD-GSLDAYSLPNQDSPYMQGDTPILGLDVWEHAYYLHYQNRRPDYVDAWWNVVNWDEVAKNYAAAKG

>G8R729_926562_Bacteroidetes_B_Mar

MSFELPDLPYSKSALEPYIDAQTMEIHHDKHHAGYTTKLNDAIEG-TELEKQSIEDILKNVSKHS----GGVRNNGGGYYNHSLFWSIMGPDAGGDPTGDVGAAIDDAFGSYENFKTEFSNAAATRFGSGWAWLIVNGE-GKLEVTSSPNQDNPLMDKGTPILGLDVWEHAYYLKYQNKRPDYISAFFNVINWDEVNRRFAEAK-

>A0A0F6YK52_927083_Proteobacteria_B_Mar

MAFTLPELPYAKDALAPHISAETLEFHHGKHHATYVTNLNKFVDDP-SLQGKSLEDIIRTS-------KGPVFNNAAQVWNHTFYWHSMRPGGGGEPTGAIKGAIDKSFGSFATFKEQFSAKAVGHFASGWAWLVKNGD--KLEIVDTHDAGTPLTMDRKPILTCDVWEHAYYVDYRNARAKYVESWWNLVNWDHANSQL-----

>M0MDJ5_931277_Halobacteria_A_Arc

SNPELPPLPYDYDALEPHISEQVLNWHHDTHHQGYVNGLDSAEETLAENRESGDY---SSTG----GALGDVTHNGSGHYLHTMFWENMSPNGGGEPSGDLADRIEEDFGSYEGWKGEFEAAAS--AASGWALLIYDPVADQLRNVAVDNHDEGALWGAHPVLALDVWEHSYYYDYGPDRGSFIDAFFEVVDWDAAEDNYQKTVS

>F4B3P9_933801_Thermoprotei_A_Arc

KKYELPPLPYNLDALEPYISKEIIDVHYNGHHRGYVNGANSFVDRVNKILKGEISSGQYDIQ----GLLRGLVFNINGHKLHSLYWQNMAPAGGGKPGGVIGDLIEKQYGSFEKFKALFTEAANSLPGTGWAVLYYEVENGNLQIMTFENHFQNHIAELPILLILDEFEHAYYLQYKNKRADYVNNWWNLVNWDFADKKLQQYMK

>W3Y1G4_936589_Firmicutes_B_Ter

KPFELAPLPYAYDFLEPVIDKETMQIHHDKHHQAYVNNLNAAIAKYPDLPYGCVDCILGDIANVPEDIRQAVINNGGGHKNHTLFWEIMTKPDSSTLSGDLKDAIDRDLGGYDAFVEAFSNAAATRFGSGWAWLVVNKD-RKLEVTSTANQDNPLLEGKTGILGLDVWEHAYYLHYQNRRPDYIKEFFCVINWDKVSENYTRALN

>A0A3N1MAT8_94_Proteobacteria_B_Mar

MAFELPPLPYAPNALEPHMSANTFSFHHAKHHQAYVTNLNNLIKDT-PLADKSLEEIILATAS--DASKAGVFNNAAQVWNHTFFWNSMKPAGGGAPSGDLAKKIDAAFGSLAKFKEEFKAAAVGQFGSGWAWLVLD-G-SDLKIVKTGNAGTPMTSGQKALLTVDVWEHAYYLDYQNRRPDFVQTFLHLVNWDFAAKNLG----

>I0AK38_945713_Ignavibacteriae_B_Mar

GKFELPPLPYSYDALEPYIDKMTMEIHHTKHHQAYINNLNKAIEG-TDMEKMTMEEMFASVSKLP----VAVRNNGGGHWNHSLFWTLMKPNGGGKPSGALADAISAQFGSFDDFKKKFSDAAAGRFGSGWAWLVKA-D-NKLIVTSTPNQDNPLMDKGSPILGLDVWEHAYYLKYQNRRPEYIENWWNVVNWDKVAELFSKAK-

>A0A4P8L1J4_980445_Proteobacteria_B_Mar

AVFKLPSLPYPEDALEPFVSARTLSFHYGKHHQGYVNNLNNLVKGK-AFERMSLEEVIVKTAG--DSGQASVFNNAAQVWNHTFYWNSMQKDGGGPPQGPVAAKISEAFGSYENFRKEFAKAAATVFGSGWAWLVLE-N-GKLKITQTSNADTPAAHGQKAVLTIDVWEHAYYLDYQNQRVDYIQTFLHLLNWDFVAQNLE----

>F4BZF7_990316_Methanomicrobia_A_Arc

KKYTLPELPYAGNALEPHITQAQLVLHHDKHHAAYVNGANAILERLDKARESGSD---IDMK----ATLKELSFQAGGHVLHSLFWANLAPAAKKEPEGALAEALKKEFGSIERFKKEFTTAAVSTEGSGWAALSWCGMTGRPIIMQIEKHNVNVYPMFRILMVLDVWEHAYYLDYKNERAKFVEAFWNIVNWDEVNRRLEAVIK

>F2IV67_991905_Proteobacteria_B_Mar

MSFQLPDLPYAYDALGPYMSAETLEYHHDKHHLAYVSNGNNLLKDS-GLEGKSVEEVVKESFG----KNPGLFNNAGQHYNHIHFWKWMKPNGGGKLPGALASKIDSDLGGYDKFRADFINAGITQFGSGWAWLALK-D-GKLAIMKTPNGENPLVHGAAPLLGVDVWEHSYYIDYRNLRPKYLEAFVNLVNWDYVLEMYEAAA-

>A0A4R7JC83_993414_Actinobacteria_B_Ter

MTYELPDLPYDYGALEPHISAKIMELHHDKHHATYVKGANTALEKLDAAREAGDY---ATIN----QLTTDLAFNLGGHVNHSIFWNNLSPDGPDRPVGNLEVAIDEFYGSFADFQAAFTAAATGIQGSGWGILAWDQLGKRLNIFQLRDQQAQVPLNQTPIVLLDMWEHAFYLDYQNVKPDYVKAWWNVVNWTDAAERFENAQS
